# Supplementary material for: What is the effect of secondary (high) schooling on subsequent medical school performance? A national, UK-based, cohort study
Source: BMJ Open. 2018 May 23;8(5):e020291. doi: 10.1136/bmjopen-2017-020291 (PMC5988088; doi:10.1136/bmjopen-2017-020291)
Supplement: Supplementary file 1 [file bmjopen-2017-020291supp001.pdf]

Supplementary document to:

What is the effect of secondary (high) schooling on subsequent medical school performance? A national, UK-based, cohort study

Lazaro M. Mwandigha , Paul A. Tiffin, Lewis W. Paton, Adetayo S. Kasim, Jan R. Böhnke

Contents

1 Descriptive and Inferential statistics 2

2 Estimation of Prior Education Attainment (PEA) 7

3 Mediation analyses 9
- 3.1 Single-level simple mediation analyses 9
- 3.2 Multi-level simple mediation analyses 10
- 3.3 Choosing between single-level and multi-level simple mediation analyses 16

4 Multi-level linear model 20

5 Missing data 22

6 Sensitivity analysis for missing data 24
- 6.1 Single-level simple mediation analyses 25
- 6.2 Multi-level linear model 28

1 Descriptive and Inferential statistics

Figure 1 shows the distribution of the entrants total UKCAT scores across the 18 medical schools in the different universities. The distribution of the total UKCAT scores seem to differ widely. This may be partly explained by the fact that different medical schools use the UKCAT differently in the selection process. Some use the UKCAT as a "border-line method" (to discriminate amongst a small number of applicants lying at a decision borderline, who are otherwise indistinguishable on the medical school's other selection criteria), or "factor method" (an applicant's UKCAT score or a proxy for that score is added to the score the applicant obtains in the medical school's usual method of selection, to provide a total score), or "threshold method" (minimum or threshold UKCAT score is adopted to create a hurdle that an applicant must cross to reach the next stage in the selection process) or "rescue" (to compensate for an applicants who would otherwise be rejected on account of their score on other selection criteria) [4].

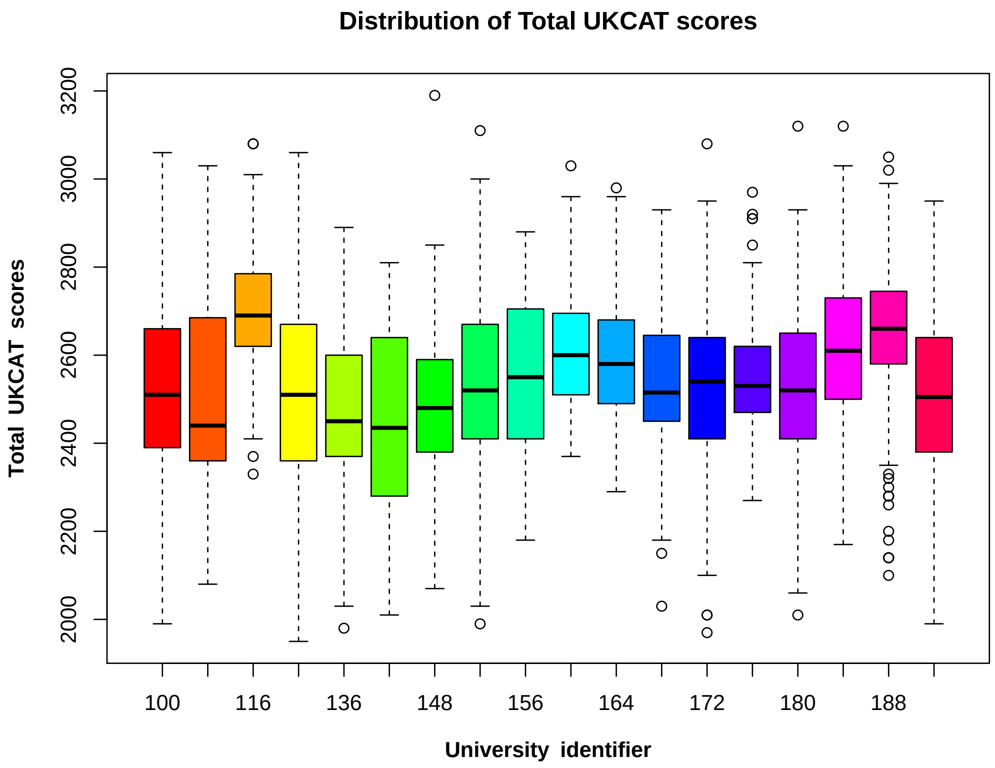

Figure 1: *Box plot of the distribution of the total UKCAT score for the different medical schools in UKCAT-consortium universities*

Table 1 shows the same information depicted in Figure 1 by means of summary statistics. To determine whether the distributional differences at university level may also be a factor of the quality of secondary school attended by a medical school entrant in a university, the total UKCAT matriculation scores were categorised into three (ranked) groups based on the standardised average performance of secondary schools attended by the entrants.

	University identifier	Mean	SD	Minimum	Maximum
1	100	2,513.06	200.45	1,990	3,060
2	108	2,498.00	246.81	2,080	3,030
3	116	2,690.69	150.22	2,330	3,080
4	120	2,506.16	213.26	1,950	3,060
5	136	2,463.36	186.42	1,980	2,890
6	144	2,448.33	197.10	2,010	2,810
7	148	2,485.37	170.25	2,070	3,190
8	152	2,521.22	219.61	1,990	3,110
9	156	2,550.00	183.80	2,180	2,880
10	160	2,610.18	136.20	2,370	3,030
11	164	2,590.61	149.13	2,290	2,980
12	168	2,524.74	169.51	2,030	2,930
13	172	2,519.26	195.10	1,970	3,080
14	176	2,552.64	120.39	2,270	2,970
15	180	2,522.39	178.94	2,010	3,120
16	184	2,615.40	175.59	2,170	3,120
17	188	2,643.50	177.32	2,100	3,050
18	192	2,502.67	190.36	1,990	2,950

Table 1: *Summary statistics of the total UKCAT score for the different medical schools in the 18 UKCAT-consortium universities*

Secondary schools were categorised into tertiles based on their on their standardised performance. Those with standardised performance of between [-2.5167, 0.3834), [0.3834, 1.2708) and [1.2708, 2.5875] were categorised into ranked groups 1, 2 and 3 respectively. The "[" and "]" indicate the limit is included in the group. The respective number of observations in the ranked groups were 622, 619 and 614 respectively. As may be observed from these values, the (ranked) groups had somewhat an equal number of ob-

servations. There were 252 observations that were ungrouped due to missing values in average school level performance. For each of the groups, the corresponding standardised UKCAT matriculation scores were examined. The distribution of the total UKCAT matriculation scores for the three ranked groups are shown in Figure 2. The lowest UKCAT performance was observed for entrants who attended secondary schools in group 1. The distribution of the total UKCAT matriculation scores in this group seemed differentiated from the other two groups. The secondary schools represented in group 2 and 3 did not seem differentiated from each other in terms of total UKCAT matriculation scores of medical school entrants who attended them.

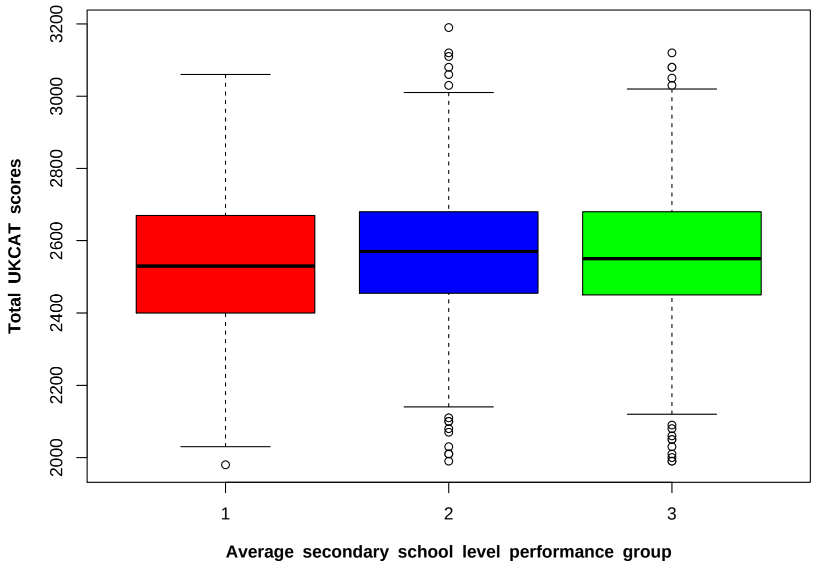

Figure 2: *Box plot of the distribution of the standardised total UKCAT score by category of rank of standardised average secondary school level performance*

To statistically confirm the trend observed in Figure 2, a one-way anova was conducted. The factor of interest was the group which had an ordered level of 1, 2 and 3 based on average secondary school level performance as already described. Following a statistically significant mean difference (p-value < 0.001) in the total UKCAT matriculation scores between the groups, the *Tukey's multiple group* comparison was conducted. This was done to determine the full extent and direction of the differences between the groups. Table 2 shows the results of this comparison which confirm the observed trend in Figure 2.

Compared to group 1, the total UKCAT matriculation scores were higher for entrants who attended secondary schools in groups 2 and 3. There was no evidence that total UKCAT matriculation scores differed for entrants who attended secondary schools in groups 2 and 3.

	Tukey's anova multiple group comparison			
Rank group	Difference	Lower 95% limit	Upper 95% limit	Adjusted p-value
2-1	43.1593	18.4357	67.8829	0.0001
3-1	34.4978	9.7238	59.2718	0.0032
3-2	-8.6615	-33.4653	16.1423	0.6912

Table 2: Total UKCAT score differences between groups based on the average secondary school level performance

Figure 3 shows the distribution of the undergraduate year one *knowledge*-based outcome scores prior to and after their standardisation within each of the university. Note that only 13 out of the 18 UKCAT consortium (medical schools) universities reported outcomes for knowledge-based exams in the first year. This is clearly seen from the number of box plots in the top and bottom panel with no corresponding standardisation in the bottom panel. The university identified with code 136 reported a single score of 63.61 hence the single line depicted instead of a box plot in the top panel. Universities identified with codes 148, 152, 164, 184 did not report any score for the undergraduate *knowledge*-based exam outcomes at the end of the first year of medical school training. Further, it may be said that standardisation does not affect the distribution of the *knowledge*-based outcome scores as the relative size of the box plots between universities remain the same before and after standardisation. Note that standardisation merely shifts the scale of comparison by allowing the different plots to have mean (value of approximately zero) that is similar across the different universities. Therefore, the underlying differences in the reported outcomes were modelled by a multi-level model (regardless of whether or not standardisation was done or not).

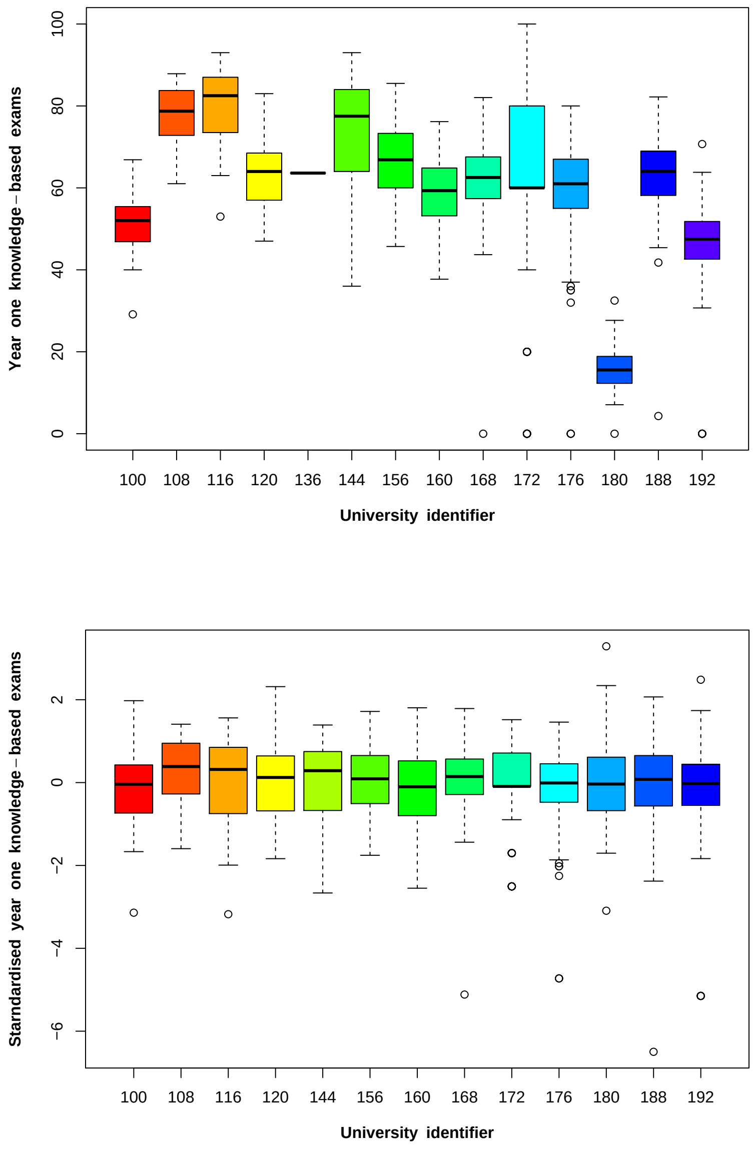

Figure 3: *Box plot of the distribution of end of year one knowledge-based outcomes for the different medical schools in UKCAT-consortium universities. The top panel and bottom panel shows the unstandardised and standardised undergraduate knowledge-based outcome scores respectively*

Table 3 shows the predictive validity of the total UKCAT score and PEA as estimated by bivariate Pearson correlation coefficients. Generally, the predictive validity of PEA was higher than that of the total UKCAT score. It was also observed that the predictive validity for the *knowledge*-based outcomes were higher than that for *skills*-based outcomes. The predictive validity of both the total UKCAT score and PEA was highest in the first two years of medical school training.

Predictor	Undergraduate knowledge-based outcome				
	Year one	Year two	Year three	Year four	Year five
PEA	0.25 (< 0.001)	0.23 (< 0.001)	0.23 (< 0.001)	0.18 (< 0.001)	0.22 (< 0.001)
total UKCAT score	0.11 (< 0.001)	0.11 (< 0.001)	0.15 (< 0.001)	0.11 (< 0.001)	0.16 (< 0.001)

Predictor	Undergraduate skills-based outcome				
	Year one	Year two	Year three	Year four	Year five
PEA	0.16 (< 0.001)	0.16 (< 0.001)	0.10 (< 0.001)	0.10 (0.0006)	0.13 (0.0012)
total UKCAT score	0.07 (0.0165)	0.06 (0.0238)	0.06 (0.0319)	0.07 (0.0164)	0.11 (0.0068)

Table 3: *Predictive validities of PEA and total UKCAT score for undergraduate medical school performance. The computed predictive validities are estimated by bivariate Pearson correlation coefficients from pairwise deleted data. The associated p-values for the reported validities are shown in brackets*

2 Estimation of Prior Education Attainment (PEA)

In order to obtain a single metric of scholastic (or academic) ability from the reported GCSEs and A Level exam scores, a novel approach described by *McManus et. al* [1] which involved conceptualising *educational achievement* as a latent variable was used. Thus PEA was estimated as a latent trait via an ordinal factor analysis using the most commonly taken A-level (both A1 and A2), and the grades obtained (e.g. A, B, C etc) used as (ordered categorical) indicators (see Table 4). The non-hierarchical version of McDonald's Omega was computed from the polychoric correlation matrix, since the factor analysis was of first order [2, 3]. The non-hierarchical McDonald's Omega was found to be 0.91. *Full Information Maximum Likelihood (FIML)* which maximizes use of the available data was used for the analysis to deal with missingness in the data (e.g. for the subjects not taken by a particular candidate). Subsequently, factor scores were then estimated for all applicants in the data, the results of the factor analysis from *Mplus* are displayed on Table 5. It was observed that generally, higher loadings were associated with Chemistry, Physics and Biology in GCSEs and A-Level (both A1 and A2) exams.

Exam	Subjects considered	Grade coding for factor analysis
GCSE	*Biology, Chemistry, Physics*, *Maths, French, History*, *Religious studies, Science, English*, *English literature* and *Geography*	C, D, E, F and G=1, B=2, A=3 and A^{*}=4
A Level (includes A1 and A2-level)	*Maths, Chemistry, Biology* and *Physics*	E and D=1 , C=2, B=3 and A=4

Table 4: *Coding of GCE A-Level and GCSE subjects for factor analysis*

Exam	Subject	Loading	Std Error	Estimate / Std. Error	Two sided p-value
GCSEs	Biology	0.805	0.009	93.988	0.000
	Chemistry	0.815	0.009	95.105	0.000
	English Literature	0.503	0.010	51.869	0.000
	English	0.572	0.009	62.060	0.000
	French	0.611	0.010	60.850	0.000
	Geography	0.696	0.011	60.710	0.000
	History	0.628	0.012	51.736	0.000
	Maths	0.693	0.008	90.998	0.000
	Physics	0.828	0.008	102.854	0.000
	Religious Education	0.510	0.012	43.155	0.000
	Science	0.749	0.049	15.233	0.000
A1-Level	Biology	0.861	0.005	171.013	0.000
	Chemistry	0.822	0.006	149.020	0.000
	Maths	0.798	0.009	93.642	0.000
	Physics	0.847	0.012	71.542	0.000
A2-Level	Biology	0.818	0.006	126.211	0.000
	Chemistry	0.798	0.007	121.959	0.000
	Maths	0.738	0.010	72.379	0.000
	Physics	0.836	0.010	86.867	0.000

Table 5: *Results from the factor analysis for the derivation of factor scores for PEA*

3 Mediation analyses

3.1 Single-level simple mediation analyses

It was aimed to *determine the extent to which an entrants PEA would mediate the predictive power of the UKCAT for two separate domains (knowledge and skills) over the period of undergraduate training*. To accomplish this a mediation model was considered. This is because, the overall *total predictive power of the UKCAT* for *knowledge* and *skills*-based undergraduate medical school exams would be partitioned into *direct* and *indirect* predictive power. This would then enable the accurate assessment of the relative, and unique, contribution UKCAT scores makes within the selection process. To demonstrate how this is done, consider Figure 4, which shows a *simple mediation model*. The term "simple" means that there is a one predictor, one mediator and one outcome variable under consideration.

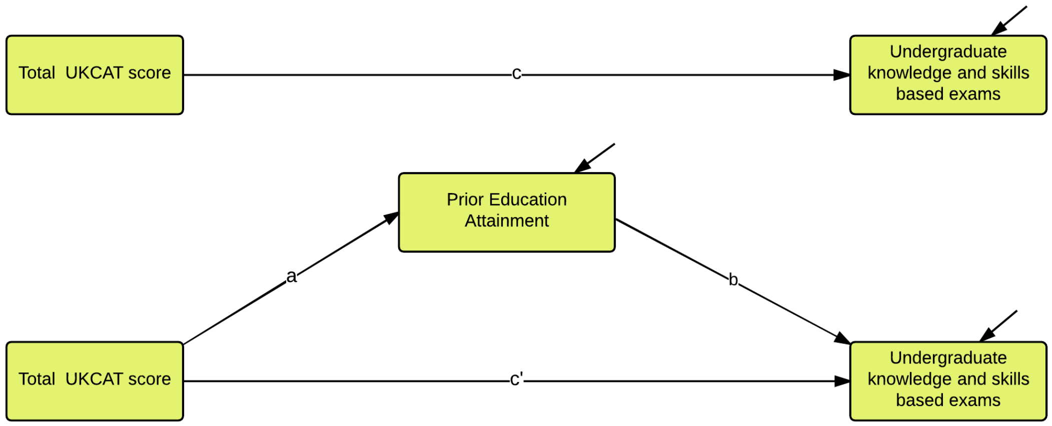

Figure 4: *Conceptual diagram of simple mediation model*

The effect denoted by *c* is the *total effect*, this may be easily obtained as a regression coefficient from a *Ordinary Least Squares (OLS)* regression model. The paths *b* and *c'* are *direct effects* for PEA and UKCAT respectively both of which may be obtained from a OLS regression model. For the purpose of the study, the paths of main interest were the *indirect effect*, product of the paths *a*b*, shown in equation 3.1. This *indirect effect* represents the non-unique contribution of the predictive power of the UKCAT. Further, a

proportion of this non-unique contribution, which is the portion of the predictive power of the UKCAT that is explained by PEA, may be expressed as $\frac{a*b}{c}$ (see Figure 3 in text of main paper) where c is the *total effect* which has been shown to be equal to sum of the *indirect* and *direct effects*

$$c = a * b + c' \tag{3.1}$$

The significance of the *indirect effect* may be obtained by testing the hypothesis $H_0 : a * b = 0$ versus $H_0 : a * b \neq 0$, traditionally, this was done by assuming a normal distribution for the *indirect effect* of $a * b$ thus necessitating the use of wald, score or likelihood ratio test with their corresponding p-value. This however, may lead to incorrect conclusions, when the *indirect effect* is not normally distributed as is often the case [5]. For this reason, most statistical software packages, such as Mplus implement a hypothesis test using a bootstrap approach which yields an empirical distribution for $a * b$. Similarly, it is possible for one to program this in any statistical software (e.g. R) by implementing a bootstrap or Monte Carlo simulation. The idea being the derivation of $(1 - \alpha)100$ bootstrap or Monte Carlo percentile confidence intervals for the purpose of determining signficance. For SAS and SPSS users, macros have been developed for estimating the significance of the *indirect effect*, they include the *INDIRECT* and *PROCESS* macros which are based on the bootstrap while *MCMED* macro is based on Monte Carlo Simulation [6, 7].

3.2 Multi-level simple mediation analyses

The structure of the data used for the study was hierarchical (clustered) because the outcomes (*knowledge* and *skills*) considered in each year of undergraduate training were nested within the 18 universities. This means that fitting a simple mediation analysis which essentially ignored the hierarchical structure of the data would potentially result in *total, direct* and *indirect effects* with induced attenuations. This may then lead to biased conclusions. For this reason, a multi-level mediation model was considered. In a nutshell,

this model constitutes fitting a simple mediation for each cluster (university) separately and subsequently pooling the effects of interest together in some defined way to form *population average total, population average direct* and *population average indirect effects*. A conceptual representation of this model may be viewed on Figure 5.

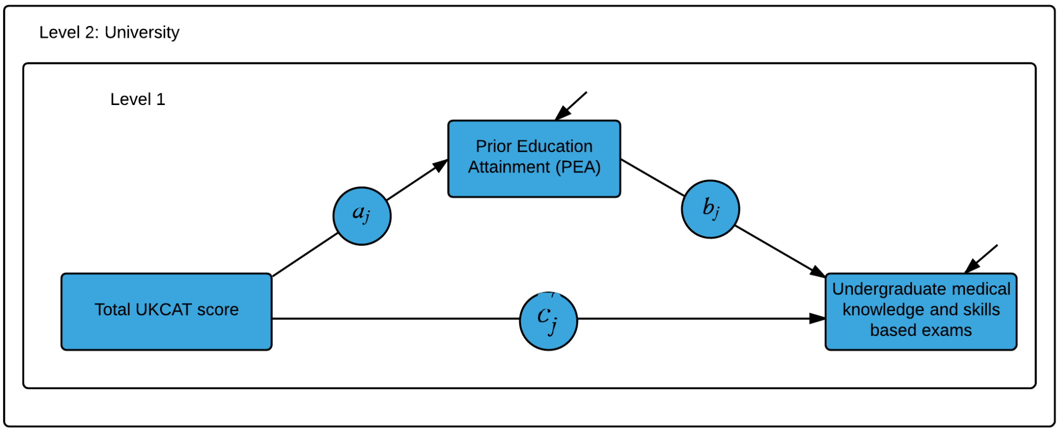

Figure 5: *Conceptual diagram of multi-level mediation model*

Note that, unlike in the case of the simple (single-level) mediation in Figure 4, the effects are now level-1 variables nested within university which is a level-2 variable. Further, all the effects are estimated as random rather than fixed effects thus allowing them to vary between the level-2 variables. This model is called the 1→ 1→ 1 mediation model since the predictor, UKCAT, the mediator, PEA, and the outcomes, *knowledge* and *skills*-based exams, all reside on level-1. In the conceptual representation of the model, the subscript j denotes that effects of interest vary between universities. These effects in the Figure are encircled to denote in *Structural Equation Modelling (SEM)* methodology that these effects are random [8]. The implementation of the 1→ 1→ 1 model is demonstrated for the *knowledge*-based exam scores (denoted by K) for brevity. The UKCAT and PEA scores are denoted by UKCAT and PEA respectively.

$$PEA_{ij} = d_{PEA_j} + a_j * UKCAT_{ij} + \varepsilon_{PEA_{ij}} \tag{3.2}$$

$$K_{ij} = d_{K_j} + b_j * PEA_{ij} + c'_j * UKCAT_{ij} + \varepsilon_{K_{ij}} \tag{3.3}$$

$$
\begin{aligned}
d_{PEA_j} &= d_{PEA} + \mu_{d_{PEA_j}} \\
d_{K_j} &= d_K + \mu_{K_j} \\
a_j &= a + \mu_{a_j} \\
b_j &= b + \mu_{b_j} \\
c'_j &= c' + \mu_{c'_j}
\end{aligned} \tag{3.4}
$$

The subscript i denotes a student and subscript j a particular university. Further, $\varepsilon_{PEA_{ij}}$ and $\varepsilon_{K_{ij}}$ are level-1 residuals for the mediator PEA and Knowledge based outcome of interest respectively. Finally, d_{PEA_j}, d_{K_j}, a_j, b_j and c'_j are the random intercepts and slopes of the models. The assumptions of the $1 \rightarrow 1 \rightarrow 1$ hierarchical mediation model are as follows

1. The predictor, $UKCAT_{ij}$ is uncorrelated with all the random effects (d_{PEA_j}, d_{K_j},a_j, b_j and c'_j) and the residuals ($\varepsilon_{PEA_{ij}}$ and $\varepsilon_{K_{ij}}$) in the model.
2. The residuals from the models, $\varepsilon_{PEA_{ij}}$ and $\varepsilon_{K_{ij}}$, are each normally distributed with an expected value of zero and are uncorrelated with one another.
3. The level-1 residuals, $\varepsilon_{PEA_{ij}}$ and $\varepsilon_{K_{ij}}$ are uncorrelated with random effects d_{PEA_j}, d_{K_j}, a_j, b_j and c'_j in the model.
4. The random effects are normally distributed with means equal to the average effects in the population. This may be expressed as,

   $$E(a_j) = \bar{a}_j = a$$

   $$E(b_j) = \bar{b}_j = b$$

   and

   $$E(c'_j) = \bar{c}'_j = c'$$

   for the slopes of interest. Further, the random effects covary with one another.
5. The distributions of PEA_{ij} is normal conditional on $UKCAT_{ij}$ and K_{ij} normal conditional on PEA_{ij} and $UKCAT_{ij}$.

These assumptions lead to the following matrix formulation of the model. Note that, it is possible to estimate the *average of effects* (which may be referred to as *"population level effects"*, quantify the effects across all universities and their corresponding variabilities)

$$\begin{bmatrix} d_{PEA_j} \\ d_{K_j} \\ a_j \\ b_j \\ c_j^{'} \end{bmatrix} \sim N \left(\begin{bmatrix} d_{PEA} \\ d_K \\ a \\ b \\ c^{'} \end{bmatrix}, \begin{bmatrix} \sigma^2_{d_{PEA_j}} & & & & \\ \sigma_{d_{PEA_j K_j}} & \sigma^2_{K_j} & & & \\ \sigma_{d_{PEA_j a_j}} & \sigma_{K_j a_j} & \sigma^2_{a_j} & & \\ \sigma_{d_{PEA_j b_j}} & \sigma_{K_j b_j} & \sigma_{a_j b_j} & \sigma^2_{b_j} & \\ \sigma_{d_{PEA_j c_j^{'}}} & \sigma_{K_j c_j^{'}} & \sigma_{a_j c_j^{'}} & \sigma_{b_j c_j^{'}} & \sigma_{c_j^{'}} \end{bmatrix} \right)$$

The *average mediation (indirect) effect* and *average total* effects may then be estimated by making use of equations 3.5 and 3.6 respectively.

$$E(a_j * b_j) = a * b + \sigma_{a_j b_j} \tag{3.5}$$

$$E(a_j * b_j + c^{'}) = a * b + \sigma_{a_j b_j} + c^{'} \tag{3.6}$$

The multi-level simple mediation model was fitted in Mplus and the estimates of *average total, average indirect* and *average direct effects* estimated from equations 3.5 and 3.6. The significance of the *average total* and *average direct effects* were obtained from the results in Mplus. To determine the significance of *average indirect effect*, a Monte Carlo 95% Percentile CI was programmed in R software by sampling 10,000 observations from the distribution in equation 3.7.

$$N \left(\begin{bmatrix} a \\ b \\ \sigma_{a_j b_j} \end{bmatrix}, \begin{bmatrix} \sigma^2_a & \sigma_{ab} & \sigma_{a,\sigma_{a_j b_j}} \\ & \sigma^2_b & \sigma_{b,\sigma_{a_j b_j}} \\ & & \sigma^2_{\sigma_{a_j b_j}} \end{bmatrix} \right) \tag{3.7}$$

The individual elements of the distribution in equation 3.7 were obtained from the results of the multi-level mediation model in Mplus using the *TECH 3* output command. Each of the 10,000 observations sampled for a, b and $\sigma_{a_j b_j}$ were plugged into equations 3.5 and 3.6 to obtain 10,000 *average indirect effect* values. Subsequently, the Monte Carlo 95%

Percentile CI was calculated by taking the 2.5^{th} and 97.5^{th} percentile of the empirical distribution of the 10,000 estimates for *indirect effect*. Figure 6 shows the plotted results from the models. It was observed that there were statistical significant *average indirect effects* in the first four years of undergraduate training of medical school for both *knowledge* and *skills*-based exams outcomes. The *indirect effects* represent the contribution of PEA towards the predictive power of the UKCAT. It was also observed that the range of the CIs widened in the third year onwards which is indicative of the missingness observed in the later years of the study (see Figure 1 and Table 1 in main text of the paper) which led to little information available for analysis in each of the university clusters in the data.

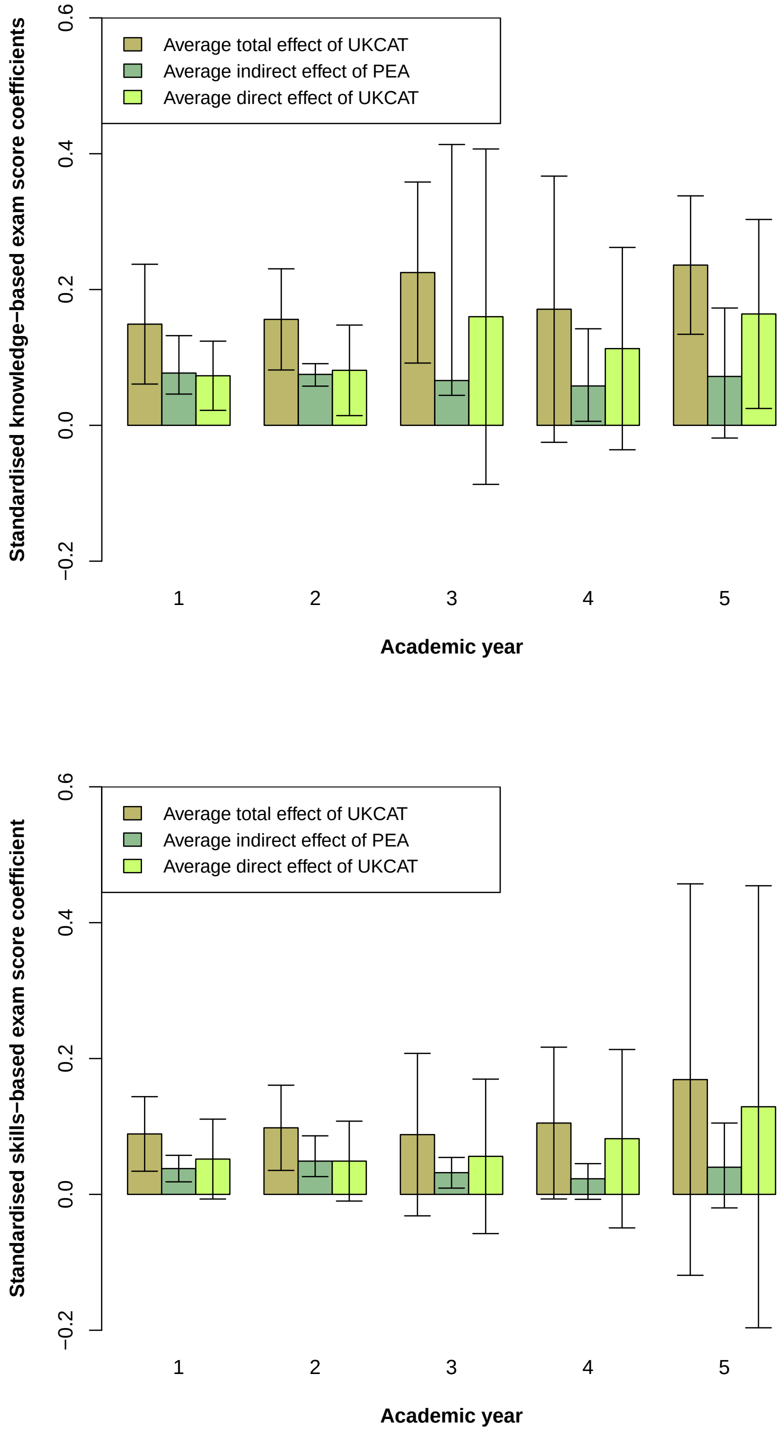

Figure 6: *Knowledge and skills-based multi-level mediation results for the average total, average direct and average indirect effects with respective 95% CI for average total and average direct effects computed from point estimates and standard errors obtained in Mplus and 95% Monte Carlo CI computed in R through simulation*

3.3 Choosing between single-level and multi-level simple mediation analyses

The multi-level mediation model fitted in section 3.2 is prone to convergence difficulties and is highly susceptible to missing data related problems. For instances where there are high attrition rates in later years of a longitudinal cohort study, it is highly likely that some or most of the clusters may have little or no data to contribute meaningfully to the analysis and this may further risk a lack of convergence. Therefore, for a given estimation problem, a single-level mediation model is preferred if there is evidence that there are no statistically significant clustering effects in the data.

To determine whether there were statistically significant clustering effects in the data equations 3.5 and 3.6 were considered. Note that from equation 3.5, when $\sigma_{a_j b_j} = 0$, the resulting *average indirect effect* is equal to what would be estimated in a single-level simple mediation analysis in section 3.1. Therefore in seeking to determine whether a single or multi-level mediation analysis should be fitted to the data, it will be sufficient to test the hypothesis, $H_0 : \sigma_{a_j b_j} = 0$ versus $H_1 : \sigma_{a_j b_j} \neq 0$. Evidence in favour of the null hypothesis would also be evidence in favour of a simple single-level mediation analysis. The results of the hypothesis test were available as part of the multi-level results in Mplus and are displayed on Table 6. It was observed that all of the p-values were > 0.05 implying that there were statistically non-significant clustering effects in the data. Further, *Intra Cluster Correlations (ICCs)* for the models computed by utilising the main diagonal of the covariance matrix from equation 3.7 and the residual variances from the model are displayed on Table 7. The observed ICCs (7th and 13th column of the Table) indicate that the proportion of variability explained by the multi-level mediation models is negligible. Therefore a simple single-level mediation model is appropriate for the data.

Following the results on Tables 6 and 7, a simple single-level mediation model was fitted using two models, for the case of *knowledge*-based exams outcomes, shown in equation

(3.9) and (3.8) respectively using the same notation as in section 3.2.

$$PEA_i = I_{PEA} + a * UKCAT_i + \varepsilon_{PEA} \tag{3.8}$$

$$K_i = I_K + c^{'} * UKCAT_i + b * PEA_i + \varepsilon_K \tag{3.9}$$

Academic year	Knowledge- based exams			Skills-based exams		
	$\sigma_{a_j b_j}$	Std. Error	P-value	$\sigma_{a_j b_j}$	Std. Error	P-value
1	-0.007	0.016	0.663	-0.006	0.007	0.414
2	-0.004	0.003	0.284	-0.005	0.012	0.673
3	-0.002	0.066	0.972	0.000	0.003	0.888
4	-0.001	0.006	0.872	-0.004	0.013	0.778
5	0.000	0.031	0.992	-0.001	-0.009	0.951

Table 6: *Results of the hypothesis testing for the statistical significance of* $\sigma_{a_j b_j}$ *from Mplus*

Academic year	Knowledge-based exams					
	σ_a^2	σ_b^2	σ_{res}^2	σ_{PEA}^2	$\sigma_{\sigma_{a_j b_j}}^2$	$\frac{\sigma_{\sigma_{a_j b_j}}^2}{(\sigma_a^2+\sigma_b^2+\sigma_{res}^2+\sigma_{PEA}^2+\sigma_{\sigma_{a_j b_j}}^2)}$
1	0.006	0.003	0.848	1.876	0.000	0.000
2	0.002	0.000	0.894	1.875	0.000	0.000
3	0.093	0.034	0.841	1.875	0.004	0.002
4	0.005	0.006	0.890	1.875	0.000	0.000
5	0.004	0.002	0.902	1.876	0.001	0.000

Academic year	Skills-based exams					
	σ_a^2	σ_b^2	σ_{res}^2	σ_{PEA}^2	$\sigma_{\sigma_{a_j b_j}}^2$	$\frac{\sigma_{\sigma_{a_j b_j}}^2}{(\sigma_a^2+\sigma_b^2+\sigma_{res}^2+\sigma_{PEA}^2+\sigma_{\sigma_{a_j b_j}}^2)}$
1	0.003	0.001	0.888	1.877	0.000	0.000
2	0.011	0.001	0.968	1.876	0.000	0.000
3	0.003	0.001	0.947	1.876	0.000	0.000
4	0.012	0.000	0.893	1.876	0.000	0.000
5	0.003	0.006	0.985	1.877	0.000	0.000

Table 7: *Intra Cluster Correlations (ICC) for knowledge and skills-based exam outcomes for the five years of undergraduate medical school training*

The mediator of interest is *PEA* while *UKCAT* and *K* are predictor and outcome of interest respectively. The *I* denotes the intercept while *a,b* and *c* are the regression coefficients to be estimated. This model was fitted both in Mplus and in SAS. The results of the models from the two software packages were expectedly similar. The statistical significance was tested using the bootstrap approach implemented in Mplus and Monte Carlo simulation in SAS using the *MCMED macro for SAS* [6]. In both Mplus and SAS, the 95% confidence intervals were obtained by taking the 2.5^{th} and 97.5^{th} percentiles of the empirical distribution for $a * b$ from 10,000 sampled observations. The single-level simple mediation model results (from SAS, similar to results from Mplus) are shown in Tables 8 and 9 for *knowledge* and *skills*-based exams outcomes respectively. For both *knowledge* and *skills*-based outcomes, in all undergraduate years, there were statistically significant *indirect effects* of UKCAT through PEA. This means that the predictive power of the UKCAT for undergraduate medical school performance can be partially explained by PEA.

	Knowledge-based exams					
	Direct effect		Indirect effect		Total effect	
Academic year	Estimate	95% CI	Estimate	95% Monte Carlo CI	Estimate	95% CI
1	0.071	(0.002, 0.139)	0.081	(0.059, 0.106)	0.151	(0.083, 0.220)
2	0.073	(0.002, 0.144)	0.074	(0.053, 0.010)	0.147	(0.077 0.217)
3	0.127	(0.058, 0.195)	0.069	(0.049, 0.094)	0.196	(0.129, 0.263)
4	0.086	(0.014, 0.159)	0.062	(0.040, 0.085)	0.148	(0.078, 0.218)
5	0.162	(0.058, 0.266)	0.052	(0.027, 0.087)	0.213	(0.109, 0.318)

Table 8: *Results of the single-level simple mediation model for the undergraduate knowledge-based exam outcome*

Academic year	Skills-based exams					
	Direct effect		Indirect effect		Total effect	
	Estimate	95% CI	Estimate	95% Monte Carlo CI	Estimate	95% CI
1	0.056	(-0.028, 0.140)	0.045	(0.026, 0.070)	0.101	(0.019, 0.184)
2	0.032	(-0.049, 0.113)	0.059	(0.038, 0.085)	0.091	(0.012, 0.170)
3	0.048	(-0.026, 0.122)	0.031	(0.012, 0.052)	0.078	(0.007, 0.150)
4	0.062	(-0.017, 0.141)	0.032	(0.012, 0.055)	0.094	(0.017, 0.170)
5	0.121	(0.010, 0.232)	0.030	(0.009, 0.063)	0.151	(0.042, 0.261)

Table 9: *Results of the single-level simple mediation model for the undergraduate skills-based exam outcome*

The results from Tables 8 and 9 were used to compute the proportions of the total UKCAT scores explained by the PEA shown in Figure 3 of main manuscript. To determine whether the proportion of total UKCAT scores explained by the PEA in each year of medical school training varied by outcome, a statistical test for the significance of the difference between the proportions was conducted as shown in equation 3.10 in each year of medical school training. The subscripts k and s denote *knowledge* and *skills*-based exams outcomes respectively. The term p denotes the proportion of the total UKCAT scores explained by the PEA. Table 10 shows the results of the statistical test conducted. It was observed that there were statistically significant differences in the proportions of the total UKCAT scores explained by the PEA between the *knowledge* and *skills*-based exams outcomes in all but the fifth year of medical training. It is was also observed that the fifth year of medical school training had very low sample sizes for the two outcomes under consideration. This contributed to a lack of sufficient power to detect differences in the proportions in that year.

$$Z = \frac{(p_k - p_s) - 0}{\sqrt(\frac{p_k(1-p_k)}{n_k} + \frac{p_s(1-p_s)}{n_s})} \tag{3.10}$$

Year	Undergraduate knowledge-based exams		Undergraduate skills-based exams		Results		
	Proportion ($p_k = \frac{a*b}{c}$)	Sample size	Proportion ($p_s = \frac{a*b}{c}$)	Sample size	$p_k - p_s$	Z	P-value
1	0.5331	1,453	0.4455	1,051	0.0875	4.3419	< 0.0001
2	0.5054	1,418	0.6443	1,233	-0.1388	-7.2948	< 0.0001
3	0.3541	1,348	0.3916	1,238	-0.0375	-1.9707	0.0488
4	0.4164	1,349	0.3369	1,072	0.0795	4.0325	0.0001
5	0.2423	626	0.2003	576	0.0420	1.7573	0.0789

Table 10: *Statistical test for the significance of the difference in the proportion of UKCAT explained by PEA between the undergraduate knowledge and skills-based exam outcomes. The p-values are estimated from a standard normal distribution*

4 Multi-level linear model

To address the second aim of the study, which was *to appraise the influence of the performance of the previous secondary school attended on an undergraduates achievement in medical school*, a multi-level linear model or *Linear Mixed Model (LMM)* was used due to its capability to handle clustering in instances where the outcomes are continuous and correlated. The term "mixed" in the Linear Mixed Model comes from the fact that the model estimates both fixed (mean structure) and random effects (random structure). The modelling framework of Linear Mixed Model may be expressed as follows:

$$Y_i = X_i\beta + Z_i b_i + \varepsilon_i \tag{4.1}$$

where

$$b_i \sim N(0, D)$$
$$\varepsilon_i \sim N(0, \Sigma_i)$$

with $b_1 \dots b_N$ and $\varepsilon_1 \dots \varepsilon_N$ being independent. Y_i is the n_i-dimensional outcome (*knowledge* or *skills*-based exams), X_i and Z_i are the design matrices for the fixed and random effects of known predictors respectively, β and b_i are fixed and university specific effects respectively, and ε_i is the vector containing the residual components [9]. X_i is a design

matrix containing the predictors; average school level performance of the school in which an entrant sat for their A-level exam, an entrant's reported A-level grade (AAA, AAB, ABB, BBB or BBC), interaction between average school level performance and reported A-level grades and the tier of an entrant's secondary school as categorised based on their performance (see Figure 2). Z_i is a design matrix containing a random intercept which modelled the correlation in the outcomes within a university by allowing the (predicted) outcomes to vary between universities.

As seen in Table 11, the effect of the secondary school group (ordered based on their performance as 1, 2 or 3) was not statistically significant. This implies the A-level grades earned by an medical school entrant and the average level performance of secondary school attended are sufficient in explaining the undergraduate medical school outcomes. Further categorisation of secondary schools based on their performance adds no value in explaining undergraduate medical school outcomes. Therefore the proposed model fitted was in line with the predictors shown in Table 12.

	P-values for undergraduate knowledge-based outcome					P-values for undergraduate skills-based outcome				
Predictor	Year one	Year two	Year three	Year four	Year five	Year one	Year two	Year three	Year four	Year five
SSLP	0.0239	0.1939	0.2100	0.4393	0.2284	0.5688	0.0324	0.2272	0.9608	0.5137
A-Level grade	< 0.0001	< 0.0001	< 0.0001	< 0.0001	< 0.0001	0.0002	< 0.0001	0.0002	0.0007	0.0124
SSLP group	0.9551	0.7273	0.5640	0.9078	0.9864	0.9072	0.8393	0.9798	0.3546	0.2564

Table 11: *Results of the multi-level model showing the type 3 tests p-values (Pr. > F) for the predictors of undergraduate knowledge and skills-based outcomes for each of the year of medical school. SSLP is the average Secondary School Level Performance and SSLP group is the three tier categorisation of secondary schools based on their reported average performance*

	P-values for undergraduate knowledge-based outcome					P-values for undergraduate skills-based outcome				
Predictor	Year one	Year two	Year three	Year four	Year five	Year one	Year two	Year three	Year four	Year five
SSLP	< 0.0001	< 0.0001	< 0.0001	< 0.0001	0.0029	0.1403	< 0.0001	0.0014	0.0099	0.5596
A-Level grade	< 0.0001	< 0.0001	< 0.0001	< 0.0001	< 0.0001	0.0002	< 0.0001	0.0002	0.0008	0.0109

Table 12: *Results of the multi-level model showing the type 3 tests p-values (Pr. > F) for the predictors of undergraduate knowledge and skills-based outcomes for each of the year of medical schoo. Only SSLP and A-level grades were been retained in the model. No interaction between SSLP and A-level grades was detected*

5 Missing data

The missing data patterns for *knowledge*-based outcomes are shown in Table 13, it was observed that only 20.84% of the entrants had complete data for the *knowledge*-based exam outcome throughout the five years of medical school. Monotone pattern of missingness accounted for 47.36% of the missingness data patterns. The most frequently occurring monotone pattern of missingness had outcome data only for year one to year three. On the other hand, the most frequently occurring arbitrary (non-monotone) pattern of missingness had outcome data missing for year one, two and five.

Table 14 shows the pattern of missingness for *skills*-based exam outcome. About 17% of the entrants had complete data for the outcome over the course of the study duration while 41.29% of the data had monotone pattern of missingness. The most occurring monotone pattern of missingness had outcome data missing for year five. The most occurring arbitrary missingness pattern compromising of about 9.5% of the arbitrary missingess pattern was for year one, two and five.

Outcome						Count	%
Group	Year 1	Year 2	Year 3	Year 4	Year 5		
Complete							
1	O	O	O	O	O	439	20.84
Monotone missingness							
2	O	O	O	O	M	272	12.91
3	O	O	O	M	M	330	15.66
4	O	O	M	M	M	199	9.44
5	O	M	M	M	M	42	1.99
6	M	M	M	M	M	155	7.36
Arbitrary missingness							
7	O	M	O	M	M	7	0.33
8	O	O	M	O	O	50	2.37
9	O	O	M	O	M	114	5.41
10	M	O	O	O	M	7	0.33
11	O	M	O	M	M	3	0.14
12	M	O	M	M	M	4	0.19
13	M	M	O	O	M	274	13
14	M	M	O	M	M	16	0.76
15	M	M	M	O	O	135	6.41
16	M	M	M	O	M	58	2.75
17	M	M	M	M	O	2	0.09
Total						2,107	100

Table 13: *Missingness data patterns for the undergraduate knowledge-based scores for the 2,107 entrants who sat for the UKCAT in 2007. Each "O" and "M" represents each instance where data are present and absent respectively (i.e. the first row represents the proportion of cases with no missing data). Patterns are categorised as either monotone (i.e. where data relating to all subsequent years are missing after the initial missing data year) or arbitrary (i.e. non-monotone)*

Outcome						Count	%
Group	Year 1	Year 2	Year 3	Year 4	Year 5		
Complete							
1	O	O	O	O	O	360	17.09
Monotone missingness							
2	O	O	O	O	M	308	14.62
3	O	O	O	M	M	61	2.9
4	O	O	M	M	M	199	9.44
5	O	M	M	M	M	26	1.23
6	M	M	M	M	M	276	13.1
Arbitrary missingness							
7	O	O	M	O	M	91	4.32
8	O	M	O	M	M	6	0.28
9	M	O	O	O	M	37	1.76
10	O	M	M	O	O	37	1.76
11	M	O	M	M	M	140	6.64
12	M	M	O	O	O	79	3.75
13	M	M	O	O	M	197	9.35
14	M	M	O	M	M	153	7.26
15	M	M	M	M	O	137	6.5
Total						2,107	100

Table 14: *Missingness patterns for the undergraduate skills-based scores for the 2,107 entrants who sat for the UKCAT in 2007. Each "O" and "M" represents each instance where data are present and absent respectively (i.e. the first row represents the proportion of cases with no missing data). Patterns are categorised as either monotone (i.e. where data relating to all subsequent years are missing after the initial missing data year) or arbitrary (i.e. non-monotone)*

6 Sensitivity analysis for missing data

Sensitivity analysis was conducted to determine to what extent the missingness in the data influenced the results of the study. The data analysis for the study assumed *Missing At Random (MAR)* mechanism. The MAR assumption was invoked by making use of *ignorability* which entailed ignoring the missingness process. The purpose of the sensitivity analysis was to investigate whether this assumption was justifiable. This involved refitting the models with multiply imputed data and comparing the results from these models with those fitted previously under ingnorability. The premise being, if ignorability is valid under MAR, and *Multiple Imputation (MI)* which is also valid under MAR, then the results under both should be similar. When this is the case, the assumption of ignorability

and MAR would be justified.

6.1 Single-level simple mediation analyses

For the single-level simple mediation analysis, the models were fitted after imputation was conducted 30 times thus creating 30 datasets. These datasets were analysed and results later summarised through pooling of the estimates. The computation of associated standard errors of their estimates was also done. The MI was conducted in SAS using the *Monte Carlo Markov Chain (MCMC)* which imputes the missing values in the data in a way that retains the overall mean and covariate structure of the data assuming a joint multivariate normal distribution [10, 11]. The results of the previous non-imputed data displayed in Table 8 and 9 for both *knowledge* and *skills*-based exams are further displayed in graphical form in Figure 7. These were compared to the results from the multiply imputed data which are found on Figure 8. It was observed that in as far as the aim of the analysis was concerned, there were no discernible difference in the estimates and conclusions regarding the *indirect effects* of UKCAT through PEA for both the *knowledge* and *skills*-based outcomes from both the multiply imputed and non-imputed data. This implies that the assumptions of ignorability and MAR were plausible and that the missingness though severe in later years of the study, did not adversely effect the results and conclusions of the statistical analysis. This is expected as the missing data was created when participating medical schools failed to submit outcome data the UKCAT database in a that particular year. Thus, it may be concluded that the missing data was unlikely to threaten the validity of the inferences drawn from the results.

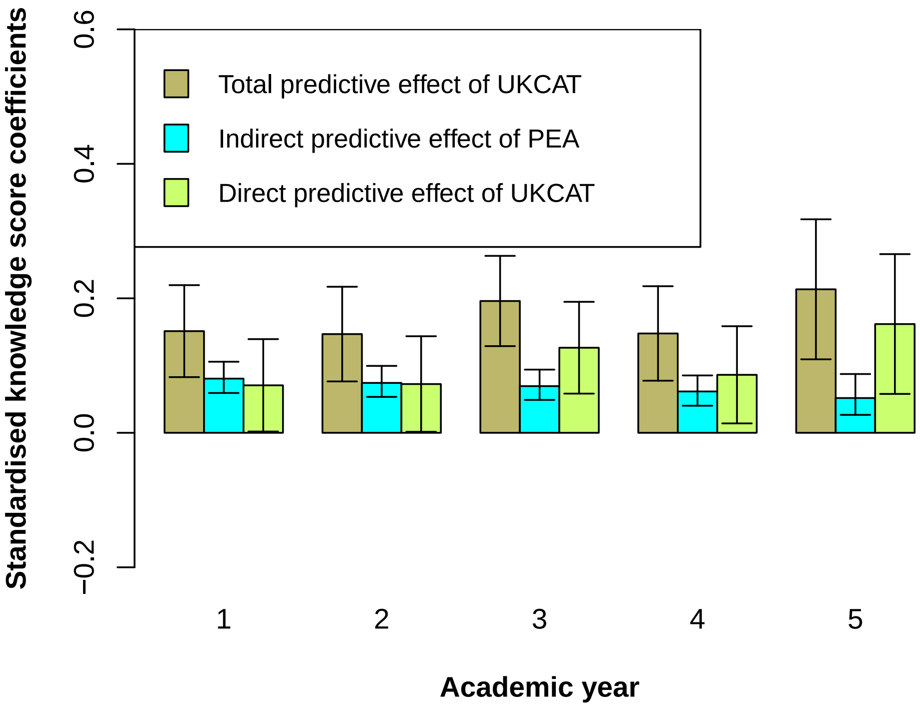

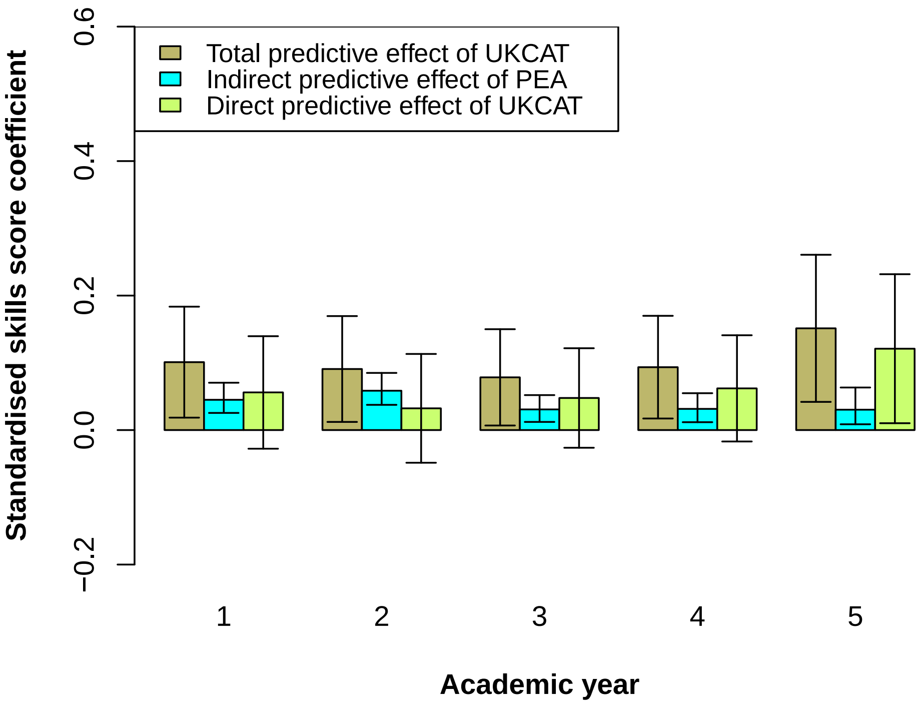

Figure 7: *Results of the single-level simple mediation analysis based on non-imputed data for undergraduate medical school knowledge and skills-based outcomes*

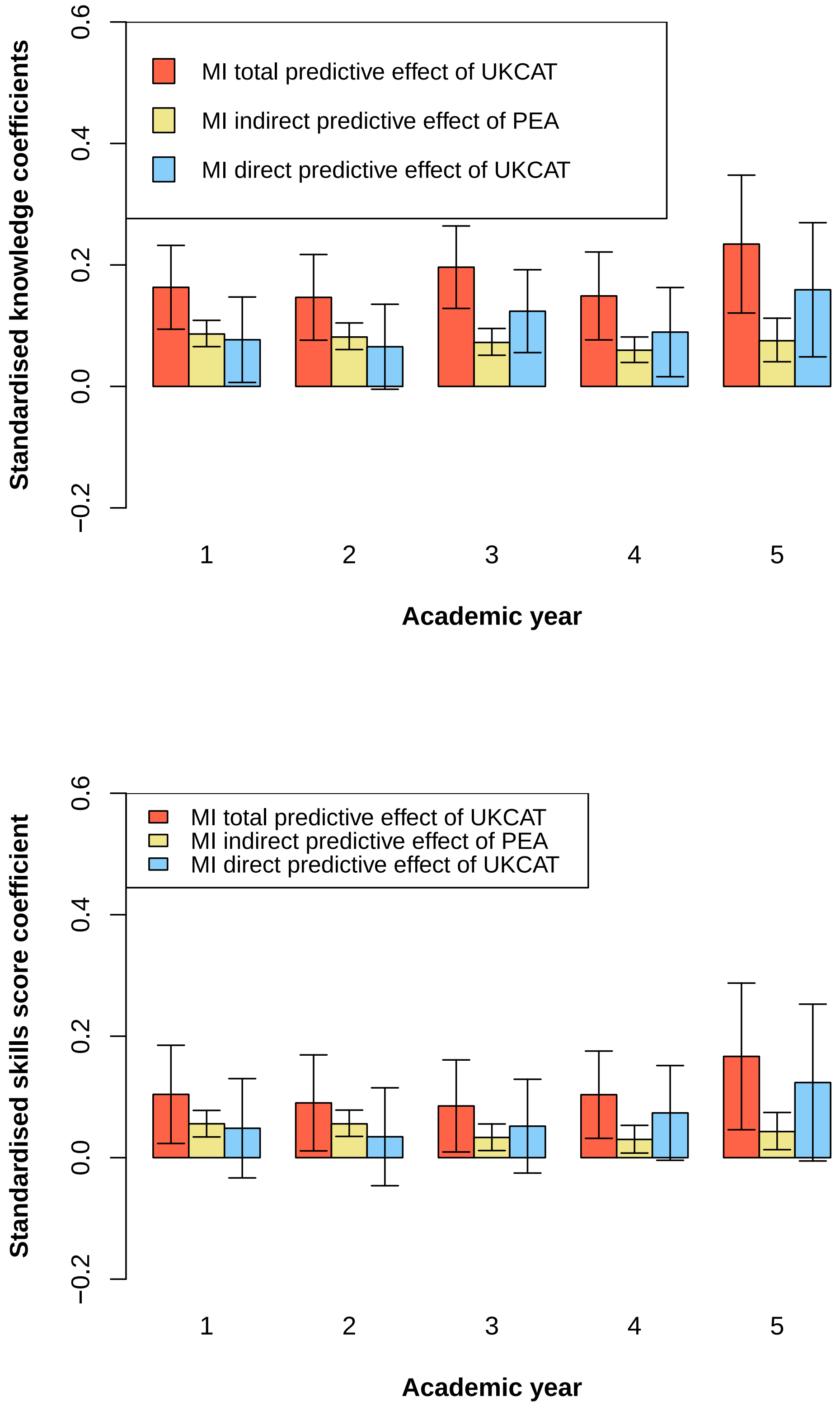

Figure 8: *Results of the single-level simple mediation analysis based on 30 MI data for undergraduate medical school knowledge and skills-based outcomes*

6.2 Multi-level linear model

Figure 9 and 10 show the plots of MI results for the model investigating the effect of average school level performance by reported grades on *knowledge* and *skills*-based exam outcomes for all five years of undergraduate medical school. All the variables of interest, that is, *knowledge* and *skills*-based undergraduate medical exam outcomes, average school level performance and PEA grades were affected by missingness. MI was conducted using *Multiple Imputations by Chain Equations (MICE)*, a MCMC based imputation technique that makes use of a collection of univariate conditional distributions of the variables with missing values given the other variables present in the data [10]. The number of imputations , M, was initially set at 5 and increased by multiples of 5 until a value of M that would yield unchanging results for the model described in section 4. The parameter estimates obtained were the same for $M >= 10$ indicating that any choice of $M >= 10$ was optimal. For comparison with results from the original data, M=15 was used.

The results from MI data were compared to those from the original data shown in Figures 4 and 5 in the main text of the paper for both *knowledge* and *skills*-based exams outcomes. The comparison revealed that the missingness did not an adverse effect on the analysis. Like in the original unimputed data, for both *knowledge* and *skills*-based exam outcomes, at each level of average school level performance students with higher grades tend to perform better compared to their counterparts with lower grades throughout undergraduate medical school. Overall, compared to students from schools with high average school level performance, students from schools with low average school level performance tend to have better scores in both *knowledge* and *skills*-based exam outcomes throughout undergraduate medical school. This suggests that the assumption of MAR invoked for the study was plausible.

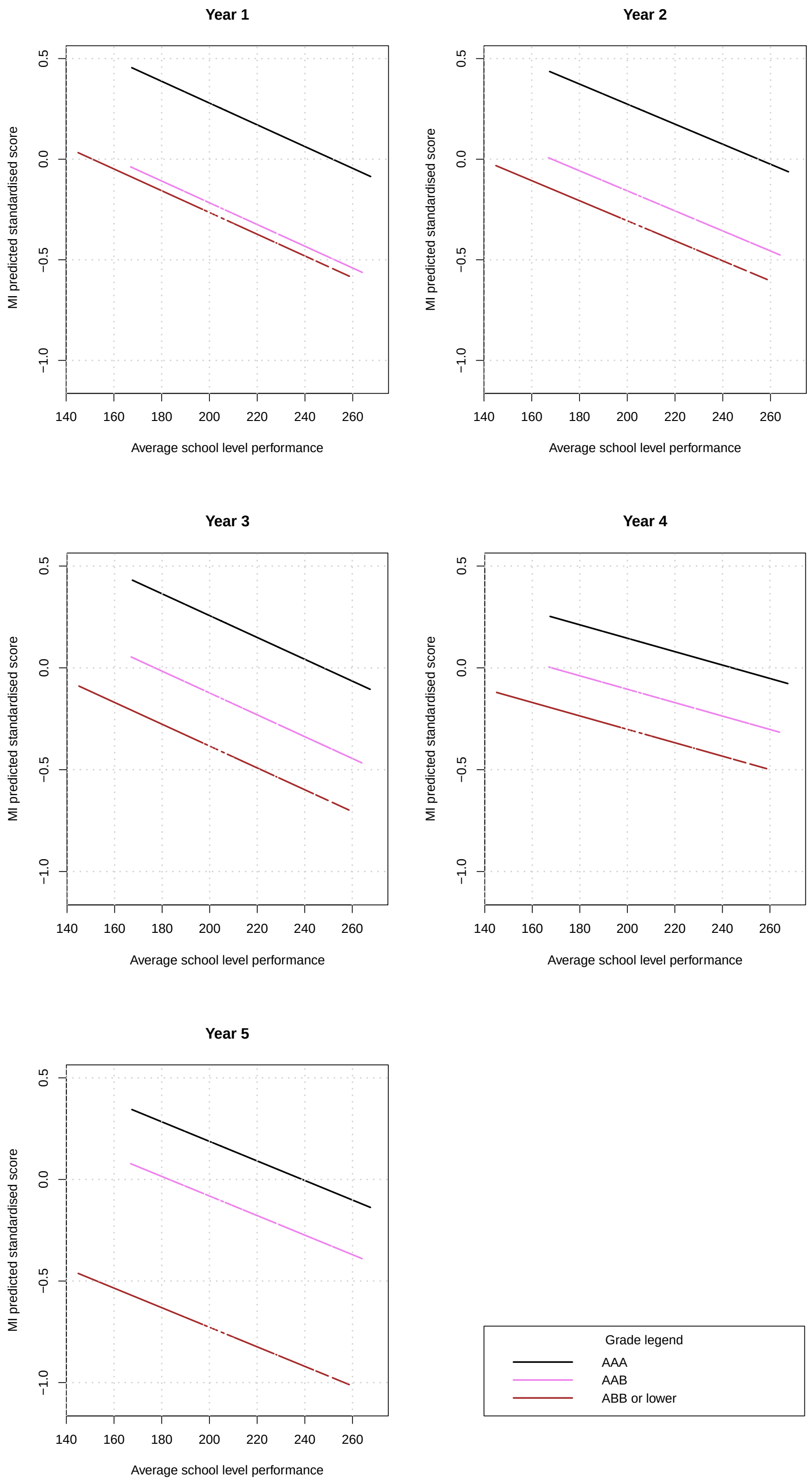

Figure 9: *Multiply imputed effect of average school level performance by reported grades on undergraduate medical school knowledge-based exams*

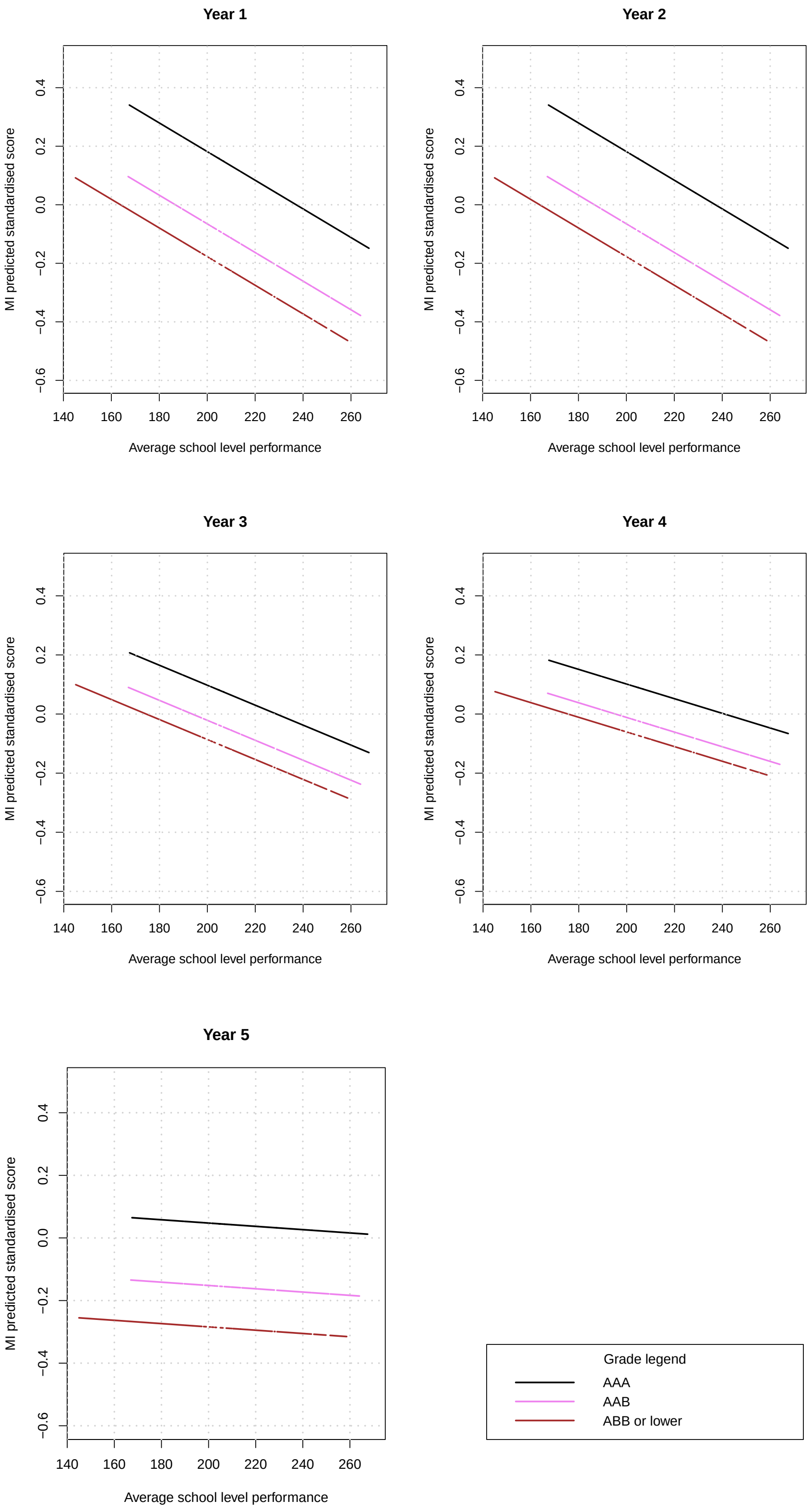

Figure 10: *Multiply imputed effect of average school level performance by reported grades on undergraduate medical school skills-based exams*

References

[1] IC McManus et al. “The UKCAT-12 study: educational attainment, aptitude test performance, demographic and socio-economic contextual factors as predictors of first year outcome in a cross-sectional collaborative study of 12 UK medical schools”. In: *BMC medicine* 11.1 (2013), p. 244.

[2] William Revelle and Richard E Zinbarg. “Coefficients alpha, beta, omega, and the glb: Comments on Sijtsma”. In: *Psychometrika* 74.1 (2009), pp. 145–154.

[3] Tenko Raykov. “Estimation of composite reliability for congeneric measures”. In: *Applied Psychological Measurement* 21.2 (1997), pp. 173–184.

[4] Jane Adam, Jon Dowell, and Rachel Greatrix. “Use of UKCAT scores in student selection by UK medical schools, 2006-2010”. In: *BMC medical education* 11.1 (2011), p. 98.

[5] Bengt O Muthén Linda K Muthén Tihomir Asparouhov. *Regression And Mediation Analysis Using Mplus*. Muthén & Muthén, 2016, pp. 61–62.

[6] Andrew F Hayes. *My Macros and Code for SPSS and SAS*. URL: http://afhayes.com/spss-sas-and-mplus-macros-and-code.html (visited on 26/01/2017).

[7] Andrew F. Hayes. *The PROCESS macro for SPSS and SAS*. URL: http://www.processmacro.org/index.html (visited on 12/07/2017).

[8] Daniel J Bauer, Kristopher J Preacher, and Karen M Gil. “Conceptualizing and testing random indirect effects and moderated mediation in multilevel models: new procedures and recommendations.” In: *Psychological methods* 11.2 (2006), p. 142.

[9] Geert Verbeke and Geert Molenberghs. *Linear mixed models for longitudinal data*. Springer Science & Business Media, 2009.

[10] Geert Molenberghs and Michael Kenward G. *Missing Data in Clinical Trials*. John Wiley & Sons Ltd, 2007. Chap. Multiple Imputation, pp. 110–114.

[11] Geert Molenberghs and Geert Verbeke. *Models for Discrete Longitudinal Data*. Springer Science & Business Media, 2005. Chap. Multiple Imputation and Expectation-Maximazation Algorithm, pp. 515–516.
