# Supplementary material for: What is the effect of secondary (high) schooling on subsequent medical school performance? A national, UK-based, cohort study
Source: BMJ Open. 2018 May 23;8(5):e020291. doi: 10.1136/bmjopen-2017-020291 (PMC5988088; doi:10.1136/bmjopen-2017-020291)
Supplement: Author's manuscript [file bmjopen-2017-020291.draft_revisions.pdf]

BMJ Open is committed to open peer review. As part of this commitment we make the peer review history of every article we publish publicly available.

When an article is published we post the peer reviewers' comments and the authors' responses online. We also post the versions of the paper that were used during peer review. These are the versions that the peer review comments apply to.

The versions of the paper that follow are the versions that were submitted during the peer review process. They are not the versions of record or the final published versions. They should not be cited or distributed as the published version of this manuscript.

BMJ Open is an open access journal and the full, final, typeset and author-corrected version of record of the manuscript is available on our site with no access controls, subscription charges or pay-per-view fees (http://bmjopen.bmj.com).

If you have any questions on BMJ Open's open peer review process please email info.bmjopen@bmj.com

BMJ Open

The impact of secondary (high) schooling on subsequent medical school performance: a national study

Journal:	*BMJ Open*
Manuscript ID	bmjopen-2017-020291
Article Type:	Research
Date Submitted by the Author:	25-Oct-2017
Complete List of Authors:	Mwandigha, Lazaro; University of York, Health Sciences<br>Tiffin, Paul; University of York, Health Sciences<br>Paton, Lewis ; University of York, Health Sciences<br>Kasim, Adetayo; Durham University, Wolfson Research Institute for Health and Wellbeing<br>Boehnke, Jan Rasmus; University of Dundee, School of Nursing and Health Sciences
**Primary Subject Heading**:	Medical education and training
Secondary Subject Heading:	Research methods
Keywords:	MEDICAL EDUCATION & TRAINING, STATISTICS & RESEARCH METHODS, EDUCATION & TRAINING (see Medical Education & Training)

SCHOLARONE™
Manuscripts

For peer review only

The impact of secondary (high) schooling on subsequent medical school performance: a national study

Lazaro M. Mwandigha, Paul A. Tiffin, Lewis W. Paton, Adetayo S. Kasim, Jan R. Böhnke

Author Details

LMM: Postgraduate student, Department of Health Sciences, University of York, Heslington, York, YO10 5DD, UK, email; lmm544@york.ac.uk

PAT(Corresponding author): Department of Health Sciences, University of York, Heslington, York, YO10 5DD, UK, Tel: 01904 321117, email; paul.tiffin@york.ac.uk

LWP: Research Fellow, Department of Health Sciences, University of York, Heslington, York, YO10 5DD, UK, email; lewis.paton@york.ac.uk

ASK: Senior Research Statistician, Wolfson Research Institute of Health and Wellbeing, Durham University, Queen's Campus, Stockton on Tees, TS17 6BH, UK, email; a.s.kasim@durham.ac.uk

JRB: Senior Research Fellow, Dundee Centre for Health and Related Research, School of Nursing and Health Sciences, University of Dundee, Dundee, DD1 4HJ, UK, AND Department of Health Sciences, University of York, Heslington, York YO10 5DD; email; j.r.boehnke@dundee.ac.uk

For peer review only

ABSTRACT

Objectives

Undergraduate academic achievement at medical school may be inversely related to the performance of the secondary (high) school an entrant attended. Indeed, some universities already offer 'grade discounts' to applicants from less well performing schools, as part of 'widening access' initiatives. At present evidence to guide such policies is lacking. In this study we analyse a national

For peer review only - http://bmjopen.bmj.com/site/about/guidelines.xhtml

dataset in order to understand the relationship between the two main predictors of medical school admission in the UK (educational achievement and performance on the United Kingdom Clinical Aptitude Test (UKCAT)) and subsequent undergraduate outcomes. Specifically, we explore the mediating role played by the performance of a candidate's secondary school in order to inform selection policy.

Methods

Data were available from a UK-based national, linked dataset. Medical school outcomes were available for the 2008 entry cohort. UKCAT scores and Prior Educational Attainment (PEA- indicated by secondary school exam grades achieved) were available for 2,107 entrants and were linked to secondary school-level performance data. A series of mediation models were developed and tested.

Results

The ability of the UKCAT scores to predict *knowledge*-based exam performance was significantly mediated by PEA in all the years of medical school. Undergraduate achievement was inversely related to secondary school-level performance. This effect waned over time and was less marked for *skills*, compared to *knowledge*-based undergraduate assessments. Thus, the predictive value of secondary school grades was dependent on the secondary school in which they were obtained.

For peer review only - http://bmjopen.bmj.com/site/about/guidelines.xhtml

Conclusions

The UKCAT scores added some value, above and beyond secondary school achievement, in predicting undergraduate performance, especially in the later years of study. Our findings suggest that the academic entry criteria should be relaxed for candidates applying from the least well performing secondary schools. In the UK, this would translate into a decrease of approximately one to two A-level grades.

ARTICLE SUMMARY

Strengths of the study

- This study managed to link schools and university data to conduct the first UK-based study UK that compares the academic performance of medical students drawn from poorly performing secondary schools against their counterparts from well-performing ones across all the five years of medical school
- The sample was relatively large with a total of 2,107 medical school students who matriculated in 2008 included in this study

Limitations of the study

- The *skills* and *knowledge*-based undergraduate assessment outcomes are local not nationally standardised measures
- There were relatively high rates of missing data in the latter years of the study, especially in relation to *skills*-based undergraduate

Word count, excluding title page, abstract, article summary, references, figures and tables is 5,295

For peer review only - http://bmjopen.bmj.com/site/about/guidelines.xhtml

INTRODUCTION

Internationally, there is high competition for places to study medicine, and the UK is no exception. Along with the academic demands of medicine as a subject, this has driven medical schools to use secondary (high) school performance as a major determinant to offer a place or not. In general, relatively high obtained (or predicted) grades at senior school are required before a candidate is considered as a potential entrant to medical courses. This emphasis on *prior educational attainment* ('PEA'- the grades obtained at formal exams during secondary education) has partly driven the over-representation of socio-economically privileged individuals in medicine. For example, in North America the majority of US medical school entrants are from relatively affluent backgrounds with around half coming from families in the top fifth for national income.[1] This issue is inevitably reflected in the educational backgrounds of students- it was recently highlighted that 80% of those studying medicine in the UK applied from only 20% of the country's secondary schools.[2]

It was partly with this in mind that 'aptitude' tests, mainly tapping into cognitive domains were introduced into medical selection.[3] Such aptitude tests were first used to compliment PEA in selection for undergraduate students in the USA in 1928 when the Medical College Admission Tests (MCAT) was developed to address high attrition rates in undergraduate medical school. [4, 5] Since this time the use of such tests for selection has spread to other parts of the world.[6-15] PEA has been demonstrated to have predictive validity for undergraduate medical school outcomes in Australia,[16] South Korea, [17] the UK,[18] Saudi Arabia,[19] India,[20] Czech Republic,[21] and New

For peer review only - http://bmjopen.bmj.com/site/about/guidelines.xhtml

Zealand.[22] Aptitude tests such as the *Medical College Admission Tests (MCAT)* in the US,[23] *Biomedical Admission Test (BMAT)* and *United Kingdom Clinical Aptitude Test (UKCAT)* in the UK,[11,24] *Undergraduate Medicine and Health Sciences Admission Test (UMAT)* in New Zealand,[25] *Hamburg Medical School Natural Science Test (HAM-Nat)* in Germany,[10] *Saudi National Aptitude Exam* in Saudi Arabia [19] and *Health Professions Admission Test- Ireland (HPAT-Ireland)* in Ireland [26] have predictive validity for medical school outcomes. Indeed, some critics have highlighted that such aptitude tests may tap into similar constructs as traditional metrics of academic achievement such as high school grades. If this is the case then such measures are unlikely to either facilitate widening access to medicine or add value within the selection process in general.

Some aptitude tests, such as the Biomedical Medical Admissions Test (BMAT) [8] and MCAT,[4] evaluate semantic knowledge of biomedical sciences. These tests may predict undergraduate medical performance, at least in the early years, but are unlikely to add predictive value above and beyond traditional measures of academic attainment.[27] Other tests place more weight on evaluating fluid concepts of cognitive ability, such as the UK Clinical Aptitude Test (UKCAT).[9] In the case of the UKCAT some, albeit modest, ability to predict undergraduate performance, even after controlling for the effects of secondary school achievement, has been demonstrated.[28] However, it is currently unclear how the predictive abilities of the UKCAT are mediated by PEA, and the extent to which this may vary across both the type of academic outcome and the five year period of undergraduate education in the UK. It has

For peer review only - http://bmjopen.bmj.com/site/about/guidelines.xhtml

been further suggested that the UKCAT scores may be somewhat less sensitive to the type of secondary school attended, compared to the A-levels sat by students in England and Wales in their final year of schooling.[29] A-levels, usually in three subject areas, are generally undertaken in the last two years of secondary schooling and are roughly equivalent to Advance Placement (AP) courses taken by some students in North America. Findings from an earlier, cross-sectional, study suggested that a strong use of the UKCAT scores during the admissions process may mitigate some of the disadvantage faced by certain under-represented groups applying to study medicine.[30] However, a subsequent study, using longitudinal data, did not report consistent effects over time in this regard.[31]

Whilst PEA does predict academic outcomes in higher education previous studies have observed an inverse relationship with the performance of the secondary (high) school attended. i.e. students from more highly performing schools tend to get poorer degree awards, after controlling for PEA.[32] To date, the evidence relating to this potential effect in medical school has been inconsistent. One national study observed such an effect in the first year of medical undergraduate training for overall academic performance.[24] A separate, local, study did not.[33] Certain medical courses, designed to widen access to medicine, already 'discounted' requirements for certain groups. For example, in Australia a scheme to encourage recruitment to remote, underserved areas, relaxes entry requirements for candidates from rural backgrounds.[34] In the US 'affirmative action' policies, albeit at times controversial and repeatedly legally challenged, have been implemented to

For peer review only - http://bmjopen.bmj.com/site/about/guidelines.xhtml

encourage those from under-represented ethnic groups to enter medical school.[35]. In the UK a number of universities have started to offer reduced academic entry requirements for A-level (high school) grades to students from disadvantaged backgrounds who have attended poorly performing secondary schools.[36, 37] Other medical schools are following suit.[38] However, evidence to support such admissions strategies is currently lacking. Thus the present study had two aims:

1. To determine the extent to which the predictive powers of the UKCAT are mediated via PEA, for two separate domains (*knowledge* and *skills*) over the period of undergraduate training. Since cognitive ability and educational attainment correlate, we attempt to achieve a more accurate assessment of the relative, and unique, contribution UKCAT scores make within the selection process.
2. To appraise the influence of the performance of the previous secondary school attended on an undergraduate's achievement in medical school. These results will usefully inform policy on grade discounting for applicants applying from poorly performing schools.

For this study we had an opportunity to link national data on the performance of secondary schools to cognitive ability (as evaluated via the UKCAT), PEA and outcomes at 18 UKCAT-consortium medical schools. Thus, there was also the possibility to better understand the interplay between secondary school-level performance, an individual's cognitive ability, their educational attainment (PEA) and how these related to subsequent undergraduate academic achievement. It

For peer review only - http://bmjopen.bmj.com/site/about/guidelines.xhtml

was therefore hoped that a relatively sophisticated approach to modelling could help understand the role of secondary schooling in both selection (partly based on PEA and aptitude test scores) and later attainment at undergraduate level.

Our findings will inform selection policy in medical school, and in particular provide guidance on the extent to which grades should be discounted for applicants from poorly performing secondary schools.

METHODS

Data availability and quality

UKCAT consortium medical schools are those medical schools that utilise UKCAT for selection in the UK. For this study, data were available for 18 UKCAT consortium medical schools in England and Wales for candidates who were enrolled between 2007 and 2013. However, Department for Education data on the performance of English secondary schools were only linked to the 2008 entry cohort. For this reason only data relating to these students were used in this study. It should be noted that an advantage of using the 2008 entry cohort was the relatively low attrition rate throughout the first four of the five year undergraduate period studied. As with similar previous studies, non-standard medical courses (e.g. 'widening participation', graduate entry etc) were excluded.[28] Only the marks attained at first sittings of undergraduate exams were retained for each student. Data relating to UKCAT scores and secondary school attainment were available for 2,107 students who entered medical

For peer review only - http://bmjopen.bmj.com/site/about/guidelines.xhtml

school in 2008 and had linked data relating to the performance of the secondary school they attended.

The secondary school exams sat by the students were nationally standardised and included General Certificate of Secondary Education (GCSE), Advanced Subsidiary (AS) Level and Advanced Level ('A-Level) exams. The GCSE exams are taken at around the age of 15-16 years. Those aspiring to eventually entering higher education usually take at least 10 subjects at GCSE level. At the time of the study, the AS levels were sometimes taken in the first year of sixth form (equivalent to high school junior year) as preparation for, or to supplement the full A-level exams taken the subsequent year. For those planning to apply for medicine three subjects at A-level are studied in the last two years of secondary schooling, almost always in the sciences. Candidates frequently take more than three A-levels though universities only count the highest three grades, that usually must be achieved at first sitting.

The completeness of the data relating to the outcomes of interest varied and the flow of the data in the study is depicted in Figure 1.

[Place Figure 1 about here]

The manner in which data related to undergraduate performance in the UKCAT consortium of universities has been collated and managed has been previously described.[28] However, to summarise, the main outcome variables used were the scores achieved at *knowledge* and *skills*-based end of year exams. It was

For peer review only - http://bmjopen.bmj.com/site/about/guidelines.xhtml

left to individual institutions to define how their assessments fell into each category. These assessment scores were provided by the universities in percentage forms (of maximum marks achievable) and then converted to standardised z-scores within each institution. Thus, the z scores were created by subtracting the mean performance for that particular year and medical school cohort from an entrant's score and dividing it by the standard deviation for their peers' scores. This created standardised scores with mean zero and a standard deviation of one for each medical school group of students. This standardisation was carried out in order to minimise the impact of any variability across medical schools, in terms of the nature of the assessment.

The UKCAT consists of four multiple choice sub-tests timed separately namely *quantitative reasoning, decision analysis*, *verbal reasoning* and *abstract reasoning. Quantitative reasoning* assesses an applicant's ability to critically evaluate information presented in numerical form; *decision analysis* assesses the ability to make sound decisions and judgements using complex information; *verbal reasoning* assesses the ability to critically evaluate information that is presented in a written form, and; *abstract reasoning* assesses the use of convergent and divergent thinking to infer relationships from information. Each of the cognitive subtests have their raw score converted to a scale score that ranges from 300 to 900. Therefore the total scale scores for all of the four subtests range from 1,200 to 3,600. The UKCAT subtests and their total scores were standardised as z-scores according to the scores for all candidates at the year of sitting. The reliability of the UKCAT subtests has previously been evaluated and reported.[39] For the purposes of this study only the total

For peer review only - http://bmjopen.bmj.com/site/about/guidelines.xhtml

UKCAT score (i.e. the summed total of all four sub-test scores) was used as a predictor. This is because it is the total score that is generally used in selection and represents a summary measure of all the four subtest scores.

In order to develop an overall, and precise, measure of PEA we implemented a novel approach that extended one previously used by McManus et al. [24, 40] This involved conceptualising 'educational achievement' as a common factor ('latent trait'). Latent traits cannot be observed or measured directly, only by their effects on behaviour. In terms of attitudes this could be observing certain responses to questionnaires, or in the case of ability, performance on exams and other assessments. Thus, in this case we treated all the commonly taken national exam grades (i.e. GCSE, AS and A-levels) as 'indicators' (i.e. observable markers) of an underlying ability (PEA). This approach allowed us to use information contained in all the commonly sat exams during secondary school in England to estimate the overall underlying educational achievement of an entrant. Because the specific method we used easily accommodated missing 'indicators' it was irrelevant if only a minority of entrants had taken a specific exam (e.g. history GCSE) and such grades could still be included when estimating PEA. The process resulted in a factor score estimate for each entrant which was provided as a standardised z score, where the mean was zero (average PEA for all applicants, with a standard deviation of 1). Thus this measure of previous educational achievement provided more information on an individual than merely their 'best of three' A-level grades.

For peer review only - http://bmjopen.bmj.com/site/about/guidelines.xhtml

This estimate of PEA was used in the models addressing the first study aim (evaluating the mediating effects of previous educational attainment on the UKCAT's ability to predict undergraduate performance). However, 'discounting' policy focuses on the 'best of three' A-level grades required for entry, usually after a provisional offer has been made to an applicant. Therefore for the models addressing the study's second aim (role of secondary school-level performance on undergraduate outcomes) we banded entrants into categories according to A-level grades. Thus, the entrants were grouped into three bands according to the highest three A-level grades achieved. Only 43 (2%) entrants were recorded as having the relatively low A-level grades 'BBB' and 'BBC'. Thus entrants were grouped into those with grades 'AAA', 'AAB' and 'ABB or lower'. Full details of the method are provided in the technical appendix.

English secondary school-level performance data for 2008 were available from the Department for Education (DfE). Thus for this study we defined secondary school-level performance as the average grades (converted to a numeric score) achieved for each student on roll at that educational establishment for that school year. Further details are available from the Department for Education for England website. In this sense 'performance' is (narrowly) defined as the average educational attainment, in terms of formal exam grades achieved, for each student on roll, in that educational establishment.

MODELLING APPROACHES

For peer review only - http://bmjopen.bmj.com/site/about/guidelines.xhtml

Modelling the relationship between UKCAT scores, PEA and Undergraduate outcomes

Our first aim was to try and understand the extent to which the ability of the UKCAT scores to predict subsequent undergraduate medical school performance were explained by PEA. To answer this question a mediation model was developed. The outcomes of interest (*knowledge* and *skills* exam results) were local to each participating medical school. The variation in the assessment results across institutions was initially explored using a multilevel modelling approach, but no statistically significant clustering effects by university were observed (for details refer to the technical appendix, section 2.3). For this reason, a simpler approach using a single-level mediation model was used for the analysis (Figure 2). Further details of the modelling process are described in section 2.1 of the technical appendix.

[Place Figure 2 about here]

Modelling the influence of secondary school performance on undergraduate outcomes

The second aim of the study was to evaluate the influence of the performance of an entrant's previous secondary school on subsequent undergraduate achievement. This involved estimating this secondary school-level effect while controlling for an entrant's A-level grades. A multilevel model was required to account for the variation in outcomes between universities.[41] Further details are supplied in the technical appendix. From the model we could derive

For peer review only - http://bmjopen.bmj.com/site/about/guidelines.xhtml

predictions about entrants' performances at medical school, for varying A-level grades and secondary school performance.

The statistical analyses were conducted using Mplus, R and SAS softwares. [42-44] Lucidchart [45] was used to produce the figures and R software was used for the graphs of the model predictions.

RESULTS

Descriptive statistics

The numbers of entrants with outcomes available in each category (type and year) are depicted in Table 1. This was not a cohort study in the conventional sense (i.e. entrants could leave and enter the study at any year). Thus, Table 1 also illustrates the attrition for only those entrants who had reported *knowledge* and *skills* outcomes in the first year of undergraduate medical school. This is to provide a picture of attrition in the conventional sense (i.e. how many participants at baseline remained at subsequent time-points).

	**Knowledge outcome**				**Skills outcome**	
**Academic Year**	**Number of universities**	**Number of students**	**% *Attrition***	**Number of universities**	**Number of students**	**% *Attrition***
1	13	1,453	-	9	1,051	-
2	13	1,404	3.72	9	1,019	3.04
3	11	1,041	28.36	7	729	30.64
4	7	711	51.07	5	668	36.44
5	4	439	69.79	2	260	75.26

Table 1: Study attrition rates due to missing data only for those students who had outcome measures reported in year one of medical school

Supplementary Tables 9 and 10 in the technical appendix provide a detailed summary of the missing data patterns for the outcomes. Of the 2,107

For peer review only - http://bmjopen.bmj.com/site/about/guidelines.xhtml

undergraduate medical school entrants, 1,855 had their secondary school-level performance available. The distribution of secondary school-level performance and UKCAT scores achieved by the entrants are depicted in Table 2.

	Year of UKCAT sitting=2007				
	**Sample size**	**Mean**	**SD**	**Minimum**	**Maximum**
Average Secondary School-level performance	1,855	225.18	20.09	145	267.5
UKCAT total score	2,107	2,544.47	188.92	1,950	3,190

Table 2: Descriptive statistics of the UKCAT total score and average point entry for the 2,107 entrants from the 987 schools.

Table 3 shows the distribution of A-level grades for the medical school entrants. Note that the majority of the entrants had achieved either AAA or AAB grades at A-level.

Grade	N	%
Missing	36	1.71
AAA	1,463	69.44
AAB	436	20.69
ABB	129	6.12
BBB	29	1.38
BBC	14	0.66

Table 3: A-level grades for the entrants in the study sample.

The prediction of medical school outcomes from UKCAT performance

For peer review only - http://bmjopen.bmj.com/site/about/guidelines.xhtml

Figure 3 summarises the results from the models investigating the potential mediating effects of PEA on the relationship between UKCAT scores and undergraduate exam outcomes. The proportion of the predictive power of the UKCAT explained by PEA shown for both *knowledge* and *skills*-based undergraduate medical school outcomes are computed as a quotient of indirect effect of UKCAT through PEA divided by total effect of UKCAT.

[Place Figure 3 about here]

Overall, PEA explains approximately over 43% (dotted black line in the Figure 3) of the statistically significant predictive power of the UKCAT for both *knowledge* and *skills* based exams only in the preclinical years (one and two) of undergraduate medical school training. For the clinical years (three to five) PEA explains approximately less than 43% of the predictive power of the UKCAT for both *knowledge* and *skills*-based exam outcomes. This proportion remains statistically significant but declines somewhat with every subsequent year of training. The full values of the total, direct and indirect effects are depicted in Supplementary Tables 5 to 8 of the technical appendix.

The effect of secondary school-level performance on subsequent medical school performance

For peer review only - http://bmjopen.bmj.com/site/about/guidelines.xhtml

Both secondary school-level performance and PEA were statistically significantly related to the undergraduate outcomes. No statistically significant interaction was observed between the two variables. Overall, compared to entrants from secondary schools with a high average student performance, those from schools with lower average attainment tended to have better subsequent scores in both *knowledge* and *skills* exams.

We intended to make our results relevant to UK medical selectors. Specifically we wished to estimate the level of 'discounting' that should be offered to applicants from disadvantaged educational backgrounds. Thus the results of our models addressing the second study aim are depicted in Figures 4 and 5. We show the actual and predicted (fitted) values from the models in the Figures. Average secondary school performance (mean enrolled student attainment for all secondary schools in England) is shown on the horizontal axis and predicted medical school performance (as a standardized z score) on the vertical axis. Figure 4 depicts the values in relation to *knowledge*-based exams, according to secondary school-level performance. Similarly, Figure 5 shows the values for *skills*-based outcomes. Superimposed on these plotted values are the estimates (with associated 95% confidence bands) for entrants depending on their A-level grades at admission to university. These represent the entrants within the three bands of A-level attainment ('AAA', 'AAB', and 'ABB or lower'). For purpose of demonstration, the horizontal black dotted lines indicate the equivalent level of performance between those entrants from secondary schools at the lower decile of performance and those at the upper decile.

For peer review only - http://bmjopen.bmj.com/site/about/guidelines.xhtml

There are a number of notable trends observed in these graphs. Firstly, students with higher A-level grades outperform those with lower educational achievement. However, this gap narrows when predicting *skills*, rather than *knowledge*-based outcomes in medical school. The difference also reduces in magnitude as undergraduate education progresses through the years. Indeed for *skills*-based outcomes, and for many of the later years, the confidence intervals for the groups' estimates generally overlap. This indicates no statistically significant inter-group differences between those with 'AAB' and 'ABB or lower grades' at the 95% confidence level.

The second most striking feature, and the focus of this study, is that students from less highly performing secondary schools generally outperform those from more highly performing educational institutions for any given A-level grade banding. That is, controlling for the effects of A-level attainment, on average, those from the more poorly performing schools tend achieve better undergraduate exam results than those from the schools with higher levels of student attainment. The vertical purple and brown dotted lines highlight this feature. They show that those with lower A-level grades (e.g. AAB or ABB) from the lowest performing secondary schools tend to have equivalent undergraduate performance to those entrants from the highest performing educational establishments with top grades (i.e. AAA). It is also notable that this 'secondary school gradient' is generally steepest for *knowledge*-based outcomes in the early years of undergraduate study. Thus, the effects of secondary school environment, as with individual previous educational

For peer review only - http://bmjopen.bmj.com/site/about/guidelines.xhtml

attainment, tends to be less marked for procedural (*skills*-based) learning and with advancing time in university study.

[Place Figure 4 about here]

[Place Figure 5 about here]

DISCUSSION

The findings from previous studies suggested some modest added value of the UKCAT scores to predict undergraduate performance, over and above that provided by conventional measures of academic achievement.[24, 28] Further, the ability of UKCAT scores to predict certain aspects of undergraduate performance was found to be largely independent of prior educational attainment (PEA). This was less true for both *knowledge* and based exams and *skills*, taken early on in the preclinical years of medical school, where a significant portion of the UKCAT's predictive ability is mediated via previous educational performance.

Our findings on the role of secondary school quality in determining subsequent undergraduate performance are in line with the findings from a previous national study utilising data from the same cohort, as well as more general analysis of data from higher education in England.[24, 32] However, we were able to demonstrate persistence (though attenuation) of these effects over the five years of medical school. Our findings were also somewhat in keeping with those from an Australian study. This reported that entrants from rural backgrounds tended to have lower educational achievement, both at entry and in the early,

For peer review only - http://bmjopen.bmj.com/site/about/guidelines.xhtml

pre-clinical years of study. However there were no significant inter-group differences in performance observed in the latter, clinical years of undergraduate training. However, some caution must be exercised in interpreting these findings as the study was single site with a relatively small number (N=856) of participating students.[34] The present findings were in contrast to those of a local study, which focussed on the fourth year of medical school, when the effects of secondary schooling are likely to have been less marked.[33] The relatively low numbers of students (N=574) involved in this latter study may have led to a deficiency in study power and thus an inability to demonstrate these effects. Also, by using a more sophisticated approach to statistical modelling we were able to delineate the direct and indirect (mediational) effects of secondary school-level performance, educational achievement and cognitive ability (as assessed via the UKCAT) in determining undergraduate medical academic performance. This highlighted the shifting relative roles that conventional academic achievement versus cognitive ability play as undergraduate training progresses. We were also able to separate, at least crudely, outcomes in this study relating to '*knowledge*' and '*skills*' (see also limitations, below). As expected, traditional academic attainment (in the form of PEA), was more predictive and mediated a greater proportion of the UKCAT effects for earlier exam performance. We also observed a narrowing of the effects of secondary education achievement as medical school progressed. This might be expected- as the time since leaving secondary schooling elapses it becomes less relevant to current academic performance. However, this narrowing gap also suggests a positive influence of the university educational environment, which renders prior disparities in educational achievement

For peer review only - http://bmjopen.bmj.com/site/about/guidelines.xhtml

between students less influential. Moreover, as medical school progresses there is an increasing emphasis on procedural (*skills*-based) learning. Thus, the academic abilities required to highly achieve at written school exams are likely to become less relevant to performance.

Our findings also build on previous research[24] and we were able to demonstrating the value, to some extent of 'contextualising' secondary school achievement across the medical undergraduate years. That is, to some extent, the grades obtained by a student at secondary school must be put in the context of the educational establishment in which they were obtained. A reduction of one to two A-level grades may not appear to be a large adjustment. However, this must be understood in the light of the highly homogenous nature of both medical school applicants and entrants where high proportions obtain the maximum achievable grades. Thus, even one grade difference could represent a standard deviation or so from the mean in a pool of high achieving medical school entrants. Internationally, selectors must understand their equivalent effects, not just for school-type attended, but a range of contextual factors that may be pertinent to their culture. Similarly, they must translate such effects into discounted offers where appropriate, in the metric of their own educational systems.

The main strength of this study is that there were a relatively large number of entrants studied from a range of UK medical schools involved. This provided sufficient study power to enable the elicitation of relatively subtle effects and suggests the findings are generalizable to England and Wales. Moreover, the

For peer review only - http://bmjopen.bmj.com/site/about/guidelines.xhtml

secondary school exams sat by this cohort were nationally standardised, with only a minority of the credits awarded for course work. Thus, any local or regional variation in standards can be assumed to be trivial. Nevertheless a number of limitations must be borne in mind when interpreting the findings. In terms of the outcome measures, the categorisation of undergraduate exams into *skills* and *knowledge* was not operationalised and therefore rely on the participating medical schools to categorise the evaluations. Thus their definition may vary across medical schools. Whilst some of this variation was handled by the use of multilevel modelling a more robust definition of '*skills*' based assessments may have been helpful in predicting clinically-orientated performance, which may have been a more faithful proxy for later medical practice. In this regard, a methodology has been proposed to achieve this through the "nationalisation" of "local" measures of undergraduate medical school performance for fair comparisons of graduating medical doctors.[46] In addition, scores from the most recently taken UKCAT scores were used. These may not have been a better metric of underlying cognitive ability (being less prone to practice effects), though some early sittings may have been used as 'practice runs' by medical school applicants. In addition, the most recent UKCAT test results are those used by selectors, thus the ones most relevant to selection policy.

The number of participating universities in the study varied from year to year with higher levels of missing data for *skills*-based assessments (compared to *knowledge*) and for the latter years of study. Fortunately, the missing data did not adversely impact the results and conclusion of the study. The results from

For peer review only - http://bmjopen.bmj.com/site/about/guidelines.xhtml

imputed versus non-imputed datasets can be compared as a form of sensitivity analysis (see section 4 of the technical appendix). These highlight that the results did not vary significantly between imputed versus non-imputed datasets.

It should also be noted that this was not a classical cohort study as subsequent years were not a subset of the original entry cohorts where participants joined or left the study mainly dependent on their medical school participating that specific year. Thus, again, some caution must be exercised when interpreting the affected results.

The quality of secondary schools previously attended by undergraduate medical school entrants varies widely across the UK. However, the fact that 80% of UK medical students come from 20% of secondary schools[2] and tend to come from economically advantaged backgrounds.[47] Thus students from selective, academically high-performing schools are grossly over- represented at medical school. Indeed, a selection process substantially based on predicted or actual A-level performance will greatly advantage applicants from such educational institutions. Paradoxically such students, once admitted, may relatively underperform in medical school, compared to their contemporaries from less well performing schools, which tend to be state funded and non-selective in nature. Already some UK medical schools are offering 'discounted' A-level offers to applicants from schools that have students with lower levels of academic attainment.[48-50] Our results suggest that such medical schools may have been (albeit serendipitously) implementing such polices broadly in line with our present findings. That is to say, entrants from the most poorly

For peer review only - http://bmjopen.bmj.com/site/about/guidelines.xhtml

performing schools have A-level grades that 'worth' one to two grades more than those from the top performing schools, in terms of their ability to predict undergraduate achievement. As can be seen from Figures 4 and 5 the definition of 'low' and 'high' performing secondary school is somewhat subjective. In addition, the suggested 'discounting' would vary according to the outcome of interest. There are also practical challenges to implementing such policies. Not all applicants to medical school will have attended schools which can supply comparable data on their institutional performance. At present even comparison across the three nations making up the UK would be very difficult. One simple way of 'equating' across countries might be to report an applicant's rank within their school. However, further evaluation would have to be performed to assess whether such a relatively crude approach was an effective way of contextualising educational achievement. There is also the possibility of 'gaming' with economically advantaged families strategically placing a student in a less well performing educational institution for the final year of schooling.

Any moves to widen access to medicine may prove controversial, as advantaging certain candidates necessarily means disadvantaging others. Thus, such policies must be based on defensible evidence, such as the kind we believe is offered by this study. Moreover, given the very low absolute numbers of applicants and entrants to medical schools from disadvantaged socioeconomic backgrounds only a radical rethinking of 'widening access' is likely to result in substantial changes to the demographics of the medical workforce.

For peer review only

To conclude, we found that the predictive ability of the UKCAT can be explained to some degree by PEA, although this is more pronounced in the early preclinical years of undergraduate school. Significant effects of secondary school-level performance exist which suggest the issue of whether offers of a place to study should be discounted for students from more poorly performing schools. This highlights an urgent need to 'contextualise' secondary school performance in applicants rather than selectors taking grades at face value.

For peer review only

For peer review only - http://bmjopen.bmj.com/site/about/guidelines.xhtml

DECLARATIONS

Contributors

All authors made substantial contribution to this study. LMM conducted the statistical analyses and contributed to the writing of the article. PAT and ASK led the conception, design, supervision of the statistical analyses, interpretation of the results and contributed to the writing of the article. JRB was involved with supervision of the statistical analysis and handling of missing data, interpretation of the results, revising and writing of the manuscript. LWP was involved in the interpretation of the results, revising, writing, and critical appraisal of the manuscript. All authors have approved the final version of the article submitted.

Acknowledgements

The authors thank Rachel Greatrix at UKCAT for assistance with facilitating access to the data used in this study.

Funding

LMM is supported in his PhD, of which this study is a component, via funding from the UKCAT Board. PAT is currently supported in his research by a National Institute for Healthcare Research (NIHR) Career Development Fellowship.

This paper presents independent research part-funded by the National Institute for Health Research (NIHR). The views expressed are those of the authors and not necessarily those of the NHS, the NIHR or the Department of Health.

For peer review only - http://bmjopen.bmj.com/site/about/guidelines.xhtml

Competing interests

LMM is supported in his PhD project via funding from the UKCAT Board and has received travel expenses incurred for attending a UKCAT Research Group meeting. PAT has previously received research funding from the Economic and Social Research Council (ESRC), the Engineering and Physical Sciences Council (EPSRC), the Department of Health for England, the UKCAT Board, and the General Medical Council (GMC). In addition, PAT has previously performed consultancy work on behalf of his employing University for the UKCAT Board and Work Psychology Group and has received travel and subsistence expenses for attendance at the UKCAT Research Group.

Ethical approval

No human subjects were tested for this study therefore no ethical approval was necessary. The anonymised raw data used in these analyses were made available by the UK Medical Education Database (www.ukmed.ac.uk) following approval of an application.

For peer review only

For peer review only - http://bmjopen.bmj.com/site/about/guidelines.xhtml

REFERENCES

1. Grbic D, Jones DJ, Case ST. The role of socioeconomic status in medical school admissions: validation of a socioeconomic indicator for use in medical school admissions. Academic Medicine. 2015 Jul 1;90(7):953-60.

2. Medical School Council. Selecting for Excellence Final Report [Internet]. 2014 [cited 2017 March 03]. Available from: https://www.medschools.ac.uk/media/1203/selecting-for-excellence-final-report.pdf

3. United Kingdom Clinical Aptitude Test (UKCAT) Consortium. UKCAT Annual Report [Internet]. 2007 [cited 2017 September 09]. Available from: https://www.ukcat.ac.uk/media/1135/annual-report-2007-2008.pdf

4. Association of American Medical Colleges (AAMC). About the Medical College Aptitude Test [Internet]. 2017 [cited 2017 June 06]. Available from: https://students-residents.aamc.org/applying-medical-school/taking-mcat-exam/about-mcat-exam/

5. McGaghie WC. Assessing readiness for medical education: evolution of the medical college admission test. Journal of the American Medical Association. 2002 Sep 4;288(9):1085-90.

6. Australian Council for Educational Research (ACER). Health Professions Admission Test-Ireland (HPAT-Ireland)[Internet]. 2017 [cited 2017 March 03]. Available from: https://hpat-ireland.acer.org/

For peer review only - http://bmjopen.bmj.com/site/about/guidelines.xhtml

7. O'Flynn S, Fitzgerald T, Mills A. Modelling the impact of old and new mechanisms of entry and selection to medical school in Ireland: who gets in?. Irish Journal of Medical Science. 2013 Sep 1;182(3):421-7.

8. Cambridge Assessment Admissions Testing. The BioMedical Admissions Test (BMAT) [Internet]. 2017 [cited 2017 March 03]. Available from: http://www.admissionstestingservice.org/for-test-takers/bmat/

9. United Kingdom Clinical Aptitude Test (UKCAT) Consortium. UKCAT [Internet]. 2017 [cited 2017 March 03]. Available from: https://www.ukcat.ac.uk/

10. Hissbach JC, Klusmann D, Hampe W. Dimensionality and predictive validity of the HAM-Nat, a test of natural sciences for medical school admission. BMC Medical Education. 2011 Oct 14;11(1):83.

11. Emery JL, Bell JF. The predictive validity of the BioMedical Admissions Test for pre-clinical examination performance. Medical Education. 2009 Jun 1;43(6):557-64.

12. Australian Council for Educational Research (ACER). Undergraduate Medicine and Health Sciences Admissions Test (UMAT) [Internet]. 2017 [cited 2017 March 03]. Available from: https://umat.acer.edu.au/

13. Hissbach J, Klusmann D, Hampe W. Reliability of a science admission test (HAM-Nat) at Hamburg medical school. GMS Zeitschrift für Medizinische Ausbildung. 2011;28(3).

14. Farrokhi-Khajeh-Pasha Y, Nedjat S, Mohammadi A, Rad EM, Majdzadeh R, Monajemi F, Jamali E, Yazdani S. The validity of Iran's national university entrance examination (Konkoor) for predicting medical students' academic performance. BMC Medical Education. 2012 Jul 28;12(1):60.

For peer review only - http://bmjopen.bmj.com/site/about/guidelines.xhtml

15. Khan JS, Biggs JS, Bano T, Mukhtar O, Tabasum S, Mubasshar MH. Medical Colleges Admission Test in Punjab, Pakistan. Journal of Ayub Medical College Abbottabad. 2013 Jun 1;25(1-2):64-7.

16. Mercer A, Puddey IB. Admission selection criteria as predictors of outcomes in an undergraduate medical course: A prospective study. Medical Teacher. 2011 Dec 1;33(12):997-1004.

17. Kim T, Chang J-Y, Myung SJ, Chang Y, Park KD, Park WB, et al. Predictors of undergraduate and postgraduate clinical performance: A longitudinal cohort study. Journal of Surgical Education. 2016;73(4):715-20.

18. McManus IC, Woolf K, Dacre J, Paice E, Dewberry C. The academic backbone: longitudinal continuities in educational achievement from secondary school and medical school to MRCP (UK) and the specialist register in UK medical students and doctors. BMC Medicine. 2013 Nov 14;11(1):242.

19. Al Alwan I, Al Kushi M, Tamim H, Magzoub M, Elzubeir M. Health sciences and medical college preadmission criteria and prediction of in-course academic performance: a longitudinal cohort study. Advances in Health Sciences Education. 2013 Aug 1;18(3):427-38.

20. Gupta N, Nagpal G, Dhaliwal U. Student performance during the medical course: role of pre-admission eligibility and selection criteria. National Medical Journal of India. 2013 Jul-Aug ; 26 (4): 223-226.

21. Štuka Č, Martinková P, Zvára K, Zvárová J. The prediction and probability for successful completion in medical study based on tests and pre-admission grades. Stanisław Juszczyk. 2012 Jan 1:138.

For peer review only - http://bmjopen.bmj.com/site/about/guidelines.xhtml

22. Shulruf B, Poole P, Wang GY, Rudland J, Wilkinson T. How well do selection tools predict performance later in a medical programme?. Advances in Health Sciences Education. 2012 Dec 1;17(5):615-26.

23. Julian ER. Validity of the Medical College Admission Test for predicting medical school performance. Academic Medicine. 2005 Oct 1;80(10):910-7.

24. McManus IC, Dewberry C, Nicholson S, Dowell JS. The UKCAT-12 study: educational attainment, aptitude test performance, demographic and socio-economic contextual factors as predictors of first year outcome in a cross-sectional collaborative study of 12 UK medical schools. BMC Medicine. 2013 Nov 14;11(1):244.

25. Poole P, Shulruf B, Rudland J, Wilkinson T. Comparison of UMAT scores and GPA in prediction of performance in medical school: a national study. Medical Education. 2012 Feb 1;46(2):163-71.

26. Kelly ME, Regan D, Dunne F, Henn P, Newell J, O'Flynn S. To what extent does the Health Professions Admission Test-Ireland predict performance in early undergraduate tests of communication and clinical skills?–An observational cohort study. BMC Medical Education. 2013 May 10;13(1):68.

27. McManus IC, Ferguson E, Wakeford R, Powis D, James D. Predictive validity of the Biomedical Admissions Test: an evaluation and case study. Medical Teacher. 2011 Jan 1;33(1):53-7.

28. Tiffin PA, Mwandigha LM, Paton LW, Hesselgreaves H, McLachlan JC, Finn GM, Kasim AS. Predictive validity of the UKCAT for medical school undergraduate performance: a national prospective cohort study. BMC Medicine. 2016 Sep 17;14(1):140.

For peer review only - http://bmjopen.bmj.com/site/about/guidelines.xhtml

29. Tiffin PA, McLachlan JC, Webster L, Nicholson S. Comparison of the sensitivity of the UKCAT and A Levels to sociodemographic characteristics: a national study. BMC Medical Education. 2014 Jan 8;14(1):7.

30. Tiffin PA, Dowell JS, McLachlan JC. Widening access to UK medical education for under-represented socioeconomic groups: modelling the impact of the UKCAT in the 2009 cohort. British Medical Journal. 2012 Apr 17;344:e1805.

31. Mathers J, Sitch A, Parry J. Population-based longitudinal analyses of offer likelihood in UK medical schools: 1996–2012. Medical Education. 2016 Jun 1;50(6):612-23.

32. Higher Education Funding Council for England (HEFCE). Differences in degree outcomes: Key findings (UMAT) [Internet]. 2014 [cited 2017 March 03]. Available from: http://www.hefce.ac.uk/pubs/year/2014/201403/

33. Thiele T, Singleton A, Pope D, Stanistreet D. Predicting students' academic performance based on school and socio-demographic characteristics. Studies in Higher Education. 2016 Aug 2;41(8):1424-46.

34. Ray R, Woolley T, Sen Gupta T. James Cook University's rurally orientated medical school selection process: quality graduates and positive workforce outcomes. Rural and Remote Health. 2015 Oct 7;15:1-1.

35. Blake V. Affirmative action and medical school admissions. American Medical Association Journal of Ethics. 2012 Dec 1;14(12):1003.

36. Garlick PB, Brown G. Widening participation in medicine. BMJ: British Medical Journal. 2008 May 17;336(7653):1111.

37. Curtis S, Blundell C, Platz C, Turner L. Successfully widening access to medicine. Part 2: Curriculum design and student progression. Journal of the Royal Society of Medicine. 2014 Oct;107(10):393-7.

For peer review only - http://bmjopen.bmj.com/site/about/guidelines.xhtml

38. University of Birmingham. Medicine and Surgery MBChB: Widening Participation [Internet]. 2017 [cited 2017 March 03]. Available from: http://www.birmingham.ac.uk/undergraduate/courses/med/medicine.aspx?OpenSection=EntryRequirements

39. Tiffin PA. Understanding the Dimensionality and Reliability of the Cognitive Scales of the UK Clinical Aptitude test (UKCAT): Summary Version of the Report [Internet]. 2013 [cited 2017 March 03]. Available from: https://www.ukcat.ac.uk/media/1182/understanding-the-dimensionality-and-reliability-of-the-cognitive-scales-of-the-ukcat.pdf.

40. McManus IC, Dewberry C, Nicholson S, Dowell JS. The UKCAT-12 Technical Report [Internet]. 2012 [cited 2017 March 03]. Available from: https://www.ukcat.ac.uk/media/1185/ukcat-technicalreport-march2012-withbackgroundandsummary-sep2013v3.pdf.

41. Verbeke G, Molenberghs G. Linear mixed models for longitudinal data New York: Springer-Verlag; 2009.

42. Sas Institute. SAS software, version 9.4. SAS Institute Cary, NC; 2002.

43. Muthén LK, Muthén BO. Mplus Version 7 user's guide. Los Angeles, CA: Muthén & Muthén. 2012.

44. R Core Team (2017). R: A language and environment for Statistical computing. R Foundation for Statistical Computing, Vienna, Austria. ISBN 3-900051-07-0, URL http://www.R-project.org.

45. Lucid Software Inc. Lucidchart [Internet]. 2017 [cited 2017 March 03]. Available from: https://www.lucidchart.com/

For peer review only - http://bmjopen.bmj.com/site/about/guidelines.xhtml

46. Tiffin PA, Paton LW. Exploring the validity of the 2013 UKCAT SJT-prediction of undergraduate performance in the first year of medical school: Summary Version of Report [Internet]. 2017 [cited 2017 September 19]. Available from: https://www.ukcat.ac.uk/media/1119/exploring-the-validity-of-the-2013-ukcat-sjt-prediction-of-ug-performance-in-1st-yr-of-med-school-summary-version-posted-27032017.pdf.

47. Steven K, Dowell J, Jackson C, Guthrie B. Fair access to medicine? Retrospective analysis of UK medical schools application data 2009-2012 using three measures of socioeconomic status. BMC Medical Education. 2016 Jan 13;16(1):11.

48. King's College London. Extended Medical Degree Programme MBBS [Internet]. 2017 [cited 2017 July 13]. Available from: https://www.kcl.ac.uk/study/undergraduate/courses/extended-medical-degree-programme-mbbs.aspx.

49. University of Birmingham. Access to Birmingham (A2B) [Internet]. 2017 [cited 2017 July 13]. Available from: http://www.birmingham.ac.uk/undergraduate/preparing-for-university/teachers-advisors/reaching-regional/a2b.aspx.

For peer review only - http://bmjopen.bmj.com/site/about/guidelines.xhtml

50. University of Birmingham. Routes to the Professions [Internet]. 2017 [cited 2017 July 13]. Available from: http://www.birmingham.ac.uk/undergraduate/preparing-for-university/teachers-advisors/reaching-regional/routes-professions.aspx.

For peer review only

For peer review only - http://bmjopen.bmj.com/site/about/guidelines.xhtml

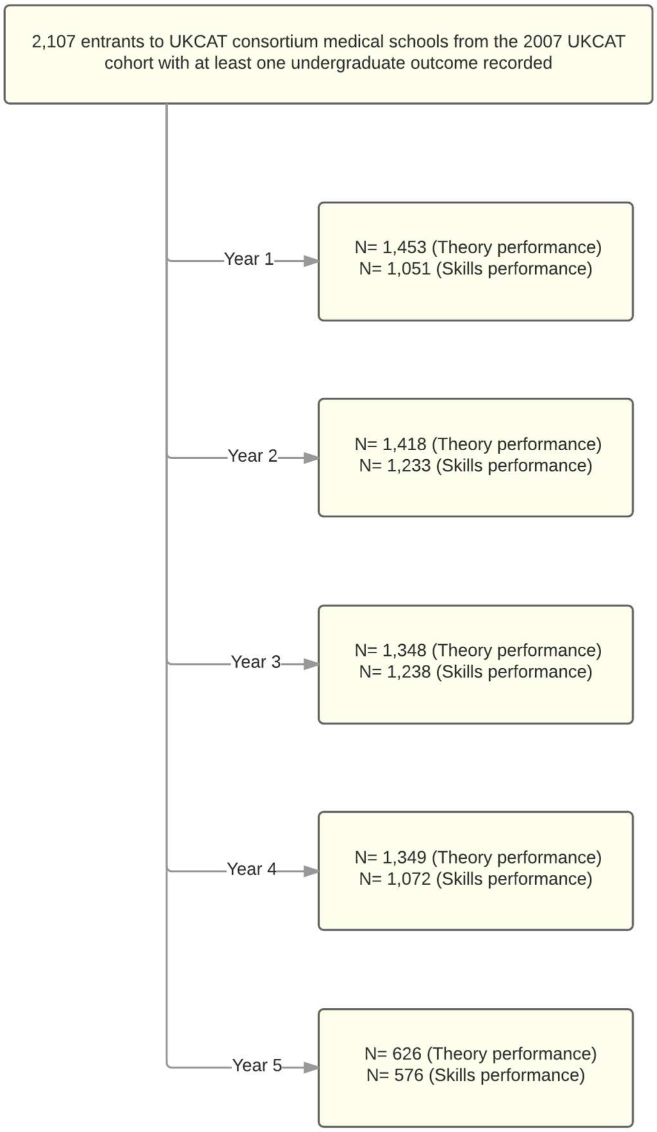

Figure 1: Flow chart of data available for the outcomes for each of the five academic years of medical school training

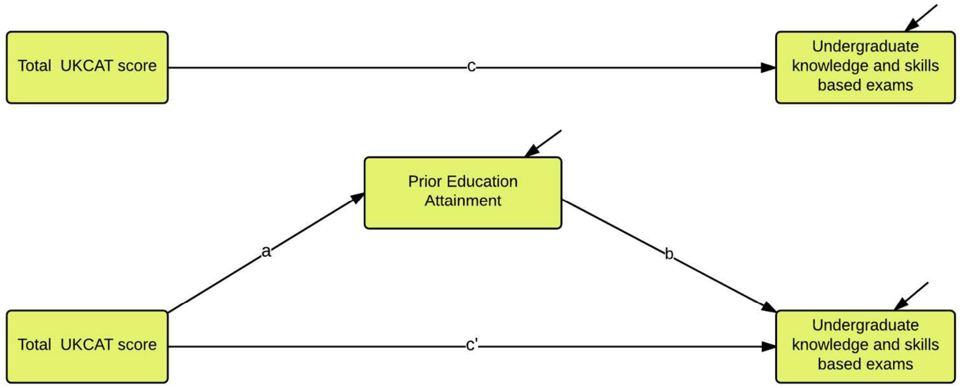

Figure 2: Illustration of the conceptual model for the single level mediation effect of previous educational attainment on the association between total UKCAT scores and undergraduate medical school knowledge and skills-based exams.

For peer review only

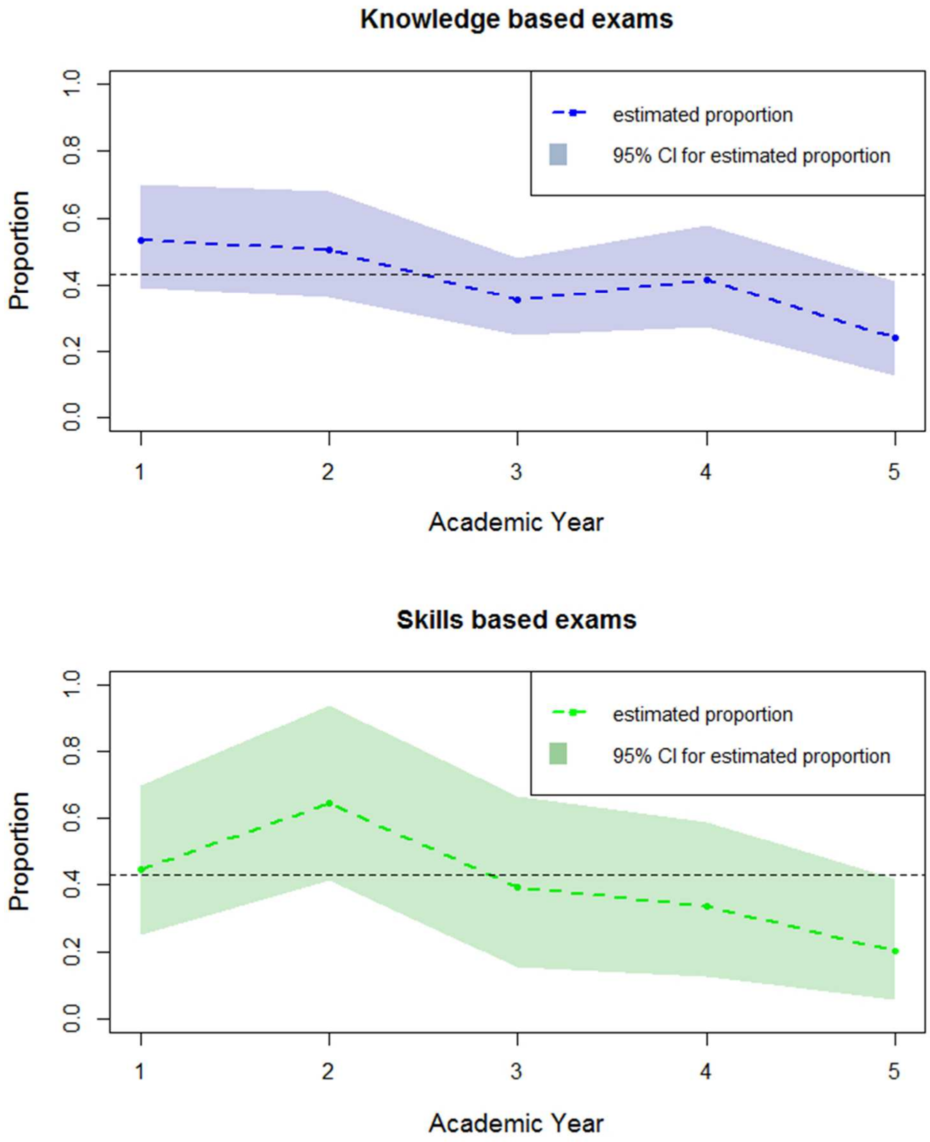

Figure 3: Proportion of the predictive power of UKCAT for knowledge and skills based exam outcomes explained by PEA in undergraduate medical school.

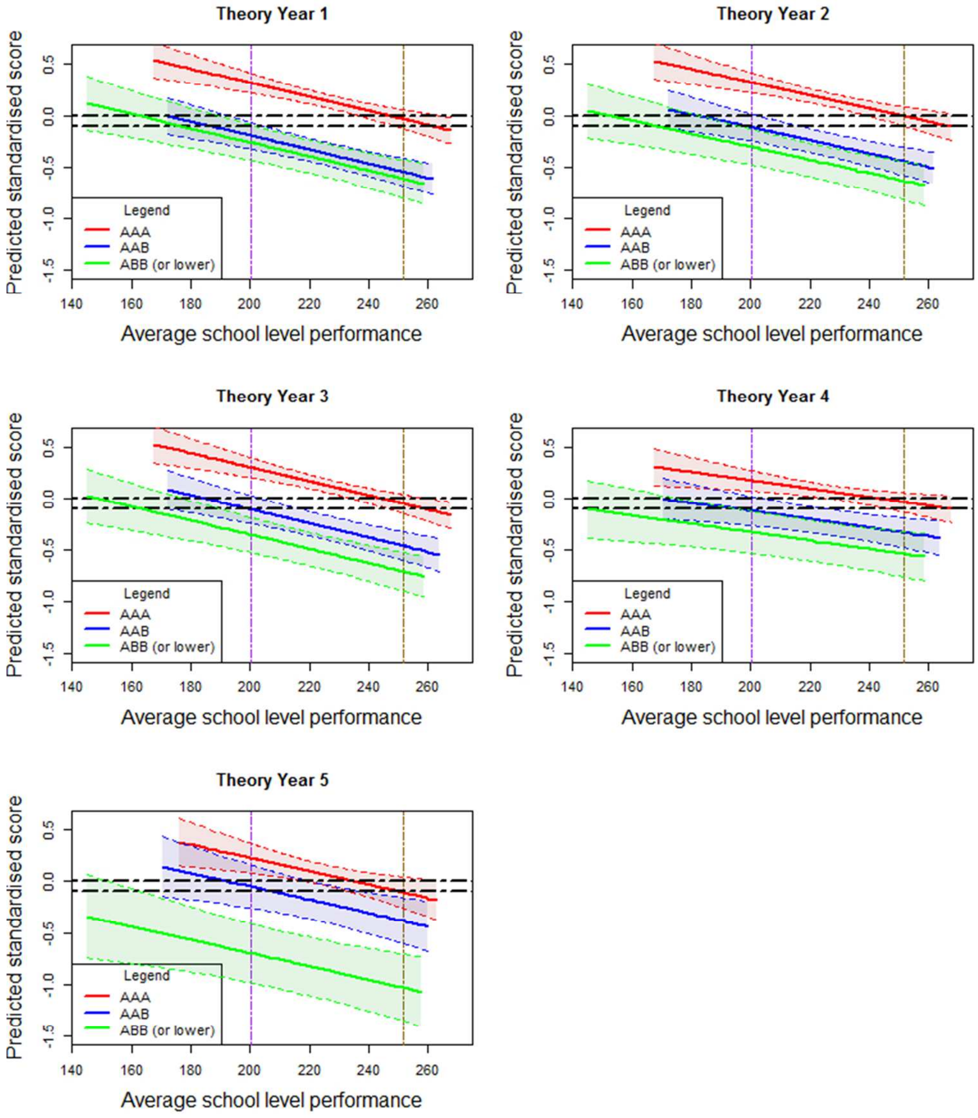

Figure 4: Effect of average school level performance by reported grades on undergraduate medical school knowledge based exams for all secondary schools in England in 2008. The 2nd and 8th deciles are denoted by the purple and brown vertical lines. The horizontal lines are arbitrary points chosen to demonstrate equivalent level of performance across the different grade levels.

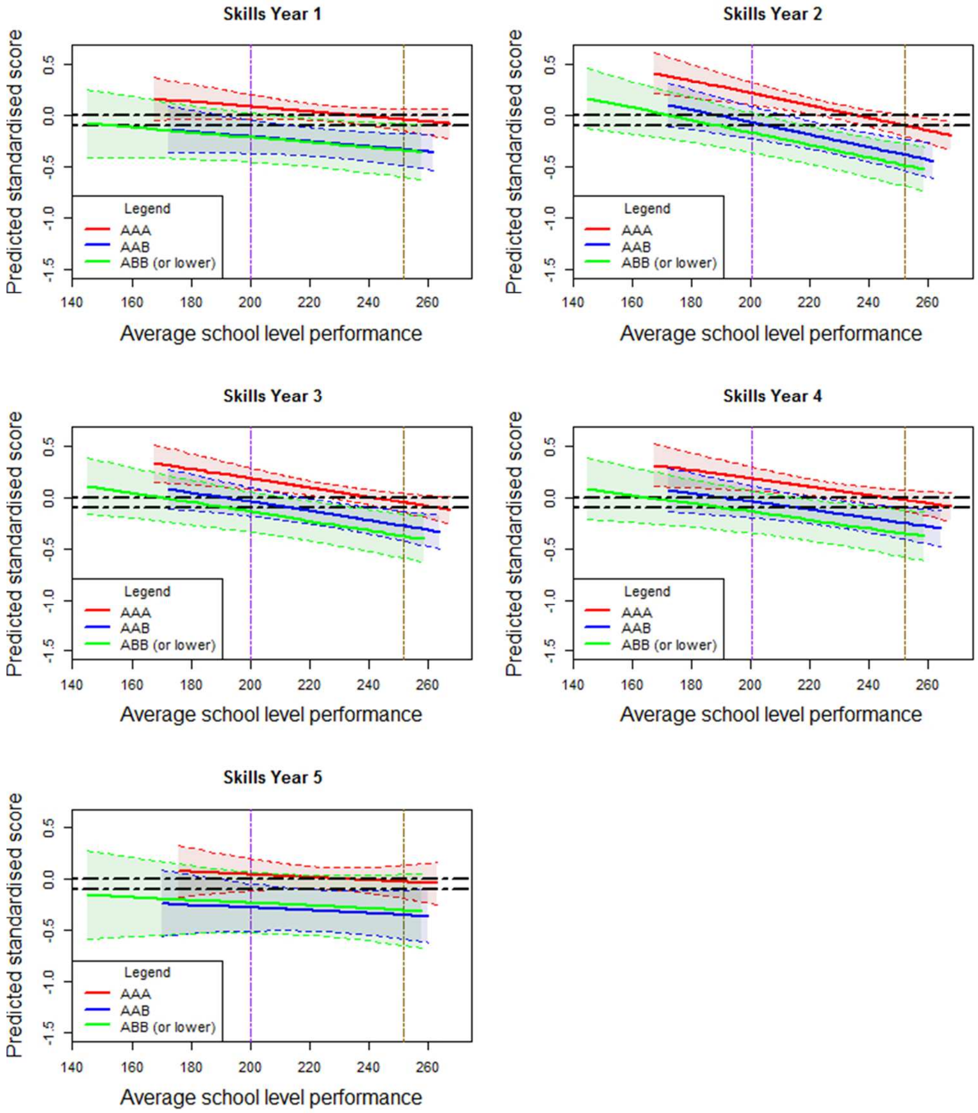

Figure 5: Effect of average school level performance by reported grades on undergraduate medical school skills based exams for all secondary schools in England in 2008. The 2nd and 8th deciles are denoted by the purple and brown vertical lines. The horizontal lines are arbitrary points chosen to demonstrate equivalent level of performance across the different grade levels.

Technical appendix to:

The impact of secondary (high) schooling on subsequent medical school performance: a national study

Contents

1 Estimation of Prior Education Attainment (PEA) 2

2 Mediation analyses 3
2.1 Single-level simple mediation analyses . . . 3
2.2 Multi-level simple mediation analyses . . . 5
2.3 Choosing between single-level and multi-level simple mediation analyses 10
2.4 Multi-level linear model . . . 14

3 Missing data 15

4 Sensitivity analysis for missing data 17
4.1 Single-level simple mediation analyses . . . 18
4.2 Multi-level linear model . . . 21

For peer review only - http://bmjopen.bmj.com/site/about/guidelines.xhtml

1 Estimation of Prior Education Attainment (PEA)

In order to obtain a single metric of scholastic (or academic) ability from the reported GCSEs and A Level exam scores, a novel approach described by *McManus et. al* [1] which involved conceptualising *'educational achievement'* as a latent variable was used. Thus PEA was estimated as a latent trait via an ordinal factor analysis using the most commonly taken A-level (both A1 and A2), and the grades obtained (e.g. A, B, C etc) used as (ordered categorical) indicators (see Table 1). The non-hierarchical version of McDonalds Omega was computed from the polychoric correlation matrix, since the factor analysis was of first order.[2, 3] The non-hierarchical McDonalds Omega was found to be 0.91. *Full Information Maximum Likelihood (FIML)* which maximizes use of the available data was used for the analysis to deal with missingness in the data (e.g. for the subjects not taken by a particular candidate). Subsequently, factor scores were then estimated for all applicants in the data, the results of the factor analysis from *Mplus* are displayed on Table 2. It was observed that generally, higher loadings were associated with Chemistry, Physics and Biology in GCSEs and A-Level (both A1 and A2) exams.

Exam	Subjects considered	Grade coding for factor analysis
GCSE	*Biology, Chemistry, Physics, Maths, French, History, Religious studies, Science, English, English literature* and *Geography*	C, D, E, F and G=1, B=2, A=3 and A^{*}=4
A Level (includes A1 and A2-level)	*Maths, Chemistry, Biology* and *Physics*	E and D=1 , C=2, B=3 and A=4

Table 1: *Coding of GCE A-Level and GCSE subjects for factor analysis.*

For peer review only - http://bmjopen.bmj.com/site/about/guidelines.xhtml

Exam	Subject	Loading	Std Error	Estimate / Std. Error	Two sided pvalue
GCSEs	Biology	0.805	0.009	93.988	0.000
	Chemistry	0.815	0.009	95.105	0.000
	English Literature	0.503	0.010	51.869	0.000
	English	0.572	0.009	62.060	0.000
	French	0.611	0.010	60.850	0.000
	Geography	0.696	0.011	60.710	0.000
	History	0.628	0.012	51.736	0.000
	Maths	0.693	0.008	90.998	0.000
	Physics	0.828	0.008	102.854	0.000
	Religious Education	0.510	0.012	43.155	0.000
	Science	0.749	0.049	15.233	0.000
A1-Level	Biology	0.861	0.005	171.013	0.000
	Chemistry	0.822	0.006	149.020	0.000
	Maths	0.798	0.009	93.642	0.000
	Physics	0.847	0.012	71.542	0.000
A2-Level	Biology	0.818	0.006	126.211	0.000
	Chemistry	0.798	0.007	121.959	0.000
	Maths	0.738	0.010	72.379	0.000
	Physics	0.836	0.010	86.867	0.000

Table 2: *Results from the factor analysis for the derivation of factor scores for PEA*

2 Mediation analyses

2.1 Single-level simple mediation analyses

Individuals who wish to study at a UKCAT consortium medical school sit the UKCAT test prior to making an application. The decision to make an offer, for those still at secondary school, is partly based on the predicted A-level (or equivalent) grades. This choice is commonly also informed by early achievement at the GCSE exams, usually taken earlier in the applicants school career. Therefore, any offers made would then be conditional on

For peer review only - http://bmjopen.bmj.com/site/about/guidelines.xhtml

For peer review only

the specified scores obtained first at the UKCAT test before the end of secondary school and later grades being achieved at A- level at end of the secondary school education (PEA which may include components of GCSEs) within each medical school selection cycle. It was thus aimed to *determine the extent to which an entrants PEA would mediate the predictive power of the UKCAT for two separate domains (knowledge and skills) over the period of undergraduate training"*. To accomplish this a mediation model was considered. This is because, the overall *total predictive power of the UKCAT* for *knowledge* and *skills* based undergraduate medical school exams would be partitioned into *direct* and *indirect* predictive power. This would then enable the accurate assessment of the relative, and unique, contribution UKCAT scores makes within the selection process. To demonstrate how this is done, consider Figure 1, which shows a *simple mediation model*. The term "simple" means that there is a one predictor, one mediator and one outcome variable under consideration.

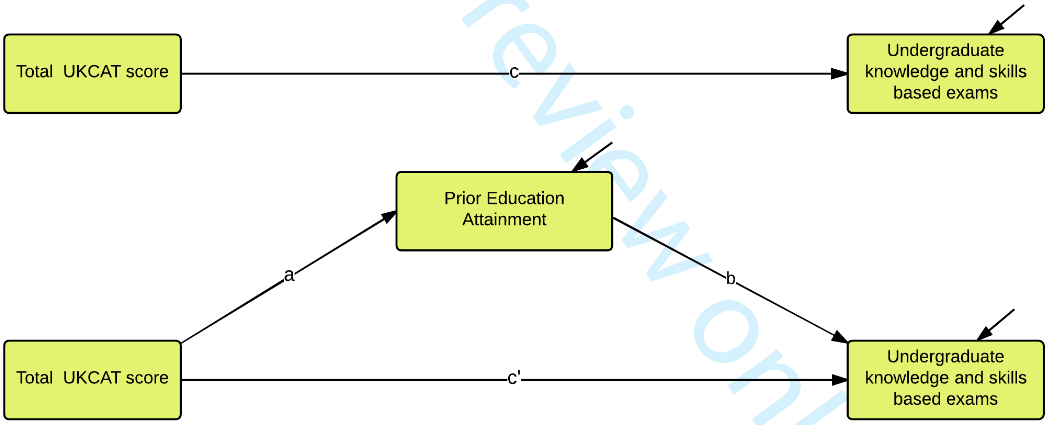

Figure 1: *Conceptual diagram of simple mediation model*

The effect denoted by *c* is the *total effect*, this may be easily obtained as a regression coefficient from a *Ordinary Least Squares (OLS)* regression model. The paths *b* and *c'* are *direct effects* for PEA and UKCAT respectively both of which may be obtained from a OLS regression model. For the purpose of the study, the paths of main interest will be the *indirect effect*, product of the paths *a*b*. This *indirect effect* represents the non-unique contribution of the predictive power of the UKCAT. Further, a proportion of this

For peer review only - http://bmjopen.bmj.com/site/about/guidelines.xhtml

non-unique contribution, which is the portion of the predictive power of the UKCAT that is explained by PEA, may be expressed as $\frac{a*b}{c}$ (see Figure 3 in text of main paper) where c is the *total effect* and has been shown to be equal to sum of the *indirect* and *direct effects*

$$c = a*b + c'$$

The significance of the *indirect effect* may be obtained by testing the hypothesis $H_0 : a*b = 0$ versus $H_0 : a*b \neq 0$, traditionally, this was done by assuming a normal distribution for the *indirect effect* of $a*b$ thus necessitating the use of wald, score or likelihood ratio test with their corresponding p-value. This however, may lead to incorrect conclusions, when the *indirect effect* is not normally distributed as is often the case.[4] For this reason, most statistical software packages, such as Mplus implement a hypothesis test using a bootstrap approach which yields an empirical distribution for $a*b$. Similarly, it is possible for one to program this in any statistical software (e.g. R) by implementing a bootstrap or Monte Carlo simulation. The idea being the derivation of $(1-\alpha)100$ bootstrap or Monte Carlo percentile confidence intervals for the purpose of determining signficance. For SAS and SPSS users, macros have been developed for estimating the significance of the *indirect effect*, they include the *INDIRECT* and *PROCESS* macros which are based on the bootstrap while *MCMED* macro is based on Monte Carlo Simulation.[5, 6]

2.2 Multi-level simple mediation analyses

The structure of the data used for the study was hierarchical (clustered) because the outcomes *knowledge* and *skills*) considered in each year of undergraduate training were nested within the 18 universities. This means that fitting a simple mediation analysis which essentially ignored the hierarchical structure of the data would potentially result in *total, direct* and *indirect effects* with induced attenuations which may then lead to biased conclusions. For this reason, a multi-level mediation model was considered. In a nutshell, this model constitutes fitting a simple mediation for each cluster (university) separately

For peer review only - http://bmjopen.bmj.com/site/about/guidelines.xhtml

and subsequently pooling the effects of interest together in some defined way to form *population average total, population average direct* and *population average indirect effects*. A conceptual representation of this model may be viewed on Figure 2.

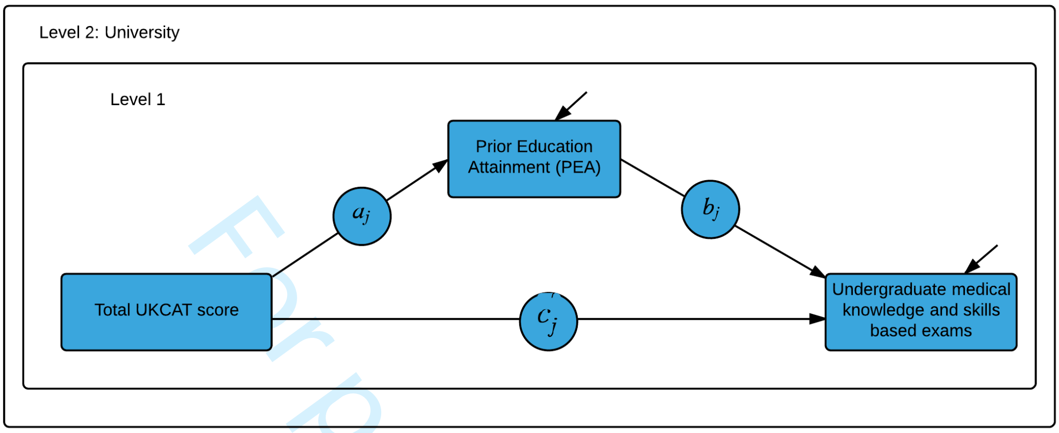

Figure 2: *Conceptual diagram of multi-level mediation model*

Note that, unlike in the case of simple (single-level) mediation in Figure 1, the effects are now level-1 variables nested within university which is a level-2 variable. Further, all the effects are estimated as random rather than fixed effects thus allowing them to vary between the level-2 variables. This model is called the 1→ 1→ 1 mediation model since the predictor, UKCAT, the mediator, PEA, and the outcomes, *knowledge* and *skills* based exams, all reside on level-1. In the conceptual representation of the model, the subscript j denotes that effects of interest vary between universities. These effects in the Figure are encircled to denote in *Structural Equation Modelling (SEM)* methodology that these effects are random.[7] The implementation of the 1→ 1→ 1 model is demonstrated for the *knowledge* based exam scores (denoted by K) for brevity. The UKCAT and PEA scores are denoted by UKCAT and PEA respectively.

$$PEA_{ij} = d_{PEA_j} + a_j * UKCAT_{ij} + \varepsilon_{PEA_{ij}} \tag{2.1}$$

$$K_{ij} = d_{K_j} + b_j * PEA_{ij} + c'_j * UKCAT_{ij} + \varepsilon_{K_{ij}} \tag{2.2}$$

For peer review only - http://bmjopen.bmj.com/site/about/guidelines.xhtml

$$d_{PEA_j} = d_{PEA} + \mu_{d_{PEA_j}}$$
$$d_{K_j} = d_K + \mu_{K_j}$$
$$a_j = a + \mu_{a_j}$$
$$b_j = b + \mu_{b_j}$$
$$c'_j = c' + \mu_{c'_j} \tag{2.3}$$

The subscript i denotes a student and subscript j a particular university. Further, $\varepsilon_{PEA_{ij}}$ and $\varepsilon_{K_{ij}}$ being level-1 residuals for the mediator PEA and Knowledge based outcome of interest respectively. Finally, d_{PEA_j}, d_{K_j},a_j,b_j and c'_j are the random intercepts and slopes of the models. The assumptions of the $1 \rightarrow 1 \rightarrow 1$ hierarchical mediation model are as follows

1. The predictor, $UKCAT_{ij}$ is uncorrelated with all the random effects (d_{PEA_j}, d_{K_j},a_j, b_j and c'_j) and the residuals ($\varepsilon_{PEA_{ij}}$ and $\varepsilon_{K_{ij}}$) in the model.
2. The residuals from the models, $\varepsilon_{PEA_{ij}}$ and $\varepsilon_{K_{ij}}$, are each normally distributed with an expected value of zero and are uncorrelated with one another.
3. The level-1 residuals, $\varepsilon_{PEA_{ij}}$ and $\varepsilon_{K_{ij}}$ are uncorrelated with random effects d_{PEA_j}, d_{K_j}, a_j, b_j and c'_j in the model.
4. The random effects are normally distributed with means equal to the average effects in the population. This may be expressed as,

   $$E(a_j) = \bar{a_j} = a$$

   $$E(b_j) = \bar{b_j} = b$$

   and

   $$E(c'_j) = \bar{c'_j} = c'$$

   for the slopes of interest. Further, the random effects covary with one another.
5. The distributions of PEA_{ij} is normal conditional on $UKCAT_{ij}$ and K_{ij} normal condi-

For peer review only - http://bmjopen.bmj.com/site/about/guidelines.xhtml

tional on PEA_{ij} and $UKCAT_{ij}$.

These assumptions lead to the following matrix formulation of the model. Note that, It is possible to estimate the *average of effects* (which may be referred to as *"population level effects"*, quantify the effects across all universities and their corresponding variabilities)

$$\begin{bmatrix} d_{PEA_j} \\ d_{K_j} \\ a_j \\ b_j \\ c'_j \end{bmatrix} \sim N\left(\begin{bmatrix} d_{PEA} \\ d_K \\ a \\ b \\ c' \end{bmatrix}, \begin{bmatrix} \sigma^2_{d_{PEA_j}} & & & & \\ \sigma_{d_{PEA_j K_j}} & \sigma^2_{K_j} & & & \\ \sigma_{d_{PEA_j a_j}} & \sigma_{K_j a_j} & \sigma^2_{a_j} & & \\ \sigma_{d_{PEA_j b_j}} & \sigma_{K_j b_j} & \sigma_{a_j b_j} & \sigma^2_{b_j} & \\ \sigma_{d_{PEA_j c'_j}} & \sigma_{K_j c'_j} & \sigma_{a_j c'_j} & \sigma_{b_j c'_j} & \sigma'_{c_j} \end{bmatrix}\right)$$

The *average mediation (indirect) effect* and *average total* effects may then be estimated by making use of equations 2.4 and 2.5 respectively.

$$E(a_j * b_j) = a * b + \sigma_{a_j b_j} \tag{2.4}$$

$$E(a_j * b_j + c') = a * b + \sigma_{a_j b_j} + c' \tag{2.5}$$

The multi-level simple mediation model was fitted in Mplus and the estimates of *average total, average indirect* and *average direct effects* estimated from equations 2.4 and 2.5. The significance of the *average total* and *average direct effects* were obtained from the results in Mplus. To determine the significance of *average indirect effect*, a Monte Carlo 95% Percentile CI was programmed in R software by sampling 10,000 observations from the distribution in equation 2.6.

$$N\left(\begin{bmatrix} a \\ b \\ \sigma_{a_j b_j} \end{bmatrix}, \begin{bmatrix} \sigma^2_a & \sigma_{ab} & \sigma_{a,\sigma_{a_j b_j}} \\ & \sigma^2_b & \sigma_{b,\sigma_{a_j b_j}} \\ & & \sigma^2_{\sigma_{a_j b_j}} \end{bmatrix}\right) \tag{2.6}$$

The individual elements of the distribution in equation 2.6 were obtained from the results of the multi-level mediation model in Mplus using the *TECH 3* output command. Each of

For peer review only - http://bmjopen.bmj.com/site/about/guidelines.xhtml

the 10,000 observations sampled for a, b and $\sigma_{a_jb_j}$ were plugged into equations 2.4 and 2.5 to obtain 10,000 *average indirect effect* values. Subsequently, the Monte Carlo 95% Percentile CI was calculated by taking the 2.5^{th} and 97.5^{th} percentile of the empirical distribution of the 10,000 estimates for *indirect effect*. Figures 3 and 4 shows the plotted results from the models.

It was observed that there were statistical significant *average indirect effects* in the first four years of undergraduate training of medical school for both *knowledge* and *skills* based exams outcomes. The *indirect effects* represent the contribution of PEA towards the predictive power of the UKCAT. It was also observed that the range of the CIs widened in the third year onwards which is indicative of the missingness observed in the later years of the study (see Figure 1 and Table 1 in main text of the paper) which led to little information available for analysis in each of the university clusters in the data.

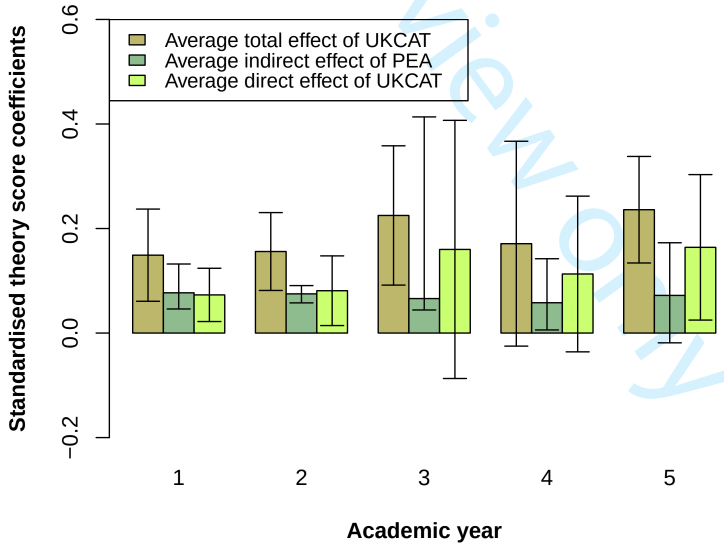

Figure 3: *Knowledge based multi-level mediation results for the average total, average direct and average indirect effects with respective 95% CI for average total and average direct effects computed from point estimates and standard errors obtained in Mplus and 95% Monte Carlo CI computed in R through simulation*

For peer review only - http://bmjopen.bmj.com/site/about/guidelines.xhtml

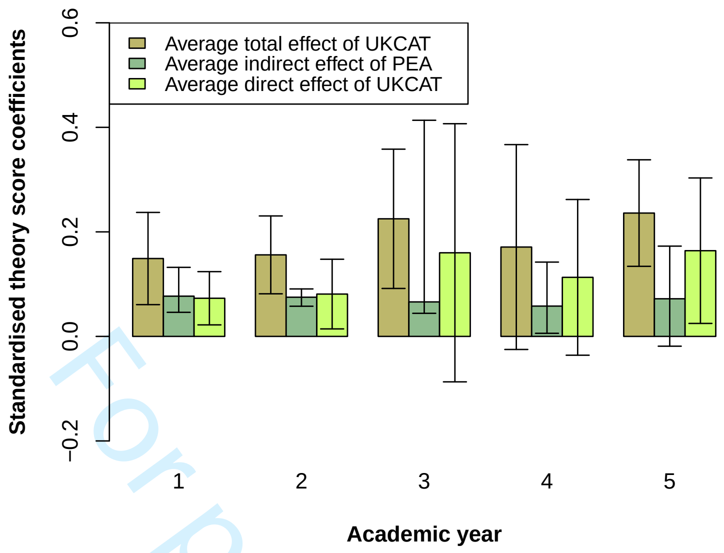

Figure 4: *Skills based multi-level mediation results for the average total, average direct and average indirect effects with respective 95% CI for average total and average direct effects computed from point estimates and standard errors obtained in Mplus and 95% Monte Carlo CI computed in R through simulation*

2.3 Choosing between single-level and multi-level simple mediation analyses

The multi-level mediation model fitted in section 2.2 is prone to convergence difficulties and is highly susceptible to missing data related problems. For instances where there are high attrition rates in later years of a longitudinal cohort study, it is highly likely that some or most of the clusters may have little or no data to contribute meaningfully to the analysis and this may further risk a lack of convergence. Therefore, for a given estimation problem, a single-level mediation model is preferred if there is evidence that there are no statistically significant clustering effects in the data. To determine whether there were statistically significant clustering effects in the data equations 2.4 and 2.5 were considered.

Note that from equation 2.4, when $\sigma_{a_j b_j} = 0$, the resulting *average indirect effect* is equal to what would be estimated in a single-level simple mediation analysis in section 2.1. Therefore in seeking to determine whether a single or multi-level mediation analysis should be fitted to the data, it will be sufficient to test the hypothesis, $H_0 : \sigma_{a_j b_j} = 0$

For peer review only - http://bmjopen.bmj.com/site/about/guidelines.xhtml

versus $H_1 : \sigma_{a_j b_j} \neq 0$. Evidence in favour of the null hypothesis would also be evidence in favour of a simple single-level mediation analysis. The results of the hypothesis test were available as part of the multi-level results in Mplus and are displayed on Table 3. It was observed that all of the p-values were > 0.05 implying that there were statistically non-significant clustering effects in the data. Further, *Intra Cluster Correlations (ICCs)* for the models computed by utilising the main diagonal of the covariance matrix from equation 2.6 and the residual variances from the model are displayed on Table 4. The observed ICCs (7th and 13th column of the Table) indicate that the proportion of variability explained by the multi-level mediation models is negligible. Therefore a simple single-level mediation model is appropriate for the data.

Academic year	Knowledge based exams			Skills based exams		
	$\sigma_{a_j b_j}$	Std. Error	Pvalue	$\sigma_{a_j b_j}$	Std. Error	Pvalue
1	-0.007	0.016	0.663	-0.006	0.007	0.414
2	-0.004	0.003	0.284	-0.005	0.012	0.673
3	-0.002	0.066	0.972	0.000	0.003	0.888
4	-0.001	0.006	0.872	-0.004	0.013	0.778
5	0.000	0.031	0.992	-0.001	-0.009	0.951

Table 3: *Results of the hypothesis testing for the statistical significance of $\sigma_{a_j b_j}$ from Mplus*

.

Academic year	Knowledge based exams						Skills based exams					
	σ_a^2	σ_b^2	σ_{res}^2	σ_{PEA}^2	$\sigma_{\sigma_{a_j b_j}}^2$	$\frac{\sigma_{\sigma_{a_j b_j}}^2}{(\sigma_a^2+\sigma_b^2+\sigma_{res}^2+\sigma_{PEA}^2+\sigma_{\sigma_{a_j b_j}}^2)}$	σ_a^2	σ_b^2	σ_{res}^2	σ_{PEA}^2	$\sigma_{\sigma_{a_j b_j}}^2$	$\frac{\sigma_{\sigma_{a_j b_j}}^2}{(\sigma_a^2+\sigma_b^2+\sigma_{res}^2+\sigma_{PEA}^2+\sigma_{\sigma_{a_j b_j}}^2)}$
1	0.006	0.003	0.848	1.876	0.000	0.000	0.003	0.001	0.888	1.877	0.000	0.000
2	0.002	0.000	0.894	1.875	0.000	0.000	0.011	0.001	0.968	1.876	0.000	0.000
3	0.093	0.034	0.841	1.875	0.004	0.002	0.003	0.001	0.947	1.876	0.000	0.000
4	0.005	0.006	0.890	1.875	0.000	0.000	0.012	0.000	0.893	1.876	0.000	0.000
5	0.004	0.002	0.902	1.876	0.001	0.000	0.003	0.006	0.985	1.877	0.000	0.000

Table 4: *Intra Cluster Correlations (ICC) for Knowledge and Skills based exam outcomes for the five years of undergraduate medical school training*

.

Following the results on Tables 3 and 4, a simple single-level mediation model was fitted using two models, for the case of *knowledge* based exams outcomes, the two models are

For peer review only - http://bmjopen.bmj.com/site/about/guidelines.xhtml

expressed as equation (2.8) and (2.7) using the same notation as in section 2.2.

$$PEA_i = I_{PEA} + a * UKCAT_i + \varepsilon_{PEA} \quad (2.7)$$

$$K_i = I_K + c^{'} * UKCAT_i + b * PEA_i + \varepsilon_K \quad (2.8)$$

The mediator of interest, PEA, is denoted by *PEA* while *UKCAT* and *K* are predictor and outcome of interest respectively. The *I* denotes the intercept while *a,b* and *c* are the regression coefficients to be estimated. This model was fitted both in Mplus and in SAS. The results of the models from the two software packages were similar as expected. The statistical significance was tested using the bootstrap approach implemented in Mplus and Monte Carlo simulation in SAS using the *MCMED macro for SAS*.[5] In both Mplus and SAS, the 95 % confidence intervals were obtained by taking the 2.5^{th} and 97.5^{th} percentiles of the empirical distribution for $a * b$ from 10,000 sampled observations. The results of the model for both Mplus and SAS can be found on Table 5 and 6 for *knowledge* based exams while Table 7 and 8 show the single-level simple mediation model results for *skills* based exams. For both *knowledge* and *skills* based outcomes, in all undergraduate years, there were statistically significant *indirect effects* of UKCAT through PEA. This means that the predictive power of the UKCAT for undergraduate medical school performance can be partially explained by PEA.

	Knowledge based exams (Mplus)					
	Direct effect		Indirect effect		Total effect	
Academic year	Estimate	95% CI	Estimate	95% Bootstrap CI	Estimate	95% CI
1	0.071	(0.060, 0.136)	0.086	(0.068, 0.110)	0.156	(0.092, 0.220)
2	0.073	(0.040, 0.140)	0.080	(0.059, 0.104)	0.152	(0.085, 0.217)
3	0.127	(0.055, 0.202)	0.071	(0.050, 0.094)	0.198	(0.128, 0.271)
4	0.086	(0.012, 0.162)	0.061	(0.041, 0.083)	0.147	(0.075, 0.220)
5	0.162	(0.055, 0.268)	0.076	(0.045, 0.110)	0.238	(0.130, 0.343)

Table 5: *Results of the single-level simple mediation model for knowledge based exams from Mplus*

.

For peer review only - http://bmjopen.bmj.com/site/about/guidelines.xhtml

Academic year	Knowledge based exams (SAS)					
	Direct effect		Indirect effect		Total effect	
	Estimate	95% CI	Estimate	95% Monte Carlo CI	Estimate	95% CI
1	0.071	(0.002, 0.139)	0.081	(0.059, 0.106)	0.151	(0.083, 0.220)
2	0.073	(0.002, 0.144)	0.074	(0.053, 0.010)	0.147	(0.077 0.217)
3	0.127	(0.058, 0.195)	0.069	(0.049, 0.094)	0.196	(0.129, 0.263)
4	0.086	(0.014, 0.159)	0.062	(0.040, 0.085)	0.148	(0.078, 0.218)
5	0.162	(0.058, 0.266)	0.052	(0.027, 0.087)	0.213	(0.109, 0.318)

Table 6: *Results of the single-level simple mediation model for knowledge based exams from SAS*

.

Academic year	Skills based exams (Mplus)					
	Direct effect		Indirect effect		Total effect	
	Estimate	95% CI	Estimate	95% Bootstrap CI	Estimate	95% CI
1	0.056	(-0.028, 0.137)	0.055	(0.032, 0.079)	0.111	(0.032, 0.189)
2	0.032	(-0.044, 0.107)	0.056	(0.036, 0.078)	0.088	(0.014, 0.160)
3	0.048	(-0.024, 0.122)	0.032	(0.012, 0.054)	0.080	(0.010, 0.150)
4	0.062	(-0.015, 0.140)	0.032	(0.012, 0.054)	0.094	(0.021, 0.168)
5	0.121	(0.050, 0.237)	0.045	(0.012, 0.080)	0.165	(0.052,0.276)

Table 7: *Results of the single-level simple mediation model for skills based exams from Mplus*

.

Academic year	Skills based exams (SAS)					
	Direct effect		Indirect effect		Total effect	
	Estimate	95% CI	Estimate	95% Monte Carlo CI	Estimate	95% CI
1	0.056	(-0.028, 0.140)	0.045	(0.026, 0.070)	0.101	(0.019, 0.184)
2	0.032	(-0.049, 0.113)	0.059	(0.038, 0.085)	0.091	(0.012, 0.170)
3	0.048	(-0.026, 0.122)	0.031	(0.012, 0.052)	0.078	(0.007, 0.150)
4	0.062	(-0.017, 0.141)	0.032	(0.012, 0.055)	0.094	(0.017, 0.170)
5	0.121	(0.010, 0.232)	0.030	(0.009, 0.063)	0.151	(0.042, 0.261)

Table 8: *Results of the single-level simple mediation model for skills based exams from SAS*

.

For peer review only - http://bmjopen.bmj.com/site/about/guidelines.xhtml

2.4 Multi-level linear model

To address the second aim of the study, which was *"to appraise the influence of the performance of the previous secondary school attended on an undergraduates achievement in medical school"*, a multi-level linear model or *Linear Mixed Model (LMM)* was used due to its capability to handle clustering in instances where the outcomes are continuous and correlated. The term "mixed" in the Linear Mixed Model comes from the fact that the model estimates both fixed (mean structure) and random effects (random structure). The modelling framework of Linear Mixed Model may be expressed as follows:

$$Y_i = X_i\beta + Z_i b_i + \varepsilon_i$$

where

$$b_i \sim N(0, D)$$

$$\varepsilon_i \sim N(0, \Sigma_i)$$

with $b_1 \ldots b_N$ and $\varepsilon_1 \ldots \varepsilon_N$ being independent.

Y_i is the n_i-dimensional outcome (*knowledge* or *skills* based exams), X_i and Z_i are the design matrices for the fixed and random effects of known predictors respectively, β and b_i are fixed and university specific effects respectively, and ε_i is the vector containing the residual components.[8] X_i is a design matrix containing predictors, average school level performance of the school a candidate sat for the A-level, their reported grade (AAA, AAB, ABB, BBB or BBC) and interaction between average school level performance and reported A-level grades whereas Z_i is a design matrix containing a random intercept which modelled the correlation in the outcomes within a university by allowing the (predicted) outcomes to vary between universities.

For peer review only - http://bmjopen.bmj.com/site/about/guidelines.xhtml

3 Missing data

From Table 9, it was observed that only 20.84% of the entrants had complete data for the *knowledge* based exam outcome throughout the five years of medical school. Monotone missingness accounted for 47.36% of the missingness pattern for the *knowledge* based exam outcome with the most frequently occurring monotone missingness pattern having outcome data for only year one to year three. On the other hand, the most frequent arbitrary (non-monotone) missingness pattern had outcome data missing for year one, two and five.

Table 10 shows the missingness pattern for *skills* based exam outcome. About 17% of the entrants had complete data for the outcome over the course of the study duration while 41.29% of the data had monotone missingness with the most occurring missingness monotone pattern having outcome data missing for year five. The most occurring arbitrary missingness pattern compromising of about 9.5% of the arbitrary missingess pattern was for year one, two and five.

For peer review only

For peer review only - http://bmjopen.bmj.com/site/about/guidelines.xhtml

Outcome						Count	%
Group	Year 1	Year 2	Year 3	Year 4	Year 5		
Complete							
1	O	O	O	O	O	439	20.84
Monotone missingness							
2	O	O	O	O	M	272	12.91
3	O	O	O	M	M	330	15.66
4	O	O	M	M	M	199	9.44
5	O	M	M	M	M	42	1.99
6	M	M	M	M	M	155	7.36
Arbitrary missingness							
7	O	M	O	M	M	7	0.33
8	O	O	M	O	O	50	2.37
9	O	O	M	O	M	114	5.41
10	M	O	O	O	M	7	0.33
11	O	M	O	M	M	3	0.14
12	M	O	M	M	M	4	0.19
13	M	M	O	O	M	274	13
14	M	M	O	M	M	16	0.76
15	M	M	M	O	O	135	6.41
16	M	M	M	O	M	58	2.75
17	M	M	M	M	O	2	0.09
Total						2,107	100

Table 9: *Missingness patterns for knowledge scores for the 2,107 entrants from 2007 UKCAT cohort. Each 'O' and 'M' represents each instance where data are present and absent respectively (i.e. the first row represents the proportion of cases with no missing data). Patterns are categorised as either monotone (i.e. where data relating to all subsequent years are missing after the initial missing data year) or arbitrary (i.e. non-monotone).*

For peer review only

Outcome						Count	%
Group	Year 1	Year 2	Year 3	Year 4	Year 5		
Complete							
1	O	O	O	O	O	360	17.09
Monotone missingness							
2	O	O	O	O	M	308	14.62
3	O	O	O	M	M	61	2.9
4	O	O	M	M	M	199	9.44
5	O	M	M	M	M	26	1.23
6	M	M	M	M	M	276	13.1
Arbitrary missingness							
7	O	O	M	O	M	91	4.32
8	O	M	O	M	M	6	0.28
9	M	O	O	O	M	37	1.76
10	O	M	M	O	O	37	1.76
11	M	O	M	M	M	140	6.64
12	M	M	O	O	O	79	3.75
13	M	M	O	O	M	197	9.35
14	M	M	O	M	M	153	7.26
15	M	M	M	M	O	137	6.5
Total						2,107	100

Table 10: *Missingness patterns for skills scores for the 2,107 entrants from 2007 UKCAT cohort. Each 'O' and 'M' represents each instance where data are present and absent respectively (i.e. the first row represents the proportion of cases with no missing data). Patterns are categorised as either monotone (i.e. where data relating to all subsequent years are missing after the initial missing data year) or arbitrary (i.e. non-monotone).*

4 Sensitivity analysis for missing data

Sensitivity analysis was conducted to determine to what extent the missingness in the data influenced the results of the study. The data analysis for the study assumed *Missing At Random (MAR)* mechanism. The MAR assumption was invoked by making use of *ignorability* which entailed ignoring the missingness process. The sensitivity analysis investigated whether this assumption was justifiable. This involved refitting the models with multiply imputed data and comparing the results from these models those with those fitted previously under ingnorability. The premise being, if ignorability is valid under MAR, and *Multiple Imputation (MI)* which is also valid under MAR, then the results under both should be similar. When this is the case, the assumption of ignorability and

For peer review only - http://bmjopen.bmj.com/site/about/guidelines.xhtml

MAR would be justified.

4.1 Single-level simple mediation analyses

For the single-level simple mediation analysis, the models were fitted after imputation was conducted 30 times thus creating 30 datasets whose analysis yielded results which were later summarised through pooling of the estimates and computation of associated standard errors of their estimates. The MI was conducted in SAS using the *Monte Carlo Markov Chain (MCMC)* which imputes the missing values in the data with plausible ones that retain the overall mean and covariate structure of the data assuming a joint multivariate normal distribution.[9, 10] The results of the previous non-imputed data displayed on Table 6 and 8 for both *knowledge* and *skills* based exams and are further displayed in graphical form on Figure 5. These were compared to the results from the multiply imputed data which are found on Figure 6. It was observed that in as far as the aim of the analysis was concerned, there were no discernible difference in the estimates and conclusions regarding the *indirect effects* of UKCAT through PEA for both the *knowledge* and *skills* based outcomes from both the multiply imputed and non-imputed data. This implies that the assumptions of ignorability and MAR were plausible and that the missingness though severe in later years of the study, did not result in adverse effects on the results and conclusions of the statistical analysis. This is in keeping with the missing data mainly related to whether a particular medical school submitted data to the UKCAT database for that particular year. Thus, we conclude the missing data was unlikely to threaten the validity of the inferences drawn from our results.

For peer review only

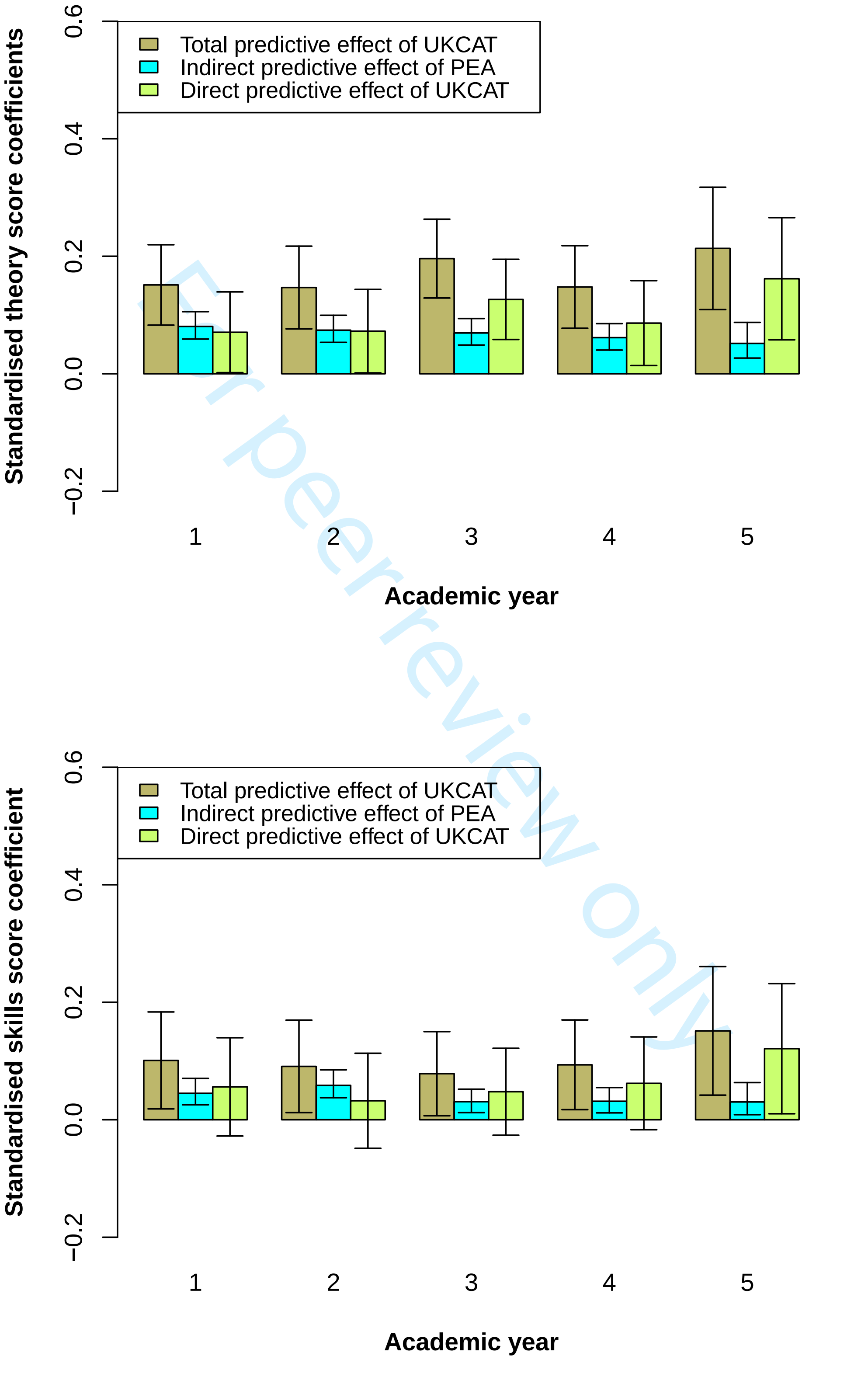

Figure 5: *Results of the single-level simple mediation analysis based on non-imputed data*

For peer review only - http://bmjopen.bmj.com/site/about/guidelines.xhtml

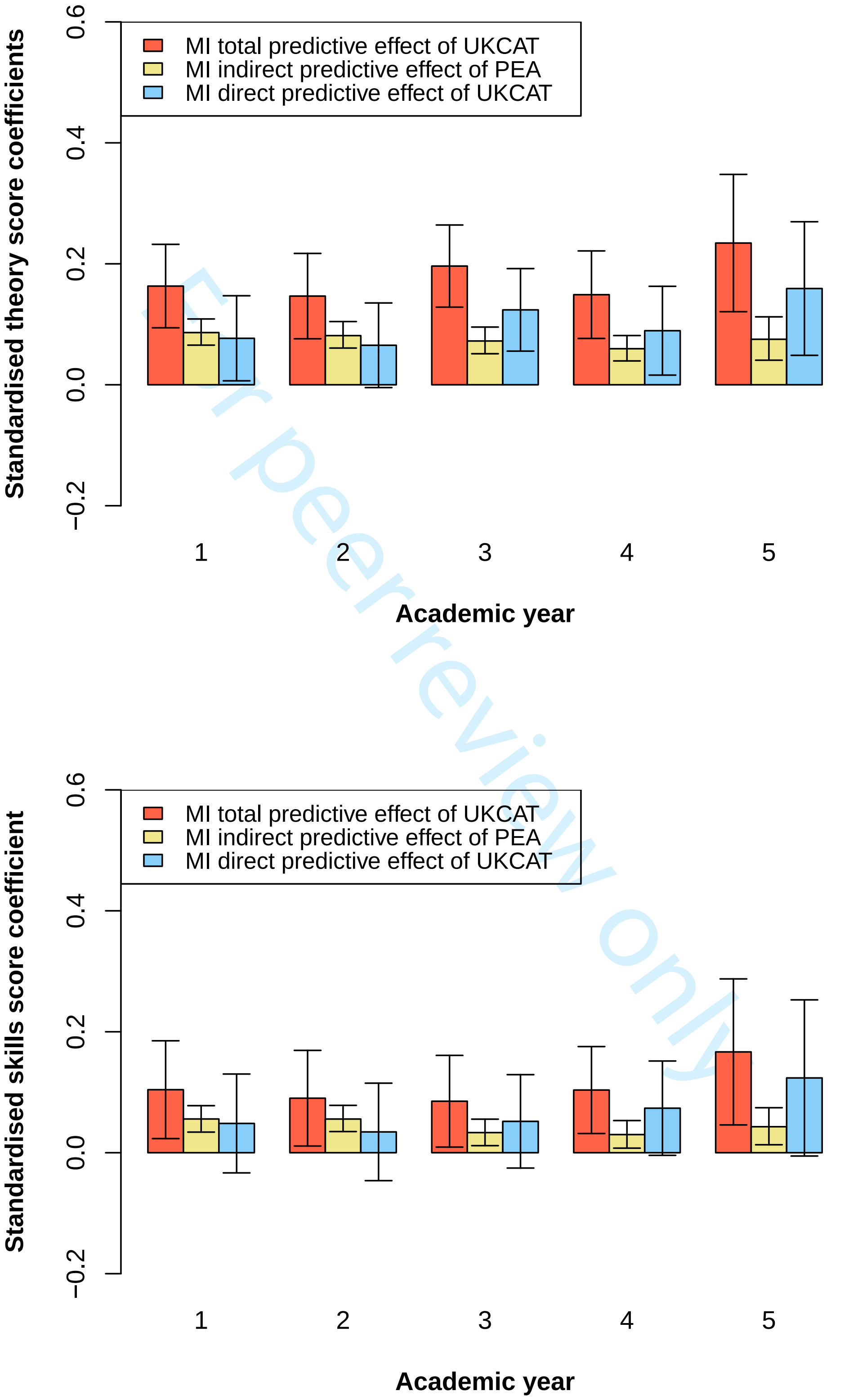

Figure 6: *Results of the single-level simple mediation analysis based on 30 MI data*

For peer review only - http://bmjopen.bmj.com/site/about/guidelines.xhtml

4.2 Multi-level linear model

Figure 7 and 8 show the plots of MI results for the model investigating the effect of average school level performance by reported grades on *knowledge* and *skills* based exam outcomes for all five years of undergraduate medical school. All the variables of interest, that is, *knowledge* and *skills* based undergraduate medical exam outcomes, average school level performance and PEA grades were affected by missingness. MI was conducted using *Multiple Imputations by Chain Equations (MICE), a MCMC based imputation technique that makes use of a collection of univariate conditional distributions of the variables with missing values given the other variables present in the data.*[9] The number of imputations , M, was initially set at 5 and increased by multiples of 5 until a value of M that would yield unchanging results for the model described in section 2.4. The parameter estimates obtained were the same for M >=10 indicating that any choice of M>=10 was optimal. For comparison with results from the original data, M=15 was used. A comparison of all the results MI with those from the original data shown in Figures 4 and 5 in the main text of the paper for both *knowledge* and *skills* based exams outcomes show that largely the missingness did not have an adverse effect on the analysis. Like in the original unimputed data, for both *knowledge* and *skills* based exam outcomes, at each level of average school level performance students with higher grades tend to perform better compared to their counterparts with lower grades throughout undergraduate medical school. Overall, compared to students from schools with high average school level performance, students from schools with low average school level performance tend to have better scores in both *knowledge* and *skills* based exam outcomes throughout undergraduate medical school. This suggests that the assumption of MAR invoked for the study was plausible.

For peer review only - http://bmjopen.bmj.com/site/about/guidelines.xhtml

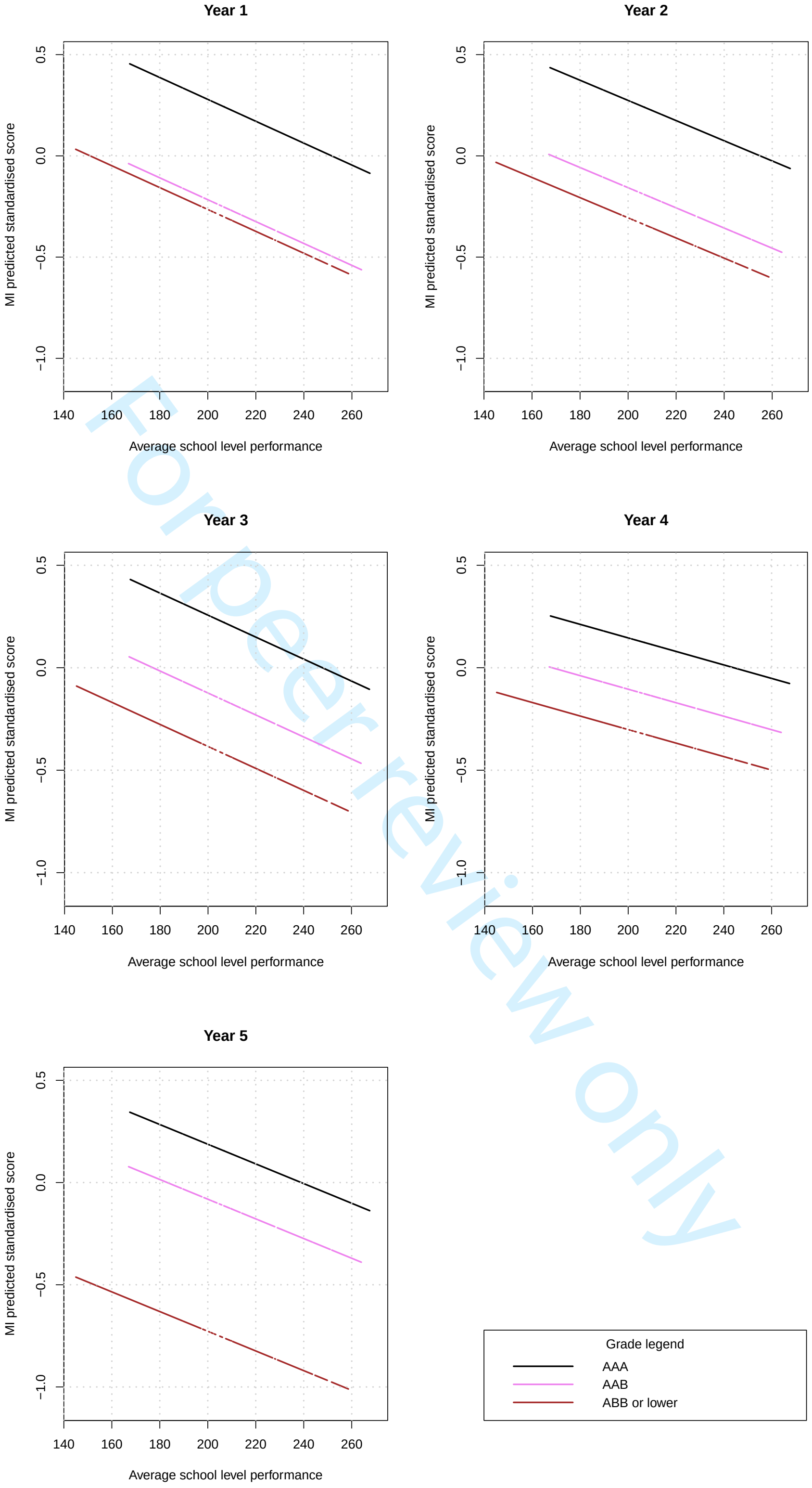

Figure 7: *Multiply imputed effect of average school level performance by reported grades on undergraduate medical school knowledge based exams.*

For peer review only - http://bmjopen.bmj.com/site/about/guidelines.xhtml

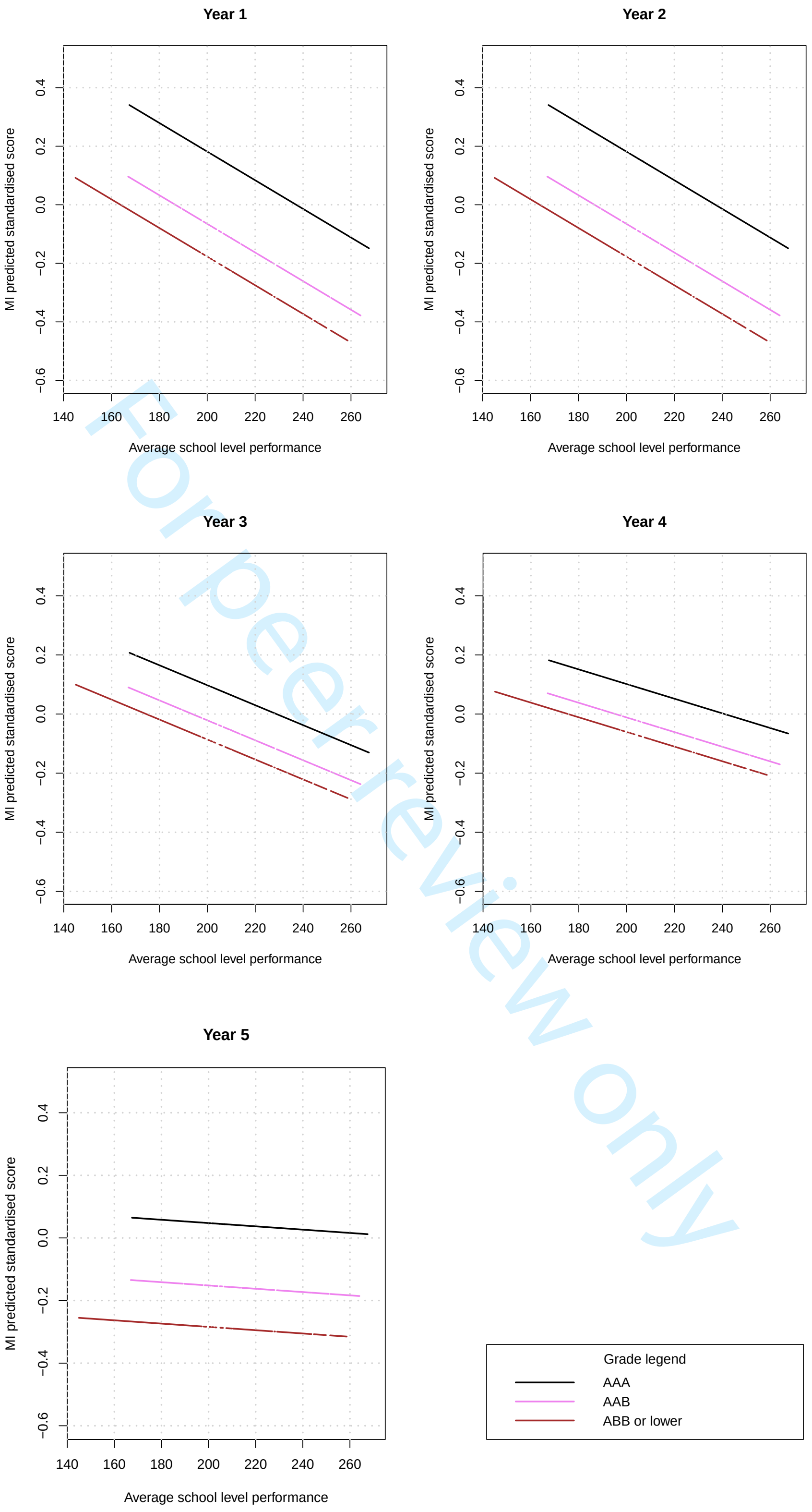

Figure 8: *Multiply imputed effect of average school level performance by reported grades on undergraduate medical school skills based exams.*

For peer review only - http://bmjopen.bmj.com/site/about/guidelines.xhtml

References

[1] IC McManus et al. “The UKCAT-12 study: educational attainment, aptitude test performance, demographic and socio-economic contextual factors as predictors of first year outcome in a cross-sectional collaborative study of 12 UK medical schools”. In: *BMC medicine* 11.1 (2013), p. 244.

[2] William Revelle and Richard E Zinbarg. “Coefficients alpha, beta, omega, and the glb: Comments on Sijtsma”. In: *Psychometrika* 74.1 (2009), pp. 145–154.

[3] Tenko Raykov. “Estimation of composite reliability for congeneric measures”. In: *Applied Psychological Measurement* 21.2 (1997), pp. 173–184.

[4] Bengt O Muthén Linda K Muthén Tihomir Asparouhov. *Regression And Mediation Analysis Using Mplus*. Muthén & Muthén, 2016, pp. 61–62.

[5] Andrew F Hayes. *My Macros and Code for SPSS and SAS*. URL: http://afhayes.com/spss-sas-and-mplus-macros-and-code.html (visited on 01/26/2017).

[6] Andrew F. Hayes. *The PROCESS macro for SPSS and SAS*. URL: http://www.processmacro.org/index.html (visited on 07/12/2017).

[7] Daniel J Bauer, Kristopher J Preacher, and Karen M Gil. “Conceptualizing and testing random indirect effects and moderated mediation in multilevel models: new procedures and recommendations.” In: *Psychological methods* 11.2 (2006), p. 142.

[8] Geert Verbeke and Geert Molenberghs. *Linear mixed models for longitudinal data*. Springer Science & Business Media, 2009.

[9] Geert Molenberghs and Michael Kenward G. *Missing Data in Clinical Trials*. John Wiley & Sons Ltd, 2007. Chap. Multiple Imputation, pp. 110–114.

[10] Geert Molenberghs and Geert Verbeke. *Models for Discrete Longitudinal Data*. Springer Science & Business Media, 2005. Chap. Multiple Imputation and Expectation-Maximazation Algorithm, pp. 515–516.

For peer review only - http://bmjopen.bmj.com/site/about/guidelines.xhtml

STROBE 2007 (v4) Statement—Checklist of items that should be included in reports of *cohort studies*

Section/Topic	Item #	Recommendation	Reported on page #
**Title and abstract**	1	(*a*) Indicate the study's design with a commonly used term in the title or the abstract	1
		(*b*) Provide in the abstract an informative and balanced summary of what was done and what was found	1-3
**Introduction**			
Background/rationale	2	Explain the scientific background and rationale for the investigation being reported	3-8
Objectives	3	State specific objectives, including any prespecified hypotheses	7
**Methods**			
Study design	4	Present key elements of study design early in the paper	6-7
Setting	5	Describe the setting, locations, and relevant dates, including periods of recruitment, exposure, follow-up, and data collection	8-9
Participants	6	(*a*) Give the eligibility criteria, and the sources and methods of selection of participants. Describe methods of follow-up	8-9
		(*b*) For matched studies, give matching criteria and number of exposed and unexposed	N/A
Variables	7	Clearly define all outcomes, exposures, predictors, potential confounders, and effect modifiers. Give diagnostic criteria, if applicable	8-12
Data sources/ measurement	8*	For each variable of interest, give sources of data and details of methods of assessment (measurement). Describe comparability of assessment methods if there is more than one group	*8-12*
Bias	9	Describe any efforts to address potential sources of bias	22-23
Study size	10	Explain how the study size was arrived at	8-9
Quantitative variables	11	Explain how quantitative variables were handled in the analyses. If applicable, describe which groupings were chosen and why	13-14
Statistical methods	12	(*a*) Describe all statistical methods, including those used to control for confounding	13-14
		(*b*) Describe any methods used to examine subgroups and interactions	N/A
		(*c*) Explain how missing data were addressed	22-23
		(*d*) If applicable, explain how loss to follow-up was addressed	22-23
		(*e*) Describe any sensitivity analyses	22-23
**Results**			

For peer review only

Participants	13*	(a) Report numbers of individuals at each stage of study—eg numbers potentially eligible, examined for eligibility, confirmed eligible, included in the study, completing follow-up, and analysed	8-9
		(b) Give reasons for non-participation at each stage	8-9
		(c) Consider use of a flow diagram	8-9
Descriptive data	14*	(a) Give characteristics of study participants (eg demographic, clinical, social) and information on exposures and potential confounders	14-15
		(b) Indicate number of participants with missing data for each variable of interest	16-17 in Supplementary document
		(c) Summarise follow-up time (eg, average and total amount)	8
Outcome data	15*	Report numbers of outcome events or summary measures over time	8-9
Main results	16	(*a*) Give unadjusted estimates and, if applicable, confounder-adjusted estimates and their precision (eg, 95% confidence interval). Make clear which confounders were adjusted for and why they were included	16-19
		(*b*) Report category boundaries when continuous variables were categorized	N/A
		(*c*) If relevant, consider translating estimates of relative risk into absolute risk for a meaningful time period	N/A
Other analyses	17	Report other analyses done—eg analyses of subgroups and interactions, and sensitivity analyses	22
**Discussion**			
Key results	18	Summarise key results with reference to study objectives	19-21
**Limitations**			
Interpretation	20	Give a cautious overall interpretation of results considering objectives, limitations, multiplicity of analyses, results from similar studies, and other relevant evidence	21-22
Generalisability	21	Discuss the generalisability (external validity) of the study results	25
**Other information**			
Funding	22	Give the source of funding and the role of the funders for the present study and, if applicable, for the original study on which the present article is based	26-27

*Give information separately for cases and controls in case-control studies and, if applicable, for exposed and unexposed groups in cohort and cross-sectional studies.

Note: An Explanation and Elaboration article discusses each checklist item and gives methodological background and published examples of transparent reporting. The STROBE checklist is best used in conjunction with this article (freely available on the Web sites of PLoS Medicine at http://www.plosmedicine.org/, Annals of Internal Medicine at http://www.annals.org/, and Epidemiology at http://www.epidem.com/). Information on the STROBE Initiative is available at www.strobe-statement.org.

For peer review only

BMJ Open

What is the effect of secondary (high) schooling on subsequent medical school performance? A national, UK-based, cohort study

Journal:	*BMJ Open*
Manuscript ID	bmjopen-2017-020291.R1
Article Type:	Research
Date Submitted by the Author:	14-Feb-2018
Complete List of Authors:	Mwandigha, Lazaro; University of York, Health Sciences<br>Tiffin, Paul; University of York, Health Sciences<br>Paton, Lewis ; University of York, Health Sciences<br>Kasim, Adetayo; Durham University, Wolfson Research Institute for Health and Wellbeing<br>Boehnke, Jan Rasmus; University of Dundee, School of Nursing and Health Sciences
**Primary Subject Heading**:	Medical education and training
Secondary Subject Heading:	Research methods
Keywords:	MEDICAL EDUCATION & TRAINING, STATISTICS & RESEARCH METHODS, EDUCATION & TRAINING (see Medical Education & Training)

SCHOLARONE™
Manuscripts

only

What is the effect of secondary (high) schooling on subsequent medical school performance? A national, UK-based, cohort study

Lazaro M. Mwandigha, Paul A. Tiffin, Lewis W. Paton, Adetayo S. Kasim, Jan R. Böhnke

Author Details

LMM: Postgraduate student, Department of Health Sciences, University of York, Heslington, York, YO10 5DD, UK, email; lmm544@york.ac.uk

PAT (Corresponding author): Reader (Associate Professor), Department of Health Sciences, University of York, Heslington, York, YO10 5DD, UK, Tel: 01904 321117, email; paul.tiffin@york.ac.uk

LWP: Research Fellow, Department of Health Sciences, University of York, Heslington, York, YO10 5DD, UK, email; lewis.paton@york.ac.uk

ASK: Senior Research Statistician, Wolfson Research Institute of Health and Wellbeing, Durham University, Queen's Campus, Stockton on Tees, TS17 6BH, UK, email; a.s.kasim@durham.ac.uk

JRB: Senior Research Fellow, Dundee Centre for Health and Related Research, School of Nursing and Health Sciences, University of Dundee, Dundee, DD1 4HJ, UK, AND Department of Health Sciences, University of York, Heslington, York YO10 5DD; email; j.r.boehnke@dundee.ac.uk

For peer review only

For peer review only - http://bmjopen.bmj.com/site/about/guidelines.xhtml

ABSTRACT

Objectives

University academic achievement may be inversely related to the performance of the secondary (high) school an entrant attended. Indeed, some medical schools already offer 'grade discounts' to applicants from less well performing schools. However, evidence to guide such policies is lacking. In this study we analyse a national dataset in order to understand the relationship between the two main predictors of medical school admission in the UK (prior educational achievement (PEA) and performance on the United Kingdom Clinical Aptitude Test (UKCAT)) and subsequent undergraduate knowledge and skills-related outcomes analysed separately. Specifically, we explore the potential mediating role played by a student's previous secondary school's performance in order to inform selection policy.

Methods

The study was based on national selection data and linked medical school outcomes for *knowledge* and *skills*-based test during the first five years of medical school. UKCAT scores and PEA grades were available for 2,107 students enrolled at 18 medical schools. A series of mediation and multi-level models were developed and tested.

Results

The ability of the UKCAT scores to predict undergraduate academic performance was significantly mediated by PEA in all five years of medical school. Undergraduate achievement was inversely related to secondary school-level performance. This

For peer review only - http://bmjopen.bmj.com/site/about/guidelines.xhtml

effect waned over time and was less marked for *skills*, compared to undergraduate *knowledge*-based outcomes. Thus, the predictive value of secondary school grades was generally dependent on the secondary school in which they were obtained.

Conclusions

The UKCAT scores added some value, above and beyond secondary school achievement, in predicting undergraduate performance, especially in the later years of study. Importantly the findings suggest that the academic entry criteria should be relaxed for candidates applying from the least well performing secondary schools. In the UK, this would translate into a decrease of approximately one to two A-level grades.

ARTICLE SUMMARY

Strengths of the study

- Schools and university data were able to be linked permitting the first UK-based study that compared the academic performance of medical students drawn from poorly performing secondary schools against their counterparts from well-performing ones across all the five years of medical school
- The sample was relatively large with a total of 2,107 medical school students who matriculated in 2008 included in this study

For peer review only - http://bmjopen.bmj.com/site/about/guidelines.xhtml

Limitations of the study

- The *skills* and *knowledge*-based undergraduate assessment outcomes are local, not nationally standardised measures
- There were relatively high rates of missing data in the latter years of the study, especially in relation to undergraduate *skills*-based exams

Word count, excluding title page, abstract, article summary, references, figures and tables is 5,800

INTRODUCTION

Internationally, there is high competition for places to study medicine, and the UK is no exception. Along with the academic demands of medicine as a subject, this has driven medical schools to use secondary (high) school performance as a major determinant to offer a place or not. In general, relatively high obtained (or predicted) grades at senior school are required before a candidate is considered as a potential entrant to medical courses. This emphasis on *prior educational attainment* ('PEA'- the grades obtained at formal exams during secondary education) has partly driven the over-representation of socio-economically privileged individuals in medicine. For example, in North America the majority of US medical school entrants are from relatively affluent backgrounds with around half coming from families in the top fifth for national income.[1] This issue is inevitably reflected in the educational backgrounds of students- it was recently highlighted that 80% of those studying medicine in the UK applied from only 20% of the country's secondary schools.[2] Most of the secondary schools that provide medical students are selective schools, which are better resourced compared to the non-selective schools. Selective schools

For peer review only - http://bmjopen.bmj.com/site/about/guidelines.xhtml

For peer review only

are also highly attended by students in higher social economic backgrounds. Therefore differences in performance between selective and non-selective schools reflect, to a high degree, differences in material deprivation rather than intellectual ability of the students from those schools.[3]

It was partly with this in mind that 'aptitude' tests, mainly tapping into cognitive domains were introduced into medical selection.[4] Such aptitude tests were first used to complement PEA in selection for undergraduate students in the USA in 1928 when the Medical College Admission Tests (MCAT) was developed to address high attrition rates in undergraduate medical school.[5,6] Since this time the use of such tests for selection has spread to other parts of the world.[7-16] PEA has been demonstrated to have predictive validity for undergraduate medical school outcomes in Australia,[17] South Korea,[18] the UK,[19] Saudi Arabia,[20] India,[21] the Czech Republic [22] and New Zealand.[23]. Aptitude tests such as the *Medical College Admission Tests (MCAT)* in the US [24] *Biomedical Admission Test (BMAT)* and *United Kingdom Clinical Aptitude Test (UKCAT)* in the UK, [3,12] *Undergraduate Medicine and Health Sciences Admission Test (UMAT)* in New Zealand,[25] *Hamburg Medical School Natural Science Test (HAM-Nat)* in Germany,[11] *Saudi National Aptitude Exam* in Saudi Arabia [20] and t*he Health Professions Admission Test- Ireland (HPAT-Ireland)* in Ireland [26] have predictive validity for medical school outcomes. Indeed, some critics have highlighted that such aptitude tests may tap into similar constructs as traditional metrics of academic achievement such as high school grades. If this is the case then such measures are unlikely to either facilitate widening access to medicine or add value within the selection process in general.

For peer review only - http://bmjopen.bmj.com/site/about/guidelines.xhtml

Some aptitude tests, such as the BMAT [9] and MCAT,[5] evaluate semantic knowledge of biomedical sciences. These tests may predict undergraduate medical performance, at least in the early years, but are unlikely to add predictive value above and beyond traditional measures of academic attainment.[27] Other tests place more weight on evaluating fluid concepts of cognitive ability, such as the UKCAT.[10] In the case of the UKCAT some, albeit modest, ability to predict undergraduate performance, even after controlling for the effects of secondary school achievement, has been demonstrated.[28] However, it is currently unclear how the predictive abilities of the UKCAT are mediated by PEA, and the extent to which this may vary across both the type of academic outcome and the five year period of undergraduate education in the UK. It has been further suggested that the UKCAT scores may be somewhat less sensitive to the type of secondary school attended, compared to the A-levels sat by students in England and Wales in their final year of schooling.[29] A-levels, usually in three subject areas, are generally undertaken in the last two years of secondary schooling and are roughly equivalent to Advance Placement (AP) courses taken by some students in North America. Findings from an earlier, cross-sectional, study suggested that a strong use of the UKCAT scores during the admissions process may mitigate some of the disadvantage faced by certain under-represented groups applying to study medicine.[30] However, a subsequent study, using longitudinal data, did not report consistent effects over time in this regard.[31]

Whilst PEA does predict academic outcomes in higher education previous studies have observed an inverse relationship with the performance of the secondary (high)

For peer review only - http://bmjopen.bmj.com/site/about/guidelines.xhtml

school attended i.e. students from more highly performing schools tend to get poorer degree awards, after controlling for PEA.[32] To date, the evidence relating to this potential effect in medical school has been inconsistent. One national study observed such an effect in the first year of medical undergraduate training for overall academic performance.[3] A separate, local, study did not.[33] Certain medical courses, designed to widen access to medicine, already 'discounted' requirements for certain groups. For example, in Australia a scheme to encourage recruitment to remote, underserved areas, relaxes entry requirements for candidates from rural backgrounds.[34] In the US 'affirmative action' policies, albeit at times controversial and repeatedly legally challenged, have been implemented to encourage those from under-represented ethnic groups to enter medical school.[35] In the UK a number of universities have started to offer reduced academic entry requirements for A-level (high school) grades to students from disadvantaged backgrounds who have attended poorly performing secondary schools.[36, 37] Other medical schools are following suit.[38] However, evidence to support such admissions strategies is currently lacking. In the UK, individuals who wish to study at a UKCAT consortium medical school sit the test prior to making an application. The decision to make an offer, for those still at secondary school, is partly based on the predicted A-level (or equivalent) grades. This choice is commonly also informed by early achievement at the GCSE exams, usually taken earlier in the applicant's school career. Therefore, any offers made would then be conditional on the specified scores obtained first at the UKCAT test before the end of secondary school and later grades being achieved at A-level at end of the secondary school education within each medical school selection cycle. Thus the present study had two aims:

For peer review only - http://bmjopen.bmj.com/site/about/guidelines.xhtml

1. To determine the extent to which the predictive powers of the UKCAT are mediated via PEA, for two separate domains (undergraduate *knowledge* and *skills*-based outcomes) over the period of undergraduate training. Since cognitive ability and educational attainment correlate, we attempt to achieve a more accurate assessment of the relative, and unique, contribution UKCAT scores make within the selection process.
2. To appraise the influence of the performance of the previous secondary school attended on an undergraduate's achievement in medical school. These results will usefully inform policy on grade discounting for applicants applying from poorly performing schools.

For this study we had an opportunity to link national data on the performance of secondary schools to cognitive ability (as evaluated via the UKCAT), PEA and outcomes at 18 UKCAT-consortium medical schools. Thus, there was also the possibility to better understand the interplay between secondary school-level performance, an individual's cognitive ability, their educational attainment (PEA) and how these related to subsequent undergraduate academic achievement. It was therefore hoped that a relatively sophisticated approach to modelling could help understand the role of secondary schooling in both selection (partly based on PEA and aptitude test scores) and later attainment at undergraduate level.

Our findings will inform selection policy in medical school, and in particular provide guidance on the extent to which grades should be discounted for applicants from poorly performing secondary schools.

For peer review only - http://bmjopen.bmj.com/site/about/guidelines.xhtml

METHODS

Data availability and quality

UKCAT consortium medical schools are those medical schools that utilise UKCAT for selection in the UK. For this study, data were available for 18 UKCAT consortium medical schools in England and Wales for candidates who were enrolled between 2007 and 2013. However, Department for Education data on the performance of English secondary schools were only linked to the 2008 entry cohort. For this reason only data relating to these students were used in this study. It should be noted that an advantage of using the 2008 entry cohort was the relatively little missing data throughout the first four of the five year undergraduate period studied. In the 2007 UKCAT testing cycle there were 26 UKCAT-consortium medical schools. Therefore the data represented 69% of the 26 UKCAT-consortium medical schools. All medical school applicants who sat for the UKCAT in 2007 and were selected to join one of the 18 UKCAT-consortium undergraduate medical schools in 2008 were included in this study. As with similar previous studies, non-standard medical courses (e.g. 'widening participation', graduate entry etc) were excluded.[28] Only the marks attained at first sittings of undergraduate exams were retained for each student. Data relating to UKCAT scores and secondary school attainment were available for 2,107 students who entered medical school in 2008 and had linked data relating to the performance of the secondary school they attended.

The secondary school exams sat by the students were nationally standardised and included General Certificate of Secondary Education (GCSE), Advanced Subsidiary (AS) Level and Advanced Level ('A-Level) exams. The GCSE exams are taken at

For peer review only - http://bmjopen.bmj.com/site/about/guidelines.xhtml

around the age of 15-16 years. Those aspiring to eventually entering higher education usually take at least 10 subjects at GCSE level. At the time of the study, the AS levels were sometimes taken in the first year of sixth form (equivalent to high school junior year) as preparation for, or to supplement the full A-level exams taken the subsequent year. For those planning to apply for medicine three subjects at A-level are studied in the last two years of secondary schooling, almost always in the sciences. Candidates frequently take more than three A-levels though universities only count the highest three grades, that usually must be achieved at first sitting.

The completeness of the data relating to the outcomes of interest varied and the flow of the data in the study is depicted in Figure 1.

[Place Figure 1 about here]

The manner in which data related to undergraduate performance in the UKCAT consortium of universities has been collated and managed has been previously described.[28] However, to summarise, the main outcome variables used were the scores achieved at undergraduate *knowledge* and *skills*-based end of year outcomes. It was left to individual institutions to define how their assessments fell into each category. These assessment scores were provided by the universities in percentage forms (of maximum marks achievable) and then converted to standardised z-scores within each institution. Thus, the z scores were created by subtracting the mean performance for that particular year and medical school cohort from an entrant's score and dividing it by the standard deviation for their peers' scores. This created standardised scores with mean zero and a standard deviation

For peer review only - http://bmjopen.bmj.com/site/about/guidelines.xhtml

of one for each medical school group of students. This standardisation was carried out in order to minimise the impact of any variability across medical schools, in terms of the nature of the assessment.

The UKCAT consists of four multiple choice sub-tests timed separately namely *quantitative reasoning, decision analysis*, *verbal reasoning* and *abstract reasoning. Quantitative reasoning* assesses an applicant's ability to critically evaluate information presented in numerical form; *decision analysis* assesses the ability to make sound decisions and judgements using complex information; *verbal reasoning* assesses the ability to critically evaluate information that is presented in a written form, and; *abstract reasoning* assesses the use of convergent and divergent thinking to infer relationships from information. Each of the cognitive subtests have their raw score converted to a scale score that ranges from 300 to 900. Therefore the total scale scores for all of the four subtests range from 1,200 to 3,600. The UKCAT subtests and their total scores were standardised as z-scores according to the scores for all candidates at the year of sitting. The reliability of the UKCAT subtests has previously been evaluated and reported.[39] For the purposes of this study only the total UKCAT score (i.e. the summed total of all four sub-test scores) was used as a predictor. This is because it is the total score that is generally used in selection and represents a summary measure of all the four subtest scores. Full details of the descriptive statistics relating to total UKCAT scores are provided in section 1 of the supplementary document.

For peer review only - http://bmjopen.bmj.com/site/about/guidelines.xhtml

In order to develop an overall, and precise, measure of PEA we implemented a novel approach that extended one previously used by McManus et al.[3, 40] This involved conceptualising 'educational achievement' as a common factor ('latent trait'). Latent traits cannot be observed or measured directly, only by their effects on behaviour. In terms of attitudes this could be observing certain responses to questionnaires, or in the case of ability, performance on exams and other assessments. Thus, in this case we treated all the commonly taken national exam grades (i.e. GCSE, AS and A-levels) as 'indicators' (i.e. observable markers) of an underlying ability (PEA). This approach allowed us to use information contained in all the commonly sat exams during secondary school in England to estimate the overall underlying educational achievement of an entrant. Because the specific method we used easily accommodated missing 'indicators' it was irrelevant if only a minority of entrants had taken a specific exam (e.g. history GCSE) and such grades could still be included when estimating PEA. The process resulted in a factor score estimate for each entrant which was provided as a standardised z score, where the mean was zero (average PEA for all applicants, with a standard deviation of 1). Thus this measure of previous educational achievement provided more information on an individual than merely their 'best of three' A-level grades. Further details of the estimation of the PEA from the reported GCSEs, AS and A-level grades are provided in section 2 of the supplementary document.

This estimate of PEA was used in the models addressing the first study aim (evaluating the mediating effects of previous educational attainment on the UKCAT's ability to predict undergraduate performance). However, 'discounting' policy focuses on the 'best of three' A-level grades required for entry, usually after a provisional

For peer review only - http://bmjopen.bmj.com/site/about/guidelines.xhtml

offer has been made to an applicant. Therefore for the models addressing the study's second aim (role of secondary school-level performance on undergraduate outcomes) we banded entrants into categories according to A-level grades. Thus, the entrants were grouped into three bands according to the highest three A-level grades achieved. Only 43 (2%) entrants were recorded as having the relatively low A-level grades 'BBB' and 'BBC'. Thus entrants were grouped into those with grades 'AAA', 'AAB' and 'ABB or lower'.

English secondary school-level performance data for 2008 were available from the Department for Education (DfE). Thus for this study we defined secondary school-level performance as the average grades (converted to a numeric score) achieved for each student on roll at that educational establishment for that school year. Further details are available from the Department for Education for England website. In this sense 'performance' is (narrowly) defined as the average educational attainment, in terms of formal exam grades achieved, for each student on roll, in that educational establishment.

MODELLING APPROACHES

Modelling the relationship between UKCAT scores, PEA and Undergraduate outcomes

Our first aim was to try and understand the extent to which the ability of the UKCAT scores to predict subsequent undergraduate medical school performance were explained by PEA. To answer this question a mediation model was developed. The outcomes of interest (undergraduate *knowledge* and *skills*-based exam results) were local to each participating medical school. The variation in the assessment results across institutions was initially explored using a multilevel modelling approach, but

For peer review only - http://bmjopen.bmj.com/site/about/guidelines.xhtml

no statistically significant clustering effects by university were observed. For this reason, a simpler approach using a single-level mediation model was used for the analysis (Figure 2). Further details of the single-level mediation model, the multi-level mediation model and rationale for choosing the single-level mediation model are described in section 3 of the supplementary document.

[Place Figure 2 about here]

Modelling the influence of secondary school performance on undergraduate outcomes

The second aim of this study was to evaluate the influence of the performance of an entrant's previous secondary school on subsequent undergraduate achievement. This involved estimating this secondary school-level effect while controlling for an entrant's A-level grades. A multilevel model was required to account for the variation in outcomes between universities.[41] Further details on the multi-level model can be found in section 4 of the supplementary document. From the model we could derive predictions about entrants' performances at medical school, for varying A-level grades and secondary school performance.

The statistical analyses were conducted using Mplus version 7.4, R and SAS softwares.[42-44] Lucidchart [45] was used to produce the figures and R software was used to generate the graphs of the model predictions.

For peer review only - http://bmjopen.bmj.com/site/about/guidelines.xhtml

RESULTS

Descriptive statistics

The numbers of entrants with outcomes available in each category (type and year) are depicted in Table 1. This was not a cohort study in the conventional sense (i.e. entrants could leave and enter the study at any year based on a university deciding when to (not) report the academic outcome measures). Thus, Table 1 also illustrates the missingness for only those entrants who had reported undergraduate *knowledge* and *skills*-based outcomes in the first year of undergraduate medical school. This is to provide a picture of attrition in the conventional sense (i.e. how many participants at baseline remained at subsequent time-points).

	**Undergraduate Knowledge-based outcome**				**Undergraduate Skills based outcome**	
**Academic Year**	**Number of universities**	**Number of students**	**% *Missing***	**Number of universities**	**Number of students**	**% Missing**
1	13	1,453	-	9	1,051	-
2	13	1,404	3.72	9	1,019	3.04
3	11	1,041	28.36	7	729	30.64
4	7	711	51.07	5	668	36.44
5	4	439	69.79	2	260	75.26

Table 1: Study attrition rates due to missing data only for those students who had outcome measures reported in year one of medical school

Section 5 in the supplementary document provide a detailed summary of the missing data patterns for the outcomes. Of the 2,107 undergraduate medical school entrants, 1,855 had their secondary school-level performance available. The

For peer review only - http://bmjopen.bmj.com/site/about/guidelines.xhtml

distribution of secondary school-level performance and UKCAT scores achieved by the entrants are depicted in Table 2.

	Year of UKCAT sitting=2007				
	**Sample size**	**Mean**	**SD**	**Minimum**	**Maximum**
Average Secondary School-level performance	1,855	225.18	20.09	145	267.5
UKCAT total score	2,107	2,544.47	188.92	1,950	3,190

Table 2: Descriptive statistics of the UKCAT total score and average point entry for the 2,107 entrants from the 987 schools.

Table 3 shows the distribution of A-level grades for the medical school entrants. Note that the majority of the entrants had achieved either AAA or AAB grades at A-level.

Grade	N	%
Missing	36	1.71
AAA	1,463	69.44
AAB	436	20.69
ABB	129	6.12
BBB	29	1.38
BBC	14	0.66

Table 3: A-level grades for the entrants in the study sample.

For peer review only - http://bmjopen.bmj.com/site/about/guidelines.xhtml

The prediction of medical school outcomes from UKCAT performance

Figure 3 summarises the results from the models investigating the potential mediating effects of PEA on the relationship between UKCAT scores and undergraduate exam outcomes. The proportion of the predictive power of the UKCAT explained by PEA shown for both undergraduate *knowledge* and *skills*-based medical school outcomes are computed as a quotient of indirect effect of UKCAT through PEA divided by total effect of UKCAT.

[Place Figure 3 about here]

Overall, PEA explains approximately over 43% (dotted black line in the Figure 3) of the statistically significant predictive power of the UKCAT for both undergraduate *knowledge* and *skills*-based exams only in the preclinical years (one and two) of medical school training. For the clinical years (three to five) PEA explains approximately less than 43% of the predictive power of the UKCAT for both undergraduate *knowledge* and *skills*-based exam outcomes. This proportion remains statistically significant but declines somewhat with every subsequent year of training.

The effect of secondary school-level performance on subsequent medical school performance

Both secondary school-level performance and PEA were statistically significantly related to the undergraduate outcomes. No statistically significant interaction was observed between the two variables. Overall, compared to entrants from secondary schools with a high average student performance, those from schools with lower

For peer review only - http://bmjopen.bmj.com/site/about/guidelines.xhtml

average attainment tended to have better subsequent scores in both undergraduate *knowledge* and *skills*-based exams. Lower levels of secondary school level performance corresponded with higher standardised undergraduate medical school performance as may be observed in Figures 4 and 5.

We intended to make our results relevant to UK medical selectors. Specifically we wished to estimate the level of 'discounting' that should be offered to applicants from disadvantaged educational backgrounds. Thus the results of our models addressing the second study aim are depicted in Figures 4 and 5. We show the actual and predicted (fitted) values from the models in the Figures. Average secondary school performance (mean enrolled student attainment for all secondary schools in England) is shown on the horizontal axis and predicted medical school performance (as a standardized z score) on the vertical axis.

Figure 4 depicts the values in relation to *knowledge*-based exams, according to secondary school-level performance. Similarly, Figure 5 shows the values for undergraduate *skills*-based outcomes. Superimposed on these plotted values are the estimates (with associated 95% confidence bands) for entrants depending on their A-level grades at admission to university. These represent the entrants within the three bands of A-level attainment ('AAA', 'AAB', and 'ABB or lower'). For purpose of demonstration, the horizontal black dotted lines indicate the equivalent level of performance between those entrants from secondary schools at the lower decile of performance and those at the upper decile.

For peer review only - http://bmjopen.bmj.com/site/about/guidelines.xhtml

There are a number of notable trends observed in these graphs. Firstly, students with higher A-level grades outperform those with lower educational achievement. However, this gap narrows when predicting undergraduate *skills*, rather than undergraduate *knowledge*-based outcomes in medical school. The difference also reduces in magnitude as undergraduate education progresses through the years. Indeed for undergraduate *skills*-based outcomes, and for many of the later years, the confidence intervals for the groups' estimates generally overlap. This indicates no statistically significant inter-group differences between those with 'AAB' and 'ABB or lower grades' at the 95% confidence level.

The second most striking feature, and the focus of this study, is that students from less highly performing secondary schools generally outperform those from more highly performing educational institutions for any given A-level grade banding. That is, controlling for the effects of A-level attainment, on average, those from the more poorly performing schools tend achieve better undergraduate exam results than those from the schools with higher levels of student attainment. The vertical purple and brown dotted lines highlight this feature. They show that those with lower A-level grades (e.g. AAB or ABB) from the lowest performing secondary schools tend to have equivalent undergraduate performance to those entrants from the highest performing educational establishments with top grades (i.e. AAA). It is also notable that this 'secondary school gradient' is generally steepest for undergraduate *knowledge*-based outcomes in the early years of undergraduate study. Thus, the effects of secondary school environment, as with individual previous educational attainment, tends to be less marked for procedural (undergraduate *skills*-based) learning and with advancing time in university study.

For peer review only - http://bmjopen.bmj.com/site/about/guidelines.xhtml

[Place Figure 4 about here]

[Place Figure 5 about here]

DISCUSSION

The findings from previous studies suggested some modest added value of the UKCAT scores to predict undergraduate performance, over and above that provided by conventional measures of academic achievement.[3, 28] Further, the ability of UKCAT scores to predict certain aspects of undergraduate performance was found to be largely independent of prior educational attainment (PEA). This was less true for both undergraduate *knowledge* and *skills* based exams, taken early on in the preclinical years of medical school, where a significant portion of the UKCAT's predictive ability is mediated via previous educational performance.

Our findings on the role of secondary school quality in determining subsequent undergraduate performance are in line with the findings from a previous national study utilising data from the same cohort, as well as more general analysis of data from higher education in England.[3, 32] However, we were able to demonstrate persistence (though attenuation) of these effects over the five years of medical school. It is also in keeping with recently published findings that showed that medical students from state-funded (mainly non-selective) secondary schools tended to academically outperform those from privately funded schools, once at university.[46] Our findings were also consistent with those from an Australian study. This reported that entrants from rural backgrounds tended to have lower educational achievement, both at entry and in the early, pre-clinical years of study. However there were no

For peer review only - http://bmjopen.bmj.com/site/about/guidelines.xhtml

significant inter-group differences in performance observed in the latter, clinical years of undergraduate training. However, some caution must be exercised in interpreting these findings as the study was single site with a relatively small number (N=856) of participating students.[34] The present findings were in contrast to those of a local study, which focussed on the fourth year of medical school, when the effects of secondary schooling are likely to have been less marked.[33] The relatively low numbers of students (N=574) involved in this latter study may have led to a deficiency in study power and thus an inability to demonstrate these effects. Also, by using a more sophisticated approach to statistical modelling we were able to delineate the direct and indirect (mediational) effects of cognitive ability (as assessed via the UKCAT) in determining undergraduate medical academic performance. This highlighted the shifting relative roles that conventional academic achievement versus cognitive ability play as undergraduate training progresses. We were also able to separate, at least crudely, undergraduate outcomes in this study relating to '*knowledge*' and '*skills*' (see also limitations, below). As expected, traditional academic attainment (in the form of PEA), was more predictive and mediated a greater proportion of the UKCAT effects for earlier exam performance. We also observed a narrowing of the effects of secondary education achievement as medical school progressed. This might be expected- as the time since leaving secondary schooling elapses it becomes less relevant to current academic performance. However, this narrowing gap may be due to a positive influence of the university educational environment, which may render prior disparities in educational achievement between students less influential. Alternatively, the shrinking disparity may be, at least in part, due to the students becoming more homogenous over time. Some, less well performing or motivated students, will leave the courses in earlier

For peer review only - http://bmjopen.bmj.com/site/about/guidelines.xhtml

years. Nevertheless, in the UK, as elsewhere, such medical school attrition rates (for all reasons) are very small, ranging from approximately 0.25% for the first year to 0.1% for the final year, for standard entry courses.[47] Therefore this effect will have been only slight. In addition, as medical school progresses there is an increasing emphasis on procedural (undergraduate *skills*-based) learning. Thus, the academic abilities required to highly achieve at written school exams are likely to become less relevant to performance.

Our findings also build on previous research [3] and we were able to demonstrating the value, to some extent of 'contextualising' secondary school achievement across the medical undergraduate years. That is, to some extent, the grades obtained by a student at secondary school must be put in the context of the educational establishment in which they were obtained. A reduction of one to two A-level grades may not appear to be a large adjustment. However, this must be understood in the light of the highly homogenous nature of both medical school applicants and entrants where high proportions obtain the maximum achievable grades. Thus, even one grade difference could represent a standard deviation or so from the mean in a pool of high achieving medical school entrants. Internationally, selectors must understand their equivalent effects, not just for school-type attended, but a range of contextual factors that may be pertinent to their culture. Similarly, they must translate such effects into discounted offers where appropriate, in the metric of their own educational systems.

The main strength of this study is that there were a relatively large number of entrants studied from a range of UK medical schools involved. This provided

For peer review only - http://bmjopen.bmj.com/site/about/guidelines.xhtml

sufficient study power to enable the elicitation of relatively subtle effects and suggests the findings are generalizable to England and Wales. Moreover, the secondary school exams sat by this cohort were nationally standardised, with only a minority of the credits awarded for course work. Thus, any local or regional variation in standards can be assumed to be trivial. Nevertheless a number of limitations must be borne in mind when interpreting the findings. In terms of the outcome measures, the categorisation of undergraduate exams into *skills* and *knowledge* was not operationalised and therefore rely on the participating medical schools to categorise the evaluations. Thus their definition may vary across medical schools. Whilst some of this variation was handled by the use of multilevel modelling a more robust definition of undergraduate '*skills*' based assessments may have been helpful in predicting clinically-orientated performance, which may have been a more faithful proxy for later medical practice. In this regard, a methodology has been proposed to achieve this through the "nationalisation" of "local" measures of undergraduate medical school performance for fair comparisons of graduating medical doctors.[48] It is also acknowledged that it is generally the case that undergraduate skills-based exams to be less reliable than knowledge-based tests.[49] It is thus possible that this likely disparity in reliability may explain the difference in the magnitude of observed relationships associated between the predictors and the two undergraduate medical school outcomes. Thus lower reliability in the measurement of an outcome would have an attenuating effect on strength of the relationships.[19] In addition, scores from the most recently taken UKCAT scores were used. These may not have been a better metric of underlying cognitive ability (being less prone to practice effects), though some early sittings may have been used as 'practice runs' by medical school

For peer review only - http://bmjopen.bmj.com/site/about/guidelines.xhtml

applicants. In addition, the most recent UKCAT test results are those used by selectors, thus the ones most relevant to selection policy.

The number of participating universities in the study varied from year to year with higher levels of missing data for undergraduate *skills*-based assessments (compared to *knowledge*) and for the latter years of study. This was a result of medical schools deciding not to return outcome exam data for that year rather than students exiting the study or dropping out from medical school. Therefore the missing data mechanism is likely MCAR or potentially MAR. This was dealt with by modelling the data using a likelihood approach and conducting sensitivity analysis to determine the effect of missingness through Multiple Imputation. Both likelihood modelling approach and Multiple Imputation are valid data handling methods under MCAR and MAR.[50, 51] The results from imputed versus non-imputed datasets can be compared as a form of sensitivity analysis (see section 6 of the supplementary document). These highlight that the results did not vary significantly between imputed versus non-imputed datasets. Therefore, missing data did not adversely impact the results and conclusion of the study.

The quality of secondary schools previously attended by undergraduate medical school entrants varies widely across the UK. However, the fact that 80% of UK medical students come from 20% of secondary schools [2] and tend to come from economically advantaged backgrounds.[52] Thus students from selective, academically high-performing schools are grossly over- represented at medical school. Indeed, a selection process substantially based on predicted or actual A-level performance will greatly advantage applicants from such educational

For peer review only - http://bmjopen.bmj.com/site/about/guidelines.xhtml

institutions. Paradoxically such students, once admitted, may relatively underperform in medical school, compared to their contemporaries from less well performing schools, which tend to be state funded and non-selective in nature. Already some UK medical schools are offering 'discounted' A-level offers to applicants from schools that have students with lower levels of academic attainment.[53-55] Our results suggest that such medical schools may have been (albeit serendipitously) implementing such polices broadly in line with our present findings. That is to say, entrants from the most poorly performing schools have A-level grades that 'worth' one to two grades more than those from the top performing schools, in terms of their ability to predict undergraduate achievement. As can be seen from Figures 4 and 5 the definition of 'low' and 'high' performing secondary school is somewhat subjective. In addition, the suggested 'discounting' would vary according to the outcome of interest. There are also practical challenges to implementing such policies. Not all applicants to medical school will have attended schools which can supply comparable data on their institutional performance. At present even comparison across the three nations making up the UK would be very difficult. One simple way of 'equating' across countries might be to report an applicant's rank within their school. However, further evaluation would have to be performed to assess whether such a relatively crude approach was an effective way of contextualising educational achievement. There is also the possibility of 'gaming' with economically advantaged families strategically placing a student in a less well performing educational institution for the final year of schooling.

Any moves to widen access to medicine may prove controversial, as advantaging certain candidates necessarily means disadvantaging others. Thus, such policies

For peer review only - http://bmjopen.bmj.com/site/about/guidelines.xhtml

must be based on defensible evidence, such as the kind we believe is offered by this study. Moreover, given the very low absolute numbers of applicants and entrants to medical schools from disadvantaged socioeconomic backgrounds only a radical rethinking of ‘widening access’ is likely to result in substantial changes to the demographics of the medical workforce.

To conclude, we found that the predictive ability of the UKCAT can be explained to some degree by PEA, although this is more pronounced in the early preclinical years of undergraduate school. Significant effects of secondary school-level performance exist which suggest the issue of whether offers of a place to study should be discounted for students from more poorly performing schools. This highlights an urgent need to ‘contextualise’ secondary school performance in applicants rather than selectors taking grades at face value.

For peer review only

For peer review only - http://bmjopen.bmj.com/site/about/guidelines.xhtml

DECLARATIONS

Contributors

All authors made substantial contribution to this study. LMM conducted the statistical analyses and contributed to the writing of the article. PAT and ASK led the conception, design, supervision of the statistical analyses, interpretation of the results and contributed to the writing of the article. JRB was involved with supervision of the statistical analysis and handling of missing data, interpretation of the results, revising and writing of the manuscript. LWP was involved in the interpretation of the results, revising, writing, and critical appraisal of the manuscript. All authors have approved the final version of the article submitted.

Acknowledgements

The authors thank Rachel Greatrix at UKCAT for assistance with facilitating access to the data used in this study.

Funding

LMM is supported in his PhD, of which this study is a component, via funding from the UKCAT Board. PAT is currently supported in his research by a National Institute for Healthcare Research (NIHR) Career Development Fellowship. PAT is also lead for the DREAMS Network, an international collaboration on selection into the professions, of which LMM, JRB and LWP are also members, which is funded by a Worldwide University Network (WUN) Research Development Fund award.

For peer review only - http://bmjopen.bmj.com/site/about/guidelines.xhtml

This paper presents independent research part-funded by the National Institute for Health Research (NIHR). The views expressed are those of the authors and not necessarily those of the NHS, the NIHR or the Department of Health.

Competing interests

LMM is supported in his PhD project via funding from the UKCAT Board and has received travel expenses incurred for attending a UKCAT Research Group meeting. PAT has previously received research funding from the Economic and Social Research Council (ESRC), the Engineering and Physical Sciences Council (EPSRC), the Department of Health for England, the UKCAT Board, and the General Medical Council (GMC). In addition, PAT has previously performed consultancy work on behalf of his employing University for the UKCAT Board and Work Psychology Group and has received travel and subsistence expenses for attendance at the UKCAT Research Group.

Ethical approval

No human subjects were tested for this study therefore no ethical approval was necessary. The anonymised raw data used in these analyses were made available by the UK Medical Education Database (www.ukmed.ac.uk) following approval of an application. From the data, no piece of information can be used to identify a secondary school, medical school or individual. As such, the identity of the participants is fully protected. All participants consented to the collection of the data for research.

For peer review only - http://bmjopen.bmj.com/site/about/guidelines.xhtml

REFERENCES

1. Grbic D, Jones DJ, Case ST. The role of socioeconomic status in medical school admissions: validation of a socioeconomic indicator for use in medical school admissions. Academic Medicine. 2015;90(7):953-60.

2. Medical School Council. Selecting for Excellence Final Report. 2014. https://www.medschools.ac.uk/media/1203/selecting-for-excellence-final-report.pdf. (accessed 3 March 2017).

3. McManus I, Dewberry C, Nicholson S, Dowell JS. The UKCAT-12 study: educational attainment, aptitude test performance, demographic and socio-economic contextual factors as predictors of first year outcome in a cross-sectional collaborative study of 12 UK medical schools. BMC medicine. 2013;11(1):244.

4. United Kingdom Clinical Aptitude Test (UKCAT) Consortium. UKCAT Annual Report. 2007. https://www.ukcat.ac.uk/media/1135/annual-report-2007-2008.pdf. (accessed 9 September 2017).

5. Association of American Medical Colleges (AAMC). About the Medical College Aptitude Test. 2017. https://students-residents.aamc.org/applying-medical-school/taking-mcat-exam/about-mcat-exam/. (accessed 6 June 2017).

6. McGaghie WC. Assessing readiness for medical education: evolution of the medical college admission test. Jama. 2002;288(9):1085-90.

7. Australian Council for Educational Research (ACER). Health Professions Admission Test-Ireland (HPAT-Ireland). 2017. https://hpat-ireland.acer.org/. (accessed 3 March 2017).

For peer review only - http://bmjopen.bmj.com/site/about/guidelines.xhtml

8. O'Flynn S, Fitzgerald T, Mills A. Modelling the impact of old and new mechanisms of entry and selection to medical school in Ireland: who gets in? Irish journal of medical science. 2013;182(3):421-7.

9. Cambridge Assessment Admissions Testing. The BioMedical Admissions Test (BMAT). 2017. http://www.admissionstestingservice.org/for-test-takers/bmat/. (accessed 3 March 2017).

10. United Kingdom Clinical Aptitude Test (UKCAT) Consortium. UKCAT. 2017. https://www.ukcat.ac.uk/. (accessed 3 March 2017).

11. Hissbach JC, Klusmann D, Hampe W. Dimensionality and predictive validity of the HAM-Nat, a test of natural sciences for medical school admission. BMC medical education. 2011;11(1):83.

12. Emery JL, Bell JF. The predictive validity of the BioMedical Admissions Test for pre-clinical examination performance. Medical education. 2009;43(6):557-64.

13. Australian Council for Educational Research (ACER). Undergraduate Medicine and Health Sciences Admissions Test (UMAT). 2017. https://umat.acer.edu.au/. (accessed 3 March 2017).

14. Hissbach J, Klusmann D, Hampe W. Reliability of a science admission test (HAM-Nat) at Hamburg medical school. GMS Zeitschrift für Medizinische Ausbildung. 2011;28(3).

15. Farrokhi-Khajeh-Pasha Y, Nedjat S, Mohammadi A, Rad EM, Majdzadeh R, Monajemi F, et al. The validity of Iran's national university entrance examination (Konkoor) for predicting medical students' academic performance. BMC medical education. 2012;12(1):60.

For peer review only

For peer review only - http://bmjopen.bmj.com/site/about/guidelines.xhtml

16. Khan JS, Biggs JS, Bano T, Mukhtar O, Tabasum S, Mubasshar MH. Medical Colleges Admissions Test in Punjab, Pakistan. Journal of Ayub Medical College Abbottabad. 2013;25(1-2):64-7.

17. Mercer A, Puddey IB. Admission selection criteria as predictors of outcomes in an undergraduate medical course: A prospective study. Medical Teacher. 2011;33(12):997-1004.

18. Kim T, Chang J-Y, Myung SJ, Chang Y, Park KD, Park WB, et al. Predictors of undergraduate and postgraduate clinical performance: A longitudinal cohort study. Journal of surgical education. 2016;73(4):715-20.

19. McManus I, Woolf K, Dacre J, Paice E, Dewberry C. The Academic Backbone: longitudinal continuities in educational achievement from secondary school and medical school to MRCP (UK) and the specialist register in UK medical students and doctors. BMC medicine. 2013;11(1):242.

20. Al Alwan I, Al Kushi M, Tamim H, Magzoub M, Elzubeir M. Health sciences and medical college preadmission criteria and prediction of in-course academic performance: a longitudinal cohort study. Advances in Health Sciences Education. 2013;18(3):427-38.

21. Gupta N, Nagpal G, Dhaliwal U. Student performance during the medical course: role of pre-admission eligibility and selection criteria. 2013.

22. Štuka Č, Martinková P, Zvára K, Zvárová J. The Prediction and Probability for Successful Completion in Medical Study Based on Tests and Pre-admission Grades. Stanisław Juszczyk. 2012:138.

23. Shulruf B, Poole P, Wang GY, Rudland J, Wilkinson T. How well do selection tools predict performance later in a medical programme? Advances in Health Sciences Education. 2012;17(5):615-26.

For peer review only - http://bmjopen.bmj.com/site/about/guidelines.xhtml

24. Julian ER. Validity of the Medical College Admission Test for predicting medical school performance. Academic Medicine. 2005;80(10):910-7.

25. Poole P, Shulruf B, Rudland J, Wilkinson T. Comparison of UMAT scores and GPA in prediction of performance in medical school: a national study. Medical education. 2012;46(2):163-71.

26. Kelly ME, Regan D, Dunne F, Henn P, Newell J, O'Flynn S. To what extent does the Health Professions Admission Test-Ireland predict performance in early undergraduate tests of communication and clinical skills?–An observational cohort study. BMC medical education. 2013;13(1):68.

27. McManus I, Ferguson E, Wakeford R, Powis D, James D. Predictive validity of the Biomedical Admissions Test: an evaluation and case study. Medical teacher. 2011;33(1):53-7.

28. Tiffin PA, Mwandigha LM, Paton LW, Hesselgreaves H, McLachlan JC, Finn GM, et al. Predictive validity of the UKCAT for medical school undergraduate performance: a national prospective cohort study. BMC medicine. 2016;14(1):140.

29. Tiffin PA, McLachlan JC, Webster L, Nicholson S. Comparison of the sensitivity of the UKCAT and A Levels to sociodemographic characteristics: a national study. BMC medical education. 2014;14(1):7.

30. Tiffin PA, Dowell JS, McLachlan JC. Widening access to UK medical education for under-represented socioeconomic groups: modelling the impact of the UKCAT in the 2009 cohort. BMJ. 2012;344:e1805.

31. Mathers J, Sitch A, Parry J. Population-based longitudinal analyses of offer likelihood in UK medical schools: 1996–2012. Medical education. 2016;50(6):612-23.

For peer review only - http://bmjopen.bmj.com/site/about/guidelines.xhtml

32. Higher Education Funding Council for England (HEFCE). Differences in degree outcomes: Key findings (UMAT). 2014. http://www.hefce.ac.uk/pubs/year/2014/201403/. (accessed 3 March 2017).

33. Thiele T, Singleton A, Pope D, Stanistreet D. Predicting students' academic performance based on school and socio-demographic characteristics. Studies in Higher Education. 2016;41(8):1424-46.

34. Ray R, Woolley T, Sen Gupta T. James Cook University's rurally orientated medical school selection process: quality graduates and positive workforce outcomes. Rural and remote health. 2015;15:1-11.

35. Blake V. Affirmative action and medical school admissions. Virtual Mentor. 2012;14(12):1003.

36. Garlick PB, Brown G. Widening participation in medicine. BMJ: British Medical Journal. 2008;336(7653):1111.

37. Curtis S, Blundell C, Platz C, Turner L. Successfully widening access to medicine. Part 2: Curriculum design and student progression. Journal of the Royal Society of Medicine. 2014;107(10):393-7.

38. University of Birmingham. Medicine and Surgery MBChB: Widening Participation. 2017. http://www.birmingham.ac.uk/undergraduate/courses/med/medicine.aspx?OpenSection=EntryRequirements. (accessed 3 March 2017).

39. Tiffin PA. Understanding the Dimensionality and Reliability of the Cognitive Scales of the UK Clinical Aptitude test (UKCAT): Summary Version of the Report. 2013. https://www.ukcat.ac.uk/media/1182/understanding-the-dimensionality-and-reliability-of-the-cognitive-scales-of-the-ukcat.pdf. (accessed 3 March 2017).

For peer review only - http://bmjopen.bmj.com/site/about/guidelines.xhtml

40. McManus IC, Dewberry C, Nicholson S, Dowell JS. The UKCAT-12 Technical Report. 2012. https://www.ukcat.ac.uk/media/1185/ukcat-technicalreport-march2012-withbackgroundandsummary-sep2013v3.pdf. (accessed 3 March 2017).

41. Verbeke G, Molenberghs G. Linear mixed models for longitudinal data: Springer Science & Business Media; 2009.

42. Sas Institute. SAS software, version 9.4. SAS Institute Cary, NC; 2002-2012.

43. Muthén L, Muthén B. Mplus User's Guide. Seventh Edition. 1998-2015.

44. R Core Team (2017). R: A language and environment for Statistical computing. R Foundation for Statistical Computing, Vienna, Austria. ISBN 3-900051-07-0, URL http://www.R-project.org.

45. Lucid Software Inc. Lucidchart [Internet]. 2017. https://www.lucidchart.com/ (accessed 3 March 2017).

46. Kumwenda B, Cleland JA, Walker K, Lee AJ, Greatrix R. The relationship between school type and academic performance at medical school: a national, multi-cohort study. BMJ open. 2017;7(8):e016291.

47. General Medical Council (GMC). Summary of 2014 Medical Schools Annual Return.. 2015. https://www.gmc-uk.org/Medical_School_Annual_Return_2014_Summary_Report_for_Publication.pdf_61816122.pdf_62533230.pdf. (accessed 12 January 2017).

48. Tiffin PA, Paton LW. Exploring the validity of the 2013 UKCAT SJT- prediction of undergraduate performance in the first year of medical school: Summary Version of Report. 2017. https://www.ukcat.ac.uk/media/1119/exploring-the-validity-of-the-2013-ukcat-sjt-prediction-of-ug-performance-in-1st-yr-of-med-school-summary-version-posted-27032017.pdf. (accessed 19 September 2017).

For peer review only - http://bmjopen.bmj.com/site/about/guidelines.xhtml

49. Wass V, McGibbon D, Van der Vleuten C. Composite undergraduate clinical examinations: how should the components be combined to maximize reliability? Medical Education. 2001;35(4):326-30.

50. Schafer JL, Graham JW. Missing data: our view of the state of the art. Psychological methods. 2002;7(2):147.

51. Enders CK. Multiple imputation as a flexible tool for missing data handling in clinical research. Behaviour research and therapy. 2016.

52. Steven K, Dowell J, Jackson C, Guthrie B. Fair access to medicine? Retrospective analysis of UK medical schools application data 2009-2012 using three measures of socioeconomic status. BMC medical education. 2016;16(1):11.

53. King's College London. Extended Medical Degree Programme MBBS. 2017. https://www.kcl.ac.uk/study/undergraduate/courses/extended-medical-degree-programme-mbbs.aspx. (accessed 13 July 2017).

54. University of Birmingham. Access to Birmingham (A2B). 2017. http://www.birmingham.ac.uk/undergraduate/preparing-for-university/teachers-advisors/reaching-regional/a2b.aspx. (accessed 13 July 2017).

55. University of Birmingham. Routes to the Professions. 2017. http://www.birmingham.ac.uk/undergraduate/preparing-for-university/teachers-advisors/reaching-regional/routes-professions.aspx. (accessed 13 July 2017).

For peer review only - http://bmjopen.bmj.com/site/about/guidelines.xhtml

Figure captions

Figure 1: Flow chart of data available for the outcomes for each of the five academic years of medical school training

Figure 2: Illustration of the conceptual model for the single level mediation effect of previous educational attainment on the association between total UKCAT scores and undergraduate medical school knowledge and skills-based exams

Figure 3: Proportion of the predictive power of UKCAT for undergraduate knowledge and skills-based exam outcomes explained by PEA in medical school. The proportion is computed as a quotient of indirect effect of UKCAT through PEA divided by total effect of UKCAT

Figure 4: Effect of average school level performance by reported grades on undergraduate medical school knowledge-based exams (as a standardized z score) for all secondary schools in England in 2008. The 2nd decile (average school level performance of 200.2) and 8th decile (average school level performance of 251.9) are denoted by the purple and brown vertical lines respectively. The horizontal black dotted lines are arbitrary points chosen to indicate the equivalent level of performance between those entrants from secondary schools at the lower decile of performance and those at the upper decile of performance

For peer review only - http://bmjopen.bmj.com/site/about/guidelines.xhtml

Figure 5: Effect of average school level performance by reported grades on undergraduate medical school skills-based exams (as a standardized z score) for all secondary schools in England in 2008. The 2nd decile (average school level performance of 200.2) and 8th decile (average school level performance of 251.9) are denoted by the purple and brown vertical lines respectively. The horizontal black dotted lines are arbitrary points chosen to indicate the equivalent level of performance between those entrants from secondary schools at the lower decile of performance and those at the upper decile of performance

For peer review only

For peer review only - http://bmjopen.bmj.com/site/about/guidelines.xhtml

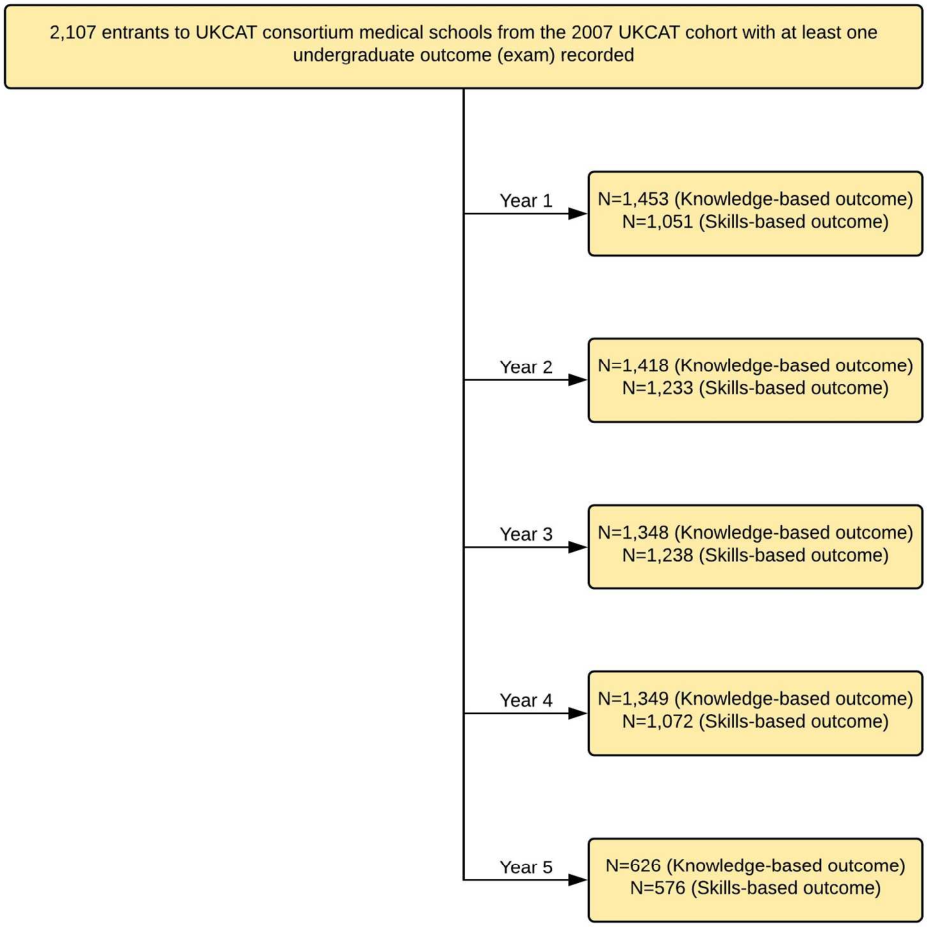
2,107 entrants to UKCAT consortium medical schools from the 2007 UKCAT cohort with at least one undergraduate outcome (exam) recorded
Year 1
N=1,453 (Knowledge-based outcome)
N=1,051 (Skills-based outcome)
Year 2
N=1,418 (Knowledge-based outcome)
N=1,233 (Skills-based outcome)
Year 3
N=1,348 (Knowledge-based outcome)
N=1,238 (Skills-based outcome)
Year 4
N=1,349 (Knowledge-based outcome)
N=1,072 (Skills-based outcome)
Year 5
N=626 (Knowledge-based outcome)
N=576 (Skills-based outcome)

Figure 1

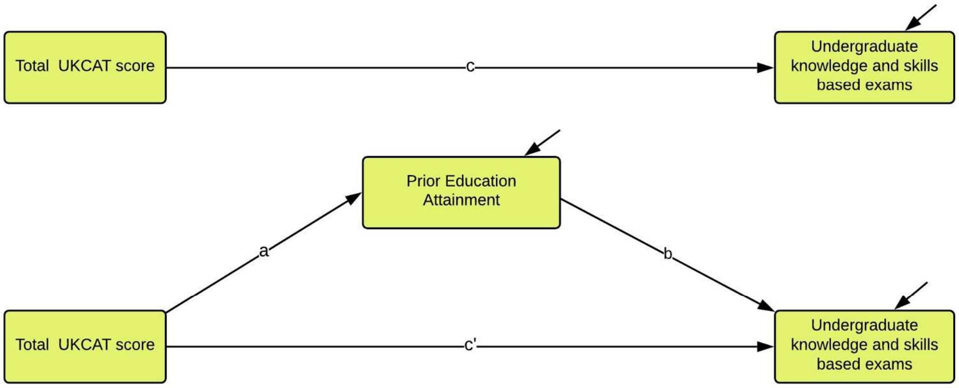

Figure 2

For peer review only

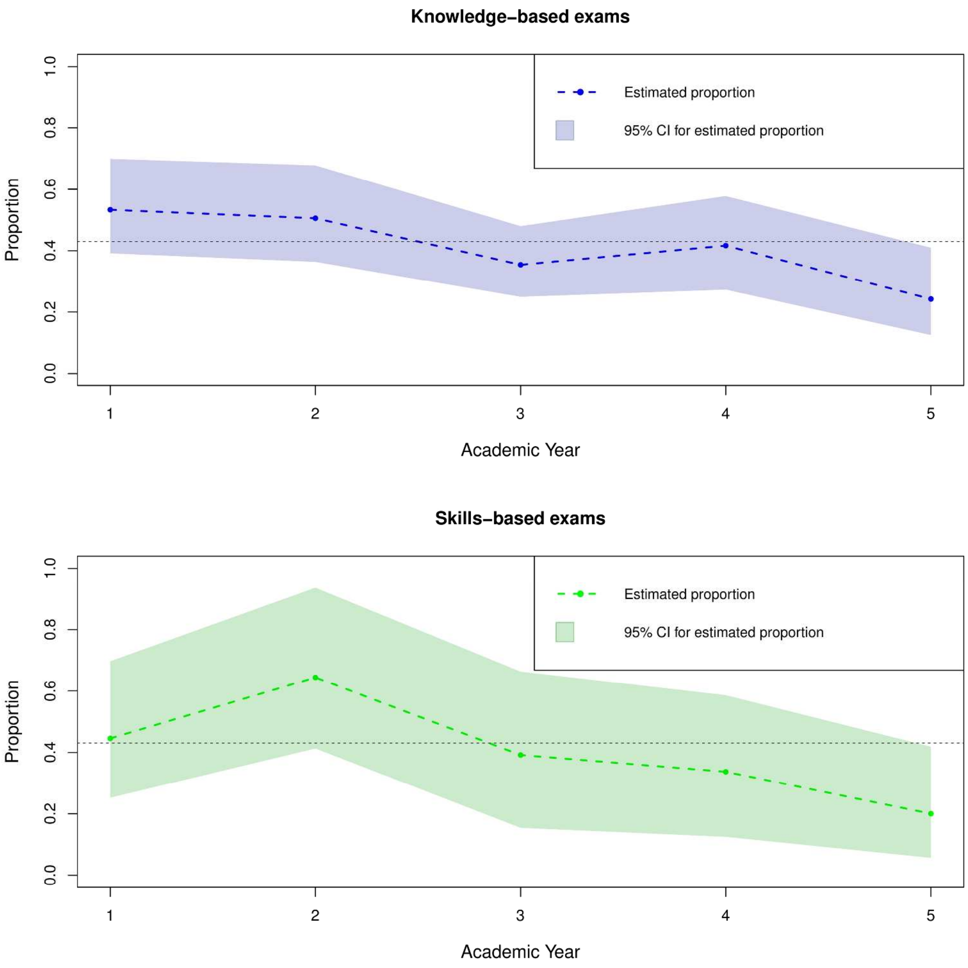

Figure 3

275x275mm (300 x 300 DPI)

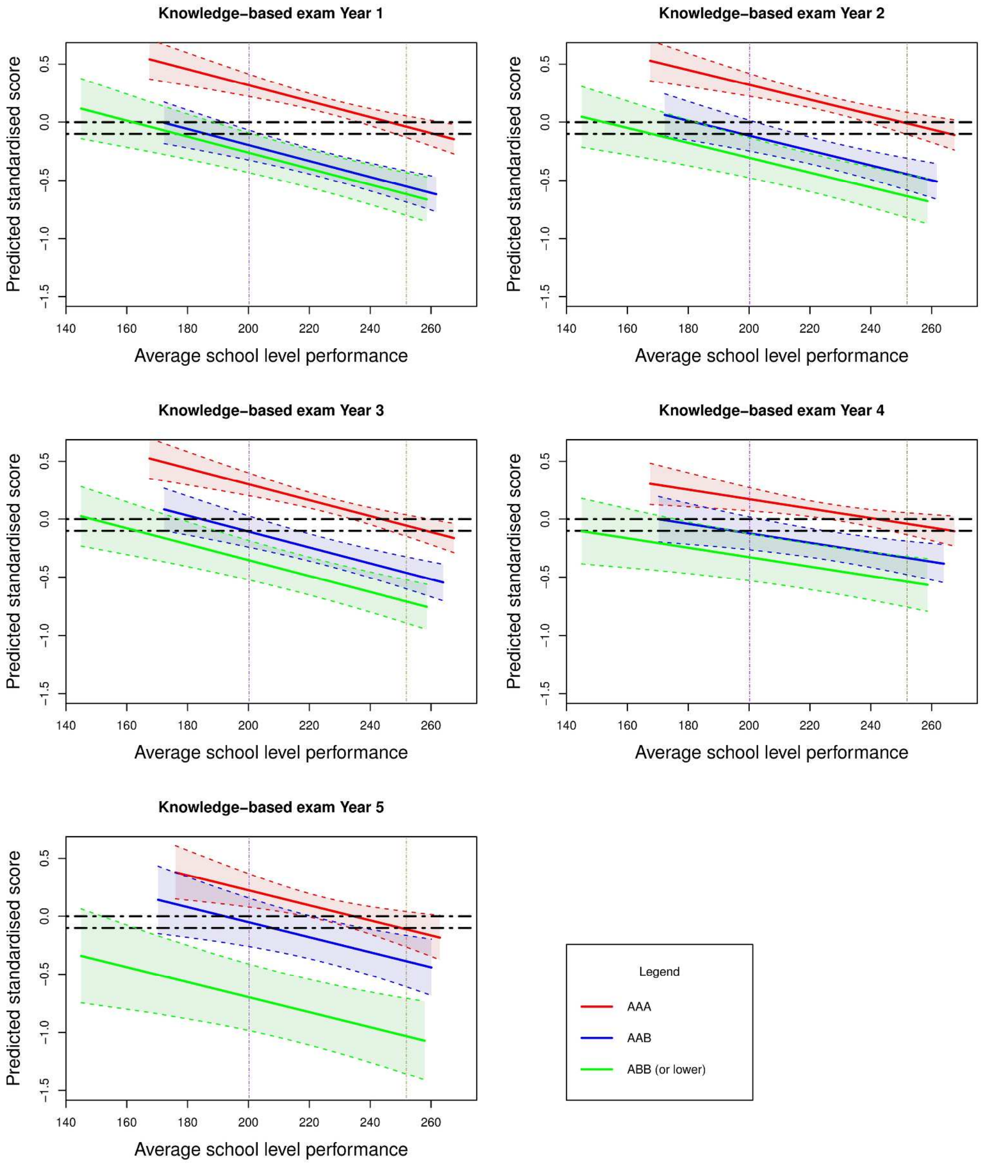

Figure 4

275x328mm (300 x 300 DPI)

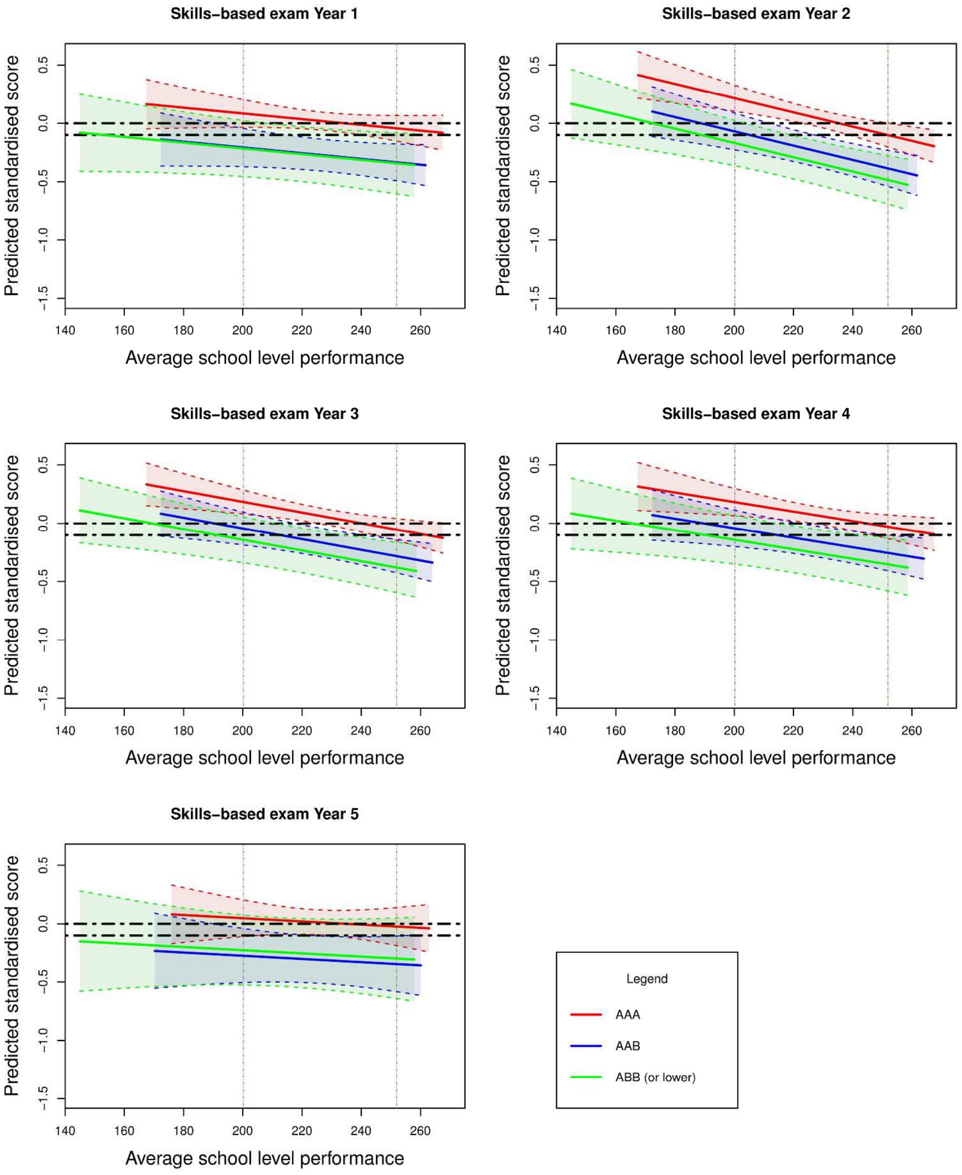

Figure 5

272x332mm (300 x 300 DPI)

Supplementary document to:

What is the effect of secondary (high) schooling on subsequent medical school performance? A national, UK-based, cohort study

Lazaro M. Mwandigha , Paul A. Tiffin, Lewis W. Paton, Adetayo S. Kasim, Jan R. Böhnke

For peer review only

Contents

1 Descriptive and Inferential statistics **2**

2 Estimation of Prior Education Attainment (PEA) **7**

3 Mediation analyses **9**
3.1 Single-level simple mediation analyses 9
3.2 Multi-level simple mediation analyses 10
3.3 Choosing between single-level and multi-level simple mediation analyses 16

4 Multi-level linear model **20**

5 Missing data **22**

6 Sensitivity analysis for missing data **24**
6.1 Single-level simple mediation analyses 25
6.2 Multi-level linear model 28

For peer review only - http://bmjopen.bmj.com/site/about/guidelines.xhtml

1 Descriptive and Inferential statistics

Figure 1 shows the distribution of the entrants total UKCAT scores across the 18 medical schools in the different universities. The distribution of the total UKCAT scores seem to differ widely. This may be partly explained by the fact that different medical schools use the UKCAT differently in the selection process. Some use the UKCAT as a "borderline method" (to discriminate amongst a small number of applicants lying at a decision borderline, who are otherwise indistinguishable on the medical school's other selection criteria), or "factor method" (an applicant's UKCAT score or a proxy for that score is added to the score the applicant obtains in the medical school's usual method of selection, to provide a total score), or "threshold method" (minimum or threshold UKCAT score is adopted to create a hurdle that an applicant must cross to reach the next stage in the selection process) or "rescue" (to compensate for an applicants who would otherwise be rejected on account of their score on other selection criteria) [4].

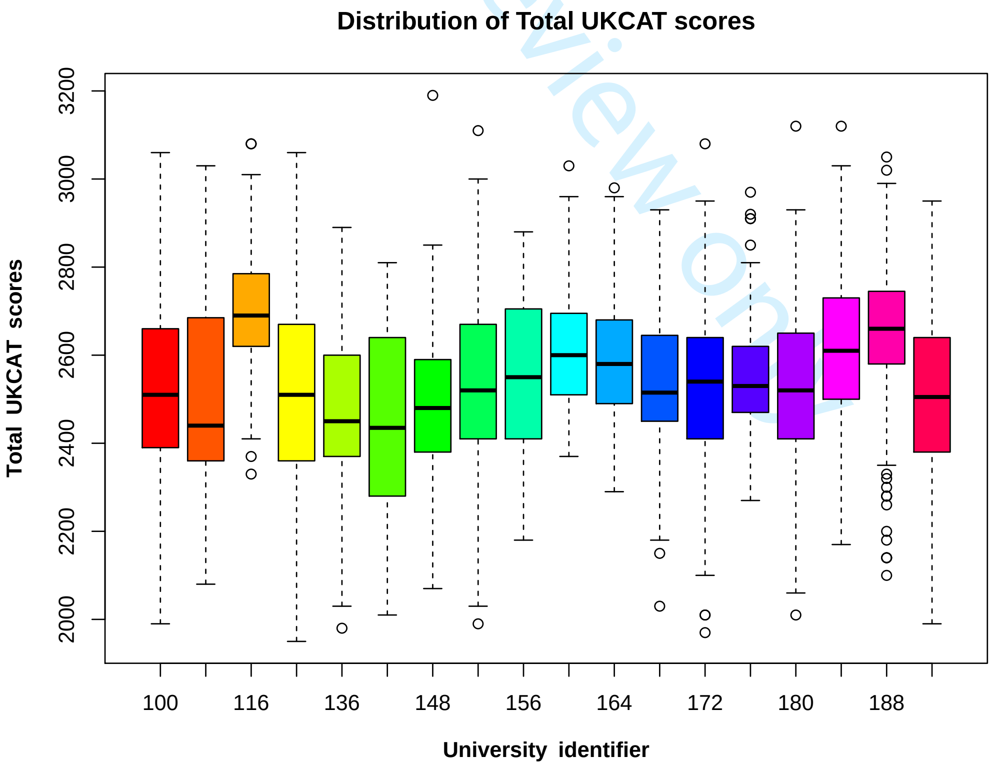

Figure 1: *Box plot of the distribution of the total UKCAT score for the different medical schools in UKCAT-consortium universities*

For peer review only - http://bmjopen.bmj.com/site/about/guidelines.xhtml

Table 1 shows the same information depicted in Figure 1 by means of summary statistics. To determine whether the distributional differences at university level may also be a factor of the quality of secondary school attended by a medical school entrant in a university, the total UKCAT matriculation scores were categorised into three (ranked) groups based on the standardised average performance of secondary schools attended by the entrants.

	University identifier	Mean	SD	Minimum	Maximum
1	100	2,513.06	200.45	1,990	3,060
2	108	2,498.00	246.81	2,080	3,030
3	116	2,690.69	150.22	2,330	3,080
4	120	2,506.16	213.26	1,950	3,060
5	136	2,463.36	186.42	1,980	2,890
6	144	2,448.33	197.10	2,010	2,810
7	148	2,485.37	170.25	2,070	3,190
8	152	2,521.22	219.61	1,990	3,110
9	156	2,550.00	183.80	2,180	2,880
10	160	2,610.18	136.20	2,370	3,030
11	164	2,590.61	149.13	2,290	2,980
12	168	2,524.74	169.51	2,030	2,930
13	172	2,519.26	195.10	1,970	3,080
14	176	2,552.64	120.39	2,270	2,970
15	180	2,522.39	178.94	2,010	3,120
16	184	2,615.40	175.59	2,170	3,120
17	188	2,643.50	177.32	2,100	3,050
18	192	2,502.67	190.36	1,990	2,950

Table 1: *Summary statistics of the total UKCAT score for the different medical schools in the 18 UKCAT-consortium universities*

For peer review only

Secondary schools were categorised into tertiles based on their on their standardised performance. Those with standardised performance of between [-2.5167, 0.3834), [0.3834, 1.2708) and [1.2708, 2.5875] were categorised into ranked groups 1, 2 and 3 respectively. The "[" and "]" indicate the limit is included in the group. The respective number of observations in the ranked groups were 622, 619 and 614 respectively. As may be observed from these values, the (ranked) groups had somewhat an equal number of ob-

For peer review only - http://bmjopen.bmj.com/site/about/guidelines.xhtml

servations. There were 252 observations that were ungrouped due to missing values in average school level performance. For each of the groups, the corresponding standardised UKCAT matriculation scores were examined. The distribution of the total UKCAT matriculation scores for the three ranked groups are shown in Figure 2. The lowest UKCAT performance was observed for entrants who attended secondary schools in group 1. The distribution of the total UKCAT matriculation scores in this group seemed differentiated from the other two groups. The secondary schools represented in group 2 and 3 did not seem differentiated from each other in terms of total UKCAT matriculation scores of medical school entrants who attended them.

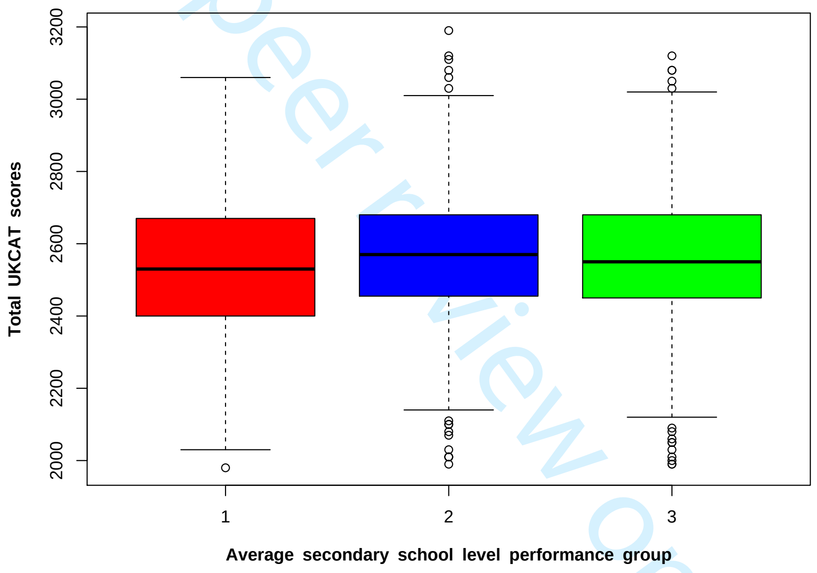

Figure 2: *Box plot of the distribution of the standardised total UKCAT score by category of rank of standardised average secondary school level performance*

To statistically confirm the trend observed in Figure 2, a one-way anova was conducted. The factor of interest was the group which had an ordered level of 1, 2 and 3 based on average secondary school level performance as already described. Following a statistically significant mean difference (p-value < 0.001) in the total UKCAT matriculation scores between the groups, the *Tukey's multiple group* comparison was conducted. This was done to determine the full extent and direction of the differences between the groups. Table 2 shows the results of this comparison which confirm the observed trend in Figure 2.

For peer review only - http://bmjopen.bmj.com/site/about/guidelines.xhtml

Compared to group 1, the total UKCAT matriculation scores were higher for entrants who attended secondary schools in groups 2 and 3. There was no evidence that total UKCAT matriculation scores differed for entrants who attended secondary schools in groups 2 and 3.

	Tukey's anova multiple group comparison			
Rank group	Difference	Lower 95% limit	Upper 95% limit	Adjusted p-value
2-1	43.1593	18.4357	67.8829	0.0001
3-1	34.4978	9.7238	59.2718	0.0032
3-2	-8.6615	-33.4653	16.1423	0.6912

Table 2: Total UKCAT score differences between groups based on the average secondary school level performance

Figure 3 shows the distribution of the undergraduate year one *knowledge*-based outcome scores prior to and after their standardisation within each of the university. Note that only 13 out of the 18 UKCAT consortium (medical schools) universities reported outcomes for knowledge-based exams in the first year. This is clearly seen from the number of box plots in the top and bottom panel with no corresponding standardisation in the bottom panel. The university identified with code 136 reported a single score of 63.61 hence the single line depicted instead of a box plot in the top panel. Universities identified with codes 148, 152, 164, 184 did not report any score for the undergraduate *knowledge*-based exam outcomes at the end of the first year of medical school training. Further, it may be said that standardisation does not affect the distribution of the *knowledge*-based outcome scores as the relative size of the box plots between universities remain the same before and after standardisation. Note that standardisation merely shifts the scale of comparison by allowing the different plots to have mean (value of approximately zero) that is similar across the different universities. Therefore, the underlying differences in the reported outcomes were modelled by a multi-level model (regardless of whether or not standardisation was done or not).

For peer review only - http://bmjopen.bmj.com/site/about/guidelines.xhtml

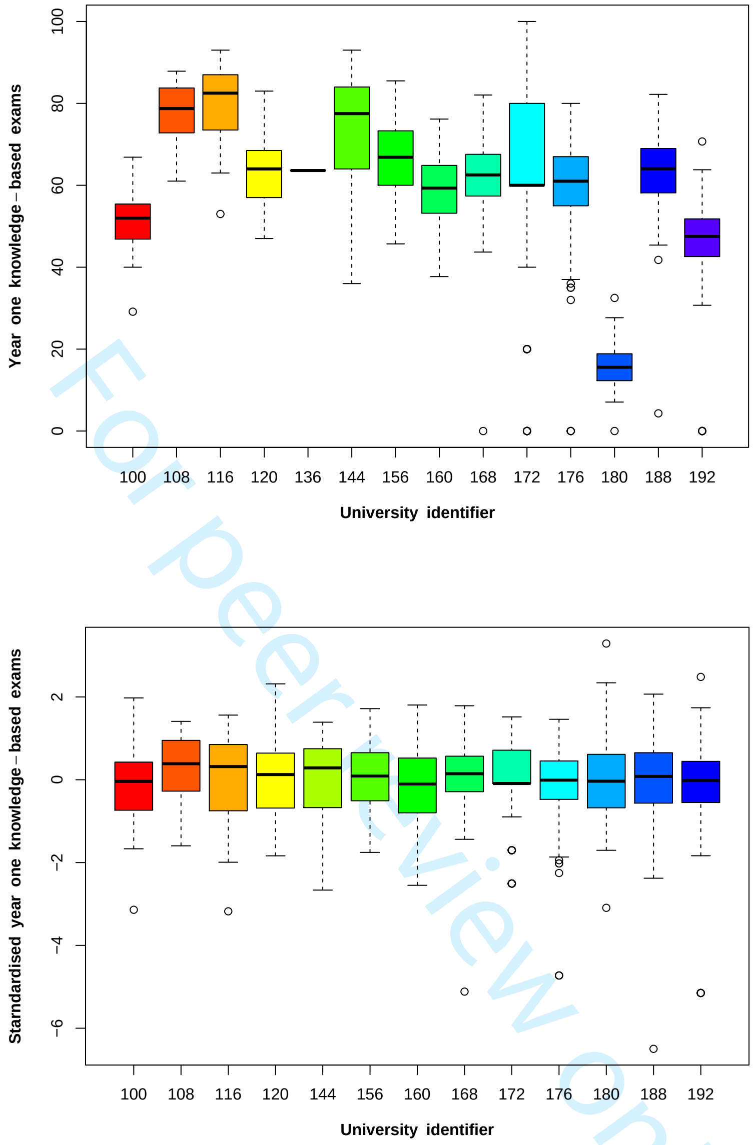

Figure 3: *Box plot of the distribution of end of year one knowledge-based outcomes for the different medical schools in UKCAT-consortium universities. The top panel and bottom panel shows the unstandardised and standardised undergraduate knowledge-based outcome scores respectively*

Table 3 shows the predictive validity of the total UKCAT score and PEA as estimated by bivariate Pearson correlation coefficients. Generally, the predictive validity of PEA was higher than that of the total UKCAT score. It was also observed that the predictive validity for the *knowledge*-based outcomes were higher than that for *skills*-based outcomes. The predictive validity of both the total UKCAT score and PEA was highest in the first two years of medical school training.

For peer review only - http://bmjopen.bmj.com/site/about/guidelines.xhtml

Predictor	Undergraduate knowledge-based outcome				
	Year one	Year two	Year three	Year four	Year five
PEA	0.25 (< 0.001)	0.23 (< 0.001)	0.23 (< 0.001)	0.18 (< 0.001)	0.22 (< 0.001)
total UKCAT score	0.11 (< 0.001)	0.11 (< 0.001)	0.15 (< 0.001)	0.11 (< 0.001)	0.16 (< 0.001)

Predictor	Undergraduate skills-based outcome				
	Year one	Year two	Year three	Year four	Year five
PEA	0.16 (< 0.001)	0.16 (< 0.001)	0.10 (< 0.001)	0.10 (0.0006)	0.13 (0.0012)
total UKCAT score	0.07 (0.0165)	0.06 (0.0238)	0.06 (0.0319)	0.07 (0.0164)	0.11 (0.0068)

Table 3: *Predictive validities of PEA and total UKCAT score for undergraduate medical school performance. The computed predictive validities are estimated by bivariate Pearson correlation coefficients from pairwise deleted data. The associated p-values for the reported validities are shown in brackets*

2 Estimation of Prior Education Attainment (PEA)

In order to obtain a single metric of scholastic (or academic) ability from the reported GCSEs and A Level exam scores, a novel approach described by *McManus et. al* [1] which involved conceptualising *educational achievement* as a latent variable was used. Thus PEA was estimated as a latent trait via an ordinal factor analysis using the most commonly taken A-level (both A1 and A2), and the grades obtained (e.g. A, B, C etc) used as (ordered categorical) indicators (see Table 4). The non-hierarchical version of McDonald's Omega was computed from the polychoric correlation matrix, since the factor analysis was of first order [2, 3]. The non-hierarchical McDonald's Omega was found to be 0.91. *Full Information Maximum Likelihood (FIML)* which maximizes use of the available data was used for the analysis to deal with missingness in the data (e.g. for the subjects not taken by a particular candidate). Subsequently, factor scores were then estimated for all applicants in the data, the results of the factor analysis from *Mplus* are displayed on Table 5. It was observed that generally, higher loadings were associated with Chemistry, Physics and Biology in GCSEs and A-Level (both A1 and A2) exams.

For peer review only

Exam	Subjects considered	Grade coding for factor analysis
GCSE	*Biology, Chemistry, Physics, Maths, French, History, Religious studies, Science, English, English literature* and *Geography*	C, D, E, F and G=1, B=2, A=3 and A^*=4
A Level (includes A1 and A2-level)	*Maths, Chemistry, Biology* and *Physics*	E and D=1 , C=2, B=3 and A=4

Table 4: *Coding of GCE A-Level and GCSE subjects for factor analysis*

Exam	Subject	Loading	Std Error	Estimate / Std. Error	Two sided p-value
GCSEs	Biology	0.805	0.009	93.988	0.000
	Chemistry	0.815	0.009	95.105	0.000
	English Literature	0.503	0.010	51.869	0.000
	English	0.572	0.009	62.060	0.000
	French	0.611	0.010	60.850	0.000
	Geography	0.696	0.011	60.710	0.000
	History	0.628	0.012	51.736	0.000
	Maths	0.693	0.008	90.998	0.000
	Physics	0.828	0.008	102.854	0.000
	Religious Education	0.510	0.012	43.155	0.000
	Science	0.749	0.049	15.233	0.000
A1-Level	Biology	0.861	0.005	171.013	0.000
	Chemistry	0.822	0.006	149.020	0.000
	Maths	0.798	0.009	93.642	0.000
	Physics	0.847	0.012	71.542	0.000
A2-Level	Biology	0.818	0.006	126.211	0.000
	Chemistry	0.798	0.007	121.959	0.000
	Maths	0.738	0.010	72.379	0.000
	Physics	0.836	0.010	86.867	0.000

Table 5: *Results from the factor analysis for the derivation of factor scores for PEA*

For peer review only - http://bmjopen.bmj.com/site/about/guidelines.xhtml

3 Mediation analyses

3.1 Single-level simple mediation analyses

It was aimed to *determine the extent to which an entrants PEA would mediate the predictive power of the UKCAT for two separate domains (knowledge and skills) over the period of undergraduate training*. To accomplish this a mediation model was considered. This is because, the overall *total predictive power of the UKCAT* for *knowledge* and *skills*-based undergraduate medical school exams would be partitioned into *direct* and *indirect* predictive power. This would then enable the accurate assessment of the relative, and unique, contribution UKCAT scores makes within the selection process. To demonstrate how this is done, consider Figure 4, which shows a *simple mediation model*. The term "simple" means that there is a one predictor, one mediator and one outcome variable under consideration.

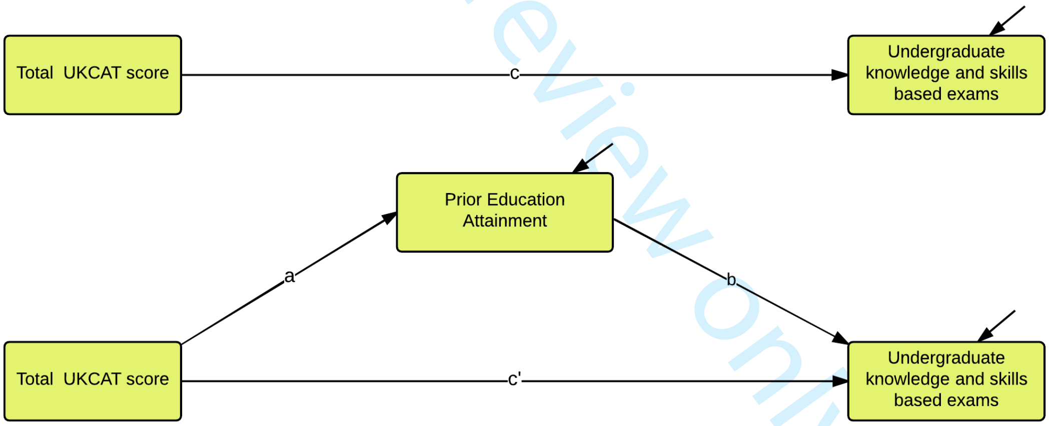

Figure 4: *Conceptual diagram of simple mediation model*

The effect denoted by *c* is the *total effect*, this may be easily obtained as a regression coefficient from a *Ordinary Least Squares (OLS)* regression model. The paths *b* and *c'* are *direct effects* for PEA and UKCAT respectively both of which may be obtained from a OLS regression model. For the purpose of the study, the paths of main interest were the *indirect effect*, product of the paths *a*b*, shown in equation 3.1. This *indirect effect* represents the non-unique contribution of the predictive power of the UKCAT. Further, a

For peer review only - http://bmjopen.bmj.com/site/about/guidelines.xhtml

proportion of this non-unique contribution, which is the portion of the predictive power of the UKCAT that is explained by PEA, may be expressed as $\frac{a*b}{c}$ (see Figure 3 in text of main paper) where c is the *total effect* which has been shown to be equal to sum of the *indirect* and *direct effects*

$$c = a*b + c' \tag{3.1}$$

The significance of the *indirect effect* may be obtained by testing the hypothesis $H_0 : a*b = 0$ versus $H_0 : a*b \neq 0$, traditionally, this was done by assuming a normal distribution for the *indirect effect* of $a*b$ thus necessitating the use of wald, score or likelihood ratio test with their corresponding p-value. This however, may lead to incorrect conclusions, when the *indirect effect* is not normally distributed as is often the case [5]. For this reason, most statistical software packages, such as Mplus implement a hypothesis test using a bootstrap approach which yields an empirical distribution for $a*b$. Similarly, it is possible for one to program this in any statistical software (e.g. R) by implementing a bootstrap or Monte Carlo simulation. The idea being the derivation of $(1-\alpha)100$ bootstrap or Monte Carlo percentile confidence intervals for the purpose of determining signficance. For SAS and SPSS users, macros have been developed for estimating the significance of the *indirect effect*, they include the *INDIRECT* and *PROCESS* macros which are based on the bootstrap while *MCMED* macro is based on Monte Carlo Simulation [6, 7].

3.2 Multi-level simple mediation analyses

The structure of the data used for the study was hierarchical (clustered) because the outcomes (*knowledge* and *skills*) considered in each year of undergraduate training were nested within the 18 universities. This means that fitting a simple mediation analysis which essentially ignored the hierarchical structure of the data would potentially result in *total, direct* and *indirect effects* with induced attenuations. This may then lead to biased conclusions. For this reason, a multi-level mediation model was considered. In a nutshell,

For peer review only - http://bmjopen.bmj.com/site/about/guidelines.xhtml

For peer review only

this model constitutes fitting a simple mediation for each cluster (university) separately and subsequently pooling the effects of interest together in some defined way to form *population average total, population average direct* and *population average indirect effects*. A conceptual representation of this model may be viewed on Figure 5.

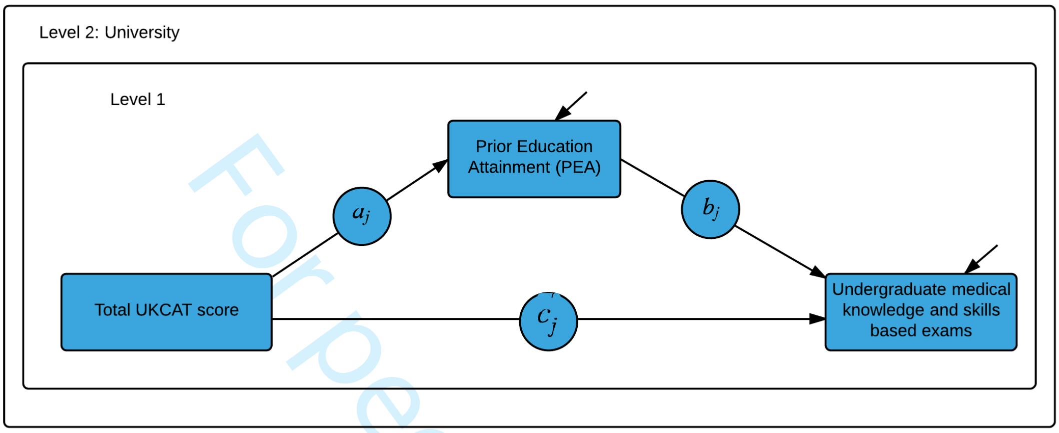

Figure 5: *Conceptual diagram of multi-level mediation model*

Note that, unlike in the case of the simple (single-level) mediation in Figure 4, the effects are now level-1 variables nested within university which is a level-2 variable. Further, all the effects are estimated as random rather than fixed effects thus allowing them to vary between the level-2 variables. This model is called the 1→ 1→ 1 mediation model since the predictor, UKCAT, the mediator, PEA, and the outcomes, *knowledge* and *skills*-based exams, all reside on level-1. In the conceptual representation of the model, the subscript *j* denotes that effects of interest vary between universities. These effects in the Figure are encircled to denote in *Structural Equation Modelling (SEM)* methodology that these effects are random [8]. The implementation of the 1→ 1→ 1 model is demonstrated for the *knowledge*-based exam scores (denoted by K) for brevity. The UKCAT and PEA scores are denoted by UKCAT and PEA respectively.

$$PEA_{ij} = d_{PEA_j} + a_j * UKCAT_{ij} + \varepsilon_{PEA_{ij}} \quad (3.2)$$

$$K_{ij} = d_{K_j} + b_j * PEA_{ij} + c'_j * UKCAT_{ij} + \varepsilon_{K_{ij}} \quad (3.3)$$

For peer review only - http://bmjopen.bmj.com/site/about/guidelines.xhtml

$$d_{PEA_j} = d_{PEA} + \mu_{d_{PEA_j}}$$
$$d_{K_j} = d_K + \mu_{K_j}$$
$$a_j = a + \mu_{a_j}$$
$$b_j = b + \mu_{b_j}$$
$$c'_j = c' + \mu_{c'_j} \quad (3.4)$$

The subscript i denotes a student and subscript j a particular university. Further, $\varepsilon_{PEA_{ij}}$ and $\varepsilon_{K_{ij}}$ are level-1 residuals for the mediator PEA and Knowledge based outcome of interest respectively. Finally, d_{PEA_j}, d_{K_j}, a_j, b_j and c'_j are the random intercepts and slopes of the models. The assumptions of the $1 \rightarrow 1 \rightarrow 1$ hierarchical mediation model are as follows

1. The predictor, $UKCAT_{ij}$ is uncorrelated with all the random effects (d_{PEA_j}, d_{K_j},a_j, b_j and c'_j) and the residuals ($\varepsilon_{PEA_{ij}}$ and $\varepsilon_{K_{ij}}$) in the model.
2. The residuals from the models, $\varepsilon_{PEA_{ij}}$ and $\varepsilon_{K_{ij}}$, are each normally distributed with an expected value of zero and are uncorrelated with one another.
3. The level-1 residuals, $\varepsilon_{PEA_{ij}}$ and $\varepsilon_{K_{ij}}$ are uncorrelated with random effects d_{PEA_j}, d_{K_j}, a_j, b_j and c'_j in the model.
4. The random effects are normally distributed with means equal to the average effects in the population. This may be expressed as,

$$E(a_j) = \bar{a}_j = a$$

$$E(b_j) = \bar{b}_j = b$$

and

$$E(c'_j) = \bar{c'}_j = c'$$

for the slopes of interest. Further, the random effects covary with one another.

5. The distributions of PEA_{ij} is normal conditional on $UKCAT_{ij}$ and K_{ij} normal conditional on PEA_{ij} and $UKCAT_{ij}$.

For peer review only - http://bmjopen.bmj.com/site/about/guidelines.xhtml

These assumptions lead to the following matrix formulation of the model. Note that, it is possible to estimate the *average of effects* (which may be referred to as *"population level effects"*, quantify the effects across all universities and their corresponding variabilities)

$$\begin{bmatrix} d_{PEA_j} \\ d_{K_j} \\ a_j \\ b_j \\ c_j^{'} \end{bmatrix} \sim N \left(\begin{bmatrix} d_{PEA} \\ d_K \\ a \\ b \\ c^{'} \end{bmatrix}, \begin{bmatrix} \sigma^2_{d_{PEA_j}} & & & & \\ \sigma_{d_{PEA_j K_j}} & \sigma^2_{K_j} & & & \\ \sigma_{d_{PEA_j a_j}} & \sigma_{K_j a_j} & \sigma^2_{a_j} & & \\ \sigma_{d_{PEA_j b_j}} & \sigma_{K_j b_j} & \sigma_{a_j b_j} & \sigma^2_{b_j} & \\ \sigma_{d_{PEA_j c_j^{'}}} & \sigma_{K_j c_j^{'}} & \sigma_{a_j c_j^{'}} & \sigma_{b_j c_j^{'}} & \sigma^{'}_{c_j} \end{bmatrix} \right)$$

For peer review only

The *average mediation (indirect) effect* and *average total* effects may then be estimated by making use of equations 3.5 and 3.6 respectively.

$$E(a_j * b_j) = a * b + \sigma_{a_j b_j} \tag{3.5}$$

$$E(a_j * b_j + c^{'}) = a * b + \sigma_{a_j b_j} + c^{'} \tag{3.6}$$

The multi-level simple mediation model was fitted in Mplus and the estimates of *average total, average indirect* and *average direct effects* estimated from equations 3.5 and 3.6. The significance of the *average total* and *average direct effects* were obtained from the results in Mplus. To determine the significance of *average indirect effect*, a Monte Carlo 95% Percentile CI was programmed in R software by sampling 10,000 observations from the distribution in equation 3.7.

$$N \left(\begin{bmatrix} a \\ b \\ \sigma_{a_j b_j} \end{bmatrix}, \begin{bmatrix} \sigma^2_a & \sigma_{ab} & \sigma_{a,\sigma_{a_j b_j}} \\ & \sigma^2_b & \sigma_{b,\sigma_{a_j b_j}} \\ & & \sigma^2_{\sigma_{a_j b_j}} \end{bmatrix} \right) \tag{3.7}$$

The individual elements of the distribution in equation 3.7 were obtained from the results of the multi-level mediation model in Mplus using the *TECH 3* output command. Each of the 10,000 observations sampled for a, b and $\sigma_{a_j b_j}$ were plugged into equations 3.5 and 3.6 to obtain 10,000 *average indirect effect* values. Subsequently, the Monte Carlo 95%

Percentile CI was calculated by taking the 2.5^{th} and 97.5^{th} percentile of the empirical distribution of the 10,000 estimates for *indirect effect*. Figure 6 shows the plotted results from the models. It was observed that there were statistical significant *average indirect effects* in the first four years of undergraduate training of medical school for both *knowledge* and *skills*-based exams outcomes. The *indirect effects* represent the contribution of PEA towards the predictive power of the UKCAT. It was also observed that the range of the CIs widened in the third year onwards which is indicative of the missingness observed in the later years of the study (see Figure 1 and Table 1 in main text of the paper) which led to little information available for analysis in each of the university clusters in the data.

For peer review only

For peer review only - http://bmjopen.bmj.com/site/about/guidelines.xhtml

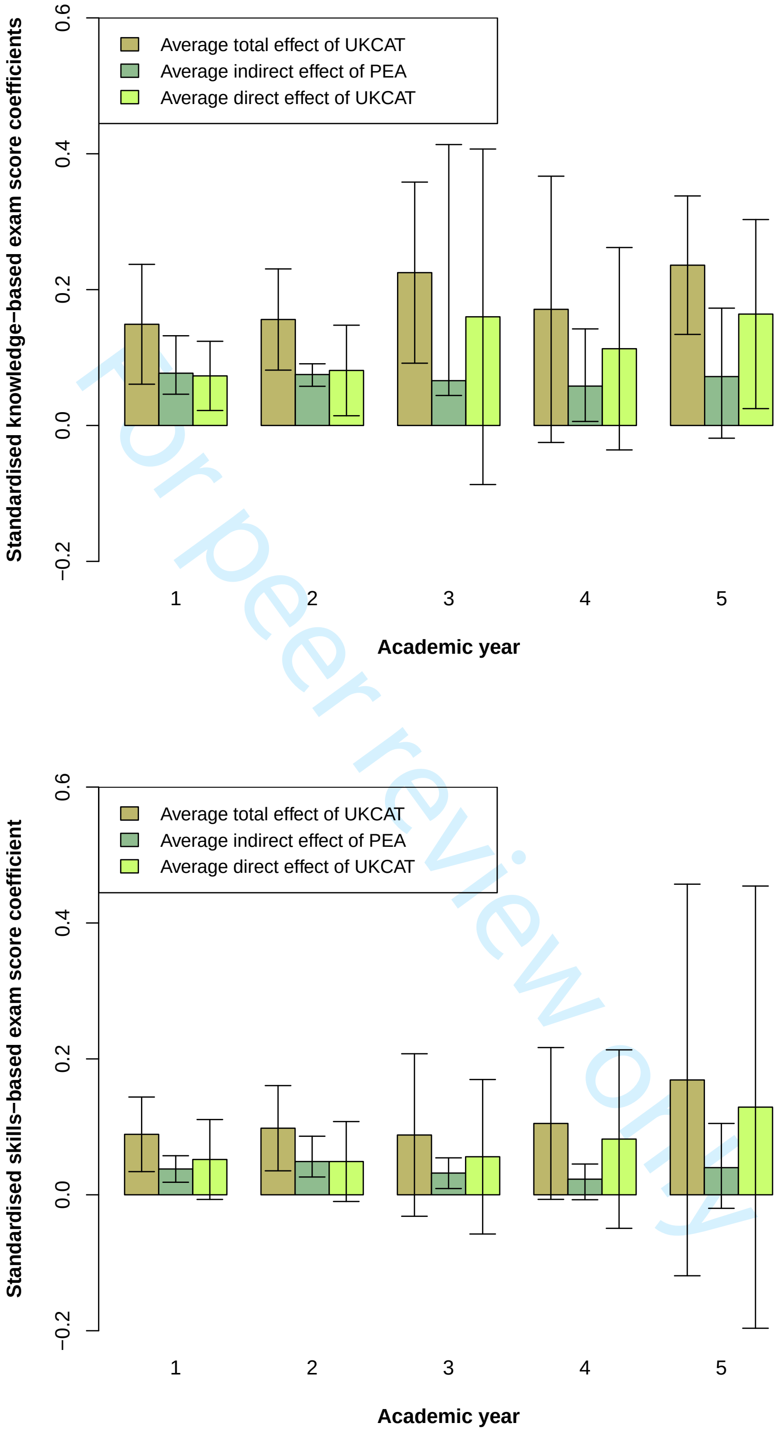

Figure 6: *Knowledge and skills-based multi-level mediation results for the average total, average direct and average indirect effects with respective 95% CI for average total and average direct effects computed from point estimates and standard errors obtained in Mplus and 95% Monte Carlo CI computed in R through simulation*

For peer review only - http://bmjopen.bmj.com/site/about/guidelines.xhtml

3.3 Choosing between single-level and multi-level simple mediation analyses

The multi-level mediation model fitted in section 3.2 is prone to convergence difficulties and is highly susceptible to missing data related problems. For instances where there are high attrition rates in later years of a longitudinal cohort study, it is highly likely that some or most of the clusters may have little or no data to contribute meaningfully to the analysis and this may further risk a lack of convergence. Therefore, for a given estimation problem, a single-level mediation model is preferred if there is evidence that there are no statistically significant clustering effects in the data.

To determine whether there were statistically significant clustering effects in the data equations 3.5 and 3.6 were considered. Note that from equation 3.5, when $\sigma_{a_j b_j} = 0$, the resulting *average indirect effect* is equal to what would be estimated in a single-level simple mediation analysis in section 3.1. Therefore in seeking to determine whether a single or multi-level mediation analysis should be fitted to the data, it will be sufficient to test the hypothesis, $H_0 : \sigma_{a_j b_j} = 0$ versus $H_1 : \sigma_{a_j b_j} \neq 0$. Evidence in favour of the null hypothesis would also be evidence in favour of a simple single-level mediation analysis. The results of the hypothesis test were available as part of the multi-level results in Mplus and are displayed on Table 6. It was observed that all of the p-values were > 0.05 implying that there were statistically non-significant clustering effects in the data. Further, *Intra Cluster Correlations (ICCs)* for the models computed by utilising the main diagonal of the covariance matrix from equation 3.7 and the residual variances from the model are displayed on Table 7. The observed ICCs (7th and 13th column of the Table) indicate that the proportion of variability explained by the multi-level mediation models is negligible. Therefore a simple single-level mediation model is appropriate for the data.

Following the results on Tables 6 and 7, a simple single-level mediation model was fitted using two models, for the case of *knowledge*-based exams outcomes, shown in equation

For peer review only - http://bmjopen.bmj.com/site/about/guidelines.xhtml

(3.9) and (3.8) respectively using the same notation as in section 3.2.

$$PEA_i = I_{PEA} + a * UKCAT_i + \varepsilon_{PEA} \tag{3.8}$$

$$K_i = I_K + c^{'} * UKCAT_i + b * PEA_i + \varepsilon_K \tag{3.9}$$

Academic year	Knowledge- based exams			Skills-based exams		
	$\sigma_{a_j b_j}$	Std. Error	P-value	$\sigma_{a_j b_j}$	Std. Error	P-value
1	-0.007	0.016	0.663	-0.006	0.007	0.414
2	-0.004	0.003	0.284	-0.005	0.012	0.673
3	-0.002	0.066	0.972	0.000	0.003	0.888
4	-0.001	0.006	0.872	-0.004	0.013	0.778
5	0.000	0.031	0.992	-0.001	-0.009	0.951

Table 6: *Results of the hypothesis testing for the statistical significance of $\sigma_{a_j b_j}$ from Mplus*

Academic year	Knowledge-based exams					
	σ^2_a	σ^2_b	σ^2_{res}	σ^2_{PEA}	$\sigma^2_{\sigma_{a_j b_j}}$	$\frac{\sigma^2_{\sigma_{a_j b_j}}}{(\sigma^2_a+\sigma^2_b+\sigma^2_{res}+\sigma^2_{PEA}+\sigma^2_{\sigma_{a_j b_j}})}$
1	0.006	0.003	0.848	1.876	0.000	0.000
2	0.002	0.000	0.894	1.875	0.000	0.000
3	0.093	0.034	0.841	1.875	0.004	0.002
4	0.005	0.006	0.890	1.875	0.000	0.000
5	0.004	0.002	0.902	1.876	0.001	0.000

Academic year	Skills-based exams					
	σ^2_a	σ^2_b	σ^2_{res}	σ^2_{PEA}	$\sigma^2_{\sigma_{a_j b_j}}$	$\frac{\sigma^2_{\sigma_{a_j b_j}}}{(\sigma^2_a+\sigma^2_b+\sigma^2_{res}+\sigma^2_{PEA}+\sigma^2_{\sigma_{a_j b_j}})}$
1	0.003	0.001	0.888	1.877	0.000	0.000
2	0.011	0.001	0.968	1.876	0.000	0.000
3	0.003	0.001	0.947	1.876	0.000	0.000
4	0.012	0.000	0.893	1.876	0.000	0.000
5	0.003	0.006	0.985	1.877	0.000	0.000

Table 7: *Intra Cluster Correlations (ICC) for knowledge and skills-based exam outcomes for the five years of undergraduate medical school training*

For peer review only - http://bmjopen.bmj.com/site/about/guidelines.xhtml

The mediator of interest is *PEA* while *UKCAT* and *K* are predictor and outcome of interest respectively. The *I* denotes the intercept while *a,b* and *c* are the regression coefficients to be estimated. This model was fitted both in Mplus and in SAS. The results of the models from the two software packages were expectedly similar. The statistical significance was tested using the bootstrap approach implemented in Mplus and Monte Carlo simulation in SAS using the *MCMED macro for SAS* [6]. In both Mplus and SAS, the 95% confidence intervals were obtained by taking the 2.5^{th} and 97.5^{th} percentiles of the empirical distribution for $a*b$ from 10,000 sampled observations. The single-level simple mediation model results (from SAS, similar to results from Mplus) are shown in Tables 8 and 9 for *knowledge* and *skills*-based exams outcomes respectively. For both *knowledge* and *skills*-based outcomes, in all undergraduate years, there were statistically significant *indirect effects* of UKCAT through PEA. This means that the predictive power of the UKCAT for undergraduate medical school performance can be partially explained by PEA.

	Knowledge-based exams					
	Direct effect		Indirect effect		Total effect	
Academic year	Estimate	95% CI	Estimate	95% Monte Carlo CI	Estimate	95% CI
1	0.071	(0.002, 0.139)	0.081	(0.059, 0.106)	0.151	(0.083, 0.220)
2	0.073	(0.002, 0.144)	0.074	(0.053, 0.010)	0.147	(0.077 0.217)
3	0.127	(0.058, 0.195)	0.069	(0.049, 0.094)	0.196	(0.129, 0.263)
4	0.086	(0.014, 0.159)	0.062	(0.040, 0.085)	0.148	(0.078, 0.218)
5	0.162	(0.058, 0.266)	0.052	(0.027, 0.087)	0.213	(0.109, 0.318)

Table 8: *Results of the single-level simple mediation model for the undergraduate knowledge-based exam outcome*

For peer review only - http://bmjopen.bmj.com/site/about/guidelines.xhtml

Academic year	Skills-based exams					
	Direct effect		Indirect effect		Total effect	
	Estimate	95% CI	Estimate	95% Monte Carlo CI	Estimate	95% CI
1	0.056	(-0.028, 0.140)	0.045	(0.026, 0.070)	0.101	(0.019, 0.184)
2	0.032	(-0.049, 0.113)	0.059	(0.038, 0.085)	0.091	(0.012, 0.170)
3	0.048	(-0.026, 0.122)	0.031	(0.012, 0.052)	0.078	(0.007, 0.150)
4	0.062	(-0.017, 0.141)	0.032	(0.012, 0.055)	0.094	(0.017, 0.170)
5	0.121	(0.010, 0.232)	0.030	(0.009, 0.063)	0.151	(0.042, 0.261)

Table 9: *Results of the single-level simple mediation model for the undergraduate skills-based exam outcome*

For peer review only

The results from Tables 8 and 9 were used to compute the proportions of the total UKCAT scores explained by the PEA shown in Figure 3 of main manuscript. To determine whether the proportion of total UKCAT scores explained by the PEA in each year of medical school training varied by outcome, a statistical test for the significance of the difference between the proportions was conducted as shown in equation 3.10 in each year of medical school training. The subscripts k and s denote *knowledge* and *skills*-based exams outcomes respectively. The term p denotes the proportion of the total UKCAT scores explained by the PEA. Table 10 shows the results of the statistical test conducted. It was observed that there were statistically significant differences in the proportions of the total UKCAT scores explained by the PEA between the *knowledge* and *skills*-based exams outcomes in all but the fifth year of medical training. It is was also observed that the fifth year of medical school training had very low sample sizes for the two outcomes under consideration. This contributed to a lack of sufficient power to detect differences in the proportions in that year.

$$Z = \frac{(p_k - p_s) - 0}{\sqrt{(\frac{p_k(1-p_k)}{n_k} + \frac{p_s(1-p_s)}{n_s})}} \tag{3.10}$$

For peer review only - http://bmjopen.bmj.com/site/about/guidelines.xhtml

Year	Undergraduate knowledge-based exams		Undergraduate skills-based exams		Results		
	Proportion ($p_k = \frac{a*b}{c}$)	Sample size	Proportion ($p_s = \frac{a*b}{c}$)	Sample size	$p_k - p_s$	Z	P-value
1	0.5331	1,453	0.4455	1,051	0.0875	4.3419	< 0.0001
2	0.5054	1,418	0.6443	1,233	-0.1388	-7.2948	< 0.0001
3	0.3541	1,348	0.3916	1,238	-0.0375	-1.9707	0.0488
4	0.4164	1,349	0.3369	1,072	0.0795	4.0325	0.0001
5	0.2423	626	0.2003	576	0.0420	1.7573	0.0789

Table 10: *Statistical test for the significance of the difference in the proportion of UKCAT explained by PEA between the undergraduate knowledge and skills-based exam outcomes. The p-values are estimated from a standard normal distribution*

4 Multi-level linear model

To address the second aim of the study, which was *to appraise the influence of the performance of the previous secondary school attended on an undergraduates achievement in medical school*, a multi-level linear model or *Linear Mixed Model (LMM)* was used due to its capability to handle clustering in instances where the outcomes are continuous and correlated. The term "mixed" in the Linear Mixed Model comes from the fact that the model estimates both fixed (mean structure) and random effects (random structure). The modelling framework of Linear Mixed Model may be expressed as follows:

$$Y_i = X_i\beta + Z_i b_i + \varepsilon_i \tag{4.1}$$

where

$$b_i \sim N(0, D)$$
$$\varepsilon_i \sim N(0, \Sigma_i)$$

with $b_1 \ldots b_N$ and $\varepsilon_1 \ldots \varepsilon_N$ being independent. Y_i is the n_i-dimensional outcome (*knowledge* or *skills*-based exams), X_i and Z_i are the design matrices for the fixed and random effects of known predictors respectively, β and b_i are fixed and university specific effects respectively, and ε_i is the vector containing the residual components [9]. X_i is a design

For peer review only - http://bmjopen.bmj.com/site/about/guidelines.xhtml

matrix containing the predictors; average school level performance of the school in which an entrant sat for their A-level exam, an entrant's reported A-level grade (AAA, AAB, ABB, BBB or BBC), interaction between average school level performance and reported A-level grades and the tier of an entrant's secondary school as categorised based on their performance (see Figure 2). Z_i is a design matrix containing a random intercept which modelled the correlation in the outcomes within a university by allowing the (predicted) outcomes to vary between universities.

As seen in Table 11, the effect of the secondary school group (ordered based on their performance as 1, 2 or 3) was not statistically significant. This implies the A-level grades earned by an medical school entrant and the average level performance of secondary school attended are sufficient in explaining the undergraduate medical school outcomes. Further categorisation of secondary schools based on their performance adds no value in explaining undergraduate medical school outcomes. Therefore the proposed model fitted was in line with the predictors shown in Table 12.

	P-values for undergraduate knowledge-based outcome					P-values for undergraduate skills-based outcome				
Predictor	Year one	Year two	Year three	Year four	Year five	Year one	Year two	Year three	Year four	Year five
SSLP	0.0239	0.1939	0.2100	0.4393	0.2284	0.5688	0.0324	0.2272	0.9608	0.5137
A-Level grade	< 0.0001	< 0.0001	< 0.0001	< 0.0001	< 0.0001	0.0002	< 0.0001	0.0002	0.0007	0.0124
SSLP group	0.9551	0.7273	0.5640	0.9078	0.9864	0.9072	0.8393	0.9798	0.3546	0.2564

Table 11: *Results of the multi-level model showing the type 3 tests p-values (Pr. $>$ F) for the predictors of undergraduate knowledge and skills-based outcomes for each of the year of medical school. SSLP is the average Secondary School Level Performance and SSLP group is the three tier categorisation of secondary schools based on their reported average performance*

	P-values for undergraduate knowledge-based outcome					P-values for undergraduate skills-based outcome				
Predictor	Year one	Year two	Year three	Year four	Year five	Year one	Year two	Year three	Year four	Year five
SSLP	< 0.0001	< 0.0001	< 0.0001	< 0.0001	0.0029	0.1403	< 0.0001	0.0014	0.0099	0.5596
A-Level grade	< 0.0001	< 0.0001	< 0.0001	< 0.0001	< 0.0001	0.0002	< 0.0001	0.0002	0.0008	0.0109

Table 12: *Results of the multi-level model showing the type 3 tests p-values (Pr. $>$ F) for the predictors of undergraduate knowledge and skills-based outcomes for each of the year of medical schoo. Only SSLP and A-level grades were been retained in the model. No interaction between SSLP and A-level grades was detected*

For peer review only - http://bmjopen.bmj.com/site/about/guidelines.xhtml

5 Missing data

The missing data patterns for *knowledge*-based outcomes are shown in Table 13, it was observed that only 20.84% of the entrants had complete data for the *knowledge*-based exam outcome throughout the five years of medical school. Monotone pattern of missingness accounted for 47.36% of the missingness data patterns. The most frequently occurring monotone pattern of missingness had outcome data only for year one to year three. On the other hand, the most frequently occurring arbitrary (non-monotone) pattern of missingness had outcome data missing for year one, two and five.

Table 14 shows the pattern of missingness for *skills*-based exam outcome. About 17% of the entrants had complete data for the outcome over the course of the study duration while 41.29% of the data had monotone pattern of missingness. The most occurring monotone pattern of missingness had outcome data missing for year five. The most occurring arbitrary missingness pattern compromising of about 9.5% of the arbitrary missingess pattern was for year one, two and five.

For peer review only

Outcome						Count	%
Group	Year 1	Year 2	Year 3	Year 4	Year 5		
Complete							
1	O	O	O	O	O	439	20.84
Monotone missingness							
2	O	O	O	O	M	272	12.91
3	O	O	O	M	M	330	15.66
4	O	O	M	M	M	199	9.44
5	O	M	M	M	M	42	1.99
6	M	M	M	M	M	155	7.36
Arbitrary missingness							
7	O	M	O	M	M	7	0.33
8	O	O	M	O	O	50	2.37
9	O	O	M	O	M	114	5.41
10	M	O	O	O	M	7	0.33
11	O	M	O	M	M	3	0.14
12	M	O	M	M	M	4	0.19
13	M	M	O	O	M	274	13
14	M	M	O	M	M	16	0.76
15	M	M	M	O	O	135	6.41
16	M	M	M	O	M	58	2.75
17	M	M	M	M	O	2	0.09
Total						2,107	100

Table 13: *Missingness data patterns for the undergraduate knowledge-based scores for the 2,107 entrants who sat for the UKCAT in 2007. Each "O" and "M" represents each instance where data are present and absent respectively (i.e. the first row represents the proportion of cases with no missing data). Patterns are categorised as either monotone (i.e. where data relating to all subsequent years are missing after the initial missing data year) or arbitrary (i.e. non-monotone)*

For peer review only

Outcome						Count	%
Group	Year 1	Year 2	Year 3	Year 4	Year 5		
Complete							
1	O	O	O	O	O	360	17.09
Monotone missingness							
2	O	O	O	O	M	308	14.62
3	O	O	O	M	M	61	2.9
4	O	O	M	M	M	199	9.44
5	O	M	M	M	M	26	1.23
6	M	M	M	M	M	276	13.1
Arbitrary missingness							
7	O	O	M	O	M	91	4.32
8	O	M	O	M	M	6	0.28
9	M	O	O	O	M	37	1.76
10	O	M	M	O	O	37	1.76
11	M	O	M	M	M	140	6.64
12	M	M	O	O	O	79	3.75
13	M	M	O	O	M	197	9.35
14	M	M	O	M	M	153	7.26
15	M	M	M	M	O	137	6.5
Total						2,107	100

Table 14: *Missingness patterns for the undergraduate skills-based scores for the 2,107 entrants who sat for the UKCAT in 2007. Each "O" and "M" represents each instance where data are present and absent respectively (i.e. the first row represents the proportion of cases with no missing data). Patterns are categorised as either monotone (i.e. where data relating to all subsequent years are missing after the initial missing data year) or arbitrary (i.e. non-monotone)*

6 Sensitivity analysis for missing data

Sensitivity analysis was conducted to determine to what extent the missingness in the data influenced the results of the study. The data analysis for the study assumed *Missing At Random (MAR)* mechanism. The MAR assumption was invoked by making use of *ignorability* which entailed ignoring the missingness process. The purpose of the sensitivity analysis was to investigate whether this assumption was justifiable. This involved refitting the models with multiply imputed data and comparing the results from these models with those fitted previously under ingnorability. The premise being, if ignorability is valid under MAR, and *Multiple Imputation (MI)* which is also valid under MAR, then the results under both should be similar. When this is the case, the assumption of ignorability

For peer review only - http://bmjopen.bmj.com/site/about/guidelines.xhtml

For peer review only

and MAR would be justified.

6.1 Single-level simple mediation analyses

For the single-level simple mediation analysis, the models were fitted after imputation was conducted 30 times thus creating 30 datasets. These datasets were analysed and results later summarised through pooling of the estimates. The computation of associated standard errors of their estimates was also done. The MI was conducted in SAS using the *Monte Carlo Markov Chain (MCMC)* which imputes the missing values in the data in a way that retains the overall mean and covariate structure of the data assuming a joint multivariate normal distribution [10, 11]. The results of the previous non-imputed data displayed in Table 8 and 9 for both *knowledge* and *skills*-based exams are further displayed in graphical form in Figure 7. These were compared to the results from the multiply imputed data which are found on Figure 8. It was observed that in as far as the aim of the analysis was concerned, there were no discernible difference in the estimates and conclusions regarding the *indirect effects* of UKCAT through PEA for both the *knowledge* and *skills*-based outcomes from both the multiply imputed and non-imputed data. This implies that the assumptions of ignorability and MAR were plausible and that the missingness though severe in later years of the study, did not adversely effect the results and conclusions of the statistical analysis. This is expected as the missing data was created when participating medical schools failed to submit outcome data the UKCAT database in a that particular year. Thus, it may be concluded that the missing data was unlikely to threaten the validity of the inferences drawn from the results.

For peer review only - http://bmjopen.bmj.com/site/about/guidelines.xhtml

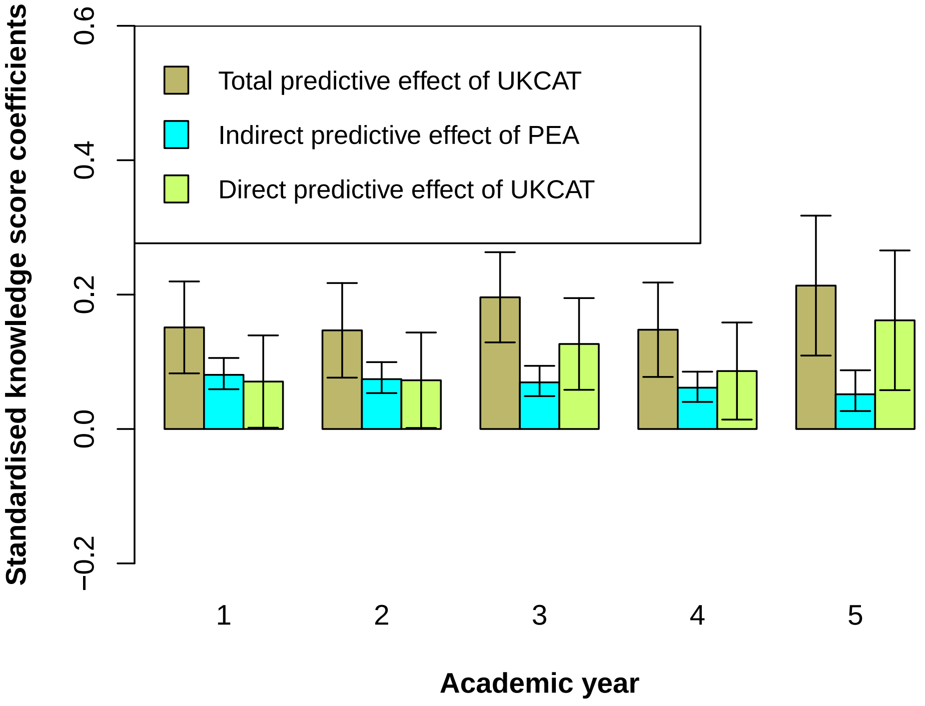

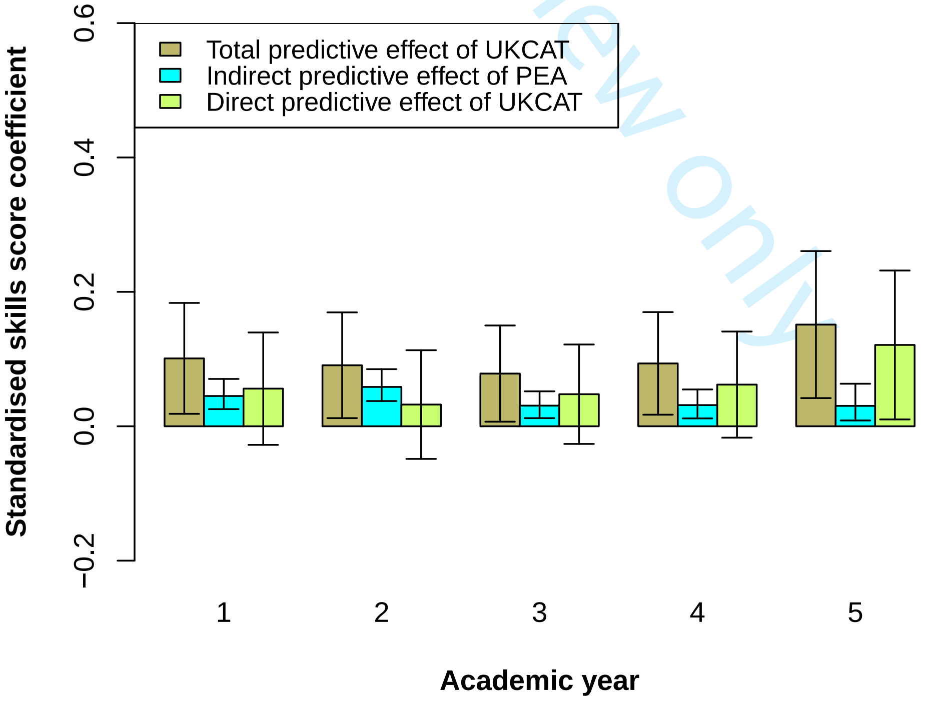

Figure 7: *Results of the single-level simple mediation analysis based on non-imputed data for undergraduate medical school knowledge and skills-based outcomes*

For peer review only - http://bmjopen.bmj.com/site/about/guidelines.xhtml

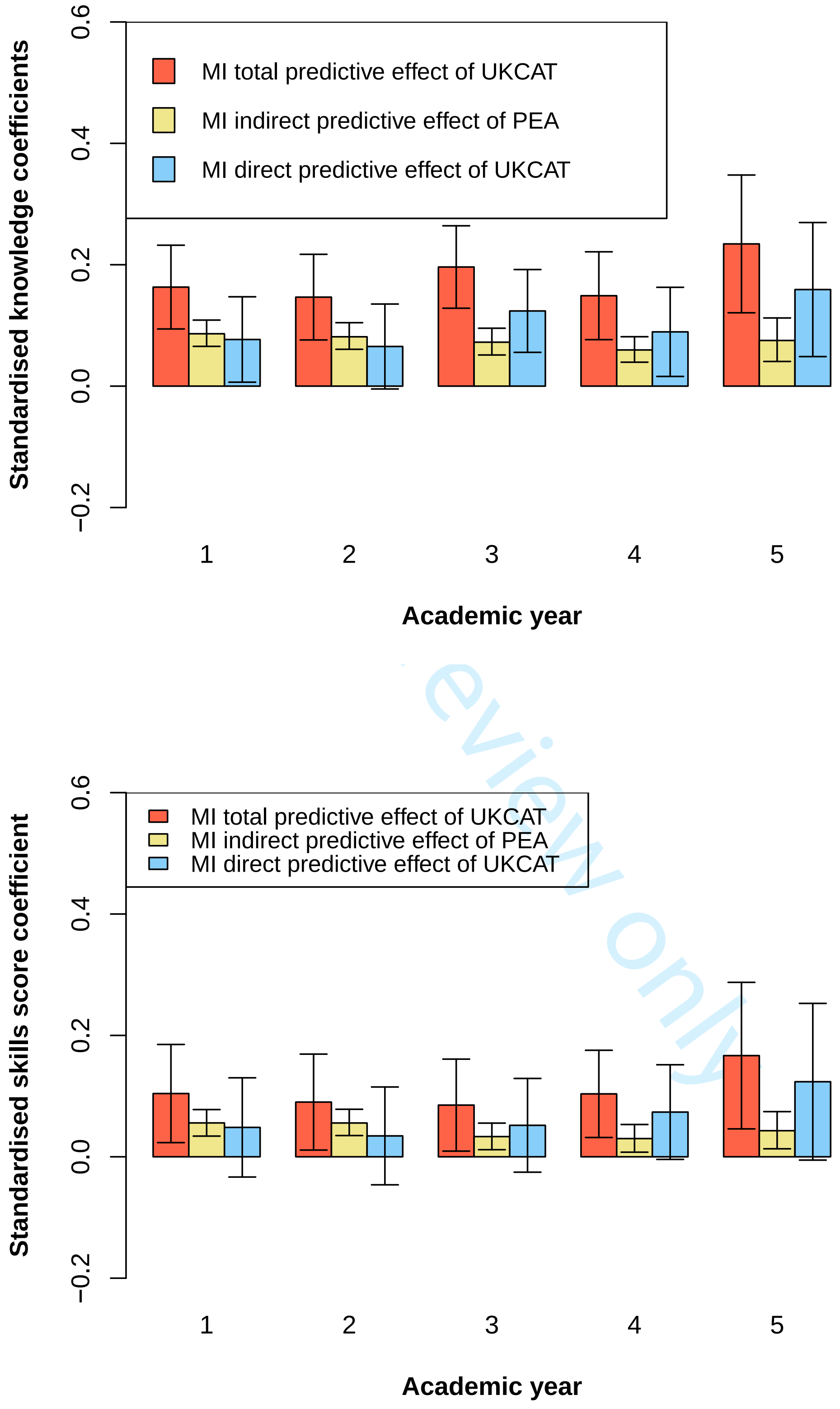

Figure 8: *Results of the single-level simple mediation analysis based on 30 MI data for undergraduate medical school knowledge and skills-based outcomes*

For peer review only - http://bmjopen.bmj.com/site/about/guidelines.xhtml

6.2 Multi-level linear model

Figure 9 and 10 show the plots of MI results for the model investigating the effect of average school level performance by reported grades on *knowledge* and *skills*-based exam outcomes for all five years of undergraduate medical school. All the variables of interest, that is, *knowledge* and *skills*-based undergraduate medical exam outcomes, average school level performance and PEA grades were affected by missingness. MI was conducted using *Multiple Imputations by Chain Equations (MICE)*, a MCMC based imputation technique that makes use of a collection of univariate conditional distributions of the variables with missing values given the other variables present in the data [10]. The number of imputations , M, was initially set at 5 and increased by multiples of 5 until a value of M that would yield unchanging results for the model described in section 4. The parameter estimates obtained were the same for $M >= 10$ indicating that any choice of $M >= 10$ was optimal. For comparison with results from the original data, M=15 was used.

The results from MI data were compared to those from the original data shown in Figures 4 and 5 in the main text of the paper for both *knowledge* and *skills*-based exams outcomes. The comparison revealed that the missingness did not an adverse effect on the analysis. Like in the original unimputed data, for both *knowledge* and *skills*-based exam outcomes, at each level of average school level performance students with higher grades tend to perform better compared to their counterparts with lower grades throughout undergraduate medical school. Overall, compared to students from schools with high average school level performance, students from schools with low average school level performance tend to have better scores in both *knowledge* and *skills*-based exam outcomes throughout undergraduate medical school. This suggests that the assumption of MAR invoked for the study was plausible.

For peer review only - http://bmjopen.bmj.com/site/about/guidelines.xhtml

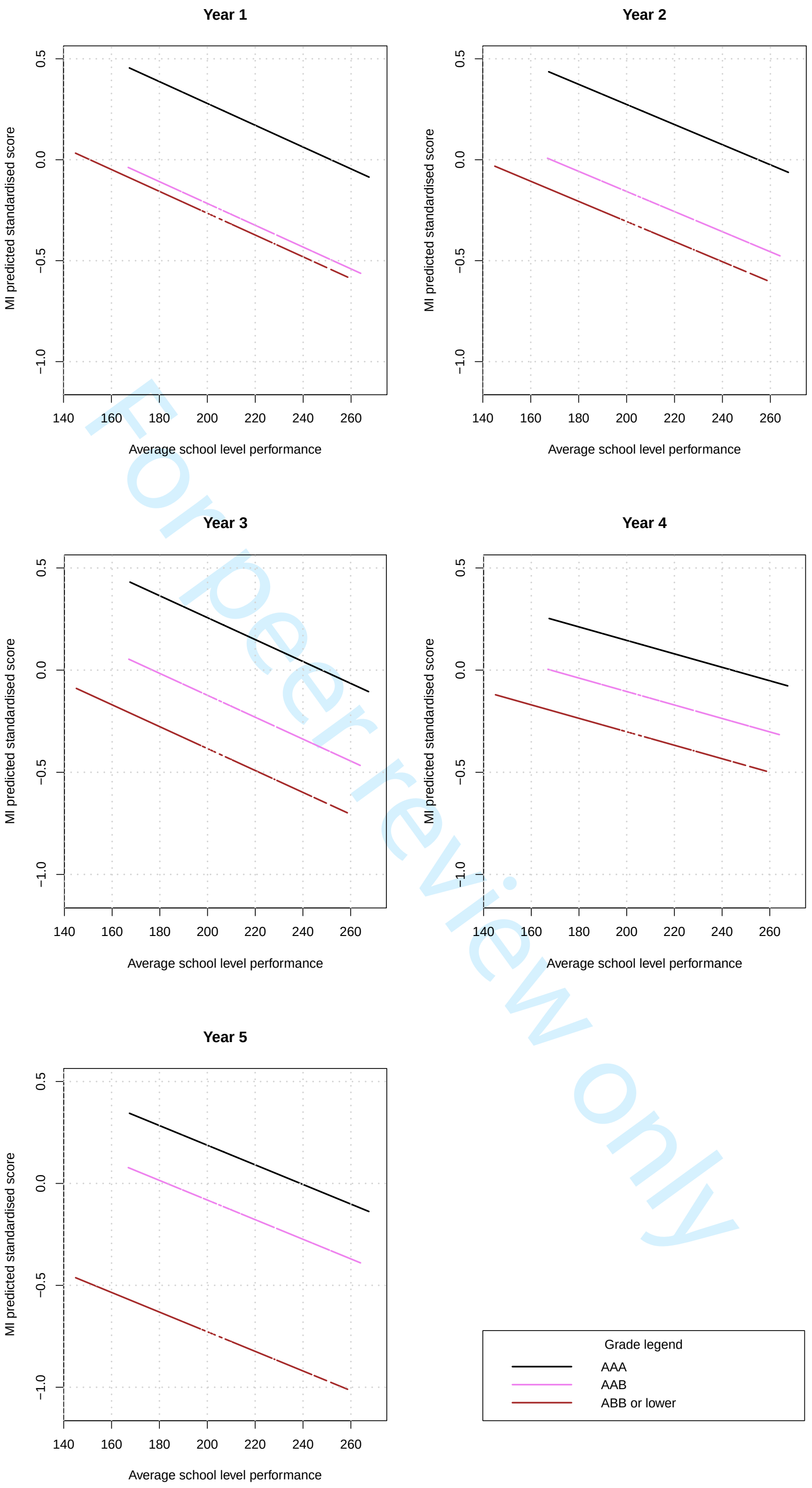

For peer review only

Figure 9: *Multiply imputed effect of average school level performance by reported grades on undergraduate medical school knowledge-based exams*

For peer review only - http://bmjopen.bmj.com/site/about/guidelines.xhtml

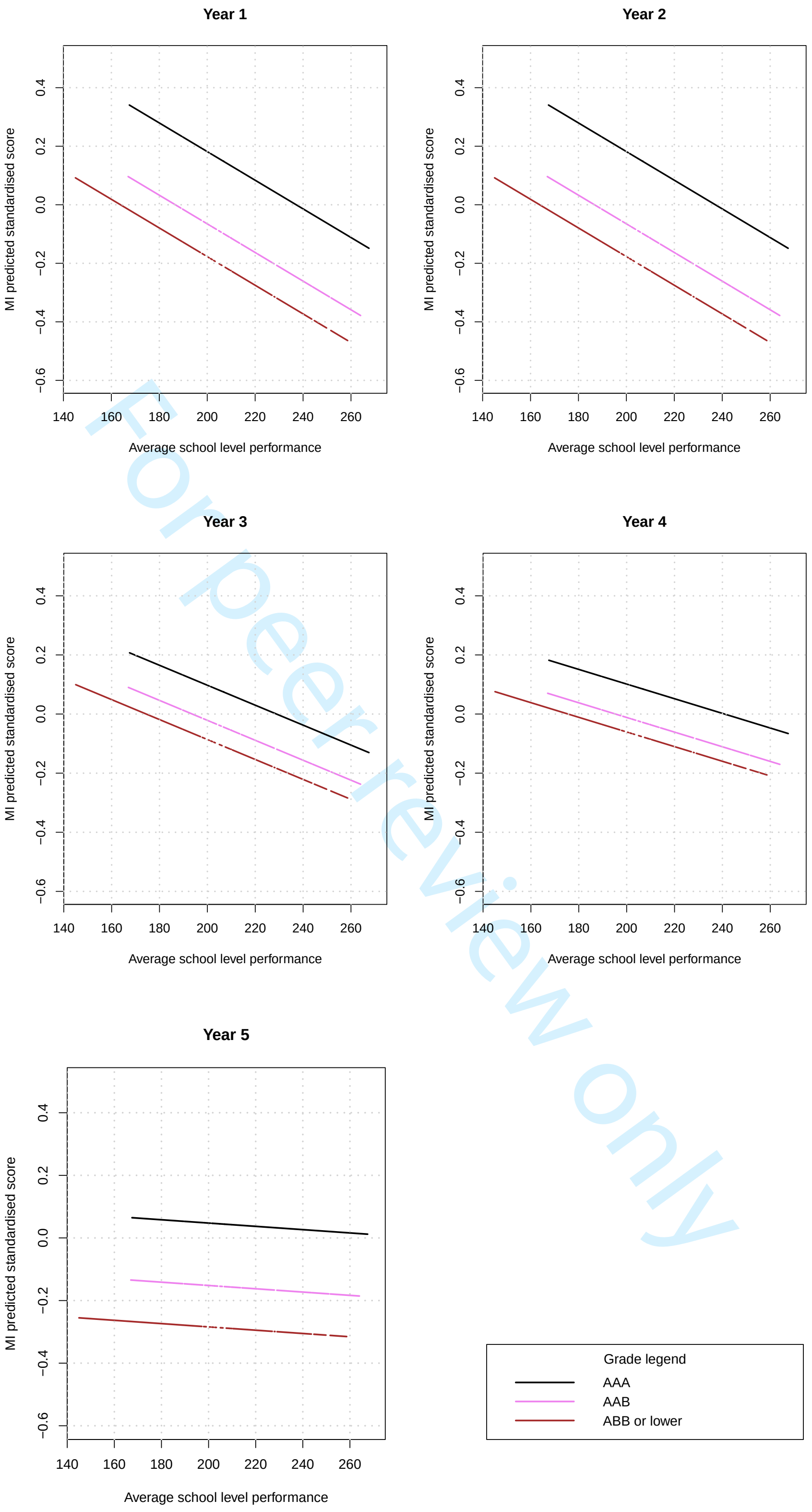

Figure 10: *Multiply imputed effect of average school level performance by reported grades on undergraduate medical school skills-based exams*

For peer review only - http://bmjopen.bmj.com/site/about/guidelines.xhtml

References

[1] IC McManus et al. “The UKCAT-12 study: educational attainment, aptitude test performance, demographic and socio-economic contextual factors as predictors of first year outcome in a cross-sectional collaborative study of 12 UK medical schools”. In: *BMC medicine* 11.1 (2013), p. 244.

[2] William Revelle and Richard E Zinbarg. “Coefficients alpha, beta, omega, and the glb: Comments on Sijtsma”. In: *Psychometrika* 74.1 (2009), pp. 145–154.

[3] Tenko Raykov. “Estimation of composite reliability for congeneric measures”. In: *Applied Psychological Measurement* 21.2 (1997), pp. 173–184.

[4] Jane Adam, Jon Dowell, and Rachel Greatrix. “Use of UKCAT scores in student selection by UK medical schools, 2006-2010”. In: *BMC medical education* 11.1 (2011), p. 98.

[5] Bengt O Muthén Linda K Muthén Tihomir Asparouhov. *Regression And Mediation Analysis Using Mplus*. Muthén & Muthén, 2016, pp. 61–62.

[6] Andrew F Hayes. *My Macros and Code for SPSS and SAS*. URL: http://afhayes.com/spss-sas-and-mplus-macros-and-code.html (visited on 26/01/2017).

[7] Andrew F. Hayes. *The PROCESS macro for SPSS and SAS*. URL: http://www.processmacro.org/index.html (visited on 12/07/2017).

[8] Daniel J Bauer, Kristopher J Preacher, and Karen M Gil. “Conceptualizing and testing random indirect effects and moderated mediation in multilevel models: new procedures and recommendations.” In: *Psychological methods* 11.2 (2006), p. 142.

[9] Geert Verbeke and Geert Molenberghs. *Linear mixed models for longitudinal data*. Springer Science & Business Media, 2009.

[10] Geert Molenberghs and Michael Kenward G. *Missing Data in Clinical Trials*. John Wiley & Sons Ltd, 2007. Chap. Multiple Imputation, pp. 110–114.

[11] Geert Molenberghs and Geert Verbeke. *Models for Discrete Longitudinal Data*. Springer Science & Business Media, 2005. Chap. Multiple Imputation and Expectation-Maximazation Algorithm, pp. 515–516.

For peer review only

For peer review only - http://bmjopen.bmj.com/site/about/guidelines.xhtml

STROBE 2007 (v4) Statement—Checklist of items that should be included in reports of *cohort studies*

Section/Topic	Item #	Recommendation	Reported on page #
**Title and abstract**	1	(*a*) Indicate the study's design with a commonly used term in the title or the abstract	1
		(*b*) Provide in the abstract an informative and balanced summary of what was done and what was found	2-3
**Introduction**			
Background/rationale	2	Explain the scientific background and rationale for the investigation being reported	4-8
Objectives	3	State specific objectives, including any prespecified hypotheses	8
**Methods**			
Study design	4	Present key elements of study design early in the paper	1-3
Setting	5	Describe the setting, locations, and relevant dates, including periods of recruitment, exposure, follow-up, and data collection	9-11
Participants	6	(*a*) Give the eligibility criteria, and the sources and methods of selection of participants. Describe methods of follow-up	9-11
		(*b*) For matched studies, give matching criteria and number of exposed and unexposed	N/A
Variables	7	Clearly define all outcomes, exposures, predictors, potential confounders, and effect modifiers. Give diagnostic criteria, if applicable	9-13
Data sources/ measurement	8*	For each variable of interest, give sources of data and details of methods of assessment (measurement). Describe comparability of assessment methods if there is more than one group	*9-13*
Bias	9	Describe any efforts to address potential sources of bias	24
Study size	10	Explain how the study size was arrived at	9
Quantitative variables	11	Explain how quantitative variables were handled in the analyses. If applicable, describe which groupings were chosen and why	9-13
Statistical methods	12	(*a*) Describe all statistical methods, including those used to control for confounding	9-13
		(*b*) Describe any methods used to examine subgroups and interactions	9-13
		(*c*) Explain how missing data were addressed	24
		(*d*) If applicable, explain how loss to follow-up was addressed	24
		(*e*) Describe any sensitivity analyses	24
**Results**			

For peer review only

Participants	13*	(a) Report numbers of individuals at each stage of study—eg numbers potentially eligible, examined for eligibility, confirmed eligible, included in the study, completing follow-up, and analysed	10
		(b) Give reasons for non-participation at each stage	24
		(c) Consider use of a flow diagram	
Descriptive data	14*	(a) Give characteristics of study participants (eg demographic, clinical, social) and information on exposures and potential confounders	15-16
		(b) Indicate number of participants with missing data for each variable of interest	15-16
		(c) Summarise follow-up time (eg, average and total amount)	2-3,9
Outcome data	15*	Report numbers of outcome events or summary measures over time	
Main results	16	(*a*) Give unadjusted estimates and, if applicable, confounder-adjusted estimates and their precision (eg, 95% confidence interval). Make clear which confounders were adjusted for and why they were included	17-20
		(*b*) Report category boundaries when continuous variables were categorized	N/A
		(*c*) If relevant, consider translating estimates of relative risk into absolute risk for a meaningful time period	N/A
Other analyses	17	Report other analyses done—eg analyses of subgroups and interactions, and sensitivity analyses	
**Discussion**			
Key results	18	Summarise key results with reference to study objectives	20-22
**Limitations**			
Interpretation	20	Give a cautious overall interpretation of results considering objectives, limitations, multiplicity of analyses, results from similar studies, and other relevant evidence	22-24
Generalisability	21	Discuss the generalisability (external validity) of the study results	24-26
**Other information**			
Funding	22	Give the source of funding and the role of the funders for the present study and, if applicable, for the original study on which the present article is based	27-28

*Give information separately for cases and controls in case-control studies and, if applicable, for exposed and unexposed groups in cohort and cross-sectional studies.

Note: An Explanation and Elaboration article discusses each checklist item and gives methodological background and published examples of transparent reporting. The STROBE checklist is best used in conjunction with this article (freely available on the Web sites of PLoS Medicine at http://www.plosmedicine.org/, Annals of Internal Medicine at http://www.annals.org/, and Epidemiology at http://www.epidem.com/). Information on the STROBE Initiative is available at www.strobe-statement.org.

For peer review only

BMJ Open

What is the effect of secondary (high) schooling on subsequent medical school performance? A national, UK-based, cohort study

Journal:	*BMJ Open*
Manuscript ID	bmjopen-2017-020291.R2
Article Type:	Research
Date Submitted by the Author:	06-Apr-2018
Complete List of Authors:	Mwandigha, Lazaro; University of York, Health Sciences<br>Tiffin, Paul; University of York, Health Sciences<br>Paton, Lewis ; University of York, Health Sciences<br>Kasim, Adetayo; Durham University, Wolfson Research Institute for Health and Wellbeing<br>Boehnke, Jan Rasmus; University of Dundee, School of Nursing and Health Sciences
**Primary Subject Heading**:	Medical education and training
Secondary Subject Heading:	Research methods
Keywords:	MEDICAL EDUCATION & TRAINING, STATISTICS & RESEARCH METHODS, EDUCATION & TRAINING (see Medical Education & Training)

SCHOLARONE™ Manuscripts

For peer review only - http://bmjopen.bmj.com/site/about/guidelines.xhtml

What is the effect of secondary (high) schooling on subsequent medical school performance? A national, UK-based, cohort study

Lazaro M. Mwandigha, Paul A. Tiffin, Lewis W. Paton, Adetayo S. Kasim, Jan R. Böhnke

Author Details

LMM: Postgraduate student, Department of Health Sciences, University of York, Heslington, York, YO10 5DD, UK, email; lmm544@york.ac.uk

PAT (Corresponding author): Reader (Associate Professor), Department of Health Sciences, University of York, Heslington, York, YO10 5DD, UK, Tel: 01904 321117, email; paul.tiffin@york.ac.uk

LWP: Research Fellow, Department of Health Sciences, University of York, Heslington, York, YO10 5DD, UK, email; lewis.paton@york.ac.uk

ASK: Senior Research Statistician, Wolfson Research Institute of Health and Wellbeing, Durham University, Queen's Campus, Stockton on Tees, TS17 6BH, UK, email; a.s.kasim@durham.ac.uk

JRB: Senior Research Fellow, Dundee Centre for Health and Related Research, School of Nursing and Health Sciences, University of Dundee, Dundee, DD1 4HJ, UK, AND Department of Health Sciences, University of York, Heslington, York YO10 5DD; email; j.r.boehnke@dundee.ac.uk

For peer review only

For peer review only - http://bmjopen.bmj.com/site/about/guidelines.xhtml

ABSTRACT

Objectives

University academic achievement may be inversely related to the performance of the secondary (high) school an entrant attended. Indeed, some medical schools already offer 'grade discounts' to applicants from less well performing schools. However, evidence to guide such policies is lacking. In this study we analyse a national dataset in order to understand the relationship between the two main predictors of medical school admission in the UK (prior educational achievement (PEA) and performance on the United Kingdom Clinical Aptitude Test (UKCAT)) and subsequent undergraduate knowledge and skills-related outcomes analysed separately.

Methods

The study was based on national selection data and linked medical school outcomes for *knowledge* and *skills*-based tests during the first five years of medical school. UKCAT scores and PEA grades were available for 2,107 students enrolled at 18 medical schools. Models were developed to investigate the potential mediating role played by a student's previous secondary school's performance. Multi-level models were created to explore the influence of students' secondary schools on undergraduate achievement in medical school.

Results

The ability of the UKCAT scores to predict undergraduate academic performance was significantly mediated by PEA in all five years of medical school. Undergraduate

For peer review only - http://bmjopen.bmj.com/site/about/guidelines.xhtml

achievement was inversely related to secondary school-level performance. This effect waned over time and was less marked for *skills*, compared to undergraduate *knowledge*-based outcomes. Thus, the predictive value of secondary school grades was generally dependent on the secondary school in which they were obtained.

Conclusions

The UKCAT scores added some value, above and beyond secondary school achievement, in predicting undergraduate performance, especially in the later years of study. Importantly the findings suggest that the academic entry criteria should be relaxed for candidates applying from the least well performing secondary schools. In the UK, this would translate into a decrease of approximately one to two A-level grades.

view only

ARTICLE SUMMARY

Strengths of the study

- Schools and university data were able to be linked permitting the first UK-based study that compared the academic performance of medical students drawn from poorly performing secondary schools against their counterparts from well-performing ones across all the five years of medical school
- The sample was relatively large with a total of 2,107 medical school students who matriculated in 2008 included in this study

For peer review only - http://bmjopen.bmj.com/site/about/guidelines.xhtml

Limitations of the study

- The *skills* and *knowledge*-based undergraduate assessment outcomes are local, not nationally standardised measures
- There were relatively high rates of missing data in the latter years of the study, especially in relation to undergraduate *skills*-based exams

Word count, excluding title page, abstract, article summary, references, figures and tables is 5,905

INTRODUCTION

Internationally, there is high competition for places to study medicine, and the UK is no exception. Along with the academic demands of medicine as a subject, this has driven medical schools to use secondary (high) school performance as a major determinant to offer a place or not. In general, relatively high obtained (or predicted) grades at senior school are required before a candidate is considered as a potential entrant to medical courses. This emphasis on *prior educational attainment* ('PEA'- the grades obtained at formal exams during secondary education) has partly driven the over-representation of socio-economically privileged individuals in medicine. For example, in North America the majority of US medical school entrants are from relatively affluent backgrounds with around half coming from families in the top fifth for national income.[1] This issue is inevitably reflected in the educational backgrounds of students- it was recently highlighted that 80% of those studying medicine in the UK applied from only 20% of the country's secondary schools.[2] Most of the secondary schools that provide medical students are selective schools, which are better resourced compared to the non-selective schools. Selective schools

For peer review only - http://bmjopen.bmj.com/site/about/guidelines.xhtml

are also highly attended by students in higher social economic backgrounds. Therefore differences in performance between selective and non-selective schools reflect, to a high degree, differences in material deprivation rather than intellectual ability of the students from those schools.[3]

It was partly with this in mind that 'aptitude' tests, mainly tapping into cognitive domains were introduced into medical selection.[4] Such aptitude tests were first used to complement PEA in selection for undergraduate students in the USA in 1928 when the Medical College Admission Tests (MCAT) was developed to address high attrition rates in undergraduate medical school.[5,6] Since this time the use of such tests for selection has spread to other parts of the world.[7-16] PEA has been demonstrated to have predictive validity for undergraduate medical school outcomes in Australia,[17] South Korea,[18] the UK,[19] Saudi Arabia,[20] India,[21] the Czech Republic [22] and New Zealand.[23]. Aptitude tests such as the *Medical College Admission Tests (MCAT)* in the US [24] *Biomedical Admission Test (BMAT)* and *United Kingdom Clinical Aptitude Test (UKCAT)* in the UK, [3,12] *Undergraduate Medicine and Health Sciences Admission Test (UMAT)* in New Zealand,[25] *Hamburg Medical School Natural Science Test (HAM-Nat)* in Germany,[11] *Saudi National Aptitude Exam* in Saudi Arabia [20] and t*he Health Professions Admission Test- Ireland (HPAT-Ireland)* in Ireland [26] have predictive validity for medical school outcomes. Indeed, some critics have highlighted that such aptitude tests may tap into similar constructs as traditional metrics of academic achievement such as high school grades. If this is the case then such measures are unlikely to either facilitate widening access to medicine or add value within the selection process in general.

For peer review only - http://bmjopen.bmj.com/site/about/guidelines.xhtml

Some aptitude tests, such as the BMAT [9] and MCAT,[5] evaluate semantic knowledge of biomedical sciences. These tests may predict undergraduate medical performance, at least in the early years, but are unlikely to add predictive value above and beyond traditional measures of academic attainment.[27] Other tests place more weight on evaluating fluid concepts of cognitive ability, such as the UKCAT.[10] In the case of the UKCAT some, albeit modest, ability to predict undergraduate performance, even after controlling for the effects of secondary school achievement, has been demonstrated.[28] However, it is currently unclear how the predictive abilities of the UKCAT are mediated by PEA, and the extent to which this may vary across both the type of academic outcome and the five year period of undergraduate education in the UK. It has been further suggested that the UKCAT scores may be somewhat less sensitive to the type of secondary school attended, compared to the A-levels sat by students in England and Wales in their final year of schooling.[29] A-levels, usually in three subject areas, are generally undertaken in the last two years of secondary schooling and are roughly equivalent to Advance Placement (AP) courses taken by some students in North America. Findings from an earlier, cross-sectional, study suggested that a strong use of the UKCAT scores during the admissions process may mitigate some of the disadvantage faced by certain under-represented groups applying to study medicine.[30] However, a subsequent study, using longitudinal data, did not report consistent effects over time in this regard.[31]

Whilst PEA does predict academic outcomes in higher education previous studies have observed an inverse relationship with the performance of the secondary (high)

For peer review only - http://bmjopen.bmj.com/site/about/guidelines.xhtml
For peer review only

school attended i.e. students from more highly performing schools tend to get poorer degree awards, after controlling for PEA.[32] To date, the evidence relating to this potential effect in medical school has been inconsistent. One national study observed such an effect in the first year of medical undergraduate training for overall academic performance.[3] A separate, local, study did not.[33] Certain medical courses, designed to widen access to medicine, already ‘discounted’ requirements for certain groups. For example, in Australia a scheme to encourage recruitment to remote, underserved areas, relaxes entry requirements for candidates from rural backgrounds.[34] In the US ‘affirmative action’ policies, albeit at times controversial and repeatedly legally challenged, have been implemented to encourage those from under-represented ethnic groups to enter medical school.[35] In the UK a number of universities have started to offer reduced academic entry requirements for A-level (high school) grades to students from disadvantaged backgrounds who have attended poorly performing secondary schools.[36, 37] Other medical schools are following suit.[38] However, evidence to support such admissions strategies is currently lacking. In the UK, individuals who wish to study at a UKCAT consortium medical school sit the test prior to making an application. The decision to make an offer, for those still at secondary school, is partly based on the predicted A-level (or equivalent) grades. This choice is commonly also informed by early achievement at the GCSE exams, usually taken earlier in the applicant’s school career. Therefore, any offers made would then be conditional on the specified scores obtained first at the UKCAT test before the end of secondary school and later grades being achieved at A-level at end of the secondary school education within each medical school selection cycle. Thus the present study had two aims:

For peer review only - http://bmjopen.bmj.com/site/about/guidelines.xhtml

1. To determine the extent to which the predictive powers of the UKCAT are mediated via PEA, for two separate domains (undergraduate *knowledge* and *skills*-based outcomes) over the period of undergraduate training. Since cognitive ability and educational attainment correlate, we attempt to achieve a more accurate assessment of the relative, and unique, contribution UKCAT scores make within the selection process.
2. To appraise the influence of the performance of the previous secondary school attended on an undergraduate's achievement in medical school. These results will usefully inform policy on grade discounting for applicants applying from poorly performing schools.

For this study we had an opportunity to link national data on the performance of secondary schools to cognitive ability (as evaluated via the UKCAT), PEA and outcomes at 18 UKCAT-consortium medical schools. Thus, there was also the possibility to better understand the interplay between secondary school-level performance, an individual's cognitive ability, their educational attainment (PEA) and how these related to subsequent undergraduate academic achievement. It was therefore hoped that a relatively sophisticated approach to modelling could help understand the role of secondary schooling in both selection (partly based on PEA and aptitude test scores) and later attainment at undergraduate level.

Our findings will inform selection policy in medical school, and in particular provide guidance on the extent to which grades should be discounted for applicants from poorly performing secondary schools.

For peer review only - http://bmjopen.bmj.com/site/about/guidelines.xhtml

METHODS

Data availability and quality

UKCAT consortium medical schools are those medical schools that utilise UKCAT for selection in the UK. For this study, data were available for 18 UKCAT consortium medical schools in England and Wales for candidates who were enrolled between 2007 and 2013. However, Department for Education data on the performance of English secondary schools were only linked to the 2008 entry cohort. For this reason only data relating to these students were used in this study. It should be noted that an advantage of using the 2008 entry cohort was the relatively little missing data throughout the first four of the five year undergraduate period studied. In the 2007 UKCAT testing cycle there were 26 UKCAT-consortium medical schools. Therefore the data represented 69% of the 26 UKCAT-consortium medical schools. All medical school applicants who sat for the UKCAT in 2007 and were selected to join one of the 18 UKCAT-consortium undergraduate medical schools in 2008 were included in this study. As with similar previous studies, non-standard medical courses (e.g. 'widening participation', graduate entry etc) were excluded.[28] Only the marks attained at first sittings of undergraduate exams were retained for each student. Data relating to UKCAT scores and secondary school attainment were available for 2,107 students who entered medical school in 2008 and had linked data relating to the performance of the secondary school they attended.

The secondary school exams sat by the students were nationally standardised and included General Certificate of Secondary Education (GCSE), Advanced Subsidiary (AS) Level and Advanced Level ('A-Level) exams. The GCSE exams are taken at around the age of 15-16 years. Those aspiring to eventually entering higher

For peer review only - http://bmjopen.bmj.com/site/about/guidelines.xhtml

education usually take at least 10 subjects at GCSE level. At the time of the study, the AS levels were sometimes taken in the first year of sixth form (equivalent to high school junior year) as preparation for, or to supplement the full A-level exams taken the subsequent year. For those planning to apply for medicine three subjects at A-level are studied in the last two years of secondary schooling, almost always in the sciences. Candidates frequently take more than three A-levels though universities only count the highest three grades, that usually must be achieved at first sitting.

The completeness of the data relating to the outcomes of interest varied and the flow of the data in the study is depicted in Figure 1.

[Place Figure 1 about here]

The manner in which data related to undergraduate performance in the UKCAT consortium of universities has been collated and managed has been previously described.[28] However, to summarise, the main outcome variables used were the scores achieved at undergraduate *knowledge* and *skills*-based end of year outcomes. It was left to individual institutions to define how their assessments fell into each category. These assessment scores were provided by the universities in percentage forms (of maximum marks achievable) and then converted to standardised z-scores within each institution. Thus, the z scores were created by subtracting the mean performance for that particular year and medical school cohort from an entrant's score and dividing it by the standard deviation for their peers' scores. This created standardised scores with mean zero and a standard deviation of one for each medical school group of students. This standardisation was carried

For peer review only - http://bmjopen.bmj.com/site/about/guidelines.xhtml

out in order to minimise the impact of any variability across medical schools, in terms of the nature of the assessment.

The UKCAT consists of four multiple choice sub-tests timed separately namely *quantitative reasoning, decision analysis*, *verbal reasoning* and *abstract reasoning. Quantitative reasoning* assesses an applicant's ability to critically evaluate information presented in numerical form; *decision analysis* assesses the ability to make sound decisions and judgements using complex information; *verbal reasoning* assesses the ability to critically evaluate information that is presented in a written form, and; *abstract reasoning* assesses the use of convergent and divergent thinking to infer relationships from information. Each of the cognitive subtests have their raw score converted to a scale score that ranges from 300 to 900. Therefore the total scale scores for all of the four subtests range from 1,200 to 3,600. The UKCAT subtests and their total scores were standardised as z-scores according to the scores for all candidates at the year of sitting. The reliability of the UKCAT subtests has previously been evaluated and reported.[39] For the purposes of this study only the total UKCAT score (i.e. the summed total of all four sub-test scores) was used as a predictor. This is because it is the total score that is generally used in selection and represents a summary measure of all the four subtest scores. Full details of the descriptive statistics relating to total UKCAT scores are provided in section 1 of the supplementary document.

In order to develop an overall, and precise, measure of PEA we implemented a novel approach that extended one previously used by McManus et al.[3, 40] This involved

For peer review only - http://bmjopen.bmj.com/site/about/guidelines.xhtml

conceptualising ‘educational achievement’ as a common factor (‘latent trait’). Latent traits cannot be observed or measured directly, only by their effects on behaviour. In terms of attitudes this could be observing certain responses to questionnaires, or in the case of ability, performance on exams and other assessments. Thus, in this case we treated all the commonly taken national exam grades (i.e. GCSE, AS and A-levels) as ‘indicators’ (i.e. observable markers) of an underlying ability (PEA). This approach allowed us to use information contained in all the commonly sat exams during secondary school in England to estimate the overall underlying educational achievement of an entrant. Because the specific method we used easily accommodated missing ‘indicators’ it was irrelevant if only a minority of entrants had taken a specific exam (e.g. history GCSE) and such grades could still be included when estimating PEA. The process resulted in a factor score estimate for each entrant which was provided as a standardised z score, where the mean was zero (average PEA for all applicants, with a standard deviation of 1). Thus this measure of previous educational achievement provided more information on an individual than merely their ‘best of three’ A-level grades. Further details of the estimation of the PEA from the reported GCSEs, AS and A-level grades are provided in section 2 of the supplementary document.

This estimate of PEA was used in the models addressing the first study aim (evaluating the mediating effects of previous educational attainment on the UKCAT’s ability to predict undergraduate performance). However, ‘discounting’ policy focuses on the ‘best of three’ A-level grades required for entry, usually after a provisional offer has been made to an applicant. Therefore for the models addressing the study’s second aim (role of secondary school-level performance on undergraduate

For peer review only - http://bmjopen.bmj.com/site/about/guidelines.xhtml

outcomes) we banded entrants into categories according to A-level grades. Thus, the entrants were grouped into three bands according to the highest three A-level grades achieved. Only 43 (2%) entrants were recorded as having the relatively low A-level grades ‘BBB’ and ‘BBC’. Thus entrants were grouped into those with grades ‘AAA’, ‘AAB’ and ‘ABB or lower’.

English secondary school-level performance data for 2008 were available from the Department for Education (DfE). Thus for this study we defined secondary school-level performance as the average grades (converted to a numeric score) achieved for each student on roll at that educational establishment for that school year. Further details are available from the Department for Education for England website. In this sense ‘performance’ is (narrowly) defined as the average educational attainment, in terms of formal exam grades achieved, for each student on roll, in that educational establishment.

Data sharing statement

This study involved the analyses of anonymised secondary data of medical school entrants. Access to the data may be obtained from the UK Medical Education Database (www.ukmed.ac.uk) following approval of an application.

Patient and public involvement

Patients, carers and members of the public were not involved in the design, conduct and analysis of this study.

MODELLING APPROACHES

For peer review only - http://bmjopen.bmj.com/site/about/guidelines.xhtml

Modelling the relationship between UKCAT scores, PEA and Undergraduate outcomes

Our first aim was to try and understand the extent to which the ability of the UKCAT scores to predict subsequent undergraduate medical school performance were explained by PEA. To answer this question a mediation model was developed. The outcomes of interest (undergraduate *knowledge* and *skills*-based exam results) were local to each participating medical school. The variation in the assessment results across institutions was initially explored using a multilevel modelling approach, but no statistically significant clustering effects by university were observed. For this reason, a simpler approach using a single-level mediation model was used for the analysis (Figure 2). Further details of the single-level mediation model, the multi-level mediation model and rationale for choosing the single-level mediation model are described in section 3 of the supplementary document.

[Place Figure 2 about here]

Modelling the influence of secondary school performance on undergraduate outcomes

The second aim of this study was to evaluate the influence of the performance of an entrant's previous secondary school on subsequent undergraduate achievement. This involved estimating this secondary school-level effect while controlling for an entrant's A-level grades. A multilevel model was required to account for the variation in outcomes between universities.[41] Further details on the multi-level model can be found in section 4 of the supplementary document. From the model we could derive predictions about entrants' performances at medical school, for varying A-level grades and secondary school performance.

For peer review only - http://bmjopen.bmj.com/site/about/guidelines.xhtml

The statistical analyses were conducted using Mplus version 7.4, R and SAS softwares.[42-44] Lucidchart [45] was used to produce the figures and R software was used to generate the graphs of the model predictions.

RESULTS

Descriptive statistics

The numbers of entrants with outcomes available in each category (type and year) are depicted in Table 1. This was not a cohort study in the conventional sense (i.e. entrants could leave and enter the study at any year based on a university deciding when to (not) report the academic outcome measures). Thus, Table 1 also illustrates the missingness for only those entrants who had reported undergraduate *knowledge* and *skills*-based outcomes in the first year of undergraduate medical school. This is to provide a picture of attrition in the conventional sense (i.e. how many participants at baseline remained at subsequent time-points).

	**Undergraduate Knowledge-based outcome**				**Undergraduate Skills based outcome**	
**Academic Year**	**Number of universities**	**Number of students**	**% *Missing***	**Number of universities**	**Number of students**	**% Missing**
1	13	1,453	-	9	1,051	-
2	13	1,404	3.72	9	1,019	3.04
3	11	1,041	28.36	7	729	30.64
4	7	711	51.07	5	668	36.44
5	4	439	69.79	2	260	75.26

Table 1: Study attrition rates due to missing data only for those students who had outcome measures reported in year one of medical school

For peer review only - http://bmjopen.bmj.com/site/about/guidelines.xhtml

Section 5 in the supplementary document provide a detailed summary of the missing data patterns for the outcomes. Of the 2,107 undergraduate medical school entrants, 1,855 had their secondary school-level performance available. The distribution of secondary school-level performance and UKCAT scores achieved by the entrants are depicted in Table 2.

	Year of UKCAT sitting=2007				
	Sample size	Mean	SD	Minimum	Maximum
Average Secondary School-level performance	1,855	225.18	20.09	145	267.5
UKCAT total score	2,107	2,544.47	188.92	1,950	3,190

Table 2: Descriptive statistics of the UKCAT total score and average point entry for the 2,107 entrants from the 987 schools.

Table 3 shows the distribution of A-level grades for the medical school entrants. Note that the majority of the entrants had achieved either AAA or AAB grades at A-level.

Grade	N	%
Missing	36	1.71
AAA	1,463	69.44
AAB	436	20.69
ABB	129	6.12
BBB	29	1.38
BBC	14	0.66

Table 3: A-level grades for the entrants in the study sample.

For peer review only

The prediction of medical school outcomes from UKCAT performance

Figure 3 summarises the results from the models investigating the potential mediating effects of PEA on the relationship between UKCAT scores and undergraduate exam outcomes. The proportion of the predictive power of the UKCAT explained by PEA shown for both undergraduate *knowledge* and *skills*-based medical school outcomes are computed as a quotient of indirect effect of UKCAT through PEA divided by total effect of UKCAT.

[Place Figure 3 about here]

Overall, PEA explains approximately over 43% (dotted black line in the Figure 3) of the statistically significant predictive power of the UKCAT for both undergraduate *knowledge* and *skills*-based exams only in the preclinical years (one and two) of medical school training. For the clinical years (three to five) PEA explains approximately less than 43% of the predictive power of the UKCAT for both undergraduate *knowledge* and *skills*-based exam outcomes. This proportion remains statistically significant but declines somewhat with every subsequent year of training.

The effect of secondary school-level performance on subsequent medical school performance

Both secondary school-level performance and PEA were statistically significantly related to the undergraduate outcomes. No statistically significant interaction was observed between the two variables. Overall, compared to entrants from secondary schools with a high average student performance, those from schools with lower average attainment tended to have better subsequent scores in both undergraduate

For peer review only - http://bmjopen.bmj.com/site/about/guidelines.xhtml

For peer review only

knowledge and *skills*-based exams. Lower levels of secondary school level performance corresponded with higher standardised undergraduate medical school performance as may be observed in Figures 4 and 5.

We intended to make our results relevant to UK medical selectors. Specifically we wished to estimate the level of 'discounting' that should be offered to applicants from disadvantaged educational backgrounds. Thus the results of our models addressing the second study aim are depicted in Figures 4 and 5. We show the actual and predicted (fitted) values from the models in the Figures. Average secondary school performance (mean enrolled student attainment for all secondary schools in England) is shown on the horizontal axis and predicted medical school performance (as a standardized z score) on the vertical axis.

Figure 4 depicts the values in relation to *knowledge*-based exams, according to secondary school-level performance. Similarly, Figure 5 shows the values for undergraduate *skills*-based outcomes. Superimposed on these plotted values are the estimates (with associated 95% confidence bands) for entrants depending on their A-level grades at admission to university. These represent the entrants within the three bands of A-level attainment ('AAA', 'AAB', and 'ABB or lower'). For purpose of demonstration, the horizontal black dotted lines indicate the equivalent level of performance between those entrants from secondary schools at the lower decile of performance and those at the upper decile.

For peer review only - http://bmjopen.bmj.com/site/about/guidelines.xhtml

There are a number of notable trends observed in these graphs. Firstly, students with higher A-level grades outperform those with lower educational achievement. However, this gap narrows when predicting undergraduate *skills*, rather than undergraduate *knowledge*-based outcomes in medical school. The difference also reduces in magnitude as undergraduate education progresses through the years. Indeed for undergraduate *skills*-based outcomes, and for many of the later years, the confidence intervals for the groups' estimates generally overlap. This indicates no statistically significant inter-group differences between those with 'AAB' and 'ABB or lower grades' at the 95% confidence level.

The second most striking feature, and the focus of this study, is that students from less highly performing secondary schools generally outperform those from more highly performing educational institutions for any given A-level grade banding. That is, controlling for the effects of A-level attainment, on average, those from the more poorly performing schools tend to achieve better undergraduate exam results than those from the schools with higher levels of student attainment. The vertical purple and brown dotted lines highlight this feature. They show that those with lower A-level grades (e.g. AAB or ABB) from the lowest performing secondary schools tend to have equivalent undergraduate performance to those entrants from the highest performing educational establishments with top grades (i.e. AAA). It is also notable that this 'secondary school gradient' is generally steepest for undergraduate *knowledge*-based outcomes in the early years of undergraduate study. Thus, the effects of secondary school environment, as with individual previous educational attainment, tends to be less marked for procedural (undergraduate *skills*-based) learning and with advancing time in university study.

For peer review only - http://bmjopen.bmj.com/site/about/guidelines.xhtml

[Place Figure 4 about here]

[Place Figure 5 about here]

DISCUSSION

The findings from previous studies suggested some modest added value of the UKCAT scores to predict undergraduate performance, over and above that provided by conventional measures of academic achievement.[3, 28] Further, the ability of UKCAT scores to predict certain aspects of undergraduate performance was found to be largely independent of prior educational attainment (PEA). This was less true for both undergraduate *knowledge* and *skills* based exams, taken early on in the preclinical years of medical school, where a significant portion of the UKCAT's predictive ability is mediated via previous educational performance.

Our findings on the role of secondary school quality in determining subsequent undergraduate performance are in line with the findings from a previous national study utilising data from the same cohort, as well as more general analysis of data from higher education in England.[3, 32] However, we were able to demonstrate persistence (though attenuation) of these effects over the five years of medical school. It is also in keeping with recently published findings that showed that medical students from state-funded (mainly non-selective) secondary schools tended to academically outperform those from privately funded schools, once at university.[46] Our findings were also consistent with those from an Australian study. This reported that entrants from rural backgrounds tended to have lower educational achievement, both at entry and in the early, pre-clinical years of study. However there were no

For peer review only - http://bmjopen.bmj.com/site/about/guidelines.xhtml

significant inter-group differences in performance observed in the latter, clinical years of undergraduate training. However, some caution must be exercised in interpreting these findings as the study was single site with a relatively small number (N=856) of participating students.[34] The present findings were in contrast to those of a local study, which focussed on the fourth year of medical school, when the effects of secondary schooling are likely to have been less marked.[33] The relatively low numbers of students (N=574) involved in this latter study may have led to a deficiency in study power and thus an inability to demonstrate these effects. Also, by using a more sophisticated approach to statistical modelling we were able to delineate the direct and indirect (mediational) effects of cognitive ability (as assessed via the UKCAT) in determining undergraduate medical academic performance. This highlighted the shifting relative roles that conventional academic achievement versus cognitive ability play as undergraduate training progresses. We were also able to separate, at least crudely, undergraduate outcomes in this study relating to '*knowledge*' and '*skills*' (see also limitations, below). As expected, traditional academic attainment (in the form of PEA), was more predictive and mediated a greater proportion of the UKCAT effects for earlier exam performance. We also observed a narrowing of the effects of secondary education achievement as medical school progressed. This might be expected- as the time since leaving secondary schooling elapses it becomes less relevant to current academic performance. However, this narrowing gap may be due to a positive influence of the university educational environment, which may render prior disparities in educational achievement between students less influential. Alternatively, the shrinking disparity may be, at least in part, due to the students becoming more homogenous over time. Some, less well performing or motivated students, will leave the courses in earlier

For peer review only - http://bmjopen.bmj.com/site/about/guidelines.xhtml

years. Nevertheless, in the UK, as elsewhere, such medical school attrition rates (for all reasons) are very small, ranging from approximately 0.25% for the first year to 0.1% for the final year, for standard entry courses.[47] Therefore this effect will have been only slight. In addition, as medical school progresses there is an increasing emphasis on procedural (undergraduate *skills*-based) learning. Thus, the academic abilities required to highly achieve at written school exams are likely to become less relevant to performance.

Our findings also build on previous research [3] and we were able to demonstrating the value, to some extent of 'contextualising' secondary school achievement across the medical undergraduate years. That is, to some extent, the grades obtained by a student at secondary school must be put in the context of the educational establishment in which they were obtained. A reduction of one to two A-level grades may not appear to be a large adjustment. However, this must be understood in the light of the highly homogenous nature of both medical school applicants and entrants where high proportions obtain the maximum achievable grades. Thus, even one grade difference could represent a standard deviation or so from the mean in a pool of high achieving medical school entrants. Internationally, selectors must understand their equivalent effects, not just for school-type attended, but a range of contextual factors that may be pertinent to their culture. Similarly, they must translate such effects into discounted offers where appropriate, in the metric of their own educational systems.

The main strength of this study is that there were a relatively large number of entrants studied from a range of UK medical schools involved. This provided

For peer review only - http://bmjopen.bmj.com/site/about/guidelines.xhtml

sufficient study power to enable the elicitation of relatively subtle effects and suggests the findings are generalizable to England and Wales. Moreover, the secondary school exams sat by this cohort were nationally standardised, with only a minority of the credits awarded for course work. Thus, any local or regional variation in standards can be assumed to be trivial. Nevertheless a number of limitations must be borne in mind when interpreting the findings. In terms of the outcome measures, the categorisation of undergraduate exams into *skills* and *knowledge* was not operationalised and therefore rely on the participating medical schools to categorise the evaluations. Thus their definition may vary across medical schools. Whilst some of this variation was handled by the use of multilevel modelling a more robust definition of undergraduate '*skills*' based assessments may have been helpful in predicting clinically-orientated performance, which may have been a more faithful proxy for later medical practice. In this regard, a methodology has been proposed to achieve this through the "nationalisation" of "local" measures of undergraduate medical school performance for fair comparisons of graduating medical doctors.[48] It is also acknowledged that it is generally the case that undergraduate skills-based exams to be less reliable than knowledge-based tests.[49] It is thus possible that this likely disparity in reliability may explain the difference in the magnitude of observed relationships associated between the predictors and the two undergraduate medical school outcomes. Thus lower reliability in the measurement of an outcome would have an attenuating effect on strength of the relationships.[19] In addition, scores from the most recently taken UKCAT scores were used. These may not have been a better metric of underlying cognitive ability (being less prone to practice effects), though some early sittings may have been used as 'practice runs' by medical school

For peer review only

applicants. In addition, the most recent UKCAT test results are those used by selectors, thus the ones most relevant to selection policy.

The number of participating universities in the study varied from year to year with higher levels of missing data for undergraduate *skills*-based assessments (compared to *knowledge*) and for the latter years of study. This was a result of medical schools deciding not to return outcome exam data for that year rather than students exiting the study or dropping out from medical school. Therefore the missing data mechanism is likely MCAR or potentially MAR. This was dealt with by modelling the data using a likelihood approach and conducting sensitivity analysis to determine the effect of missingness through Multiple Imputation. Both likelihood modelling approach and Multiple Imputation are valid data handling methods under MCAR and MAR.[50, 51] The results from imputed versus non-imputed datasets can be compared as a form of sensitivity analysis (see section 6 of the supplementary document). These highlight that the results did not vary significantly between imputed versus non-imputed datasets. Therefore, missing data did not adversely impact the results and conclusion of the study.

The quality of secondary schools previously attended by undergraduate medical school entrants varies widely across the UK. However, the fact that 80% of UK medical students come from 20% of secondary schools [2] and tend to come from economically advantaged backgrounds.[52] Thus students from selective, academically high-performing schools are grossly over- represented at medical school. Indeed, a selection process substantially based on predicted or actual A-level performance will greatly advantage applicants from such educational

For peer review only - http://bmjopen.bmj.com/site/about/guidelines.xhtml

institutions. Paradoxically such students, once admitted, may relatively underperform in medical school, compared to their contemporaries from less well performing schools, which tend to be state funded and non-selective in nature. Already some UK medical schools are offering 'discounted' A-level offers to applicants from schools that have students with lower levels of academic attainment.[53-55] Our results suggest that such medical schools may have been (albeit serendipitously) implementing such polices broadly in line with our present findings. That is to say, entrants from the most poorly performing schools have A-level grades that 'worth' one to two grades more than those from the top performing schools, in terms of their ability to predict undergraduate achievement. As can be seen from Figures 4 and 5 the definition of 'low' and 'high' performing secondary school is somewhat subjective. In addition, the suggested 'discounting' would vary according to the outcome of interest. There are also practical challenges to implementing such policies. Not all applicants to medical school will have attended schools which can supply comparable data on their institutional performance. At present even comparison across the three nations making up the UK would be very difficult. One simple way of 'equating' across countries might be to report an applicant's rank within their school. However, further evaluation would have to be performed to assess whether such a relatively crude approach was an effective way of contextualising educational achievement. There is also the possibility of 'gaming' with economically advantaged families strategically placing a student in a less well performing educational institution for the final year of schooling.

Any moves to widen access to medicine may prove controversial, as advantaging certain candidates necessarily means disadvantaging others. Thus, such policies

For peer review only - http://bmjopen.bmj.com/site/about/guidelines.xhtml

must be based on defensible evidence, such as the kind we believe is offered by this study. Moreover, given the very low absolute numbers of applicants and entrants to medical schools from disadvantaged socioeconomic backgrounds only a radical rethinking of 'widening access' is likely to result in substantial changes to the demographics of the medical workforce.

To conclude, we found that the predictive ability of the UKCAT can be explained to some degree by PEA, although this is more pronounced in the early preclinical years of undergraduate school. Significant effects of secondary school-level performance exist which suggest the issue of whether offers of a place to study should be discounted for students from more poorly performing schools. This highlights an urgent need to 'contextualise' secondary school performance in applicants rather than selectors taking grades at face value.

For peer review only

For peer review only - http://bmjopen.bmj.com/site/about/guidelines.xhtml

DECLARATIONS

Contributors

All authors made substantial contribution to this study. LMM conducted the statistical analyses and contributed to the writing of the article. PAT and ASK led the conception, design, supervision of the statistical analyses, interpretation of the results and contributed to the writing of the article. JRB was involved with supervision of the statistical analysis and handling of missing data, interpretation of the results, revising and writing of the manuscript. LWP was involved in the interpretation of the results, revising, writing, and critical appraisal of the manuscript. All authors have approved the final version of the article submitted.

Acknowledgements

The authors thank Rachel Greatrix at UKCAT for assistance with facilitating access to the data used in this study.

Funding

LMM is supported in his PhD, of which this study is a component, via funding from the UKCAT Board. PAT is currently supported in his research by a National Institute for Healthcare Research (NIHR) Career Development Fellowship. PAT is also lead for the DREAMS Network, an international collaboration on selection into the professions, of which LMM, JRB and LWP are also members, which is funded by a Worldwide University Network (WUN) Research Development Fund award.

For peer review only - http://bmjopen.bmj.com/site/about/guidelines.xhtml

This paper presents independent research part-funded by the National Institute for Health Research (NIHR). The views expressed are those of the authors and not necessarily those of the NHS, the NIHR or the Department of Health.

Competing interests

LMM is supported in his PhD project via funding from the UKCAT Board and has received travel expenses incurred for attending a UKCAT Research Group meeting. PAT has previously received research funding from the Economic and Social Research Council (ESRC), the Engineering and Physical Sciences Council (EPSRC), the Department of Health for England, the UKCAT Board, and the General Medical Council (GMC). In addition, PAT has previously performed consultancy work on behalf of his employing University for the UKCAT Board and Work Psychology Group and has received travel and subsistence expenses for attendance at the UKCAT Research Group.

Ethical approval

No human subjects were tested for this study therefore no ethical approval was necessary. The anonymised raw data used in these analyses were made available by the UK Medical Education Database (www.ukmed.ac.uk) following approval of an application. From the data, no piece of information can be used to identify a secondary school, medical school or individual. As such, the identity of the participants is fully protected. All participants consented to the collection of the data for research.

For peer review only - http://bmjopen.bmj.com/site/about/guidelines.xhtml

REFERENCES

1. Grbic D, Jones DJ, Case ST. The role of socioeconomic status in medical school admissions: validation of a socioeconomic indicator for use in medical school admissions. Academic Medicine. 2015;90(7):953-60.

2. Medical School Council. Selecting for Excellence Final Report. 2014. https://www.medschools.ac.uk/media/1203/selecting-for-excellence-final-report.pdf. (accessed 3 March 2017).

3. McManus I, Dewberry C, Nicholson S, Dowell JS. The UKCAT-12 study: educational attainment, aptitude test performance, demographic and socio-economic contextual factors as predictors of first year outcome in a cross-sectional collaborative study of 12 UK medical schools. BMC medicine. 2013;11(1):244.

4. United Kingdom Clinical Aptitude Test (UKCAT) Consortium. UKCAT Annual Report. 2007. https://www.ukcat.ac.uk/media/1135/annual-report-2007-2008.pdf. (accessed 9 September 2017).

5. Association of American Medical Colleges (AAMC). About the Medical College Aptitude Test. 2017. https://students-residents.aamc.org/applying-medical-school/taking-mcat-exam/about-mcat-exam/. (accessed 6 June 2017).

6. McGaghie WC. Assessing readiness for medical education: evolution of the medical college admission test. Jama. 2002;288(9):1085-90.

7. Australian Council for Educational Research (ACER). Health Professions Admission Test-Ireland (HPAT-Ireland). 2017. https://hpat-ireland.acer.org/. (accessed 3 March 2017).

For peer review only - http://bmjopen.bmj.com/site/about/guidelines.xhtml

8. O'Flynn S, Fitzgerald T, Mills A. Modelling the impact of old and new mechanisms of entry and selection to medical school in Ireland: who gets in? Irish journal of medical science. 2013;182(3):421-7.

9. Cambridge Assessment Admissions Testing. The BioMedical Admissions Test (BMAT). 2017. http://www.admissionstestingservice.org/for-test-takers/bmat/. (accessed 3 March 2017).

10. United Kingdom Clinical Aptitude Test (UKCAT) Consortium. UKCAT. 2017. https://www.ukcat.ac.uk/. (accessed 3 March 2017).

11. Hissbach JC, Klusmann D, Hampe W. Dimensionality and predictive validity of the HAM-Nat, a test of natural sciences for medical school admission. BMC medical education. 2011;11(1):83.

12. Emery JL, Bell JF. The predictive validity of the BioMedical Admissions Test for pre-clinical examination performance. Medical education. 2009;43(6):557-64.

13. Australian Council for Educational Research (ACER). Undergraduate Medicine and Health Sciences Admissions Test (UMAT). 2017. https://umat.acer.edu.au/. (accessed 3 March 2017).

14. Hissbach J, Klusmann D, Hampe W. Reliability of a science admission test (HAM-Nat) at Hamburg medical school. GMS Zeitschrift für Medizinische Ausbildung. 2011;28(3).

15. Farrokhi-Khajeh-Pasha Y, Nedjat S, Mohammadi A, Rad EM, Majdzadeh R, Monajemi F, et al. The validity of Iran's national university entrance examination (Konkoor) for predicting medical students' academic performance. BMC medical education. 2012;12(1):60.

For peer review only - http://bmjopen.bmj.com/site/about/guidelines.xhtml

16. Khan JS, Biggs JS, Bano T, Mukhtar O, Tabasum S, Mubasshar MH. Medical Colleges Admissions Test in Punjab, Pakistan. Journal of Ayub Medical College Abbottabad. 2013;25(1-2):64-7.

17. Mercer A, Puddey IB. Admission selection criteria as predictors of outcomes in an undergraduate medical course: A prospective study. Medical Teacher. 2011;33(12):997-1004.

18. Kim T, Chang J-Y, Myung SJ, Chang Y, Park KD, Park WB, et al. Predictors of undergraduate and postgraduate clinical performance: A longitudinal cohort study. Journal of surgical education. 2016;73(4):715-20.

19. McManus I, Woolf K, Dacre J, Paice E, Dewberry C. The Academic Backbone: longitudinal continuities in educational achievement from secondary school and medical school to MRCP (UK) and the specialist register in UK medical students and doctors. BMC medicine. 2013;11(1):242.

20. Al Alwan I, Al Kushi M, Tamim H, Magzoub M, Elzubeir M. Health sciences and medical college preadmission criteria and prediction of in-course academic performance: a longitudinal cohort study. Advances in Health Sciences Education. 2013;18(3):427-38.

21. Gupta N, Nagpal G, Dhaliwal U. Student performance during the medical course: role of pre-admission eligibility and selection criteria. 2013.

22. Štuka Č, Martinková P, Zvára K, Zvárová J. The Prediction and Probability for Successful Completion in Medical Study Based on Tests and Pre-admission Grades. Stanisław Juszczyk. 2012:138.

23. Shulruf B, Poole P, Wang GY, Rudland J, Wilkinson T. How well do selection tools predict performance later in a medical programme? Advances in Health Sciences Education. 2012;17(5):615-26.

For peer review only - http://bmjopen.bmj.com/site/about/guidelines.xhtml

24. Julian ER. Validity of the Medical College Admission Test for predicting medical school performance. Academic Medicine. 2005;80(10):910-7.

25. Poole P, Shulruf B, Rudland J, Wilkinson T. Comparison of UMAT scores and GPA in prediction of performance in medical school: a national study. Medical education. 2012;46(2):163-71.

26. Kelly ME, Regan D, Dunne F, Henn P, Newell J, O'Flynn S. To what extent does the Health Professions Admission Test-Ireland predict performance in early undergraduate tests of communication and clinical skills?–An observational cohort study. BMC medical education. 2013;13(1):68.

27. McManus I, Ferguson E, Wakeford R, Powis D, James D. Predictive validity of the Biomedical Admissions Test: an evaluation and case study. Medical teacher. 2011;33(1):53-7.

28. Tiffin PA, Mwandigha LM, Paton LW, Hesselgreaves H, McLachlan JC, Finn GM, et al. Predictive validity of the UKCAT for medical school undergraduate performance: a national prospective cohort study. BMC medicine. 2016;14(1):140.

29. Tiffin PA, McLachlan JC, Webster L, Nicholson S. Comparison of the sensitivity of the UKCAT and A Levels to sociodemographic characteristics: a national study. BMC medical education. 2014;14(1):7.

30. Tiffin PA, Dowell JS, McLachlan JC. Widening access to UK medical education for under-represented socioeconomic groups: modelling the impact of the UKCAT in the 2009 cohort. BMJ. 2012;344:e1805.

31. Mathers J, Sitch A, Parry J. Population-based longitudinal analyses of offer likelihood in UK medical schools: 1996–2012. Medical education. 2016;50(6):612-23.

For peer review only - http://bmjopen.bmj.com/site/about/guidelines.xhtml

32. Higher Education Funding Council for England (HEFCE). Differences in degree outcomes: Key findings (UMAT). 2014. http://www.hefce.ac.uk/pubs/year/2014/201403/. (accessed 3 March 2017).

33. Thiele T, Singleton A, Pope D, Stanistreet D. Predicting students' academic performance based on school and socio-demographic characteristics. Studies in Higher Education. 2016;41(8):1424-46.

34. Ray R, Woolley T, Sen Gupta T. James Cook University's rurally orientated medical school selection process: quality graduates and positive workforce outcomes. Rural and remote health. 2015;15:1-11.

35. Blake V. Affirmative action and medical school admissions. Virtual Mentor. 2012;14(12):1003.

36. Garlick PB, Brown G. Widening participation in medicine. BMJ: British Medical Journal. 2008;336(7653):1111.

37. Curtis S, Blundell C, Platz C, Turner L. Successfully widening access to medicine. Part 2: Curriculum design and student progression. Journal of the Royal Society of Medicine. 2014;107(10):393-7.

38. University of Birmingham. Medicine and Surgery MBChB: Widening Participation. 2017. http://www.birmingham.ac.uk/undergraduate/courses/med/medicine.aspx?OpenSection=EntryRequirements. (accessed 3 March 2017).

39. Tiffin PA. Understanding the Dimensionality and Reliability of the Cognitive Scales of the UK Clinical Aptitude test (UKCAT): Summary Version of the Report. 2013. https://www.ukcat.ac.uk/media/1182/understanding-the-dimensionality-and-reliability-of-the-cognitive-scales-of-the-ukcat.pdf. (accessed 3 March 2017).

For peer review only - http://bmjopen.bmj.com/site/about/guidelines.xhtml

40. McManus IC, Dewberry C, Nicholson S, Dowell JS. The UKCAT-12 Technical Report. 2012. https://www.ukcat.ac.uk/media/1185/ukcat-technicalreport-march2012-withbackgroundandsummary-sep2013v3.pdf. (accessed 3 March 2017).

41. Verbeke G, Molenberghs G. Linear mixed models for longitudinal data: Springer Science & Business Media; 2009.

42. Sas Institute. SAS software, version 9.4. SAS Institute Cary, NC; 2002-2012.

43. Muthén L, Muthén B. Mplus User's Guide. Seventh Edition. 1998-2015.

44. R Core Team (2017). R: A language and environment for Statistical computing. R Foundation for Statistical Computing, Vienna, Austria. ISBN 3-900051-07-0, URL http://www.R-project.org.

45. Lucid Software Inc. Lucidchart [Internet]. 2017. https://www.lucidchart.com/ (accessed 3 March 2017).

46. Kumwenda B, Cleland JA, Walker K, Lee AJ, Greatrix R. The relationship between school type and academic performance at medical school: a national, multi-cohort study. BMJ open. 2017;7(8):e016291.

47. General Medical Council (GMC). Summary of 2014 Medical Schools Annual Return.. 2015. https://www.gmc-uk.org/Medical_School_Annual_Return_2014_Summary_Report_for_Publication.pdf_61816122.pdf_62533230.pdf. (accessed 12 January 2017).

48. Tiffin PA, Paton LW. Exploring the validity of the 2013 UKCAT SJT- prediction of undergraduate performance in the first year of medical school: Summary Version of Report. 2017. https://www.ukcat.ac.uk/media/1119/exploring-the-validity-of-the-2013-ukcat-sjt-prediction-of-ug-performance-in-1st-yr-of-med-school-summary-version-posted-27032017.pdf. (accessed 19 September 2017).

For peer review only

For peer review only - http://bmjopen.bmj.com/site/about/guidelines.xhtml

49. Wass V, McGibbon D, Van der Vleuten C. Composite undergraduate clinical examinations: how should the components be combined to maximize reliability? Medical Education. 2001;35(4):326-30.

50. Schafer JL, Graham JW. Missing data: our view of the state of the art. Psychological methods. 2002;7(2):147.

51. Enders CK. Multiple imputation as a flexible tool for missing data handling in clinical research. Behaviour research and therapy. 2016.

52. Steven K, Dowell J, Jackson C, Guthrie B. Fair access to medicine? Retrospective analysis of UK medical schools application data 2009-2012 using three measures of socioeconomic status. BMC medical education. 2016;16(1):11.

53. King's College London. Extended Medical Degree Programme MBBS. 2017. https://www.kcl.ac.uk/study/undergraduate/courses/extended-medical-degree-programme-mbbs.aspx. (accessed 13 July 2017).

54. University of Birmingham. Access to Birmingham (A2B). 2017. http://www.birmingham.ac.uk/undergraduate/preparing-for-university/teachers-advisors/reaching-regional/a2b.aspx. (accessed 13 July 2017).

55. University of Birmingham. Routes to the Professions. 2017. http://www.birmingham.ac.uk/undergraduate/preparing-for-university/teachers-advisors/reaching-regional/routes-professions.aspx. (accessed 13 July 2017).

For peer review only

For peer review only - http://bmjopen.bmj.com/site/about/guidelines.xhtml

Figure captions

Figure 1: Flow chart of data available for the outcomes for each of the five academic years of medical school training

Figure 2: Illustration of the conceptual model for the single level mediation effect of previous educational attainment on the association between total UKCAT scores and undergraduate medical school knowledge and skills-based exams

Figure 3: Proportion of the predictive power of UKCAT for undergraduate knowledge and skills-based exam outcomes explained by PEA in medical school. The proportion is computed as a quotient of indirect effect of UKCAT through PEA divided by total effect of UKCAT. The black dotted line denotes the threshold at 43% selected so as to contrast the trend between the 'pre-clinical' (first two) years and the 'clinical' years (three to five) of medical school undergraduate training

Figure 4: Effect of average school level performance by reported grades on undergraduate medical school knowledge-based exams (as a standardized z score) for all secondary schools in England in 2008. The 2nd decile (average school level performance of 200.2) and 8th decile (average school level performance of 251.9) are denoted by the purple and brown vertical lines respectively. The horizontal black dotted lines are arbitrary points chosen to indicate the equivalent level of performance between those entrants from secondary schools at the lower decile of performance and those at the upper decile of performance

For peer review only - http://bmjopen.bmj.com/site/about/guidelines.xhtml

Figure 5: Effect of average school level performance by reported grades on undergraduate medical school skills-based exams (as a standardized z score) for all secondary schools in England in 2008. The 2nd decile (average school level performance of 200.2) and 8th decile (average school level performance of 251.9) are denoted by the purple and brown vertical lines respectively. The horizontal black dotted lines are arbitrary points chosen to indicate the equivalent level of performance between those entrants from secondary schools at the lower decile of performance and those at the upper decile of performance

For peer review only

For peer review only - http://bmjopen.bmj.com/site/about/guidelines.xhtml

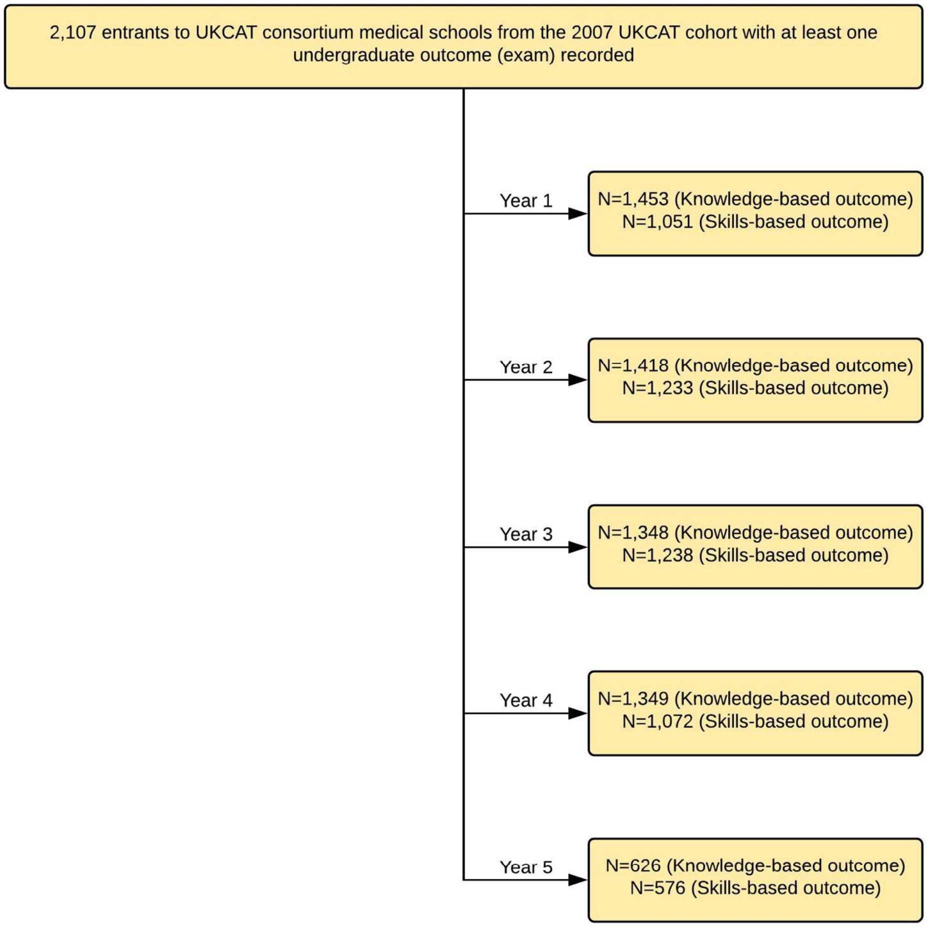
2,107 entrants to UKCAT consortium medical schools from the 2007 UKCAT cohort with at least one undergraduate outcome (exam) recorded
Year 1
N=1,453 (Knowledge-based outcome)
N=1,051 (Skills-based outcome)
Year 2
N=1,418 (Knowledge-based outcome)
N=1,233 (Skills-based outcome)
Year 3
N=1,348 (Knowledge-based outcome)
N=1,238 (Skills-based outcome)
Year 4
N=1,349 (Knowledge-based outcome)
N=1,072 (Skills-based outcome)
Year 5
N=626 (Knowledge-based outcome)
N=576 (Skills-based outcome)

Figure 1

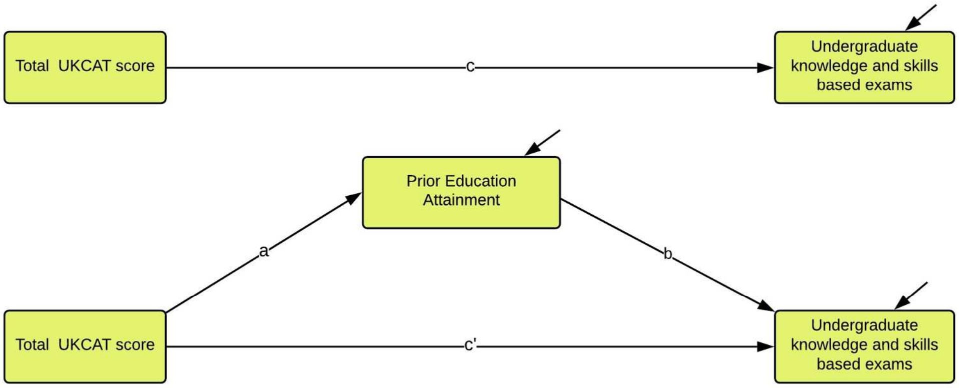

Figure 2

For peer review only - http://bmjopen.bmj.com/site/about/guidelines.xhtml

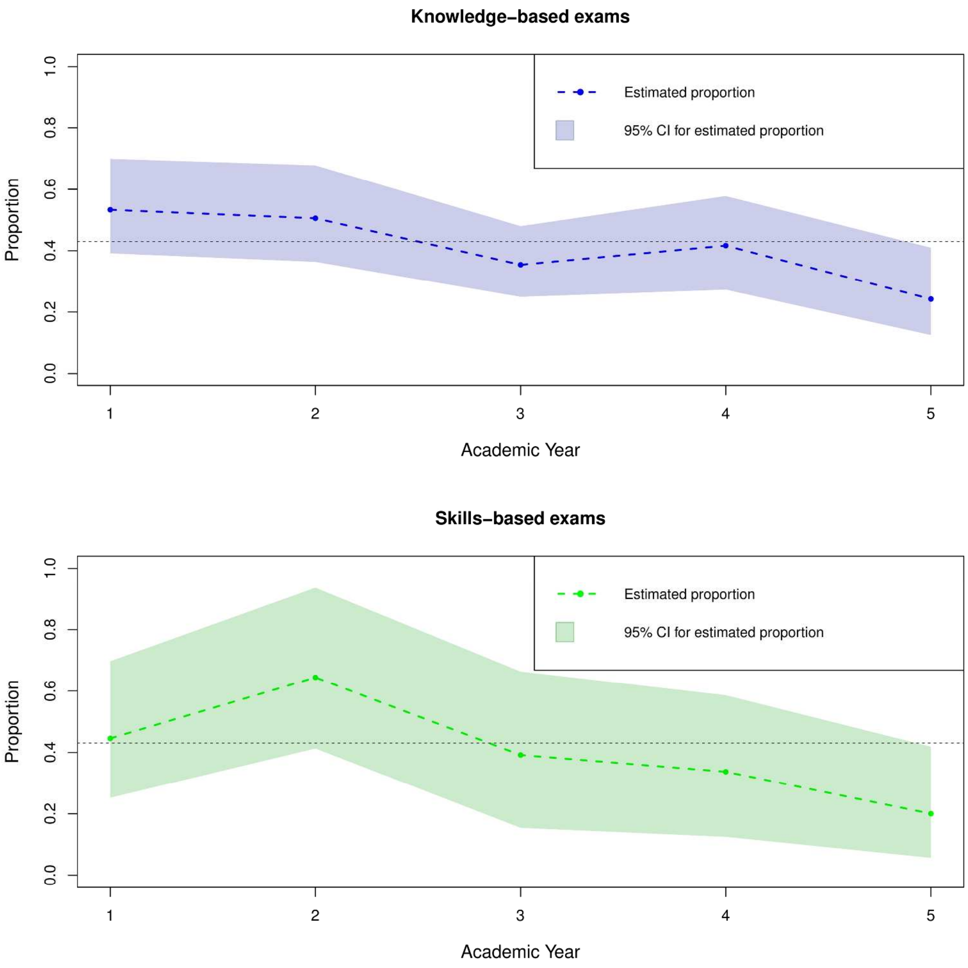

Figure 3

275x275mm (300 x 300 DPI)

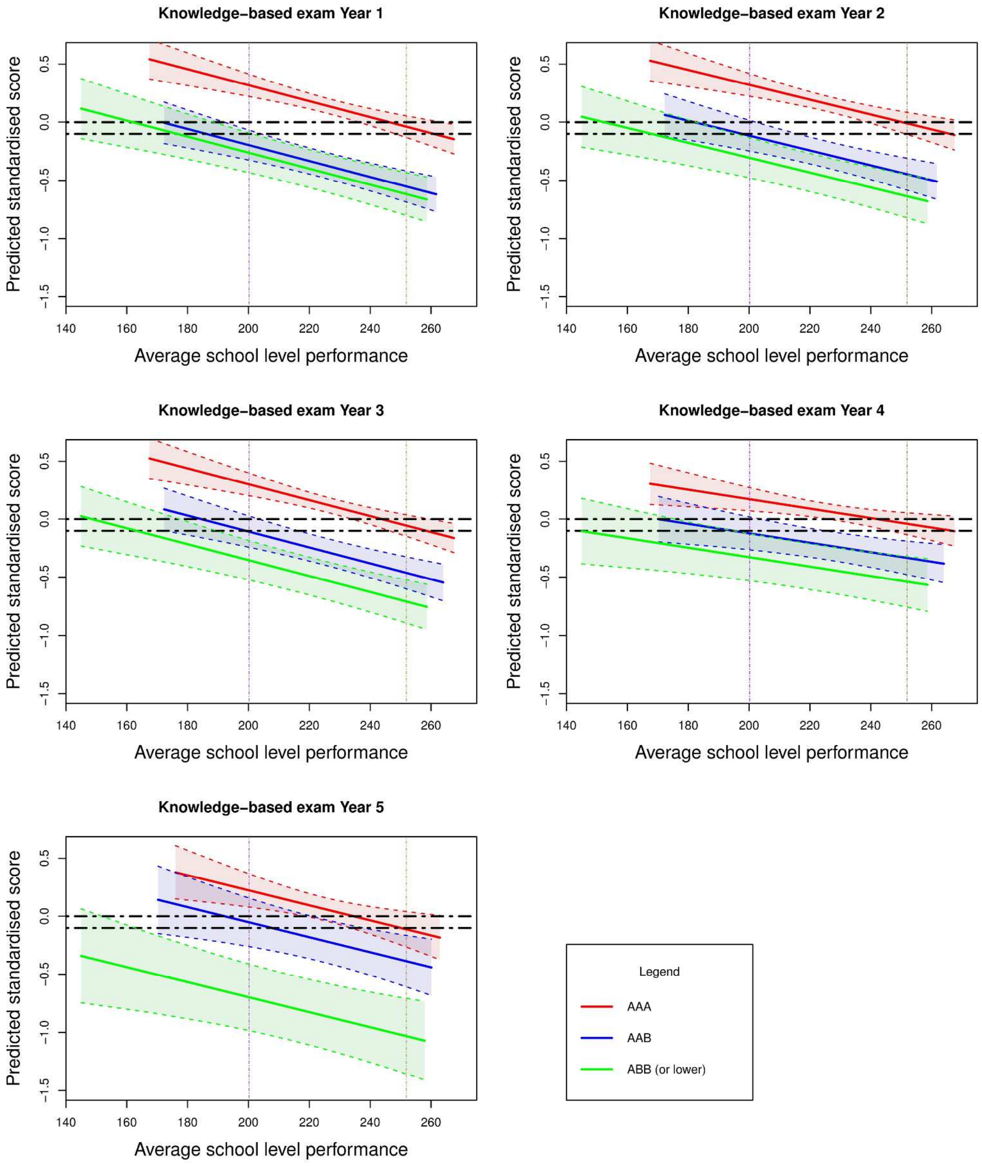

Figure 4

275x328mm (300 x 300 DPI)

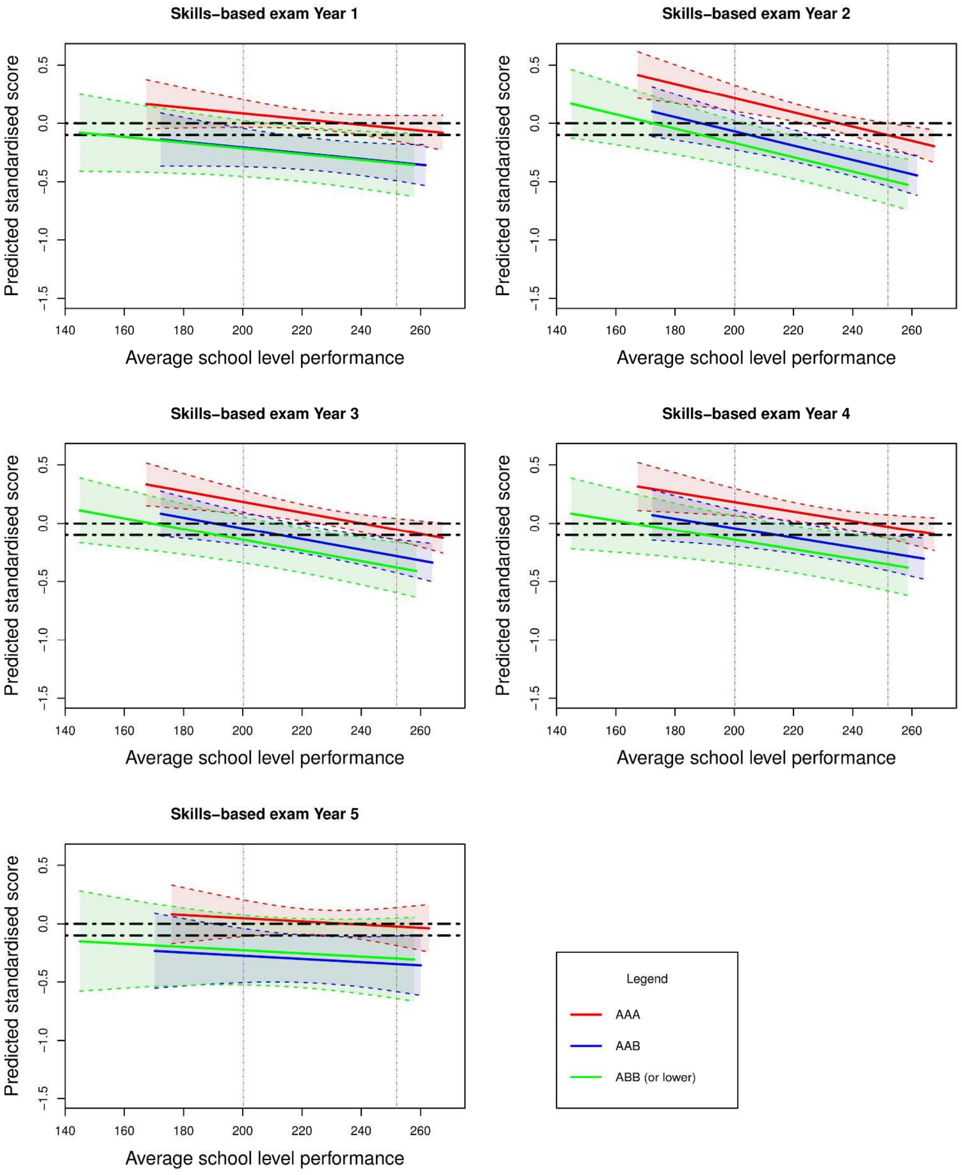

Figure 5

272x332mm (300 x 300 DPI)

Supplementary document to:

What is the effect of secondary (high) schooling on subsequent medical school performance? A national, UK-based, cohort study

Lazaro M. Mwandigha , Paul A. Tiffin, Lewis W. Paton, Adetayo S. Kasim, Jan R. Böhnke

Contents

1 Descriptive and Inferential statistics **2**

2 Estimation of Prior Education Attainment (PEA) **7**

3 Mediation analyses **9**
3.1 Single-level simple mediation analyses 9
3.2 Multi-level simple mediation analyses 10
3.3 Choosing between single-level and multi-level simple mediation analyses 16

4 Multi-level linear model **20**

5 Missing data **22**

6 Sensitivity analysis for missing data **24**
6.1 Single-level simple mediation analyses 25
6.2 Multi-level linear model 28

For peer review only

For peer review only - http://bmjopen.bmj.com/site/about/guidelines.xhtml

1 Descriptive and Inferential statistics

Figure 1 shows the distribution of the entrants total UKCAT scores across the 18 medical schools in the different universities. The distribution of the total UKCAT scores seem to differ widely. This may be partly explained by the fact that different medical schools use the UKCAT differently in the selection process. Some use the UKCAT as a "border-line method" (to discriminate amongst a small number of applicants lying at a decision borderline, who are otherwise indistinguishable on the medical school's other selection criteria), or "factor method" (an applicant's UKCAT score or a proxy for that score is added to the score the applicant obtains in the medical school's usual method of selection, to provide a total score), or "threshold method" (minimum or threshold UKCAT score is adopted to create a hurdle that an applicant must cross to reach the next stage in the selection process) or "rescue" (to compensate for an applicants who would otherwise be rejected on account of their score on other selection criteria) [4].

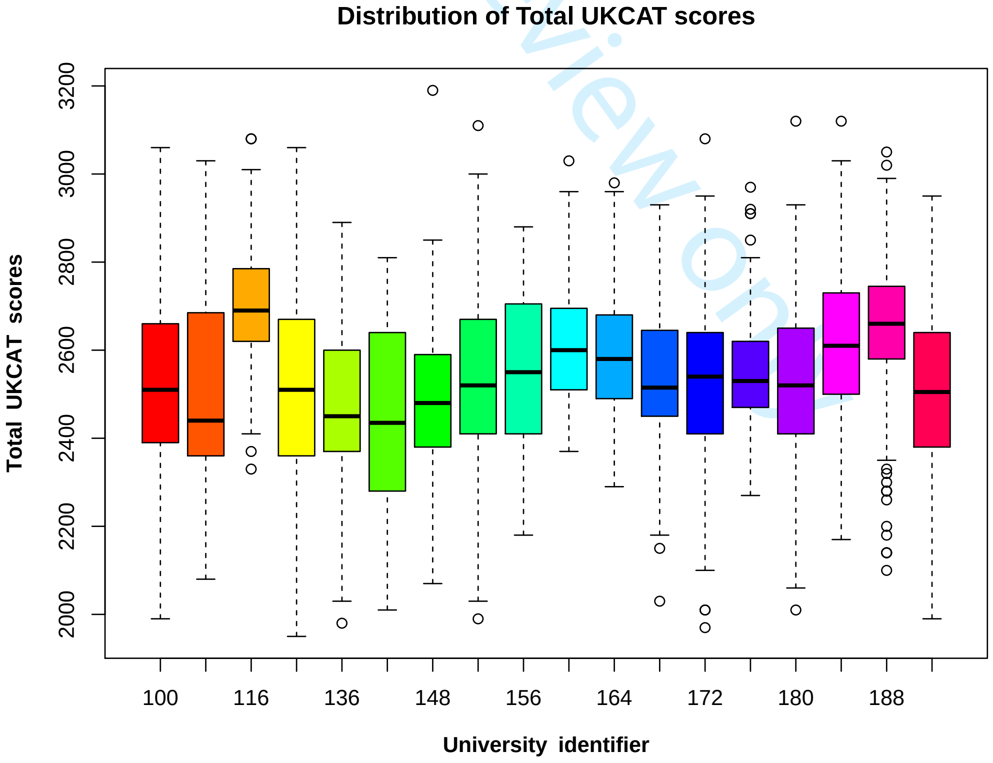

Figure 1: *Box plot of the distribution of the total UKCAT score for the different medical schools in UKCAT-consortium universities*

For peer review only - http://bmjopen.bmj.com/site/about/guidelines.xhtml

Table 1 shows the same information depicted in Figure 1 by means of summary statistics. To determine whether the distributional differences at university level may also be a factor of the quality of secondary school attended by a medical school entrant in a university, the total UKCAT matriculation scores were categorised into three (ranked) groups based on the standardised average performance of secondary schools attended by the entrants.

	University identifier	Mean	SD	Minimum	Maximum
1	100	2,513.06	200.45	1,990	3,060
2	108	2,498.00	246.81	2,080	3,030
3	116	2,690.69	150.22	2,330	3,080
4	120	2,506.16	213.26	1,950	3,060
5	136	2,463.36	186.42	1,980	2,890
6	144	2,448.33	197.10	2,010	2,810
7	148	2,485.37	170.25	2,070	3,190
8	152	2,521.22	219.61	1,990	3,110
9	156	2,550.00	183.80	2,180	2,880
10	160	2,610.18	136.20	2,370	3,030
11	164	2,590.61	149.13	2,290	2,980
12	168	2,524.74	169.51	2,030	2,930
13	172	2,519.26	195.10	1,970	3,080
14	176	2,552.64	120.39	2,270	2,970
15	180	2,522.39	178.94	2,010	3,120
16	184	2,615.40	175.59	2,170	3,120
17	188	2,643.50	177.32	2,100	3,050
18	192	2,502.67	190.36	1,990	2,950

Table 1: *Summary statistics of the total UKCAT score for the different medical schools in the 18 UKCAT-consortium universities*

Secondary schools were categorised into tertiles based on their on their standardised performance. Those with standardised performance of between [-2.5167, 0.3834), [0.3834, 1.2708) and [1.2708, 2.5875] were categorised into ranked groups 1, 2 and 3 respectively. The "[" and "]" indicate the limit is included in the group. The respective number of observations in the ranked groups were 622, 619 and 614 respectively. As may be observed from these values, the (ranked) groups had somewhat an equal number of ob-

For peer review only - http://bmjopen.bmj.com/site/about/guidelines.xhtml

servations. There were 252 observations that were ungrouped due to missing values in average school level performance. For each of the groups, the corresponding standardised UKCAT matriculation scores were examined. The distribution of the total UKCAT matriculation scores for the three ranked groups are shown in Figure 2. The lowest UKCAT performance was observed for entrants who attended secondary schools in group 1. The distribution of the total UKCAT matriculation scores in this group seemed differentiated from the other two groups. The secondary schools represented in group 2 and 3 did not seem differentiated from each other in terms of total UKCAT matriculation scores of medical school entrants who attended them.

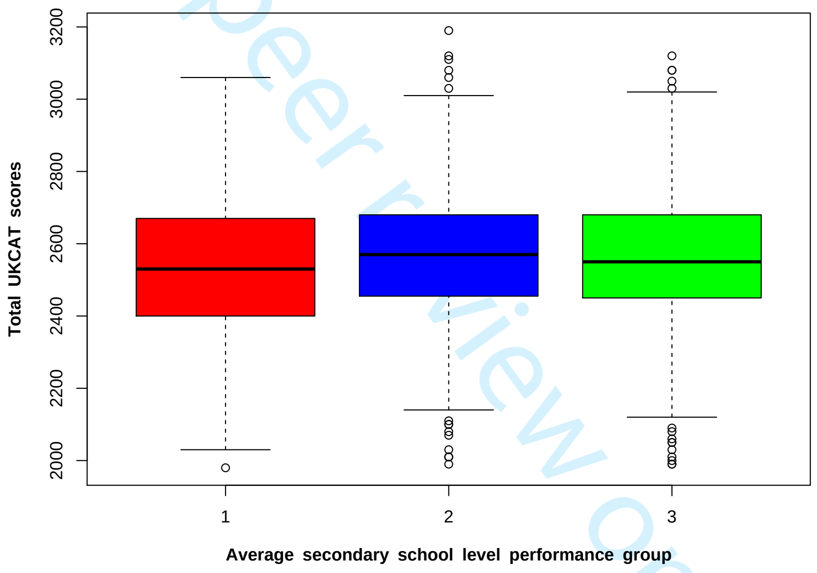

Figure 2: *Box plot of the distribution of the standardised total UKCAT score by category of rank of standardised average secondary school level performance*

To statistically confirm the trend observed in Figure 2, a one-way anova was conducted. The factor of interest was the group which had an ordered level of 1, 2 and 3 based on average secondary school level performance as already described. Following a statistically significant mean difference (p-value < 0.001) in the total UKCAT matriculation scores between the groups, the *Tukey's multiple group* comparison was conducted. This was done to determine the full extent and direction of the differences between the groups. Table 2 shows the results of this comparison which confirm the observed trend in Figure 2.

For peer review only - http://bmjopen.bmj.com/site/about/guidelines.xhtml

Compared to group 1, the total UKCAT matriculation scores were higher for entrants who attended secondary schools in groups 2 and 3. There was no evidence that total UKCAT matriculation scores differed for entrants who attended secondary schools in groups 2 and 3.

	Tukey's anova multiple group comparison			
Rank group	Difference	Lower 95% limit	Upper 95% limit	Adjusted p-value
2-1	43.1593	18.4357	67.8829	0.0001
3-1	34.4978	9.7238	59.2718	0.0032
3-2	-8.6615	-33.4653	16.1423	0.6912

Table 2: Total UKCAT score differences between groups based on the average secondary school level performance

Figure 3 shows the distribution of the undergraduate year one *knowledge*-based outcome scores prior to and after their standardisation within each of the university. Note that only 13 out of the 18 UKCAT consortium (medical schools) universities reported outcomes for knowledge-based exams in the first year. This is clearly seen from the number of box plots in the top and bottom panel with no corresponding standardisation in the bottom panel. The university identified with code 136 reported a single score of 63.61 hence the single line depicted instead of a box plot in the top panel. Universities identified with codes 148, 152, 164, 184 did not report any score for the undergraduate *knowledge*-based exam outcomes at the end of the first year of medical school training. Further, it may be said that standardisation does not affect the distribution of the *knowledge*-based outcome scores as the relative size of the box plots between universities remain the same before and after standardisation. Note that standardisation merely shifts the scale of comparison by allowing the different plots to have mean (value of approximately zero) that is similar across the different universities. Therefore, the underlying differences in the reported outcomes were modelled by a multi-level model (regardless of whether or not standardisation was done or not).

For peer review only - http://bmjopen.bmj.com/site/about/guidelines.xhtml

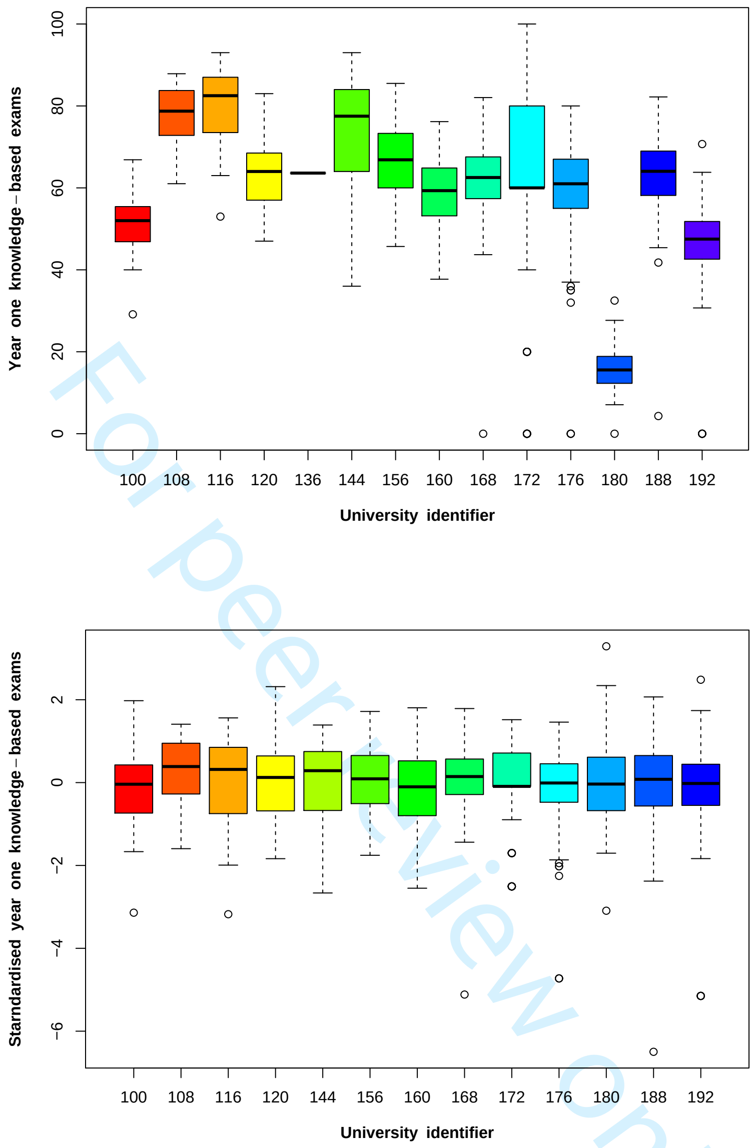

Figure 3: *Box plot of the distribution of end of year one knowledge-based outcomes for the different medical schools in UKCAT-consortium universities. The top panel and bottom panel shows the unstandardised and standardised undergraduate knowledge-based outcome scores respectively*

Table 3 shows the predictive validity of the total UKCAT score and PEA as estimated by bivariate Pearson correlation coefficients. Generally, the predictive validity of PEA was higher than that of the total UKCAT score. It was also observed that the predictive validity for the *knowledge*-based outcomes were higher than that for *skills*-based outcomes. The predictive validity of both the total UKCAT score and PEA was highest in the first two years of medical school training.

For peer review only - http://bmjopen.bmj.com/site/about/guidelines.xhtml

Predictor	Undergraduate knowledge-based outcome				
	Year one	Year two	Year three	Year four	Year five
PEA	0.25 ($<$ 0.001)	0.23 ($<$ 0.001)	0.23 ($<$ 0.001)	0.18 ($<$ 0.001)	0.22 ($<$ 0.001)
total UKCAT score	0.11 ($<$ 0.001)	0.11 ($<$ 0.001)	0.15 ($<$ 0.001)	0.11 ($<$ 0.001)	0.16 ($<$ 0.001)
**Predictor**	**Undergraduate skills-based outcome**				
	Year one	Year two	Year three	Year four	Year five
PEA	0.16 ($<$ 0.001)	0.16 ($<$ 0.001)	0.10 ($<$ 0.001)	0.10 (0.0006)	0.13 (0.0012)
total UKCAT score	0.07 (0.0165)	0.06 (0.0238)	0.06 (0.0319)	0.07 (0.0164)	0.11 (0.0068)

Table 3: *Predictive validities of PEA and total UKCAT score for undergraduate medical school performance. The computed predictive validities are estimated by bivariate Pearson correlation coefficients from pairwise deleted data. The associated p-values for the reported validities are shown in brackets*

2 Estimation of Prior Education Attainment (PEA)

In order to obtain a single metric of scholastic (or academic) ability from the reported GCSEs and A Level exam scores, a novel approach described by *McManus et. al* [1] which involved conceptualising *educational achievement* as a latent variable was used. Thus PEA was estimated as a latent trait via an ordinal factor analysis using the most commonly taken A-level (both A1 and A2), and the grades obtained (e.g. A, B, C etc) used as (ordered categorical) indicators (see Table 4). The non-hierarchical version of McDonald's Omega was computed from the polychoric correlation matrix, since the factor analysis was of first order [2, 3]. The non-hierarchical McDonald's Omega was found to be 0.91. *Full Information Maximum Likelihood (FIML)* which maximizes use of the available data was used for the analysis to deal with missingness in the data (e.g. for the subjects not taken by a particular candidate). Subsequently, factor scores were then estimated for all applicants in the data, the results of the factor analysis from *Mplus* are displayed on Table 5. It was observed that generally, higher loadings were associated with Chemistry, Physics and Biology in GCSEs and A-Level (both A1 and A2) exams.

For peer review only - http://bmjopen.bmj.com/site/about/guidelines.xhtml

Exam	Subjects considered	Grade coding for factor analysis
GCSE	*Biology, Chemistry, Physics, Maths, French, History, Religious studies, Science, English, English literature* and *Geography*	C, D, E, F and G=1, B=2, A=3 and A^{*}=4
A Level (includes A1 and A2-level)	*Maths, Chemistry, Biology* and *Physics*	E and D=1 , C=2, B=3 and A=4

Table 4: *Coding of GCE A-Level and GCSE subjects for factor analysis*

Exam	Subject	Loading	Std Error	Estimate / Std. Error	Two sided p-value
GCSEs	Biology	0.805	0.009	93.988	0.000
	Chemistry	0.815	0.009	95.105	0.000
	English Literature	0.503	0.010	51.869	0.000
	English	0.572	0.009	62.060	0.000
	French	0.611	0.010	60.850	0.000
	Geography	0.696	0.011	60.710	0.000
	History	0.628	0.012	51.736	0.000
	Maths	0.693	0.008	90.998	0.000
	Physics	0.828	0.008	102.854	0.000
	Religious Education	0.510	0.012	43.155	0.000
	Science	0.749	0.049	15.233	0.000
A1-Level	Biology	0.861	0.005	171.013	0.000
	Chemistry	0.822	0.006	149.020	0.000
	Maths	0.798	0.009	93.642	0.000
	Physics	0.847	0.012	71.542	0.000
A2-Level	Biology	0.818	0.006	126.211	0.000
	Chemistry	0.798	0.007	121.959	0.000
	Maths	0.738	0.010	72.379	0.000
	Physics	0.836	0.010	86.867	0.000

Table 5: *Results from the factor analysis for the derivation of factor scores for PEA*

For peer review only - http://bmjopen.bmj.com/site/about/guidelines.xhtml

3 Mediation analyses

3.1 Single-level simple mediation analyses

It was aimed to *determine the extent to which an entrants PEA would mediate the predictive power of the UKCAT for two separate domains (knowledge and skills) over the period of undergraduate training*. To accomplish this a mediation model was considered. This is because, the overall *total predictive power of the UKCAT* for *knowledge* and *skills*-based undergraduate medical school exams would be partitioned into *direct* and *indirect* predictive power. This would then enable the accurate assessment of the relative, and unique, contribution UKCAT scores makes within the selection process. To demonstrate how this is done, consider Figure 4, which shows a *simple mediation model*. The term "simple" means that there is a one predictor, one mediator and one outcome variable under consideration.

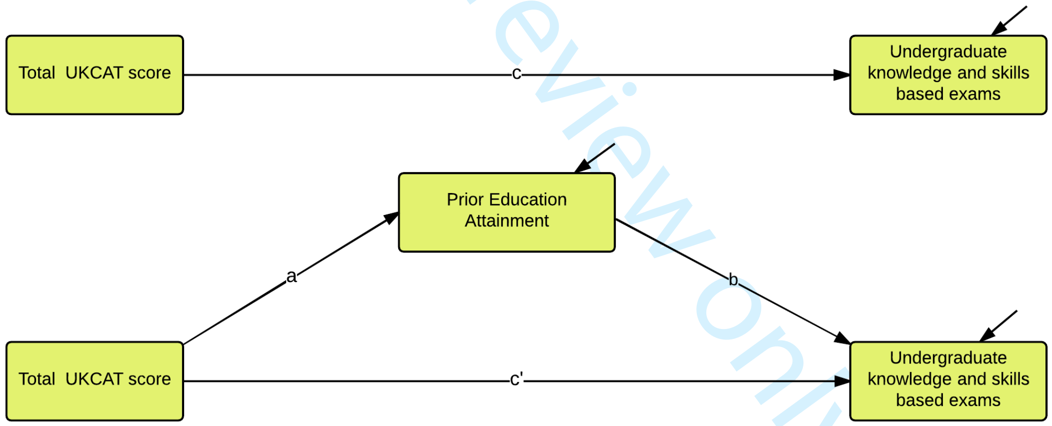

Figure 4: *Conceptual diagram of simple mediation model*

The effect denoted by *c* is the *total effect*, this may be easily obtained as a regression coefficient from a *Ordinary Least Squares (OLS)* regression model. The paths *b* and *c'* are *direct effects* for PEA and UKCAT respectively both of which may be obtained from a OLS regression model. For the purpose of the study, the paths of main interest were the *indirect effect*, product of the paths *a*b*, shown in equation 3.1. This *indirect effect* represents the non-unique contribution of the predictive power of the UKCAT. Further, a

For peer review only - http://bmjopen.bmj.com/site/about/guidelines.xhtml

proportion of this non-unique contribution, which is the portion of the predictive power of the UKCAT that is explained by PEA, may be expressed as $\frac{a*b}{c}$ (see Figure 3 in text of main paper) where c is the *total effect* which has been shown to be equal to sum of the *indirect* and *direct effects*

$$c = a*b + c' \tag{3.1}$$

The significance of the *indirect effect* may be obtained by testing the hypothesis $H_0 : a*b = 0$ versus $H_0 : a*b \neq 0$, traditionally, this was done by assuming a normal distribution for the *indirect effect* of $a*b$ thus necessitating the use of wald, score or likelihood ratio test with their corresponding p-value. This however, may lead to incorrect conclusions, when the *indirect effect* is not normally distributed as is often the case [5]. For this reason, most statistical software packages, such as Mplus implement a hypothesis test using a bootstrap approach which yields an empirical distribution for $a*b$. Similarly, it is possible for one to program this in any statistical software (e.g. R) by implementing a bootstrap or Monte Carlo simulation. The idea being the derivation of $(1-\alpha)100$ bootstrap or Monte Carlo percentile confidence intervals for the purpose of determining signficance. For SAS and SPSS users, macros have been developed for estimating the significance of the *indirect effect*, they include the *INDIRECT* and *PROCESS* macros which are based on the bootstrap while *MCMED* macro is based on Monte Carlo Simulation [6, 7].

3.2 Multi-level simple mediation analyses

The structure of the data used for the study was hierarchical (clustered) because the outcomes (*knowledge* and *skills*) considered in each year of undergraduate training were nested within the 18 universities. This means that fitting a simple mediation analysis which essentially ignored the hierarchical structure of the data would potentially result in *total, direct* and *indirect effects* with induced attenuations. This may then lead to biased conclusions. For this reason, a multi-level mediation model was considered. In a nutshell,

For peer review only - http://bmjopen.bmj.com/site/about/guidelines.xhtml

this model constitutes fitting a simple mediation for each cluster (university) separately and subsequently pooling the effects of interest together in some defined way to form *population average total, population average direct* and *population average indirect effects*. A conceptual representation of this model may be viewed on Figure 5.

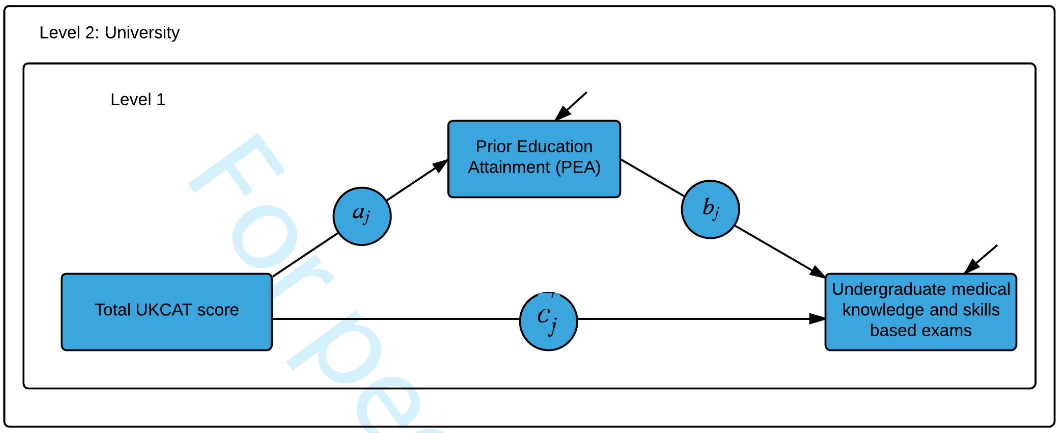

Figure 5: *Conceptual diagram of multi-level mediation model*

Note that, unlike in the case of the simple (single-level) mediation in Figure 4, the effects are now level-1 variables nested within university which is a level-2 variable. Further, all the effects are estimated as random rather than fixed effects thus allowing them to vary between the level-2 variables. This model is called the 1→ 1→ 1 mediation model since the predictor, UKCAT, the mediator, PEA, and the outcomes, *knowledge* and *skills*-based exams, all reside on level-1. In the conceptual representation of the model, the subscript j denotes that effects of interest vary between universities. These effects in the Figure are encircled to denote in *Structural Equation Modelling (SEM)* methodology that these effects are random [8]. The implementation of the 1→ 1→ 1 model is demonstrated for the *knowledge*-based exam scores (denoted by K) for brevity. The UKCAT and PEA scores are denoted by UKCAT and PEA respectively.

$$PEA_{ij} = d_{PEA_j} + a_j * UKCAT_{ij} + \varepsilon_{PEA_{ij}} \tag{3.2}$$

$$K_{ij} = d_{K_j} + b_j * PEA_{ij} + c'_j * UKCAT_{ij} + \varepsilon_{K_{ij}} \tag{3.3}$$

For peer review only

$$d_{PEA_j} = d_{PEA} + \mu_{d_{PEA_j}}$$

$$d_{K_j} = d_K + \mu_{K_j}$$

$$a_j = a + \mu_{a_j}$$

$$b_j = b + \mu_{b_j}$$

$$c^{'}_j = c^{'} + \mu_{c^{'}_j} \tag{3.4}$$

The subscript i denotes a student and subscript j a particular university. Further, $\varepsilon_{PEA_{ij}}$ and $\varepsilon_{K_{ij}}$ are level-1 residuals for the mediator PEA and Knowledge based outcome of interest respectively. Finally, d_{PEA_j}, d_{K_j}, a_j, b_j and $c^{'}_j$ are the random intercepts and slopes of the models. The assumptions of the $1 \to 1 \to 1$ hierarchical mediation model are as follows

1. The predictor, $UKCAT_{ij}$ is uncorrelated with all the random effects (d_{PEA_j}, d_{K_j},a_j, b_j and $c^{'}_j$) and the residuals ($\varepsilon_{PEA_{ij}}$ and $\varepsilon_{K_{ij}}$) in the model.

2. The residuals from the models, $\varepsilon_{PEA_{ij}}$ and $\varepsilon_{K_{ij}}$, are each normally distributed with an expected value of zero and are uncorrelated with one another.

3. The level-1 residuals, $\varepsilon_{PEA_{ij}}$ and $\varepsilon_{K_{ij}}$ are uncorrelated with random effects d_{PEA_j}, d_{K_j}, a_j, b_j and $c^{'}_j$ in the model.

4. The random effects are normally distributed with means equal to the average effects in the population. This may be expressed as,

$$E(a_j) = \bar{a}_j = a$$

$$E(b_j) = \bar{b}_j = b$$

and

$$E(c^{'}_j) = \bar{c^{'}_j} = c^{'}$$

for the slopes of interest. Further, the random effects covary with one another.

5. The distributions of PEA_{ij} is normal conditional on $UKCAT_{ij}$ and K_{ij} normal conditional on PEA_{ij} and $UKCAT_{ij}$.

For peer review only - http://bmjopen.bmj.com/site/about/guidelines.xhtml

These assumptions lead to the following matrix formulation of the model. Note that, it is possible to estimate the *average of effects* (which may be referred to as *"population level effects"*, quantify the effects across all universities and their corresponding variabilities)

$$\begin{bmatrix} d_{PEA_j} \\ d_{K_j} \\ a_j \\ b_j \\ c'_j \end{bmatrix} \sim N\left(\begin{bmatrix} d_{PEA} \\ d_K \\ a \\ b \\ c' \end{bmatrix}, \begin{bmatrix} \sigma^2_{d_{PEA_j}} & & & & \\ \sigma_{d_{PEA_j K_j}} & \sigma^2_{K_j} & & & \\ \sigma_{d_{PEA_j a_j}} & \sigma_{K_j a_j} & \sigma^2_{a_j} & & \\ \sigma_{d_{PEA_j b_j}} & \sigma_{K_j b_j} & \sigma_{a_j b_j} & \sigma^2_{b_j} & \\ \sigma_{d_{PEA_j c'_j}} & \sigma_{K_j c'_j} & \sigma_{a_j c'_j} & \sigma_{b_j c'_j} & \sigma'_{c_j} \end{bmatrix}\right)$$

The *average mediation (indirect) effect* and *average total* effects may then be estimated by making use of equations 3.5 and 3.6 respectively.

$$E(a_j * b_j) = a * b + \sigma_{a_j b_j} \tag{3.5}$$

$$E(a_j * b_j + c') = a * b + \sigma_{a_j b_j} + c' \tag{3.6}$$

The multi-level simple mediation model was fitted in Mplus and the estimates of *average total, average indirect* and *average direct effects* estimated from equations 3.5 and 3.6. The significance of the *average total* and *average direct effects* were obtained from the results in Mplus. To determine the significance of *average indirect effect*, a Monte Carlo 95% Percentile CI was programmed in R software by sampling 10,000 observations from the distribution in equation 3.7.

$$N\left(\begin{bmatrix} a \\ b \\ \sigma_{a_j b_j} \end{bmatrix}, \begin{bmatrix} \sigma^2_a & \sigma_{ab} & \sigma_{a,\sigma_{a_j b_j}} \\ & \sigma^2_b & \sigma_{b,\sigma_{a_j b_j}} \\ & & \sigma^2_{\sigma_{a_j b_j}} \end{bmatrix}\right) \tag{3.7}$$

The individual elements of the distribution in equation 3.7 were obtained from the results of the multi-level mediation model in Mplus using the *TECH 3* output command. Each of the 10,000 observations sampled for a, b and $\sigma_{a_j b_j}$ were plugged into equations 3.5 and 3.6 to obtain 10,000 *average indirect effect* values. Subsequently, the Monte Carlo 95%

For peer review only - http://bmjopen.bmj.com/site/about/guidelines.xhtml

Percentile CI was calculated by taking the 2.5^{th} and 97.5^{th} percentile of the empirical distribution of the 10,000 estimates for *indirect effect*. Figure 6 shows the plotted results from the models. It was observed that there were statistical significant *average indirect effects* in the first four years of undergraduate training of medical school for both *knowledge* and *skills*-based exams outcomes. The *indirect effects* represent the contribution of PEA towards the predictive power of the UKCAT. It was also observed that the range of the CIs widened in the third year onwards which is indicative of the missingness observed in the later years of the study (see Figure 1 and Table 1 in main text of the paper) which led to little information available for analysis in each of the university clusters in the data.

For peer review only

For peer review only - http://bmjopen.bmj.com/site/about/guidelines.xhtml

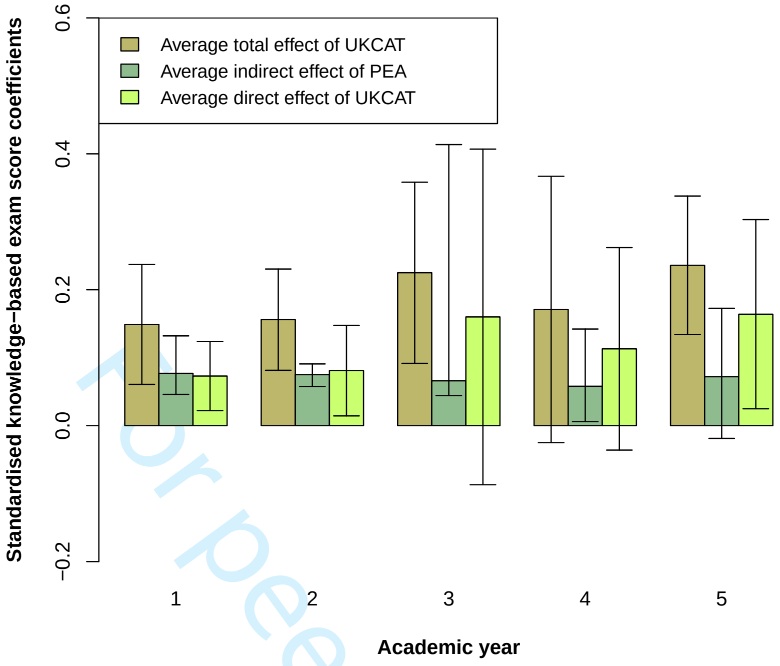

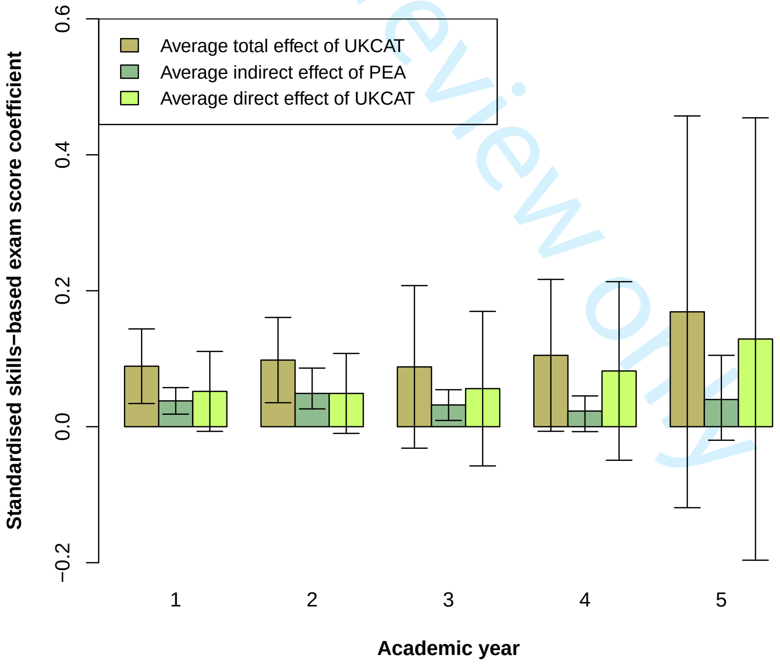

Figure 6: *Knowledge and skills-based multi-level mediation results for the average total, average direct and average indirect effects with respective 95% CI for average total and average direct effects computed from point estimates and standard errors obtained in Mplus and 95% Monte Carlo CI computed in R through simulation*

For peer review only - http://bmjopen.bmj.com/site/about/guidelines.xhtml

3.3 Choosing between single-level and multi-level simple mediation analyses

The multi-level mediation model fitted in section 3.2 is prone to convergence difficulties and is highly susceptible to missing data related problems. For instances where there are high attrition rates in later years of a longitudinal cohort study, it is highly likely that some or most of the clusters may have little or no data to contribute meaningfully to the analysis and this may further risk a lack of convergence. Therefore, for a given estimation problem, a single-level mediation model is preferred if there is evidence that there are no statistically significant clustering effects in the data.

To determine whether there were statistically significant clustering effects in the data equations 3.5 and 3.6 were considered. Note that from equation 3.5, when $\sigma_{a_j b_j} = 0$, the resulting *average indirect effect* is equal to what would be estimated in a single-level simple mediation analysis in section 3.1. Therefore in seeking to determine whether a single or multi-level mediation analysis should be fitted to the data, it will be sufficient to test the hypothesis, $H_0 : \sigma_{a_j b_j} = 0$ versus $H_1 : \sigma_{a_j b_j} \neq 0$. Evidence in favour of the null hypothesis would also be evidence in favour of a simple single-level mediation analysis. The results of the hypothesis test were available as part of the multi-level results in Mplus and are displayed on Table 6. It was observed that all of the p-values were > 0.05 implying that there were statistically non-significant clustering effects in the data. Further, *Intra Cluster Correlations (ICCs)* for the models computed by utilising the main diagonal of the covariance matrix from equation 3.7 and the residual variances from the model are displayed on Table 7. The observed ICCs (7th and 13th column of the Table) indicate that the proportion of variability explained by the multi-level mediation models is negligible. Therefore a simple single-level mediation model is appropriate for the data.

Following the results on Tables 6 and 7, a simple single-level mediation model was fitted using two models, for the case of *knowledge*-based exams outcomes, shown in equation

For peer review only - http://bmjopen.bmj.com/site/about/guidelines.xhtml

(3.9) and (3.8) respectively using the same notation as in section 3.2.

$$PEA_i = I_{PEA} + a * UKCAT_i + \varepsilon_{PEA} \quad (3.8)$$

$$K_i = I_K + c^{'} * UKCAT_i + b * PEA_i + \varepsilon_K \quad (3.9)$$

Academic year	Knowledge- based exams			Skills-based exams		
	$\sigma_{a_j b_j}$	Std. Error	P-value	$\sigma_{a_j b_j}$	Std. Error	P-value
1	-0.007	0.016	0.663	-0.006	0.007	0.414
2	-0.004	0.003	0.284	-0.005	0.012	0.673
3	-0.002	0.066	0.972	0.000	0.003	0.888
4	-0.001	0.006	0.872	-0.004	0.013	0.778
5	0.000	0.031	0.992	-0.001	-0.009	0.951

Table 6: *Results of the hypothesis testing for the statistical significance of* $\sigma_{a_j b_j}$ *from Mplus*

Academic year	Knowledge-based exams					
	σ_a^2	σ_b^2	σ_{res}^2	σ_{PEA}^2	$\sigma_{\sigma_{a_j b_j}}^2$	$\frac{\sigma_{\sigma_{a_j b_j}}^2}{(\sigma_a^2+\sigma_b^2+\sigma_{res}^2+\sigma_{PEA}^2+\sigma_{\sigma_{a_j b_j}}^2)}$
1	0.006	0.003	0.848	1.876	0.000	0.000
2	0.002	0.000	0.894	1.875	0.000	0.000
3	0.093	0.034	0.841	1.875	0.004	0.002
4	0.005	0.006	0.890	1.875	0.000	0.000
5	0.004	0.002	0.902	1.876	0.001	0.000

Academic year	Skills-based exams					
	σ_a^2	σ_b^2	σ_{res}^2	σ_{PEA}^2	$\sigma_{\sigma_{a_j b_j}}^2$	$\frac{\sigma_{\sigma_{a_j b_j}}^2}{(\sigma_a^2+\sigma_b^2+\sigma_{res}^2+\sigma_{PEA}^2+\sigma_{\sigma_{a_j b_j}}^2)}$
1	0.003	0.001	0.888	1.877	0.000	0.000
2	0.011	0.001	0.968	1.876	0.000	0.000
3	0.003	0.001	0.947	1.876	0.000	0.000
4	0.012	0.000	0.893	1.876	0.000	0.000
5	0.003	0.006	0.985	1.877	0.000	0.000

Table 7: *Intra Cluster Correlations (ICC) for knowledge and skills-based exam outcomes for the five years of undergraduate medical school training*

For peer review only - http://bmjopen.bmj.com/site/about/guidelines.xhtml

The mediator of interest is *PEA* while *UKCAT* and *K* are predictor and outcome of interest respectively. The *I* denotes the intercept while *a*,*b* and *c* are the regression coefficients to be estimated. This model was fitted both in Mplus and in SAS. The results of the models from the two software packages were expectedly similar. The statistical significance was tested using the bootstrap approach implemented in Mplus and Monte Carlo simulation in SAS using the *MCMED macro for SAS* [6]. In both Mplus and SAS, the 95% confidence intervals were obtained by taking the 2.5^{th} and 97.5^{th} percentiles of the empirical distribution for $a * b$ from 10,000 sampled observations. The single-level simple mediation model results (from SAS, similar to results from Mplus) are shown in Tables 8 and 9 for *knowledge* and *skills*-based exams outcomes respectively. For both *knowledge* and *skills*-based outcomes, in all undergraduate years, there were statistically significant *indirect effects* of UKCAT through PEA. This means that the predictive power of the UKCAT for undergraduate medical school performance can be partially explained by PEA.

	Knowledge-based exams					
	Direct effect		Indirect effect		Total effect	
Academic year	Estimate	95% CI	Estimate	95% Monte Carlo CI	Estimate	95% CI
1	0.071	(0.002, 0.139)	0.081	(0.059, 0.106)	0.151	(0.083, 0.220)
2	0.073	(0.002, 0.144)	0.074	(0.053, 0.010)	0.147	(0.077 0.217)
3	0.127	(0.058, 0.195)	0.069	(0.049, 0.094)	0.196	(0.129, 0.263)
4	0.086	(0.014, 0.159)	0.062	(0.040, 0.085)	0.148	(0.078, 0.218)
5	0.162	(0.058, 0.266)	0.052	(0.027, 0.087)	0.213	(0.109, 0.318)

Table 8: *Results of the single-level simple mediation model for the undergraduate knowledge-based exam outcome*

For peer review only - http://bmjopen.bmj.com/site/about/guidelines.xhtml

Academic year	Skills-based exams					
	Direct effect		Indirect effect		Total effect	
	Estimate	95% CI	Estimate	95% Monte Carlo CI	Estimate	95% CI
1	0.056	(-0.028, 0.140)	0.045	(0.026, 0.070)	0.101	(0.019, 0.184)
2	0.032	(-0.049, 0.113)	0.059	(0.038, 0.085)	0.091	(0.012, 0.170)
3	0.048	(-0.026, 0.122)	0.031	(0.012, 0.052)	0.078	(0.007, 0.150)
4	0.062	(-0.017, 0.141)	0.032	(0.012, 0.055)	0.094	(0.017, 0.170)
5	0.121	(0.010, 0.232)	0.030	(0.009, 0.063)	0.151	(0.042, 0.261)

Table 9: *Results of the single-level simple mediation model for the undergraduate skills-based exam outcome*

The results from Tables 8 and 9 were used to compute the proportions of the total UKCAT scores explained by the PEA shown in Figure 3 of main manuscript. To determine whether the proportion of total UKCAT scores explained by the PEA in each year of medical school training varied by outcome, a statistical test for the significance of the difference between the proportions was conducted as shown in equation 3.10 in each year of medical school training. The subscripts k and s denote *knowledge* and *skills*-based exams outcomes respectively. The term p denotes the proportion of the total UKCAT scores explained by the PEA. Table 10 shows the results of the statistical test conducted. It was observed that there were statistically significant differences in the proportions of the total UKCAT scores explained by the PEA between the *knowledge* and *skills*-based exams outcomes in all but the fifth year of medical training. It is was also observed that the fifth year of medical school training had very low sample sizes for the two outcomes under consideration. This contributed to a lack of sufficient power to detect differences in the proportions in that year.

$$Z = \frac{(p_k - p_s) - 0}{\sqrt{(\frac{p_k(1-p_k)}{n_k} + \frac{p_s(1-p_s)}{n_s})}} \quad (3.10)$$

For peer review only - http://bmjopen.bmj.com/site/about/guidelines.xhtml

Year	Undergraduate knowledge-based exams		Undergraduate skills-based exams		Results		
	Proportion ($p_k = \frac{a*b}{c}$)	Sample size	Proportion ($p_s = \frac{a*b}{c}$)	Sample size	$p_k - p_s$	Z	P-value
1	0.5331	1,453	0.4455	1,051	0.0875	4.3419	< 0.0001
2	0.5054	1,418	0.6443	1,233	-0.1388	-7.2948	< 0.0001
3	0.3541	1,348	0.3916	1,238	-0.0375	-1.9707	0.0488
4	0.4164	1,349	0.3369	1,072	0.0795	4.0325	0.0001
5	0.2423	626	0.2003	576	0.0420	1.7573	0.0789

Table 10: *Statistical test for the significance of the difference in the proportion of UKCAT explained by PEA between the undergraduate knowledge and skills-based exam outcomes. The p-values are estimated from a standard normal distribution*

4 Multi-level linear model

To address the second aim of the study, which was *to appraise the influence of the performance of the previous secondary school attended on an undergraduates achievement in medical school*, a multi-level linear model or *Linear Mixed Model (LMM)* was used due to its capability to handle clustering in instances where the outcomes are continuous and correlated. The term "mixed" in the Linear Mixed Model comes from the fact that the model estimates both fixed (mean structure) and random effects (random structure). The modelling framework of Linear Mixed Model may be expressed as follows:

$$Y_i = X_i\beta + Z_ib_i + \varepsilon_i \tag{4.1}$$

where

$$b_i \sim N(0, D)$$
$$\varepsilon_i \sim N(0, \Sigma_i)$$

with $b_1 \dots b_N$ and $\varepsilon_1 \dots \varepsilon_N$ being independent. Y_i is the n_i-dimensional outcome (*knowledge* or *skills*-based exams), X_i and Z_i are the design matrices for the fixed and random effects of known predictors respectively, β and b_i are fixed and university specific effects respectively, and ε_i is the vector containing the residual components [9]. X_i is a design

For peer review only - http://bmjopen.bmj.com/site/about/guidelines.xhtml

matrix containing the predictors; average school level performance of the school in which an entrant sat for their A-level exam, an entrant's reported A-level grade (AAA, AAB, ABB, BBB or BBC), interaction between average school level performance and reported A-level grades and the tier of an entrant's secondary school as categorised based on their performance (see Figure 2). Z_i is a design matrix containing a random intercept which modelled the correlation in the outcomes within a university by allowing the (predicted) outcomes to vary between universities.

As seen in Table 11, the effect of the secondary school group (ordered based on their performance as 1, 2 or 3) was not statistically significant. This implies the A-level grades earned by an medical school entrant and the average level performance of secondary school attended are sufficient in explaining the undergraduate medical school outcomes. Further categorisation of secondary schools based on their performance adds no value in explaining undergraduate medical school outcomes. Therefore the proposed model fitted was in line with the predictors shown in Table 12.

	P-values for undergraduate knowledge-based outcome					P-values for undergraduate skills-based outcome				
Predictor	Year one	Year two	Year three	Year four	Year five	Year one	Year two	Year three	Year four	Year five
SSLP	0.0239	0.1939	0.2100	0.4393	0.2284	0.5688	0.0324	0.2272	0.9608	0.5137
A-Level grade	< 0.0001	< 0.0001	< 0.0001	< 0.0001	< 0.0001	0.0002	< 0.0001	0.0002	0.0007	0.0124
SSLP group	0.9551	0.7273	0.5640	0.9078	0.9864	0.9072	0.8393	0.9798	0.3546	0.2564

Table 11: *Results of the multi-level model showing the type 3 tests p-values (Pr. > F) for the predictors of undergraduate knowledge and skills-based outcomes for each of the year of medical school. SSLP is the average Secondary School Level Performance and SSLP group is the three tier categorisation of secondary schools based on their reported average performance*

	P-values for undergraduate knowledge-based outcome					P-values for undergraduate skills-based outcome				
Predictor	Year one	Year two	Year three	Year four	Year five	Year one	Year two	Year three	Year four	Year five
SSLP	< 0.0001	< 0.0001	< 0.0001	< 0.0001	0.0029	0.1403	< 0.0001	0.0014	0.0099	0.5596
A-Level grade	< 0.0001	< 0.0001	< 0.0001	< 0.0001	< 0.0001	0.0002	< 0.0001	0.0002	0.0008	0.0109

Table 12: *Results of the multi-level model showing the type 3 tests p-values (Pr. > F) for the predictors of undergraduate knowledge and skills-based outcomes for each of the year of medical schoo. Only SSLP and A-level grades were been retained in the model. No interaction between SSLP and A-level grades was detected*

For peer review only - http://bmjopen.bmj.com/site/about/guidelines.xhtml

5 Missing data

The missing data patterns for *knowledge*-based outcomes are shown in Table 13, it was observed that only 20.84% of the entrants had complete data for the *knowledge*-based exam outcome throughout the five years of medical school. Monotone pattern of missingness accounted for 47.36% of the missingness data patterns. The most frequently occurring monotone pattern of missingness had outcome data only for year one to year three. On the other hand, the most frequently occurring arbitrary (non-monotone) pattern of missingness had outcome data missing for year one, two and five.

Table 14 shows the pattern of missingness for *skills*-based exam outcome. About 17% of the entrants had complete data for the outcome over the course of the study duration while 41.29% of the data had monotone pattern of missingness. The most occurring monotone pattern of missingness had outcome data missing for year five. The most occurring arbitrary missingness pattern compromising of about 9.5% of the arbitrary missingess pattern was for year one, two and five.

For peer review only

For peer review only - http://bmjopen.bmj.com/site/about/guidelines.xhtml

Outcome						Count	%
Group	Year 1	Year 2	Year 3	Year 4	Year 5		
Complete							
1	O	O	O	O	O	439	20.84
Monotone missingness							
2	O	O	O	O	M	272	12.91
3	O	O	O	M	M	330	15.66
4	O	O	M	M	M	199	9.44
5	O	M	M	M	M	42	1.99
6	M	M	M	M	M	155	7.36
Arbitrary missingness							
7	O	M	O	M	M	7	0.33
8	O	O	M	O	O	50	2.37
9	O	O	M	O	M	114	5.41
10	M	O	O	O	M	7	0.33
11	O	M	O	M	M	3	0.14
12	M	O	M	M	M	4	0.19
13	M	M	O	O	M	274	13
14	M	M	O	M	M	16	0.76
15	M	M	M	O	O	135	6.41
16	M	M	M	O	M	58	2.75
17	M	M	M	M	O	2	0.09
Total						2,107	100

Table 13: *Missingness data patterns for the undergraduate knowledge-based scores for the 2,107 entrants who sat for the UKCAT in 2007. Each "O" and "M" represents each instance where data are present and absent respectively (i.e. the first row represents the proportion of cases with no missing data). Patterns are categorised as either monotone (i.e. where data relating to all subsequent years are missing after the initial missing data year) or arbitrary (i.e. non-monotone)*

For peer review only - http://bmjopen.bmj.com/site/about/guidelines.xhtml

Outcome						Count	%
Group	Year 1	Year 2	Year 3	Year 4	Year 5		
Complete							
1	O	O	O	O	O	360	17.09
Monotone missingness							
2	O	O	O	O	M	308	14.62
3	O	O	O	M	M	61	2.9
4	O	O	M	M	M	199	9.44
5	O	M	M	M	M	26	1.23
6	M	M	M	M	M	276	13.1
Arbitrary missingness							
7	O	O	M	O	M	91	4.32
8	O	M	O	M	M	6	0.28
9	M	O	O	O	M	37	1.76
10	O	M	M	O	O	37	1.76
11	M	O	M	M	M	140	6.64
12	M	M	O	O	O	79	3.75
13	M	M	O	O	M	197	9.35
14	M	M	O	M	M	153	7.26
15	M	M	M	M	O	137	6.5
Total						2,107	100

Table 14: *Missingness patterns for the undergraduate skills-based scores for the 2,107 entrants who sat for the UKCAT in 2007. Each "O" and "M" represents each instance where data are present and absent respectively (i.e. the first row represents the proportion of cases with no missing data). Patterns are categorised as either monotone (i.e. where data relating to all subsequent years are missing after the initial missing data year) or arbitrary (i.e. non-monotone)*

6 Sensitivity analysis for missing data

Sensitivity analysis was conducted to determine to what extent the missingness in the data influenced the results of the study. The data analysis for the study assumed *Missing At Random (MAR)* mechanism. The MAR assumption was invoked by making use of *ignorability* which entailed ignoring the missingness process. The purpose of the sensitivity analysis was to investigate whether this assumption was justifiable. This involved refitting the models with multiply imputed data and comparing the results from these models with those fitted previously under ingnorability. The premise being, if ignorability is valid under MAR, and *Multiple Imputation (MI)* which is also valid under MAR, then the results under both should be similar. When this is the case, the assumption of ignorability

For peer review only - http://bmjopen.bmj.com/site/about/guidelines.xhtml

For peer review only

and MAR would be justified.

6.1 Single-level simple mediation analyses

For the single-level simple mediation analysis, the models were fitted after imputation was conducted 30 times thus creating 30 datasets. These datasets were analysed and results later summarised through pooling of the estimates. The computation of associated standard errors of their estimates was also done. The MI was conducted in SAS using the *Monte Carlo Markov Chain (MCMC)* which imputes the missing values in the data in a way that retains the overall mean and covariate structure of the data assuming a joint multivariate normal distribution [10, 11]. The results of the previous non-imputed data displayed in Table 8 and 9 for both *knowledge* and *skills*-based exams are further displayed in graphical form in Figure 7. These were compared to the results from the multiply imputed data which are found on Figure 8. It was observed that in as far as the aim of the analysis was concerned, there were no discernible difference in the estimates and conclusions regarding the *indirect effects* of UKCAT through PEA for both the *knowledge* and *skills*-based outcomes from both the multiply imputed and non-imputed data. This implies that the assumptions of ignorability and MAR were plausible and that the missingness though severe in later years of the study, did not adversely effect the results and conclusions of the statistical analysis. This is expected as the missing data was created when participating medical schools failed to submit outcome data the UKCAT database in a that particular year. Thus, it may be concluded that the missing data was unlikely to threaten the validity of the inferences drawn from the results.

For peer review only - http://bmjopen.bmj.com/site/about/guidelines.xhtml

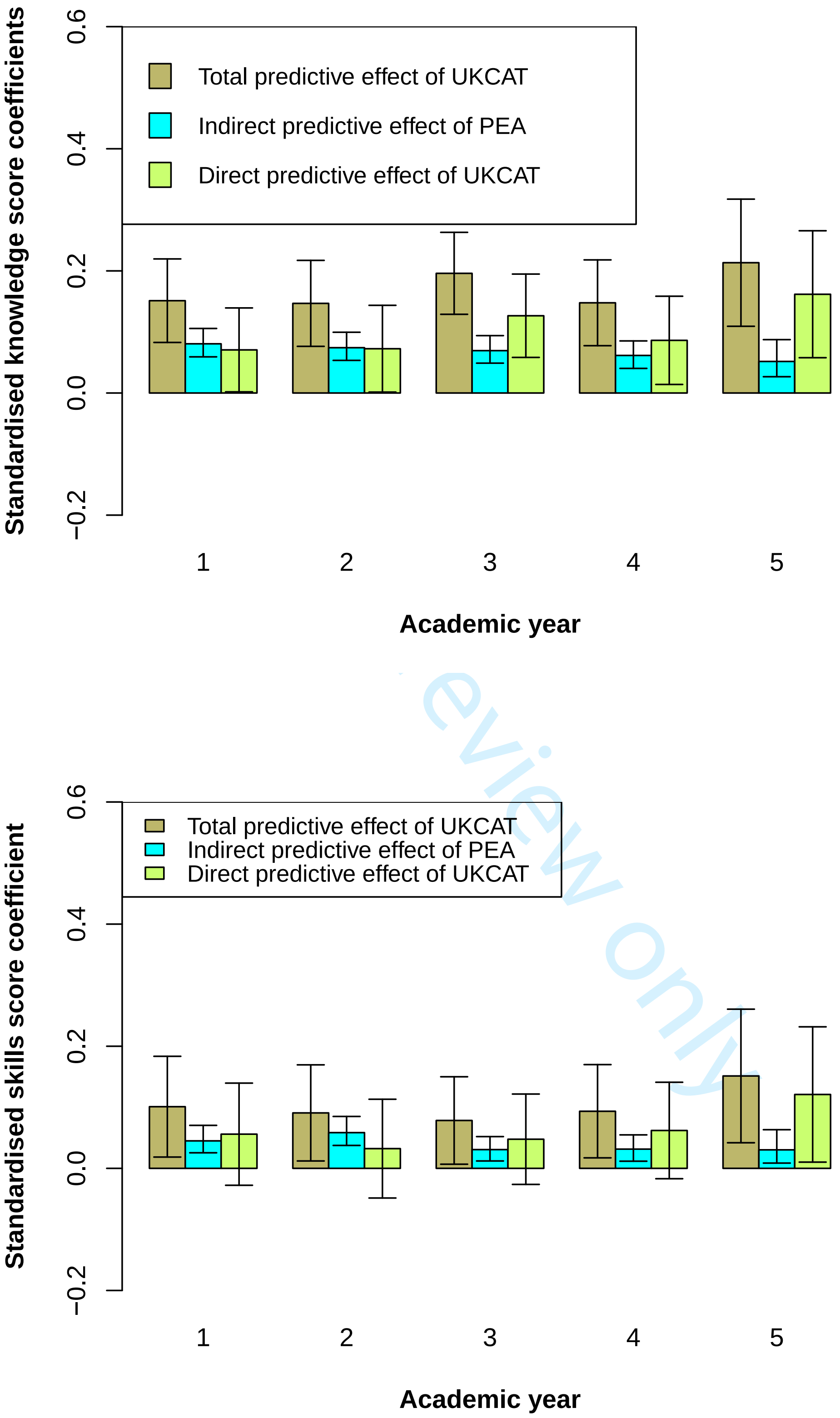

Figure 7: *Results of the single-level simple mediation analysis based on non-imputed data for undergraduate medical school knowledge and skills-based outcomes*

For peer review only - http://bmjopen.bmj.com/site/about/guidelines.xhtml

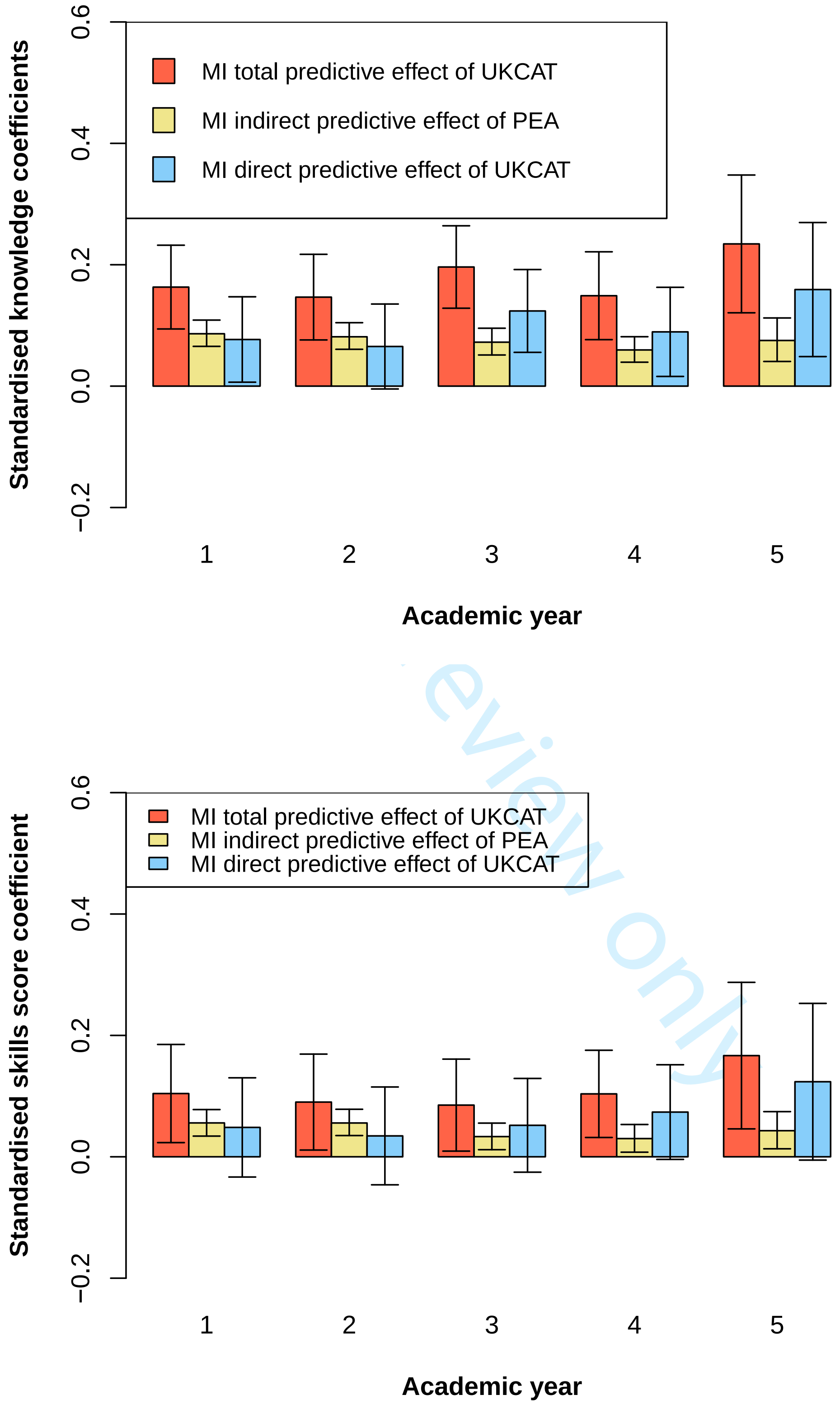

Figure 8: *Results of the single-level simple mediation analysis based on 30 MI data for undergraduate medical school knowledge and skills-based outcomes*

For peer review only - http://bmjopen.bmj.com/site/about/guidelines.xhtml

6.2 Multi-level linear model

Figure 9 and 10 show the plots of MI results for the model investigating the effect of average school level performance by reported grades on *knowledge* and *skills*-based exam outcomes for all five years of undergraduate medical school. All the variables of interest, that is, *knowledge* and *skills*-based undergraduate medical exam outcomes, average school level performance and PEA grades were affected by missingness. MI was conducted using *Multiple Imputations by Chain Equations (MICE)*, a MCMC based imputation technique that makes use of a collection of univariate conditional distributions of the variables with missing values given the other variables present in the data [10]. The number of imputations , M, was initially set at 5 and increased by multiples of 5 until a value of M that would yield unchanging results for the model described in section 4. The parameter estimates obtained were the same for $M >= 10$ indicating that any choice of $M >= 10$ was optimal. For comparison with results from the original data, M=15 was used.

The results from MI data were compared to those from the original data shown in Figures 4 and 5 in the main text of the paper for both *knowledge* and *skills*-based exams outcomes. The comparison revealed that the missingness did not an adverse effect on the analysis. Like in the original unimputed data, for both *knowledge* and *skills*-based exam outcomes, at each level of average school level performance students with higher grades tend to perform better compared to their counterparts with lower grades throughout undergraduate medical school. Overall, compared to students from schools with high average school level performance, students from schools with low average school level performance tend to have better scores in both *knowledge* and *skills*-based exam outcomes throughout undergraduate medical school. This suggests that the assumption of MAR invoked for the study was plausible.

For peer review only - http://bmjopen.bmj.com/site/about/guidelines.xhtml

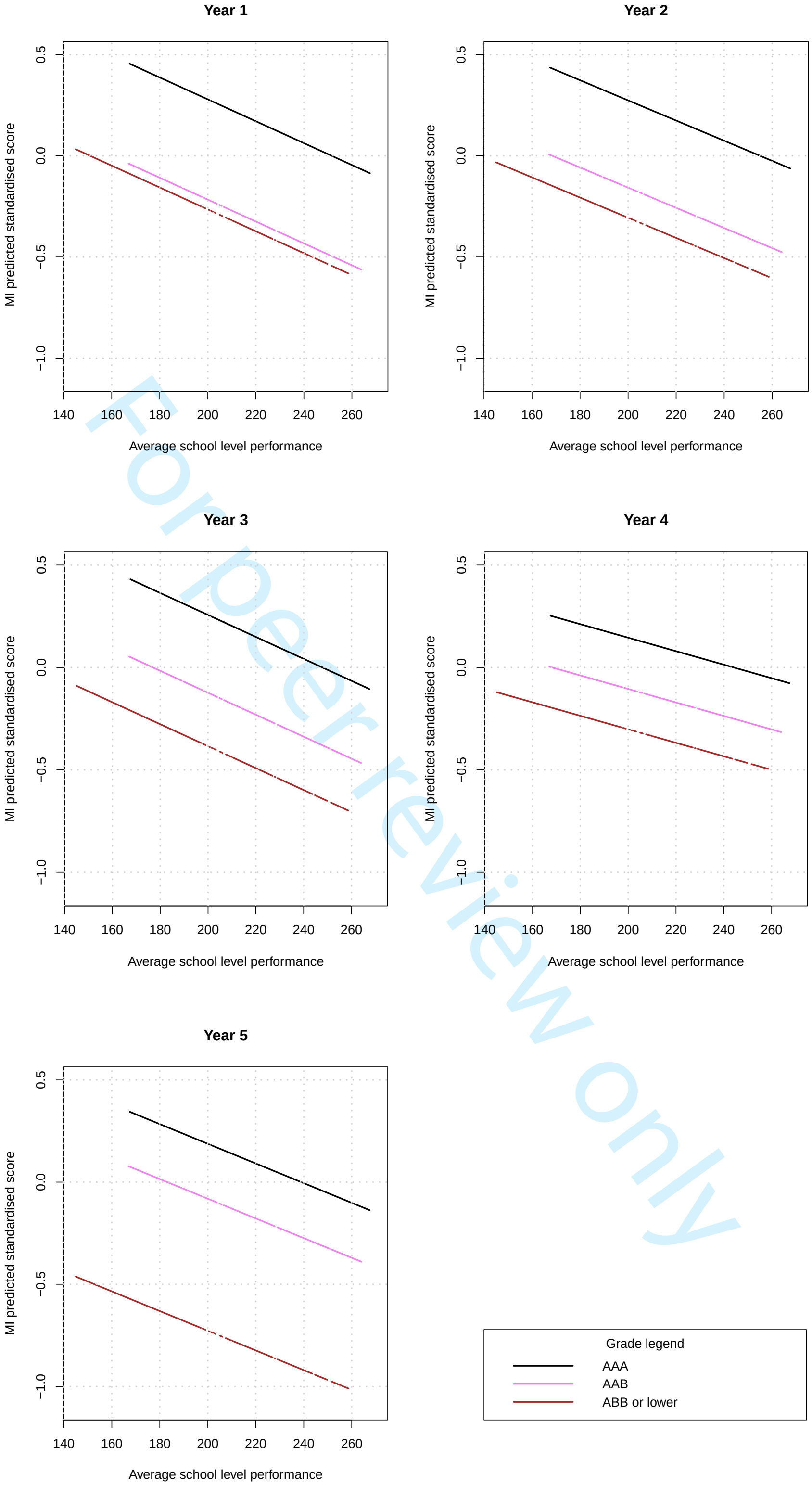

Figure 9: *Multiply imputed effect of average school level performance by reported grades on undergraduate medical school knowledge-based exams*

For peer review only

For peer review only - http://bmjopen.bmj.com/site/about/guidelines.xhtml

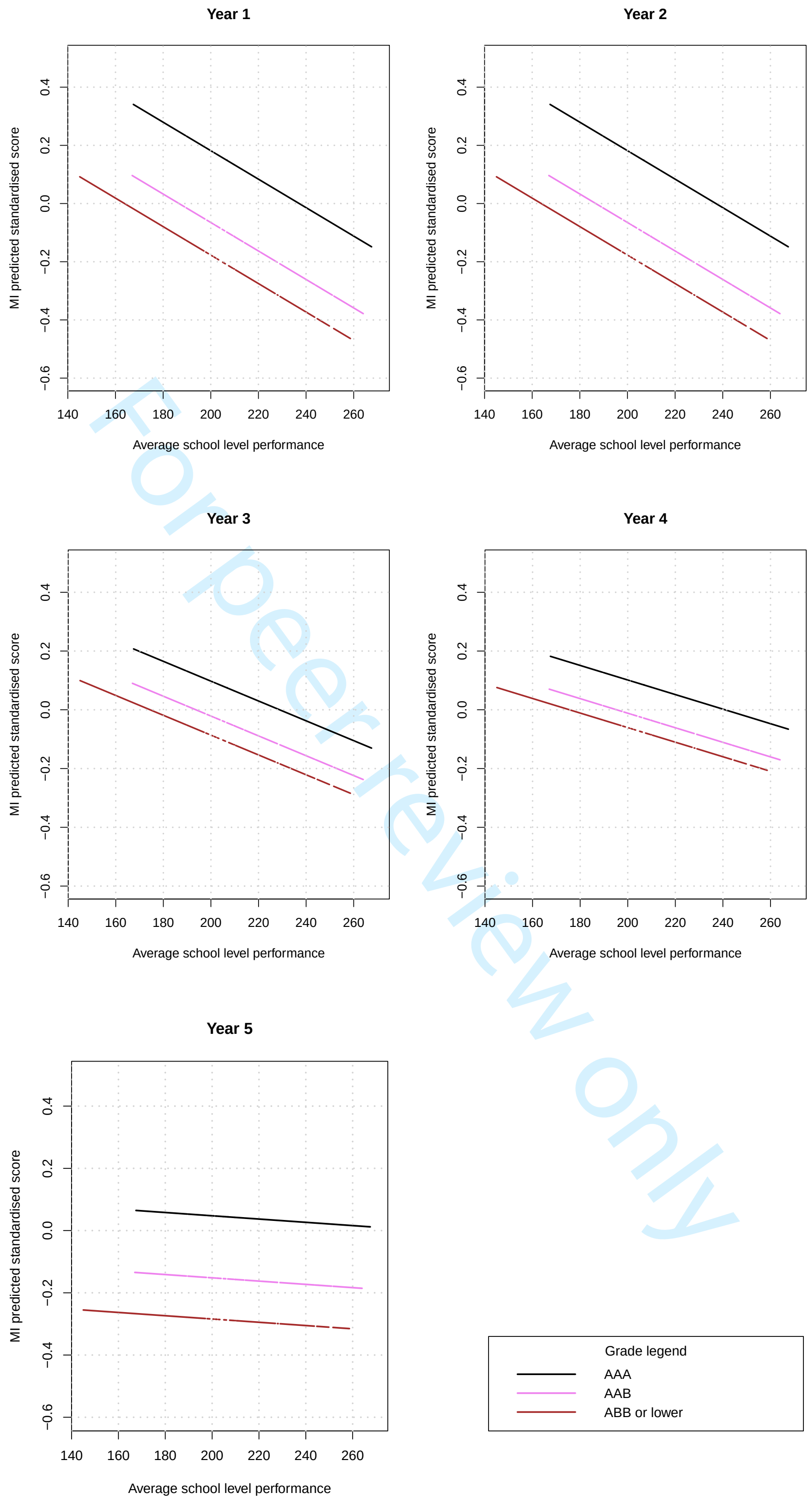

Figure 10: *Multiply imputed effect of average school level performance by reported grades on undergraduate medical school skills-based exams*

For peer review only - http://bmjopen.bmj.com/site/about/guidelines.xhtml

References

[1] IC McManus et al. “The UKCAT-12 study: educational attainment, aptitude test performance, demographic and socio-economic contextual factors as predictors of first year outcome in a cross-sectional collaborative study of 12 UK medical schools”. In: *BMC medicine* 11.1 (2013), p. 244.

[2] William Revelle and Richard E Zinbarg. “Coefficients alpha, beta, omega, and the glb: Comments on Sijtsma”. In: *Psychometrika* 74.1 (2009), pp. 145–154.

[3] Tenko Raykov. “Estimation of composite reliability for congeneric measures”. In: *Applied Psychological Measurement* 21.2 (1997), pp. 173–184.

[4] Jane Adam, Jon Dowell, and Rachel Greatrix. “Use of UKCAT scores in student selection by UK medical schools, 2006-2010”. In: *BMC medical education* 11.1 (2011), p. 98.

[5] Bengt O Muthén Linda K Muthén Tihomir Asparouhov. *Regression And Mediation Analysis Using Mplus*. Muthén & Muthén, 2016, pp. 61–62.

[6] Andrew F Hayes. *My Macros and Code for SPSS and SAS*. URL: http://afhayes.com/spss-sas-and-mplus-macros-and-code.html (visited on 26/01/2017).

[7] Andrew F. Hayes. *The PROCESS macro for SPSS and SAS*. URL: http://www.processmacro.org/index.html (visited on 12/07/2017).

[8] Daniel J Bauer, Kristopher J Preacher, and Karen M Gil. “Conceptualizing and testing random indirect effects and moderated mediation in multilevel models: new procedures and recommendations.” In: *Psychological methods* 11.2 (2006), p. 142.

[9] Geert Verbeke and Geert Molenberghs. *Linear mixed models for longitudinal data*. Springer Science & Business Media, 2009.

[10] Geert Molenberghs and Michael Kenward G. *Missing Data in Clinical Trials*. John Wiley & Sons Ltd, 2007. Chap. Multiple Imputation, pp. 110–114.

[11] Geert Molenberghs and Geert Verbeke. *Models for Discrete Longitudinal Data*. Springer Science & Business Media, 2005. Chap. Multiple Imputation and Expectation-Maximazation Algorithm, pp. 515–516.

For peer review only

For peer review only - http://bmjopen.bmj.com/site/about/guidelines.xhtml

STROBE 2007 (v4) Statement—Checklist of items that should be included in reports of *cohort studies*

Section/Topic	Item #	Recommendation	Reported on page #
**Title and abstract**	1	(*a*) Indicate the study's design with a commonly used term in the title or the abstract	1
		(*b*) Provide in the abstract an informative and balanced summary of what was done and what was found	2-3
**Introduction**			
Background/rationale	2	Explain the scientific background and rationale for the investigation being reported	4-8
Objectives	3	State specific objectives, including any prespecified hypotheses	8
**Methods**			
Study design	4	Present key elements of study design early in the paper	1-4
Setting	5	Describe the setting, locations, and relevant dates, including periods of recruitment, exposure, follow-up, and data collection	9-11
Participants	6	(*a*) Give the eligibility criteria, and the sources and methods of selection of participants. Describe methods of follow-up	9-11
		(*b*) For matched studies, give matching criteria and number of exposed and unexposed	N/A
Variables	7	Clearly define all outcomes, exposures, predictors, potential confounders, and effect modifiers. Give diagnostic criteria, if applicable	9-13
Data sources/ measurement	8*	For each variable of interest, give sources of data and details of methods of assessment (measurement). Describe comparability of assessment methods if there is more than one group	*9-13*
Bias	9	Describe any efforts to address potential sources of bias	24
Study size	10	Explain how the study size was arrived at	9
Quantitative variables	11	Explain how quantitative variables were handled in the analyses. If applicable, describe which groupings were chosen and why	9-13
Statistical methods	12	(*a*) Describe all statistical methods, including those used to control for confounding	9-13
		(*b*) Describe any methods used to examine subgroups and interactions	9-13
		(*c*) Explain how missing data were addressed	24
		(*d*) If applicable, explain how loss to follow-up was addressed	24
		(*e*) Describe any sensitivity analyses	24
**Results**			

For peer review only

Participants	13*	(a) Report numbers of individuals at each stage of study—eg numbers potentially eligible, examined for eligibility, confirmed eligible, included in the study, completing follow-up, and analysed	9-11
		(b) Give reasons for non-participation at each stage	24
		(c) Consider use of a flow diagram	
Descriptive data	14*	(a) Give characteristics of study participants (eg demographic, clinical, social) and information on exposures and potential confounders	15-16
		(b) Indicate number of participants with missing data for each variable of interest	15-16
		(c) Summarise follow-up time (eg, average and total amount)	2-3,9
Outcome data	15*	Report numbers of outcome events or summary measures over time	
Main results	16	(*a*) Give unadjusted estimates and, if applicable, confounder-adjusted estimates and their precision (eg, 95% confidence interval). Make clear which confounders were adjusted for and why they were included	17-20
		(*b*) Report category boundaries when continuous variables were categorized	N/A
		(*c*) If relevant, consider translating estimates of relative risk into absolute risk for a meaningful time period	N/A
Other analyses	17	Report other analyses done—eg analyses of subgroups and interactions, and sensitivity analyses	
**Discussion**			
Key results	18	Summarise key results with reference to study objectives	20-22
**Limitations**			
Interpretation	20	Give a cautious overall interpretation of results considering objectives, limitations, multiplicity of analyses, results from similar studies, and other relevant evidence	22-24
Generalisability	21	Discuss the generalisability (external validity) of the study results	24-26
**Other information**			
Funding	22	Give the source of funding and the role of the funders for the present study and, if applicable, for the original study on which the present article is based	27-28

*Give information separately for cases and controls in case-control studies and, if applicable, for exposed and unexposed groups in cohort and cross-sectional studies.

Note: An Explanation and Elaboration article discusses each checklist item and gives methodological background and published examples of transparent reporting. The STROBE checklist is best used in conjunction with this article (freely available on the Web sites of PLoS Medicine at http://www.plosmedicine.org/, Annals of Internal Medicine at http://www.annals.org/, and Epidemiology at http://www.epidem.com/). Information on the STROBE Initiative is available at www.strobe-statement.org.

For peer review only
